# Redox-induced umpolung of transition metal carbenes†
†Electronic supplementary information (ESI) available: Characterization data for all new compounds, computational results, single crystal X-ray structure analysis of complexes **4–6**, **8–12**. CCDC 1404570–1404575, 1416513–1416514. For ESI and crystallographic data in CIF or other electronic format see DOI: 10.1039/c5sc02859k



**DOI:** 10.1039/c5sc02859k

**Published:** 2015-09-25

**Authors:** Peng Cui, Vlad M. Iluc

**Affiliations:** a Department of Chemistry and Biochemistry , University of Notre Dame , Notre Dame , IN 46556 , USA . Email: viluc@nd.edu

## Abstract

Metal carbene complexes have been at the forefront of organic and organometallic synthesis and are instrumental in guiding future sustainable chemistry efforts. While classical Fischer and Schrock type carbenes have been intensely studied, compounds that do not fall within one of these categories have attracted attention only recently. In addition, applications of carbene complexes rarely take advantage of redox processes, which could open up a new dimension for their use in practical processes. Herein, we report an umpolung of a nucleophilic palladium carbene complex, [{PC(sp^2^)P}^*t*Bu^Pd(PMe_3_)] ({PC(sp^2^)P}^*t*Bu^ = bis[2-(di-iso-propylphosphino)-4-*tert*-butylphenyl]methylene), realized by successive one-electron oxidations that generated a cationic carbene complex, [{PC(sp^2^)P}^*t*Bu^PdI]^+^, *via* a carbene radical, [{PC˙(sp^2^)P}^*t*Bu^PdI]. An EPR spectroscopic study of [{PC˙(sp^2^)P}^*t*Bu^PdI] indicated the presence of a ligand-centered radical, also supported by the results of reactions with 9,10-dihydroanthracene and PhSSPh. The cationic carbene complex shows electrophilic behavior toward nucleophiles such as NaH, ^*p*^TolNHLi, PhONa, and PMe_3_, resulting from an inversion of the electronic character of the Pd–C_carbene_ bond in [{PC(sp^2^)P}^*t*Bu^Pd(PMe_3_)]. The redox induced umpolung is reversible and unprecedented.

## Abstract 2

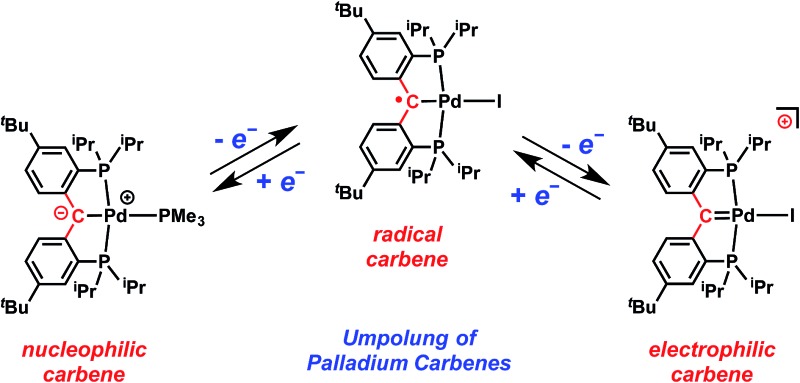
An unprecedented umpolung of a nucleophilic palladium carbene complex was realized by successive one-electron oxidations to generate a cationic carbene complex, which shows electrophilic behavior toward nucleophiles resulting from a polarity inversion of the Pd–C_carbene_ bond.

## Introduction

**Fig. 1:**
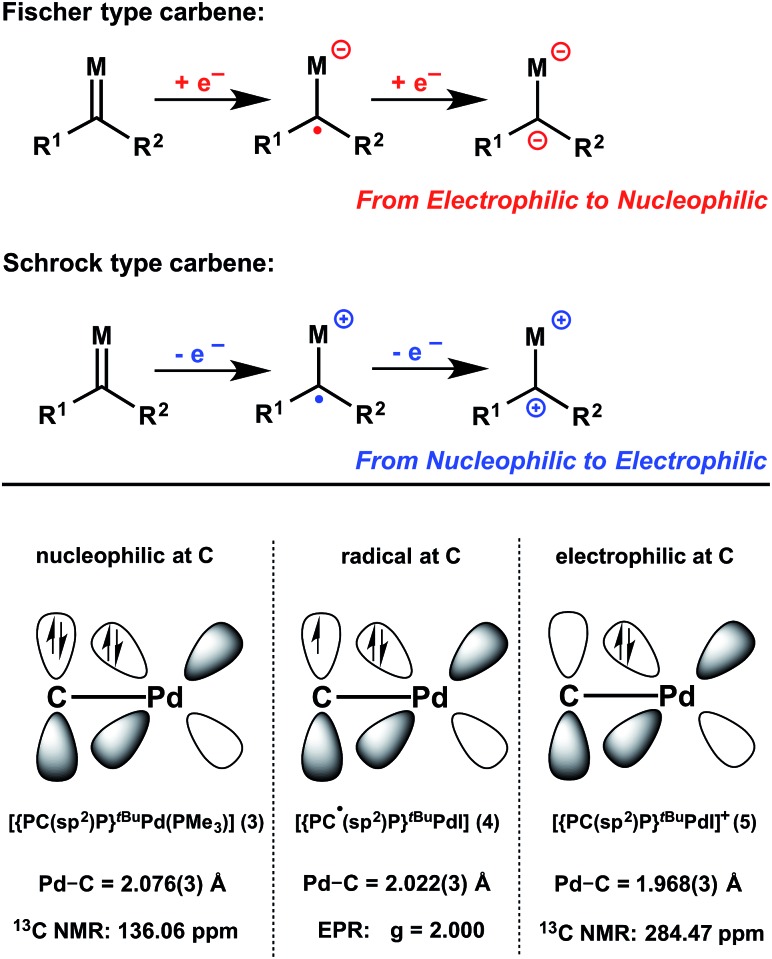
Top: Umpolung of transition metal carbenes by redox reactions. Bottom: Illustration of umpolung of palladium carbene's character by redox processes described in this work.

Transition metal carbene complexes, usually classified as Fischer and Schrock type according to the polarity of the M=C_carbene_ bond,^1^ are among the most extensively studied organometallic species.^2^ While the reactivity of the M=C_carbene_ fragment is generally governed by the electronic properties of the metal center and the substituents of the carbene moiety,^3^ its behavior in response to redox reactions has not been studied in detail.^4^ For example, although the one-electron reduction of a Fischer-type carbene leading to a carbene radical anion, which upon a second one-electron reduction generates a dianion, is known,^5^ the corresponding oxidation processes that could be applied to Schrock-type carbenes (Fig. 1) have not been reported so far. These electron transfer processes cause an umpolung of the original carbene's character, namely the inversion of the M=C_carbene_ bond polarity,^6^ and could open up new applications for these compounds in synthesis.

The development of redox chemistry of transition metal carbenes and reactivity studies of the species generated under redox conditions are still in their infancy,^7^ with only a few examples reported exclusively for Fischer carbenes.^8^ Interestingly, Fischer type carbenes of group 9 metals in the +2 oxidation state exhibited a remarkable radical character, their redox noninnocent behavior being considered crucial to the asymmetric cyclopropanation of olefins catalyzed by Co^II^(porphyrins).^8d–i^ Pioneering work by Casey and later by Cooper *et al.* showed that Fischer type carbene complexes could be reduced to the corresponding radical and dianionic entities.^5^ The latter reacted with CO_2_ to form a malonate, indicating an umpolung from the electrophilic character of the Fischer type carbene to nucleophilic.^5*b*^

As mentioned above, the redox behavior of Schrock type carbenes has not been explored. We reasoned that palladium nucleophilic carbenes^9^ represent good candidates for such a study given the higher stability of late transition metal carbene complexes when compared to that of their early metal counterparts. In addition, the combination between palladium, which can undergo two-electron processes (oxidative addition/reductive elimination), and a redox active ligand, which can undergo sequential one electron processes, has only recently been investigated.^10^ No examples in which a carbene carbon is the site of redox activity are known.

On the other hand, although it is known that palladium carbene species generated from diazo compounds show unique properties and undergo novel transformations, their isolation and characterization is still challenging due to their highly reactive nature.^11^ We previously reported the nucleophilic Pd(ii) carbene complexes [{PC(sp^2^)P}^H^Pd(PR_3_)] (**1**: R = Me; **2**: R = Ph) ([PC(sp^2^)P]^H^ = bis[2-(di-iso-propylphosphino)phenyl]-methylene)^9a,12^ and [{PC(sp^2^)P}^*t*Bu^Pd(PMe_3_)] (**3**, {PC(sp^2^)P}^*t*Bu^ = bis[2-(di-iso-propylphosphino)-4-*tert*-butylphenyl]methylene),^9*b*^ in which the Pd–C_carbene_ bonds are best described as ylide-like, M^+^–C^–^ type, as demonstrated by the reactivity of **1** toward MeI, HCl, MeOH and *para*-toluidine^9*a*^ and also by C–H activation reactions.^13^ The strong nucleophilic character of **1** and **3** was also demonstrated by the rapid Lewis acid/base “quenching” reactions with B(C_6_F_5_)_3_.^9*b*^ As indicated by DFT calculations, the HOMO of **1** is largely localized on the carbene carbon atom, therefore, an oxidation process to induce the loss of electrons might occur from that orbital. Consequently, the one-electron oxidation of **1** with 0.5 equivalents of I_2_ afforded a stable radical complex, [{PC˙(sp^2^)P}^H^PdI], which persisted in solution but dimerized in the solid state. Interestingly, the chloride and bromide congeners are monomers in both phases.^14^

These results prompted us to study the oxidation of the radical species further to the corresponding cations, and therefore to realize an umpolung of a nucleophilic carbene. In order to prevent dimerization and also simplify the synthetic procedure, carbene complex **3** bearing bulky *tert*-butyl groups was employed instead of **1**.^14^ The good solubility provided by the *t*-Bu groups also facilitated reactivity studies. Herein, we report the successive one-electron oxidation of **3** leading to a cationic carbene complex, [{PC(sp^2^)P}^*t*Bu^PdI][BAr^F^_4_] (**5**), *via* a monomeric radical species [{PC˙(sp^2^)P}^*t*Bu^PdI] (**4**). Reactivity studies on both complexes are consistent with their unique electronic properties, and the umpolung of carbene complex **3** was undoubtedly demonstrated by the reactions of **5** with various nucleophiles. Notably, the electron transfer processes to generate the carbene, radical, and cationic species are reversible.

## Results and discussion

### Synthesis and characterization of palladium radical and cationic carbene complexes

**Scheme 1:**
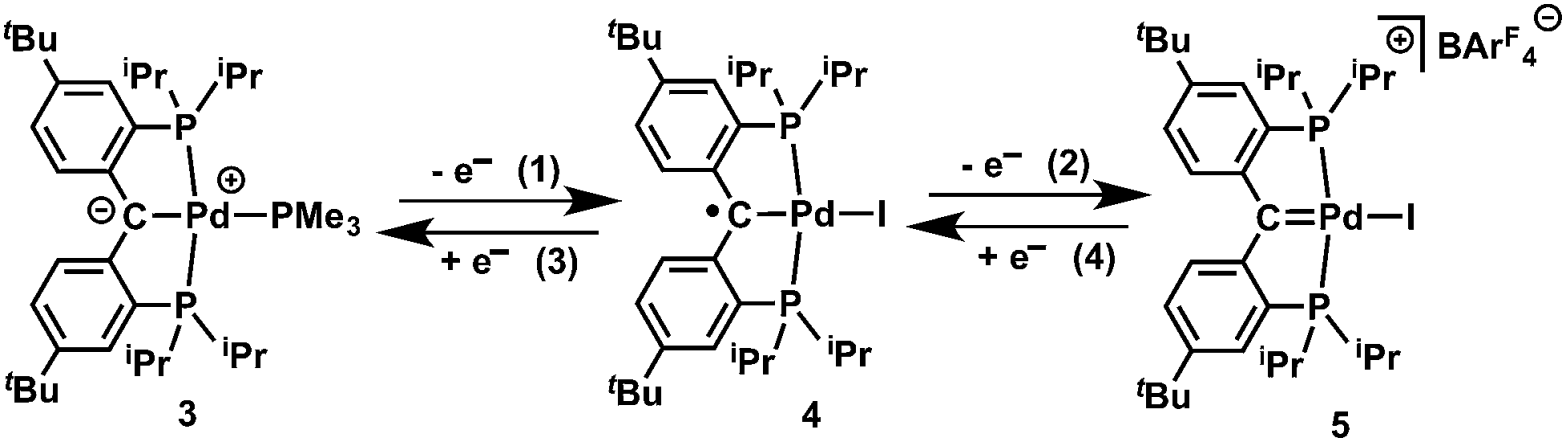
Synthesis of radical and cationic carbene complexes: (**1**) 0.5 I_2_, –35 °C, THF, 30 min; (**2**) [Cp_2_Fe][BAr^F^_4_], –35 °C, diethyl ether, 15 min; (**3**) KC_8_, PMe_3_, THF, 5 min; (**4**) KC_8_, THF, 5 min.

Slow addition of 0.5 equivalents of I_2_ to the dark-brown solution of [{PC(sp^2^)P}^*t*Bu^Pd(PMe_3_)] (**3**) in THF at –35 °C instantly generated a dark-green solution, from which dark-green crystals of [{PC˙(sp^2^)P}^*t*Bu^PdI] (**4**) were isolated in 97% yield (Scheme 1). Complex **4** is silent by both ^1^H and ^31^P{^1^H} NMR spectroscopy. The magnetic susceptibility measurement at 298 K using the Evans method gave an effective magnetic moment *μ*_eff_ of 1.85 *μ*_B_, thus indicating an *S* = 1/2 ground state. The X-band EPR spectrum recorded at 298 K revealed an isotropic signal typical for a carbon centered radical complex with a *g* value of 2; no hyperfine coupling was observed (Fig. S1†). Complex **4** is stable toward water but highly sensitive toward oxygen. Upon exposing the dark-green solution of **4** to air, the color changed immediately to yellow. However, attempting to react **4** with a stoichiometric amount (1 or 0.5 equivalents) of pure oxygen only led to complicated mixtures of products. Interestingly, in contrast to radicals generated *in situ* from Fischer type carbenes,^4,5^ complex **4** is thermally robust: no significant decomposition was observed after heating **4** in C_6_D_6_ at 80 °C for a week, likely a consequence of the radical delocalization over both phenyl rings and of the steric protection from the two *tert*-butyl groups present in **4**.

**Fig. 2:**
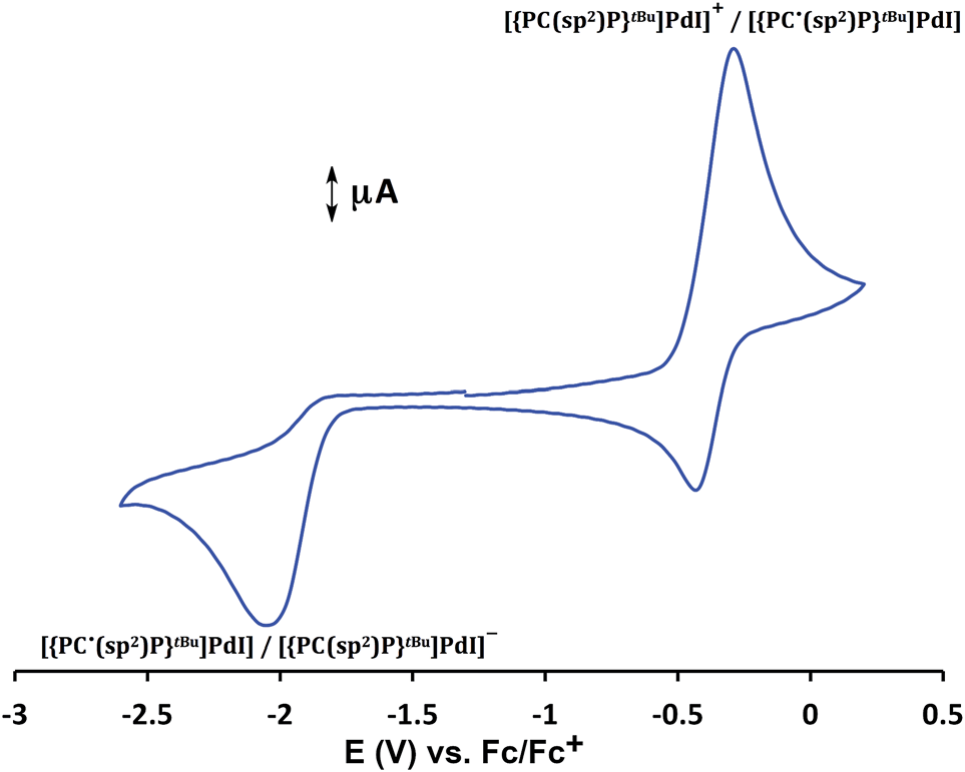
Cyclic voltammetry of **4** in THF (1 × 10^–3^ M), scan rate: 100 mV, electrolyte: [N^*n*^Bu_4_][PF_6_] (0.1 M), ambient temperature, Pt electrode. Referenced to Fc/Fc^+^.

Cyclic voltammetry studies of **4** performed in a THF solution (Fig. 2) showed a quasi-reversible event at *E*_1/2_ = –0.38 V, assigned to [{PC(sp^2^)P}^*t*Bu^PdI]^+^/[{PC˙(sp^2^)P}^*t*Bu^PdI], and an irreversible reduction event at *E* = –2.03 V (*vs.* Fc/Fc^+^), assigned to the reduction to [{PC(sp^2^)P}^*t*Bu^PdI]^–^.

**Fig. 3:**
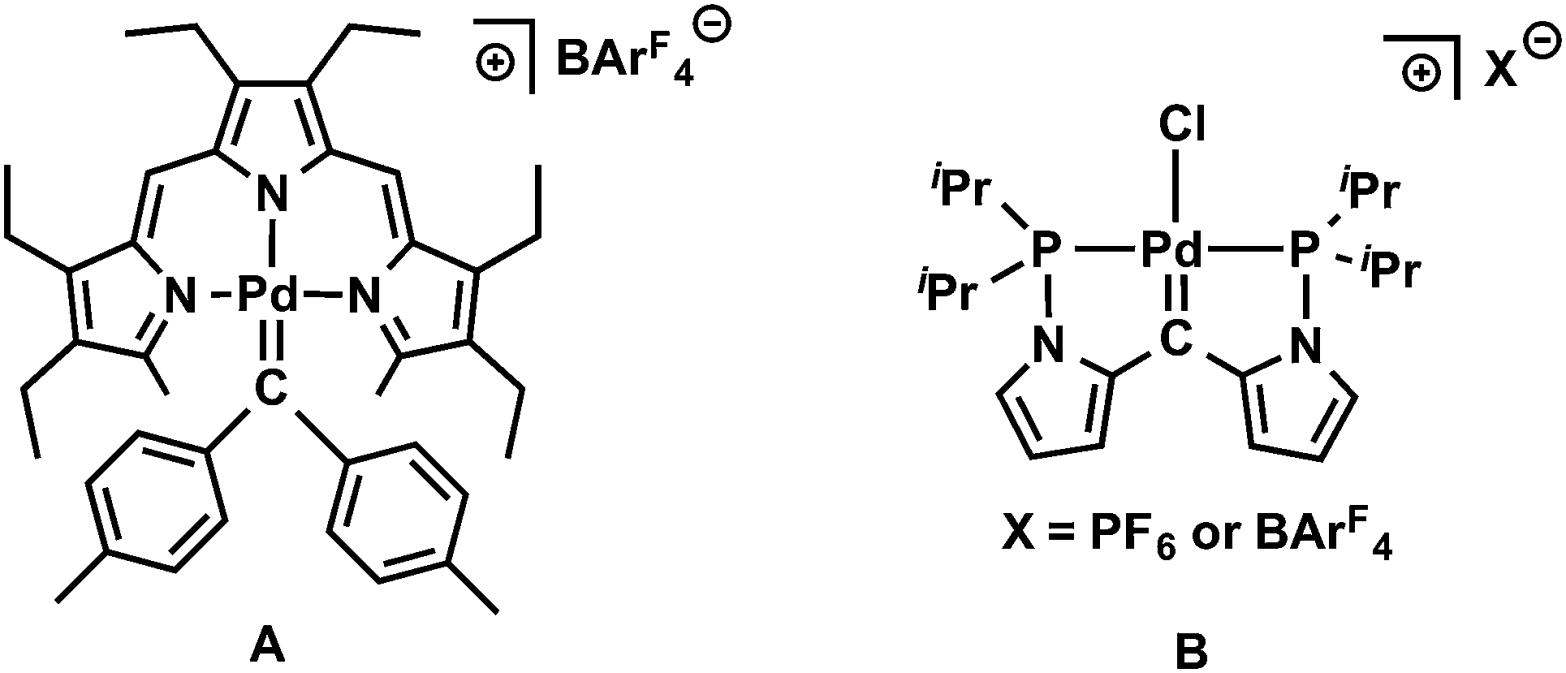
Cationic Pd(ii) carbene complexes synthesized by triflate or hydride abstraction.

Treatment of the dark-green solution of **4** with an equivalent of [Cp_2_Fe][BAr^F^_4_] in diethyl ether at –35 °C instantly formed a dark-red solution, and the dark-red crystalline complex [{PC(sp^2^)P}^*t*Bu^PdI][BAr^F^_4_] (**5**) was isolated in 94% yield (Scheme 1). To the best of our knowledge, only two examples of non-heteroatom-stabilized cationic Pd(ii) carbene complexes are known: complex **A** (Fig. 3) was prepared from a cationic precursor by triflate abstraction with di-*p*-tolyldiazomethane,^15^ while compound **B** was synthesized by hydride abstraction from a PCHP pincer complex;^16^ reactivity studies have not been reported for these species. In addition, cationic alkylidene complexes of other late transition metals are also rare.^17^ Cationic Ir(iii) alkylidene complexes generated either by chloride or hydride abstraction showed electrophilic reactivity toward PMe_3_ and LiAlH_4_ to form the phosphonium ylide and the alkyl species, respectively.^18^ A cationic Pt(ii) alkylidene complex synthesized by hydride abstraction also showed similar reactivity toward Lewis bases such as DMAP (4-dimethylaminopyridine).^19^ It is worth mentioning that, unlike the synthesis of the previously reported cationic carbene complexes, the synthesis of **5** is based on a redox process.

Due to its ionic nature, **5** is only soluble in ethers and chlorinated solvents. The ^1^H NMR spectrum of **5** in CDCl_3_ indicates a *C*_2_ symmetric species. The ^31^P{^1^H} NMR spectrum shows one sharp singlet at *δ* = 72.04 ppm, which shifted to lower field compared to that of 61.86 ppm in **3**. The counter anion [BAr^F^_4_]^–^ was confirmed by singlets at *δ* = –6.62 and –65.79 ppm in the ^11^B{^1^H} and ^19^F{^1^H} NMR spectra, respectively. The characteristic signal for the carbenium carbon was observed at *δ* = 284.47 ppm as a singlet in the ^13^C{^1^H} NMR spectrum, which is significantly shifted to lower field compared to the corresponding values of 136.06 ppm for **3**, –18.3 ppm for [Cy_2_P(S)CP(S)Ph_2_]Pd(PPh_3_),^9*d*^ and 189.6 ppm for the cationic carbene complex [P_2_C=PdCl]X (**B**, Fig. 3).^16^ However, this value is close to that of 313.4 ppm for [(Trpy)Pd=C(*p*-Tol)_2_][BAr^F^_4_] (Trpy = 3,4,8,9,13,14-hexaethyl-2,15-dimethyltripyrrinato, **A**, Fig. 3).^15^

**Fig. 4:**
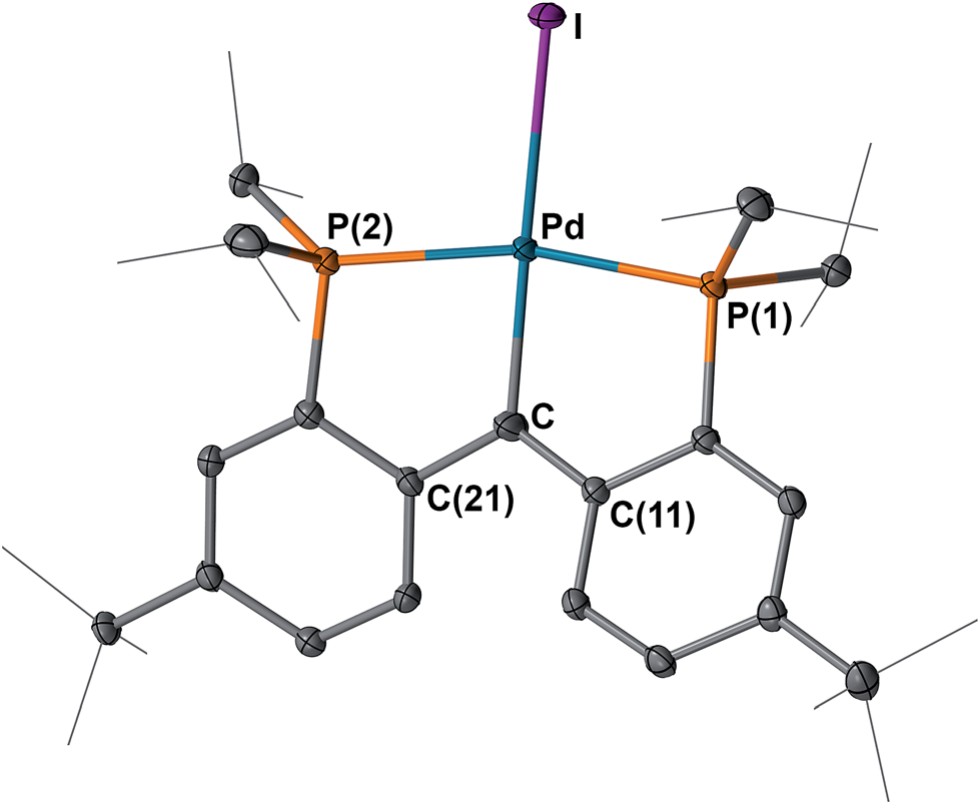
Molecular structure of **4** with displacement parameters at 50% probability level. Hydrogen atoms are omitted for clarity. Selected distances (Å) and angles (degree): Pd–C = 2.022(3), Pd–P(1) = 2.2880(7), Pd–P(2) = 2.2931(8), Pd–I = 2.675(5), P(1)–Pd–P(2) = 163.13(3), Pd–C–C(11) = 118.5(2), Pd–C–C(21) = 118.8(2), C(11) –C–C(21) = 122.5(2).

**Fig. 5:**
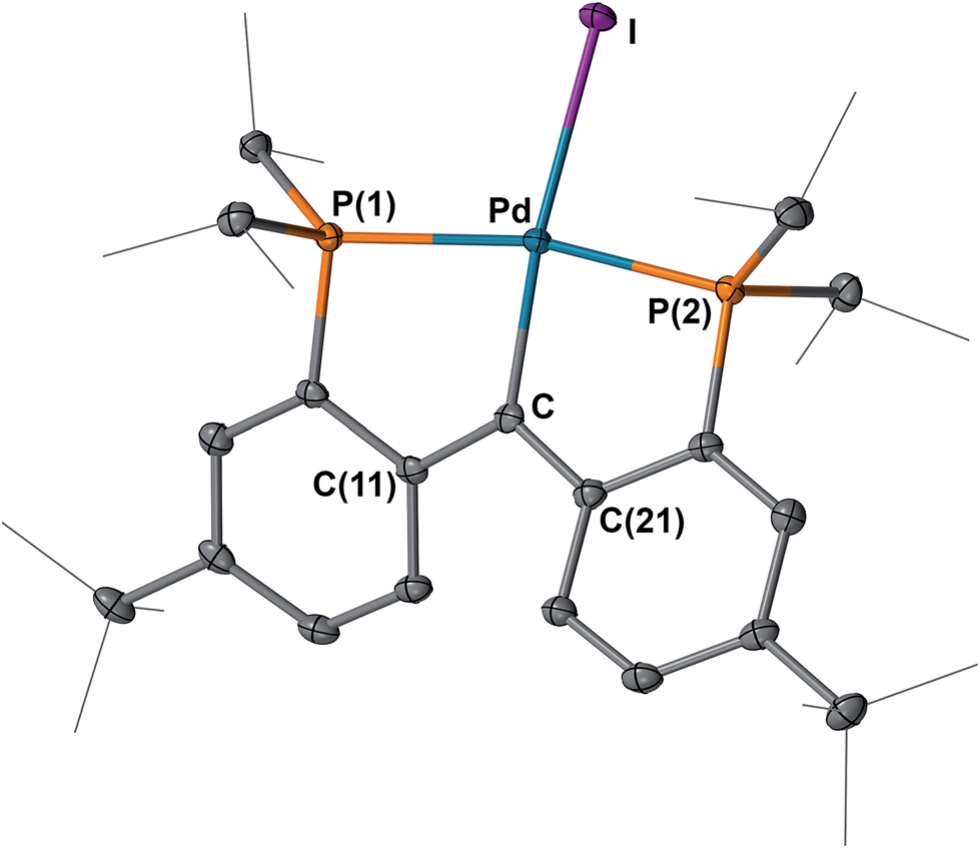
Molecular structure of **5** with displacement parameters at 50% probability level. Hydrogen atoms and the counter anion are omitted for clarity. Selected distances (Å) and angles (degree): Pd–C = 1.968(3), Pd–P(1) = 2.3161(7), Pd–P(2) = 2.2884(7), Pd–I = 2.6255(3), P(1)–Pd–P(2) = 164.98(3), Pd–C–C(11) = 117.59(19), Pd–C–C(21) = 121.2(2), C(11)–C–C(21) = 120.9(2).

The structures of **4** and **5** were unambiguously determined by single crystal X-ray diffraction studies. As shown in Fig. 4 and 5, both complexes contain square-planar palladium centers bound to the sp^2^ hybridized backbone carbons (the sum of the angles at C is 359.8° for **4** and 359.7° for **5**). The Pd–C_carbene_ distance of 2.076(3) Å in **3** contracts to 2.022(3) Å in **4** and further to 1.968(3) Å in **5**. The Pd–C_carbene_ distance of 1.968(3) Å in **5** is comparable to that of 1.999(4) Å in [P_2_C=PdCl][PF_6_] (**B**, Fig. 3).^16^ Although the Pd–C_carbene_ bond in **3** is better described as a single bond,^9a,9b,14,20^ the contraction observed for **4** and **5** can be attributed to the increased bond order between the metal center and the carbene carbon, achieved by the sequential removal of electrons from the π* antibonding orbital (*vide infra*). This trend also indicated a change of the electronic property of this atom from an anion in **3** to a cation in **5** *via* the radical in **4**. Therefore, the isolation of **4** and **5** is remarkable and represents the first example of successive oxidations of a transition metal nucleophilic carbene leading to a well-defined radical and cation, with all three complexes characterized by X-ray crystallography.

### DFT calculations

**Fig. 6:**
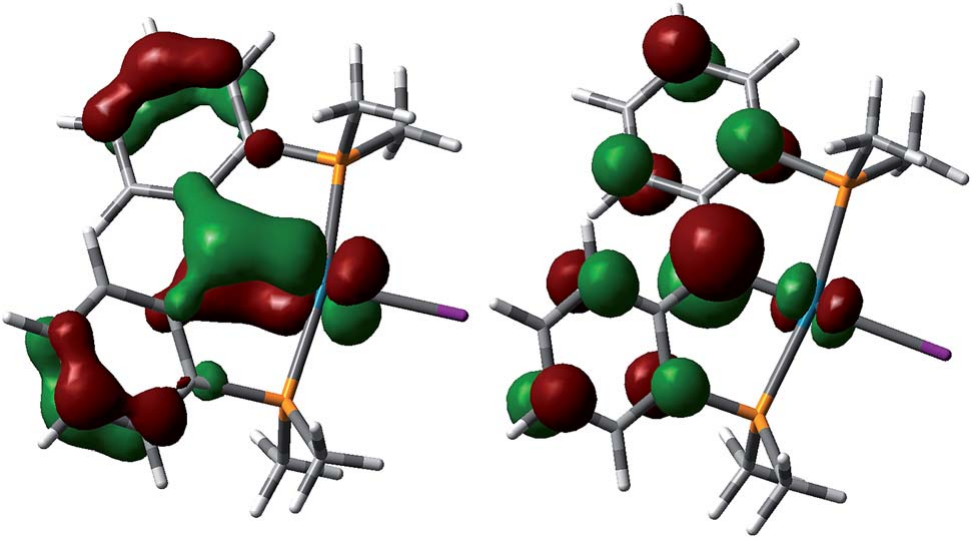
Molecular orbitals for the Pd–C π interaction for **5**. Left: HOMO–11, right: LUMO.

DFT calculations were performed on a model of the cationic carbene **5** (Fig. 6). In contrast to the antibonding character of HOMO observed for **3**, a similar antibonding π type interaction was found for the LUMO of **5** and the SOMO of **4**, resulting from the successive removal of electrons from the HOMO of **3** that is largely localized on the carbene carbon. The bonding component of this π bond was found in HOMO–11 for **5**. Therefore, consistent with the observed contraction of the Pd–C_carbene_ distances from **3** (2.076(3) Å) to **5** (1.968(3) Å), the frontier molecular orbital analysis also indicates an increase of the Pd–C_carbene_ bond order for **5**. This trend was observed in the calculated Mayer bond indices^21^ of the Pd–C_carbene_ (0.82 for **3** and 0.95 for **5**) as well as the Wiberg bond indices^22^ (0.91 for **3** and 0.98 for **5**). The calculated charge on the carbene carbon decreases from –0.37 for **3** to –0.19 for **4** and –0.03 for **5**, in agreement with the observed reactivity (*vide infra*).

A successive addition of electrons to the LUMO of **5** should regenerate the carbene radical **4** and, ultimately, the carbene **3**. Treatment of the dark-red THF solution of **5** with KC_8_ (1 eq.) at room temperature immediately led to a dark-green solution of **4**, as confirmed by its reaction with 9,10-dihydroanthracene in C_6_D_6_ to form **6** (*vide infra*). In the presence of PMe_3_, the further reduction of **4** with KC_8_ (1 eq.) in THF also regenerated **3**. Such reversible electron transfer among the carbene species **3**, **4**, and **5** allowed us to tune their electronic properties by redox methods (Fig. 1).^4^ Accordingly, treatment of cation **5** with 1 eq. of carbene **3** in diethyl ether immediately generated a dark green solution containing carbene radical **4** and the cationic radical complex [{PC˙(sp^2^)P}^*t*Bu^Pd(PMe_3_)][BAr^F^_4_] (Fig. S41†), which were confirmed by their hydrogen atom abstraction reactions with 9,10-dihydroanthracene (*vide infra*).

### Reactivity of palladium carbene radical complex

**Scheme 2:**
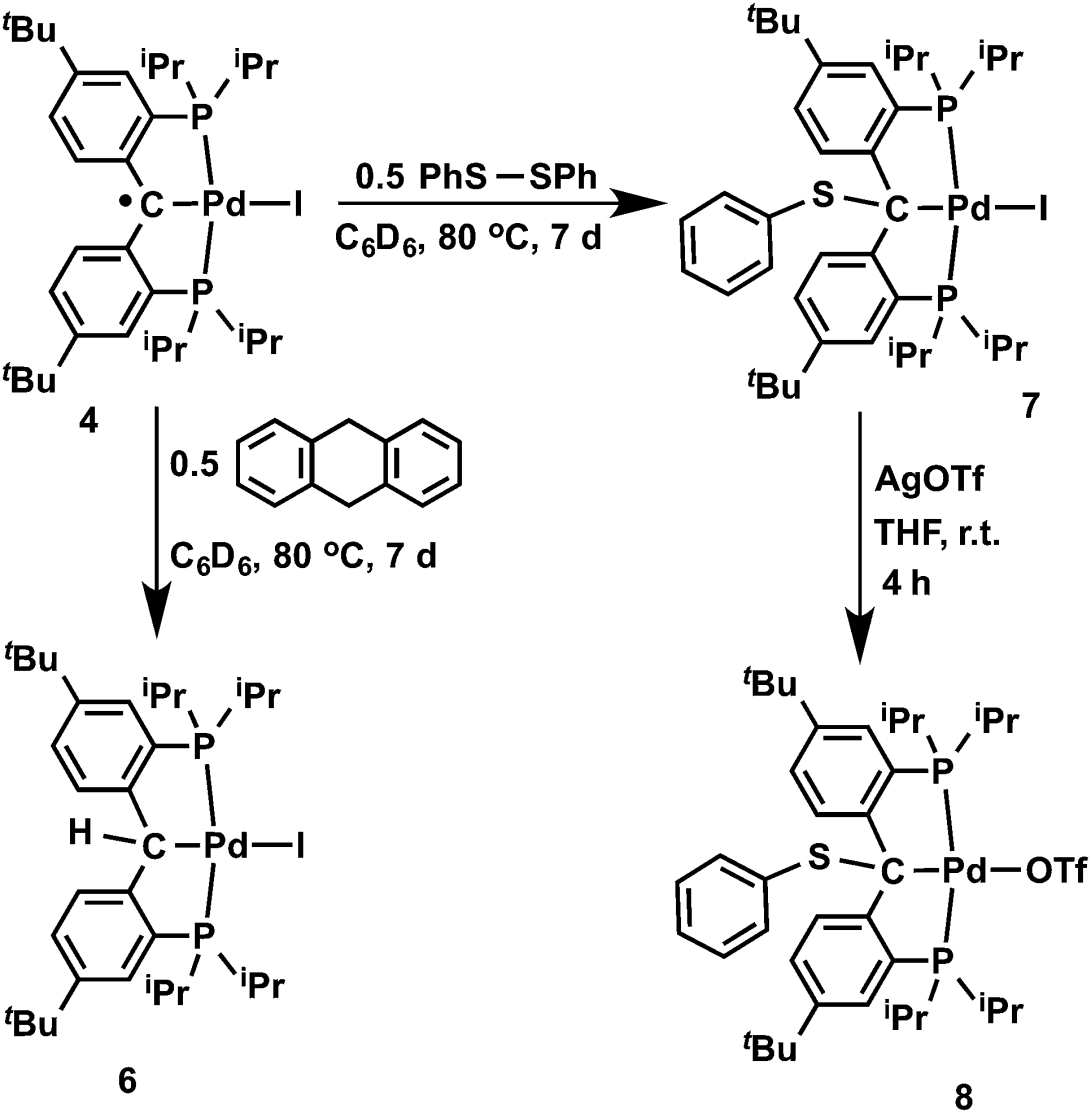
Reactions of carbene radical **4**.

The carbene radical complex **4** is expected to undergo ligand centered radical-type reactions, such as hydrogen atom abstraction.^14^ Despite this prediction, heating a C_6_D_6_ solution of **4** with ^*n*^Bu_3_SnH at 80 °C for one week did not produce the hydrogen atom abstraction product, and the color of the solution remained dark green. However, **4** reacted slowly reacted with 0.5 equivalents of 9,10-dihydroanthracene under similar conditions to generate a bright yellow solution, from which the expected hydrogen abstraction product [{PC(sp^3^)HP}^*t*Bu^PdI] (**6**) was isolated as an orange crystalline solid in 81% yield, after recrystallization from *n*-pentane (Scheme 2). The formation of the by-product anthracene was also confirmed by NMR spectroscopy.

**Fig. 7:**
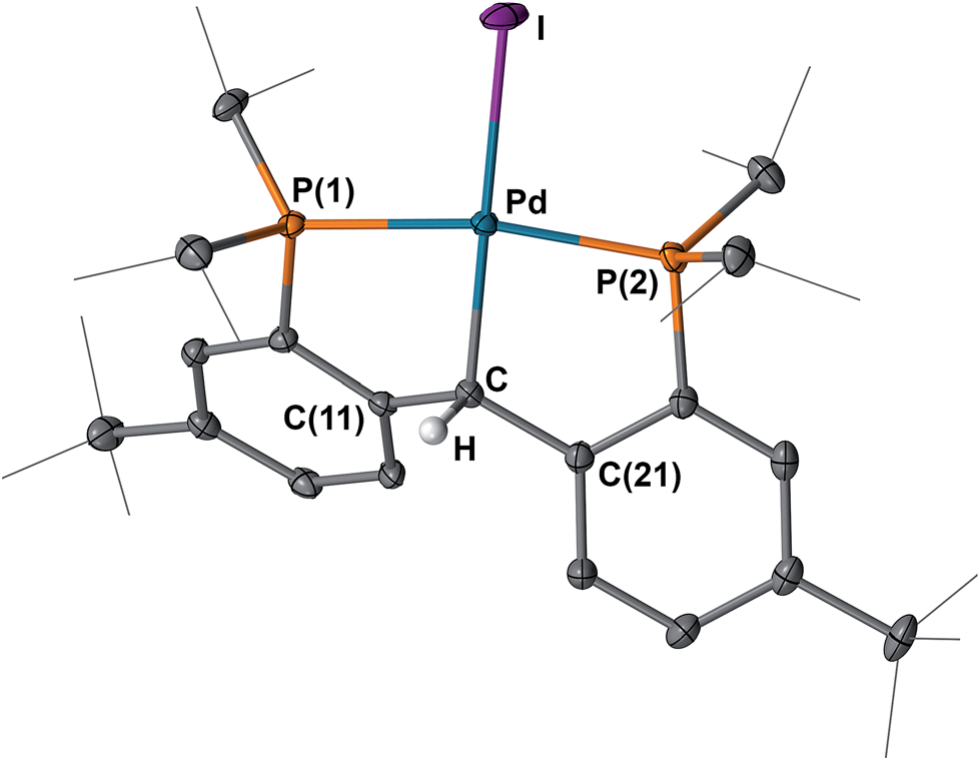
Molecular structure of **6** with displacement parameters at 50% probability level. Most hydrogen atoms are omitted for clarity. Selected distances (Å) and angles (degree): Pd–C = 2.094(2), Pd–P(1) = 2.3123(7), Pd–P(2) = 2.2698(7), Pd–I = 2.6763(3), P(1)–Pd–P(2) = 165.40(2), Pd–C–C(11) = 109.32(16), Pd–C–C(21) = 115.91(16), C(11)–C–C(21) = 116.7(2).

The ^1^H NMR spectrum of **6** in C_6_D_6_ showed *C*_s_ symmetry as observed for its chloride analogue [{PC(sp^3^)HP}^*t*Bu^PdCl].^9*b*^ The benzylic proton of **6** was observed at *δ* = 6.41 ppm as a singlet, while the benzylic carbon was observed at *δ* = 58.35 ppm as a triplet (^2^ *J*_PC_ = 4.4 Hz) in the corresponding ^13^C{^1^H} NMR spectrum. Both values are slightly downfield shifted compared to those of the chloride analogue (*δ* = 6.21 and 50.59 ppm for the ^1^H and ^13^C{^1^H} NMR spectra, respectively).^9*b*^ In the ^31^P{^1^H} NMR spectrum, only one sharp singlet at *δ* = 51.25 ppm was observed. The crystal structure of **6** is analogous to that of [{PC(sp^3^)HP}^*t*Bu^PdCl] (Fig. 7), in that both contain an sp^3^ hybridized backbone carbon atom. The Pd–C_backbone_ distance of 2.094(2) Å is also close to that of 2.078(2) Å in [{PC(sp^3^)HP}^*t*Bu^PdCl],^9*b*^ but longer than the Pd–C_carbene_ distance of 2.022(3) Å for **4**.

**Fig. 8:**
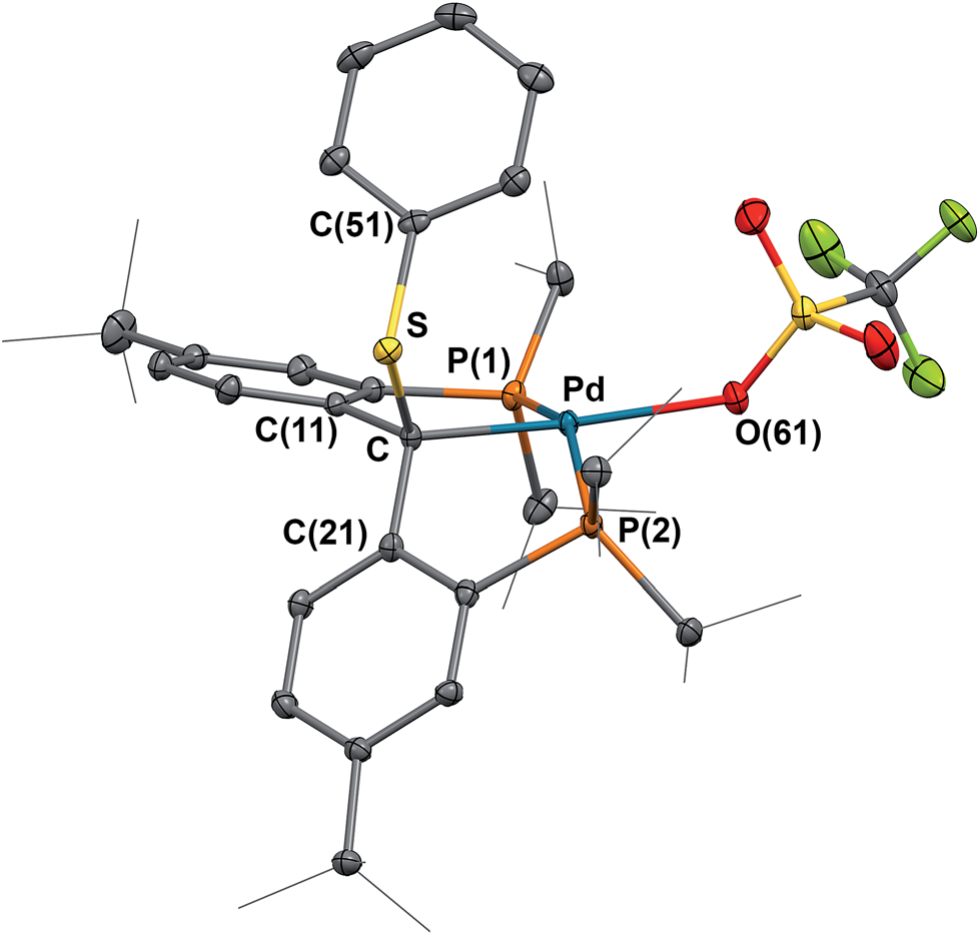
Molecular structure of **8** with displacement parameters at 50% probability level. Hydrogen atoms and the solvent molecule are omitted for clarity. Selected distances (Å) and angles (degree): Pd–C = 2.051(2), Pd–P(1) = 2.2745(6), Pd–P(2) = 2.3554(6), Pd–O(61) = 2.1895(17), S–C = 1.840(2), P(1)–Pd–P(2) = 154.14(2).

Coupling of the radical in **4** with a thiyl radical occurs in the reaction with diphenyl disulfide (Scheme 2). Albeit slow, heating **4** with 0.5 equivalents of PhSSPh in C_6_D_6_ at 80 °C for 7 days formed [{PC(sp^3^)(SPh)P}^*t*Bu^PdI] (**7**), which was isolated as a yellow waxy solid in 94% yield. The ^1^H NMR spectrum of **7** in C_6_D_6_ showed *C*_s_ symmetry, indicating an sp^3^ hybridized backbone carbon. No benzylic proton was observed and the quaternary backbone carbon showed a triplet at *δ* = 79.58 (^2^ *J*_PC_ = 6.1 Hz) in the ^31^C{^1^H} NMR spectrum, both facts being consistent with a C–S bond formation between the carbene radical of **4** and the [PhS]˙ radical. The ^31^P{^1^H} NMR spectrum showed only a sharp singlet at *δ* = 48.59 ppm. However, all attempts to crystallize **7** failed due to its high solubility in hydrocarbons. Therefore, complex **7** was treated with an equivalent of AgOTf in THF,^23^ and the expected complex [{PC(sp^3^)(SPh)P}^*t*Bu^Pd(OTf)] (**8**) was obtained as yellow blocks in 61% yield after recrystallization from diethyl ether. The crystal structure of **8** was determined by X-ray diffraction (Fig. 8), showing the sp^3^ hybridized backbone carbon bound to a PhS group. The phenyl ring of the PhS group is pointing somewhat toward the triflate substituent with the corresponding dihedral angles between the plane of the phenyl ring and the plane defined by O, Pd, C_backbone_ and S being 73.49°.

According to the standard redox potential of PhSSPh (–1.7 V *vs.* SCE, –2.1 V *vs.* Fc/Fc^+^)^24^ and the observed value for the [{PC˙(sp^2^)P}^*t*Bu^PdI]/[{PC(sp^2^)P}^*t*Bu^PdI]^+^, redox couple (–0.38 V *vs.* Fc/Fc^+^), it is less likely that the carbene radical **4** was oxidized by PhSSPh to the carbene cation [{PC(sp^2^)P}^*t*Bu^PdI]^+^ which reacted with the [PhS]^–^ anion to form **7**. Therefore, compound **7** was probably formed by direct radical coupling between the carbene radical and [PhS˙] generated by the homolysis of PhSSPh.

**Scheme 3:**
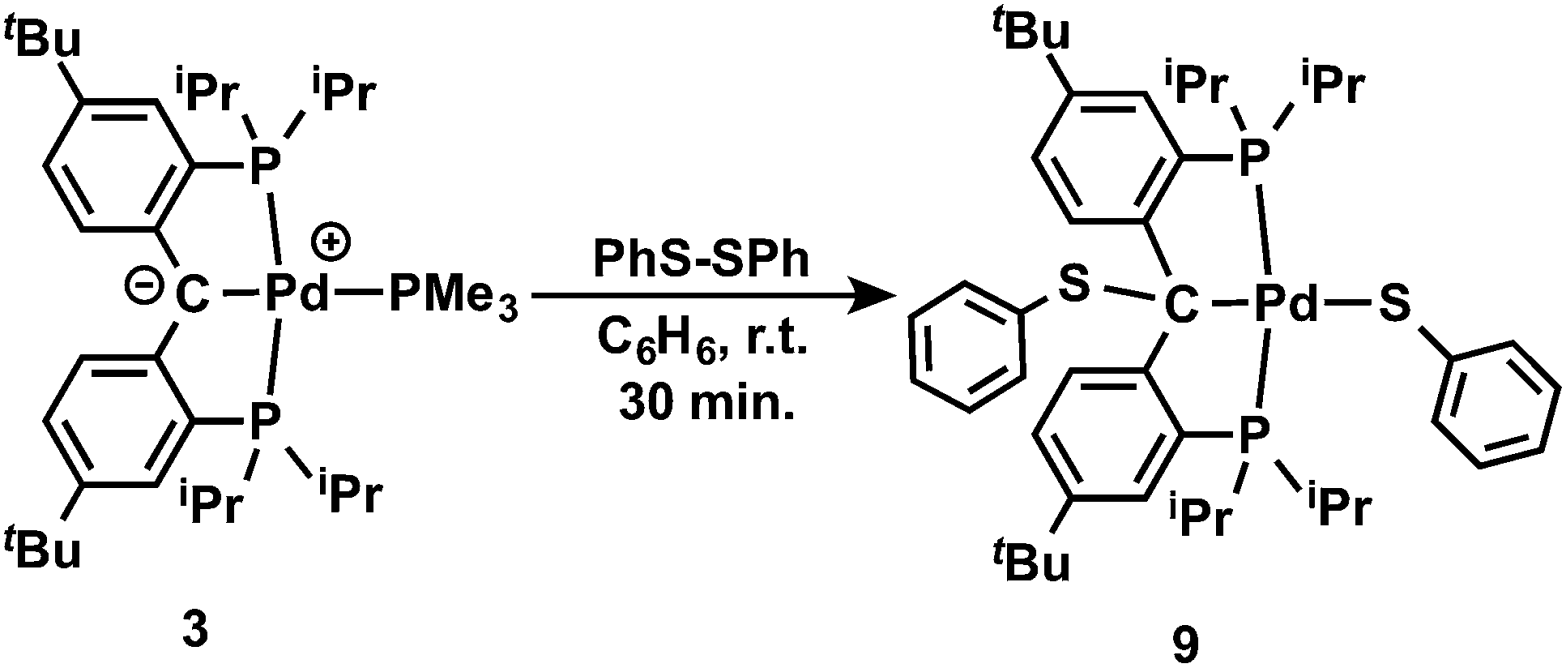
Reaction of carbene **3** with PhS-SPh.

**Fig. 9:**
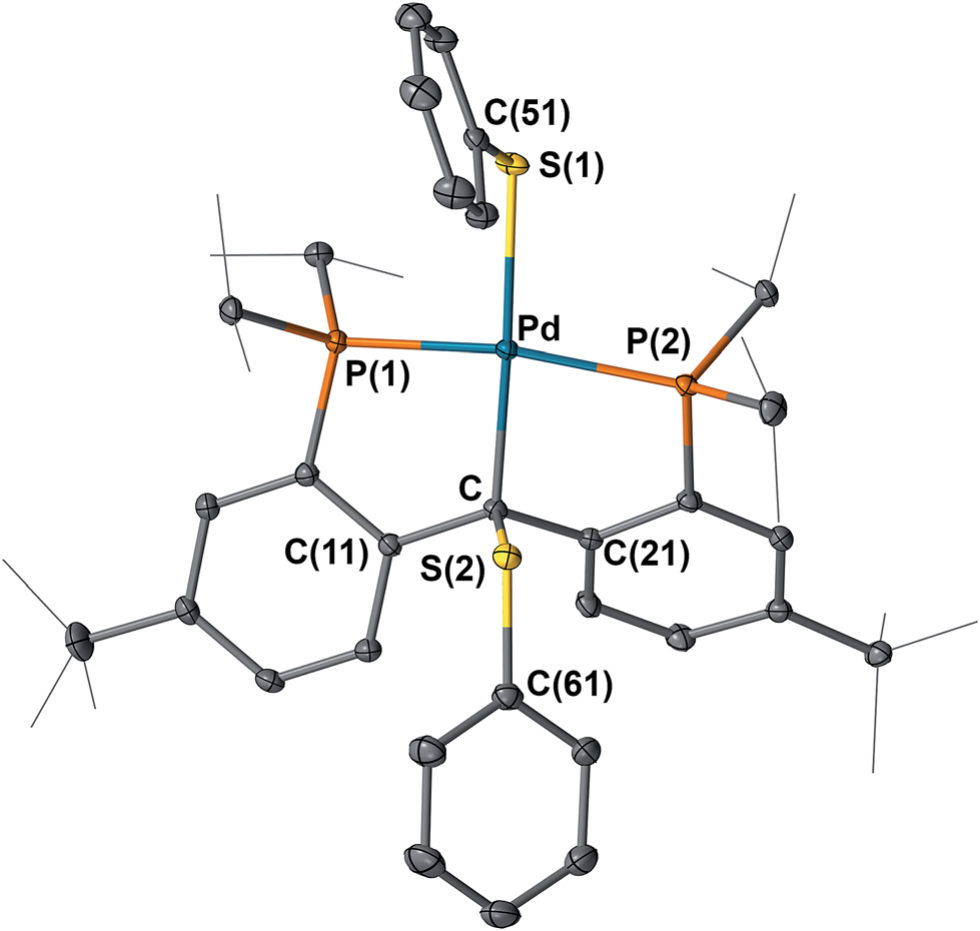
Molecular structure of **9** with displacement parameters at 50% probability level. Hydrogen atoms and the solvent molecule are omitted for clarity. Selected distances (Å) and angles (degree): Pd–C = 2.119(2), Pd–P(1) = 2.2666(7), Pd–P(2) = 2.3449(8), Pd–S(1) 2.3980(7), C–S(2) 1.852(2), P(1)–Pd–P(2) = 164.32(2).

It is also interesting to note that the carbene complex **3** can be readily oxidized by PhSSPh. Treatment of **3** with an equivalent of PhSSPh in C_6_H_6_ immediately generated a bright yellow solution at room temperature, from which the yellow crystalline solid of [{PC(sp^3^)(SPh)P}^*t*Bu^Pd(SPh)] (**9**) was isolated in quantitative yield after recrystallization from *n*-pentane (Scheme 3). The ^1^H NMR spectrum of **9** in C_6_D_6_ also showed *C*_s_ symmetry. No benzylic proton was observed and the corresponding quaternary backbone carbon showed a triplet at *δ* = 74.78 (^2^ *J*_PC_ = 3.2 Hz) in the corresponding ^31^C{^1^H} NMR spectrum. Only a sharp singlet at *δ* = 44.26 ppm was observed in the ^31^P{^1^H} NMR spectrum. The structure of **9** was further confirmed by X-ray diffraction studies. As shown in Fig. 9, complex **9** contains two PhS moieties, one bound to the sp^3^ hybridized carbon backbone and the other to the palladium center. Unlike **8**, the phenyl ring of the PhS group on the backbone carbon points away from the Pd center.

When carbene **3** was treated with only 0.5 equivalents of PhSSPh in C_6_D_6_, compound **9** and unreacted **3** were observed by ^1^H and ^31^P NMR spectra; which upon the addition of another 0.5 equivalents of PhSSPh, the mixture led to **9**. It is worth noting that a radical complex [{PC˙(sp^2^)P}^*t*Bu^PdSPh] (**C**) was not formed under these conditions, but it could be an intermediate species that generates **9** by rapid coupling with the [PhS]˙ radical. This intermediate, **C**, can be generated by an one-electron transfer from carbene **3** to PhSSPh, however, direct addition of PhSSPh across Pd^+^–C^–^_carbene_ moiety or oxidative addition to palladium followed by the migration of the PhS^–^ moiety to the carbene carbon to form **9**, cannot be ruled out.

### Reactivity of palladium cationic carbene complex

**Scheme 4:**
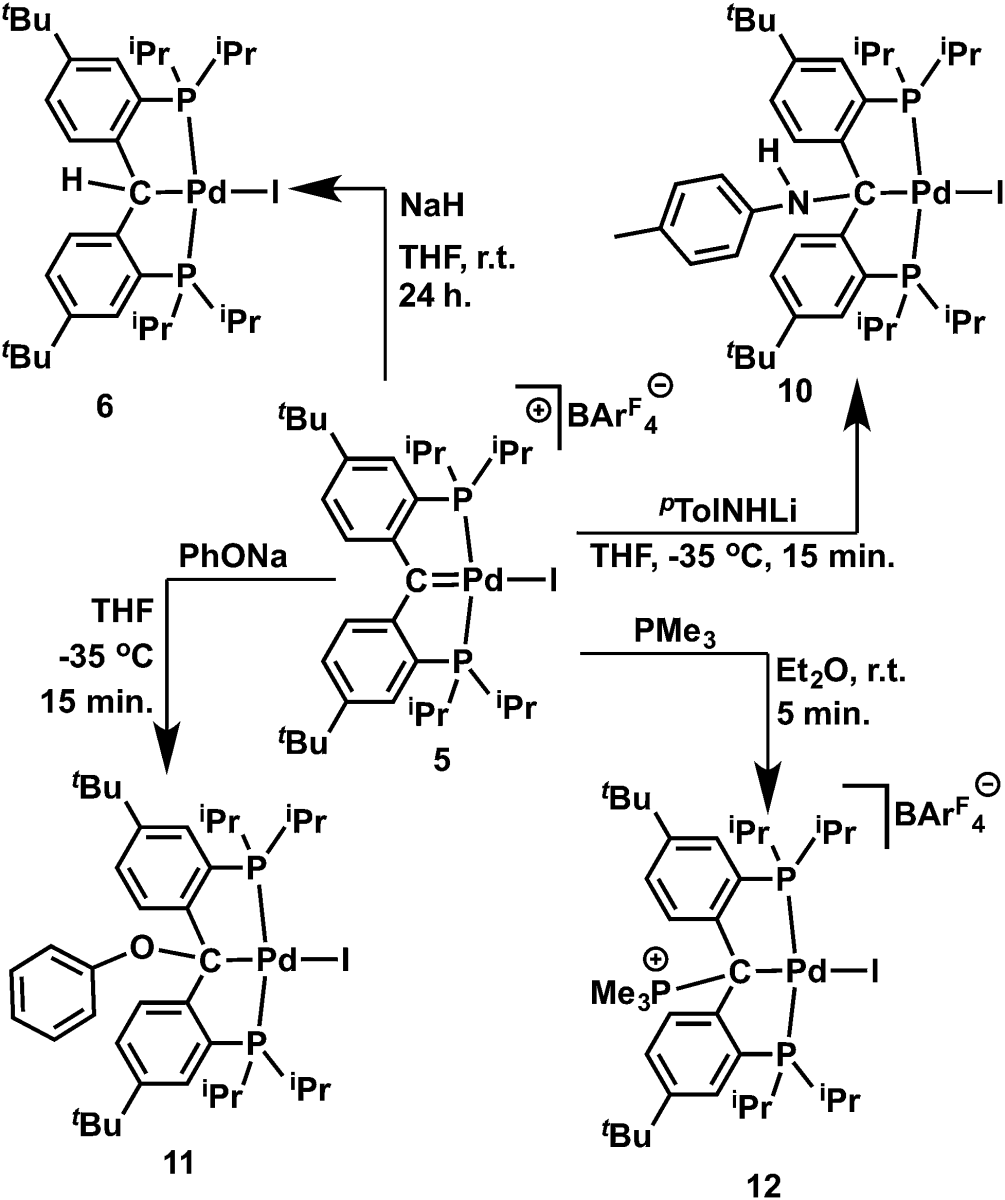
Reactions of the cationic carbene **5** with nucleophiles.

The cationic carbene complex **5** is expected to be electrophilic, therefore its reactivity toward various nucleophiles was studied. We had previously isolated complex **6** from a hydrogen atom abstraction reaction with the carbene radical **4**, and thus assumed that a nucleophilic attack on the cationic carbene **5** by a H^–^ nucleophile would also produce **6**. Treatment of a dark-red solution of **5** with an equivalent of NaH in THF at room temperature gradually generated a bright yellow solution within 24 hours. Both ^1^H and ^31^P{^1^H} NMR spectra showed that the **6** was formed in quantitative yield together with the by-product Na[BAr^F^_4_] (Scheme 4). Since the cationic Ir(iii) metallacyclic alkylidene complex was reported to react with LiAlH_4_ even at low temperature,^18*b*^ the slow reaction with **5** was attributed to the poor nucleophilicity of NaH, but also to its poor solublility in THF.

**Fig. 10:**
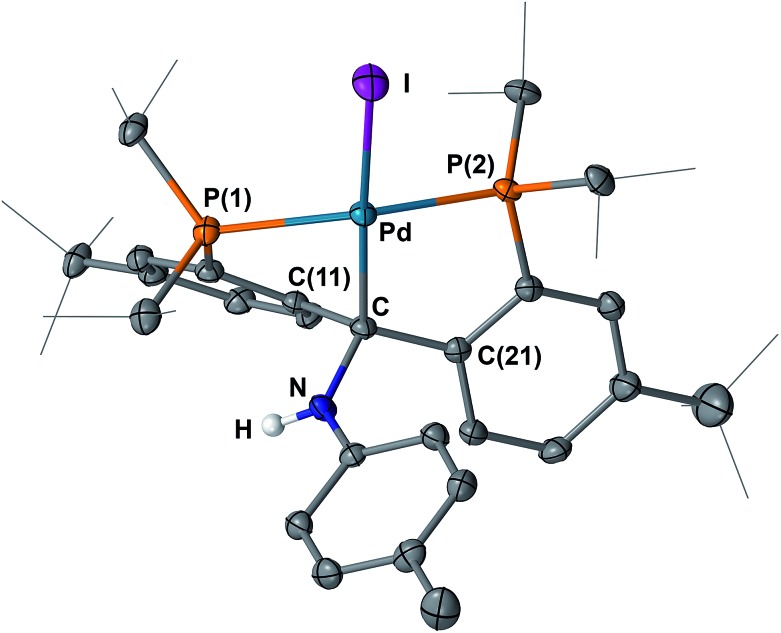
Molecular structure of **10** with displacement parameters at 50% probability level. Most hydrogen atoms are omitted for clarity. Selected distances (Å) and angles (degree): Pd–C = 2.116(3), Pd–P(1) = 2.3206(7), Pd–P(2) = 2.2609(8), Pd–I = 2.6903(3), C–N = 1.461(3), P(1)–Pd–P(2) = 155.81(3).

**Fig. 11:**
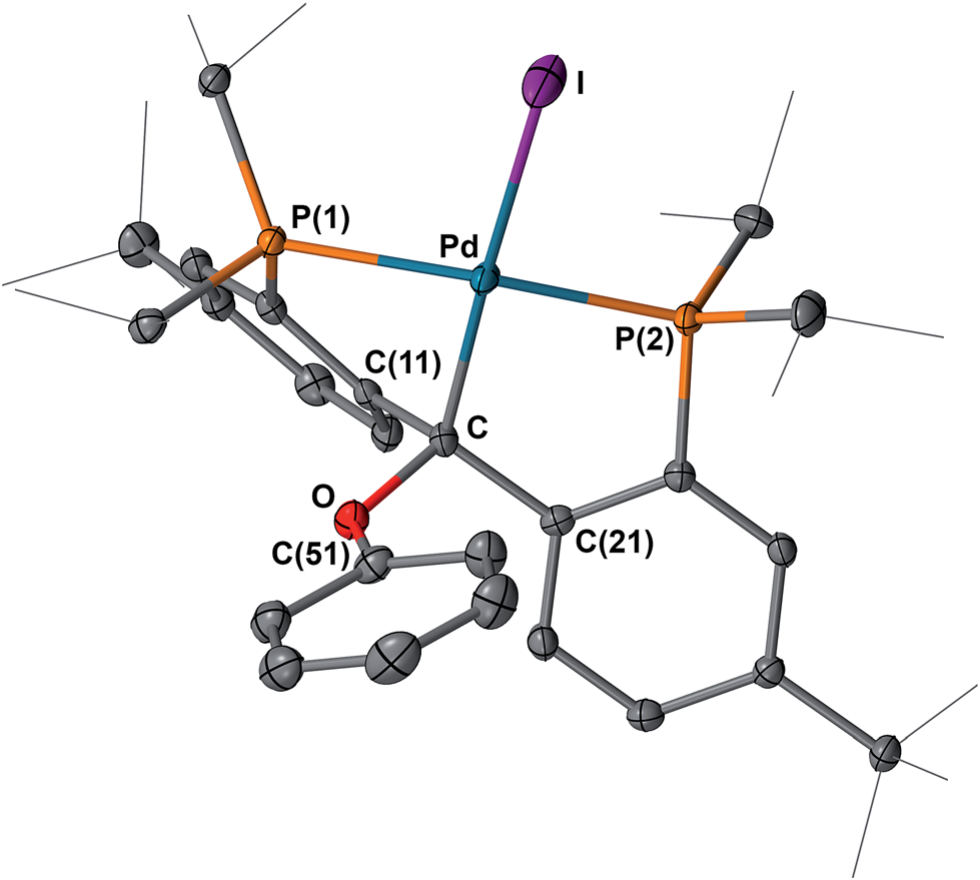
Molecular structure of one of the two crystallographically independent molecules of **11** with displacement parameters at 50% probability level. Hydrogen atoms are omitted for clarity. Selected distances (Å) and angles (degree): Pd–C = 2.089(4), Pd–P(1) = 2.3282(10), Pd–P(2) = 2.2741(11), Pd–I = 2.6746(4), C–O = 1.477(5), P(1)–Pd–P(2) = 157.20(4).

We then studied the reaction of **5** with the anionic nucleophiles LiNH^*p*^Tol and PhONa, since both salts are readily soluble in THF. Slow addition of a THF solution of LiNH^*p*^Tol to the dark-red solution of **5** at –35 °C immediately formed a brownish-green solution. Similarly, the addition of PhONa to **5** resulted in the formation of a bright-yellow solution. The expected products [{PC(sp^3^)(NH^*p*^Tol)P}^*t*Bu^PdI] (**10**) and [{PC(sp^3^)(OPh)P}^*t*Bu^PdI] (**11**) were isolated in moderate yields (Scheme 4) as yellow crystalline solids, which are soluble in aliphatic solvents. Multiple recrystallizations were performed to remove the Na[BAr^F^_4_] salt. The structures of **10** and **11** were confirmed by X-ray diffraction studies. As shown in Fig. 10 and 11, both contain a square planar palladium center anchored with the sp^3^ hybridized carbon. The hydrogen atom of the NH group was located in the electron density map. The short N–C_*ipso*_ distance of 1.378(4) Å indicated a delocalization of the lone pair of the nitrogen atom to the phenyl ring of the *para*-toluidine group and the geometry at the nitrogen atom is essentially trigonal planar (sum of the angles of 360°). Interestingly, both phenyl rings of the NH^*p*^Tol and OPh groups point somewhat toward palladium, with the corresponding dihedral angles between the planes of the phenyl rings and the planes defined by I, Pd, C_backbone_ and X (X = N or O) being 64.05° and 58.91°, respectively, which are smaller than that of 73.49° in **8**.

**Fig. 12:**
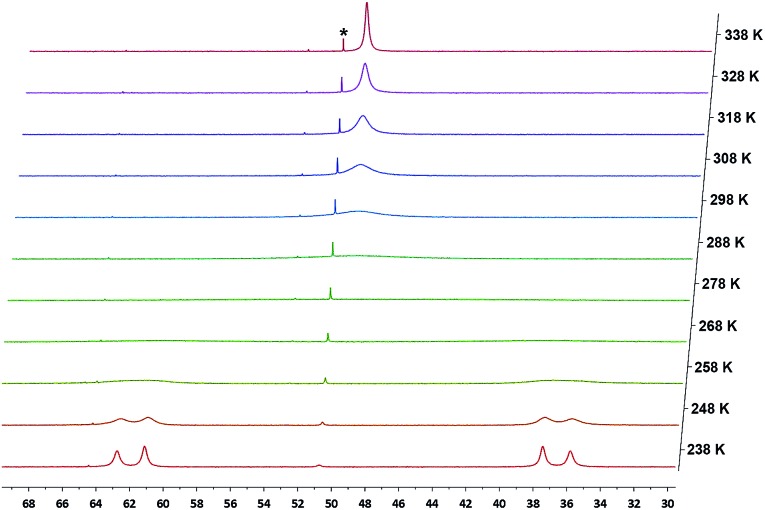
Variable temperature ^31^P{^1^H} NMR spectra of **10** in toluene-d_8_ (*designates a small amount of impurity of **6**).

As expected, the ^1^H NMR spectra of **10** and **11** in C_6_D_6_ showed *C*_s_ symmetry. The proton of the N*H* group in **10** was observed at *δ* = 4.22 ppm as a triplet (^4^ *J*_PH_ = 2.8 Hz). Interestingly, the ^31^P{^1^H} NMR spectra of **10** and **11** at 298 K showed only broad singlets at *δ* = 49.89 (Δ*ν*_1/2_ ≈ 909 Hz) and 51.09 (Δ*ν*_1/2_ ≈ 505 Hz) ppm, respectively. Upon cooling, complex **10** showed coalescence at 278 K, and further splitting to two sharp doublets at 238 K (Fig. 12). For **11**, a slightly lower temperature (268 K) was required to reach coalescence and two sharp doublets were observed at 228 K (Fig. S34†). Both **10** and **11** showed only one sharp singlet in the corresponding ^31^P{^1^H} NMR spectra at 338 K. Since a similarly dynamic behavior was not observed for **8** and **9**, in which both backbone carbons are bound to PhS groups, we attribute the dynamic behavior of **10** and **11** to the relatively short N–C_backbone_ (1.461(3) Å) and O–C_backbone_ (1.477(5) Å) distances compared to the S–C_backbone_ distance of 1.840(2) in **8** and 1.852(2) Å in **9**, causing a higher energy barrier for the conformational exchange of the ligand framework and the rotation of the ^*p*^TolNH and PhO groups. The activation parameters obtained from Eyring plots for **10** are: Δ*G* ^‡^ = 11.1 ± 0.2 kcal mol^–1^ (298 K), Δ*H* ^‡^ = 13.9 ± 0.4 kcal mol^–1^ and Δ*S* ^‡^ = 10 ± 2 cal (mol^–1^ K); and for **11** are: Δ*G* ^‡^ = 10.7 ± 0.4 kcal mol^–1^ (298 K), Δ*H* ^‡^ = 11.8 ± 0.4 kcal mol^–1^ and Δ*S* ^‡^ = 4 ± 2 cal (mol^–1^ K).

Although the nucleophiles H^–^, ^*p*^TolNH^–^, and PhO^–^ afforded the expected products **6**, **10**, and **11**, respectively, the reaction of **5** with PhCH_2_K did not lead to an isolable product. Also, the lithium salts MeOLi and PhOLi did not react with **5**, probably due to their weaker nucleophilic character compared to that of the sodium salt PhONa.

A neutral nucleophile such as PMe_3_ also reacts instantly with **5** in diethyl ether to form a bright-yellow solution; yellow crystals of [{PC(sp^3^)(PMe_3_)P}^*t*Bu^PdI][BAr^F^_4_] (**12**) were isolated in quantitative yield by the diffusion of *n*-pentane into a fluorobenzene solution at room temperature. Compound **12** is only soluble in etheral and chlorinated solvents. Its ^1^H NMR spectrum in CDCl_3_ showed *C*_s_ symmetry as was also observed for compounds **6–11**. The protons of the PMe_3_ group were observed at *δ* = 1.31 ppm as a doublet (^2^ *J*_HP_ = 11.5 Hz), which correlates with a doublet at *δ* = 13.86 ppm (^1^ *J*_CP_ = 54.3 Hz) in the ^31^C{^1^H} NMR spectrum. In the ^31^P{^1^H} NMR, two sharp singlets at *δ* = 42.17 and 31.01 ppm were observed for the two ^*i*^Pr_2_P phosphines and the PMe_3_ on the backbone carbon, respectively. Both signals remain sharp when the temperature was lowered to 248 K, indicating a lower energy barrier for the rotation of PMe_3_ group compared to those for the ^*p*^TolNH and PhO groups in **10** and **11**. In the ^13^C{^1^H} NMR spectrum, the backbone carbon resonance was observed at *δ* = 63.65 (dt, ^1^ *J*_CP_ = 21.5 Hz, ^2^ *J*_CP_ = 2.4 Hz) ppm, which is significantly shifted to higher field compared to 284.47 ppm observed for **5**, and close to the value of 58.35 ppm in **6**. Thus, the strong donation from PMe_3_ to the carbenium carbon effectively offsets the charge on this atom. The exclusive formation of **12** is consistent with its strong electrophilic character. Heating this compound at 60 °C in CDCl_3_ for 24 h did not lead to any decomposition. However, the cationic Ir(iii) alkylidene hydride species [(C_5_Me_5_)HIr^*i*^Pr_2_P(Xyl)][BAr^F^_4_] generated by chloride abstraction was reported to react with PMe_3_ to form a similar phosphonium ylide only as a kinetically favored product, which converts to the Ir(iii) alkyl phosphine adduct at 45 °C by migratory insertion of the hydride ligand into the alkylidene functionality.^18*a*^

**Fig. 13:**
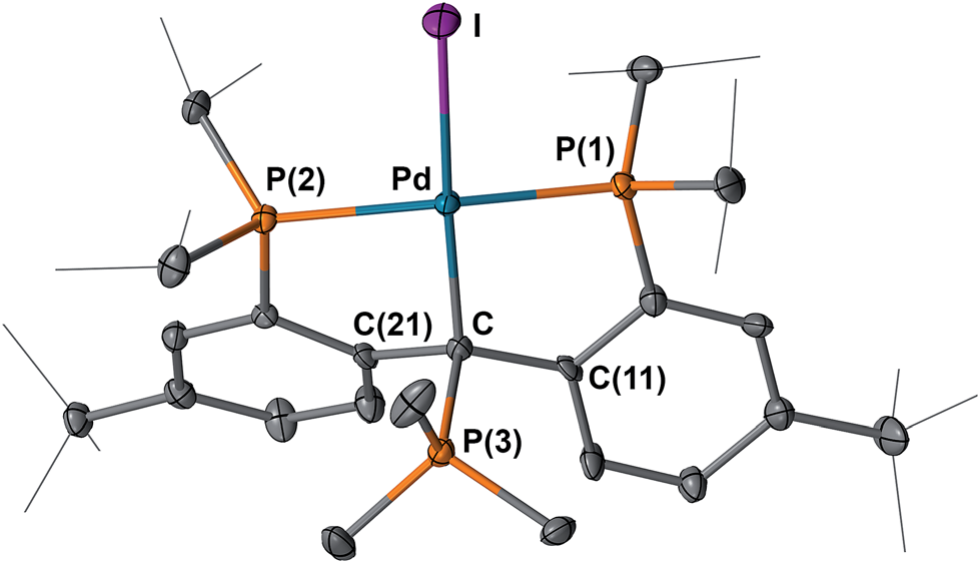
Molecular structure of one of the two crystallographically independent molecules of **12** with displacement parameters at 50% probability level. Hydrogen atoms and the counter anion are omitted for clarity. Selected distances (Å) and angles (degree): Pd–C = 2.119(7), Pd–P(1) = 2.264(2), Pd–P(2) = 2.3279(18), Pd–I = 2.6340(7), C–P(3) = 1.856(7), P(1)–Pd–P(2) = 162.70(7).

The structure of **12** was also determined by X-ray diffraction studies (Fig. 13). Compound **12** contains an sp^3^ hybridized backbone carbon bound to PMe_3_, with an average dihedral angle of 78.9° between the planes defined by P(3), C_backbone_, Pd, I(1) and P(1), P(2), Pd, C_backbone_. The P(3)–C_backbone_ distance of 1.856(7) Å is in the range of 1.85–1.90 Å, typical for a P–C single bond^25^ and close to the corresponding values in the ionic compounds [Me_3_PCPh_3_][B(C_6_F_5_)_4_] (1.887(4) Å)^26^ and [Mes(Me)_2_PCHPh_2_][OTf] (1.854(2) Å).^27^ Other phosphines, such as Ph_3_P did not afford an isolable product probably because of steric crowding. A broad peak at *δ* = 72 ppm was observed in the ^31^P{^1^H} NMR spectrum of the reaction mixture of **5** and Ph_3_P, indicating that some interaction between the carbenium carbon and Ph_3_P occurred upon mixing, but a complicated mixture was formed after heating the reaction at 60 °C.

## Conclusions

In conclusion, the one-electron oxidation of the palladium carbene complex [{PC(sp^2^)P}^*t*Bu^Pd(PMe_3_)] (**3**) with I_2_ generated a monomeric radical carbene complex [{PC˙(sp^2^)P}^*t*Bu^PdI} (**4**), which upon a second one-electron oxidation with [Cp_2_Fe][BAr^F^_4_] formed a cationic carbene complex, [{PC(sp^2^)P}^*t*Bu^PdI][BAr^F^_4_] (**5**). We were able to isolate and characterize for the first time a whole series (Fig. 1) of an anionic carbene (**3**), a carbene radical (**4**), and a cationic carbene (**5**). Our studies show that: (1) transition metal carbene complexes possess a rich redox chemistry that allows the tuning of the electronic properties of the carbene carbon; (2) an umpolung of the M=C_carbene_ bond can be accomplished by successive electron transfer processes; and (3) the electron transfers among the carbene, radical, and cationic species are reversible.

## Experimental

All experiments are performed under an inert atmosphere of N_2_ using standard glovebox techniques. Solvents hexanes, *n*-pentane, diethyl ether, and CH_2_Cl_2_ were dried by passing through a column of activated alumina and stored in the glovebox. THF was dried over LiAlH_4_ followed by vacuum transfer and stored in the glovebox. CDCl_3_ was dried over 4 Å molecular sieves under N_2_, while C_6_D_6_ was dried over CaH_2_ followed by vacuum transfer, and stored in the glovebox. Complex **3**, [Cp_2_Fe][BAr^F^_4_] and KC_8_ were prepared according to literature procedures.^9b,28^ ^*p*^TolNHLi was prepared by deprotonation of *p*-toluidine with ^*n*^BuLi, while PhONa was prepared from NaH and phenol. ^1^H, ^13^C{^1^H}, ^31^P{^1^H}, ^19^F{^1^H} and ^11^B{^1^H} NMR spectra were recorded on a Bruker DRX 500 spectrometer. All chemical shifts are reported in *δ* (ppm) with reference to the residual solvent resonance of deuterated solvents for proton and carbon chemical shifts, and to external H_3_PO_4_, BF_3_·OEt_2_, and CFCl_3_ for ^31^P, ^11^B, and ^19^F chemical shifts, respectively. Magnetic moments were determined by the Evans method^29^ by using a capillary containing 1,3,5-trimethoxybenzene in C_6_D_6_ as a reference. EPR spectrum of compound **4** was recorded on a Bruker EMXplus EPR spectrometer with a standard X-band EMXplus resonator and an EMX premium microwave bridge. Cyclic voltammetry was performed on a Metrohm Autolab PGSTAT-128N instrument. Elemental analyses were performed on a CE-440 Elemental analyzer, or by Midwest Microlab. Gaussian 03 (revision D.02)^30^ was used for all reported calculations. The B3LYP (DFT) method was used to carry out the geometry optimizations on model compounds specified in text using the LANL2DZ basis set. The validity of the true minima was checked by the absence of negative frequencies in the energy Hessian.

### Synthesis of [{PC˙(sp^2^)P}^*t*Bu^PdI] (**4**)

Iodine (11 mg, 0.043 mmol) in 1 mL of THF was slowly added to a dark-brown solution of **3** (60 mg, 0.087 mmol) in 1 mL of THF at –35 °C. Upon addition, a dark-green solution was formed immediately and stirred at ambient temperature for 30 min. All volatiles were removed under reduced pressure and the dark-green residue was extracted with *n*-pentane (2 × 4 mL). After reducing the volume of the pentane solution to about 1 mL, the solution was stored at –35 °C to give **4** as a dark-green crystalline solid; yield 62 mg (97%). Compound **4** is paramagnetic. Magnetic moment (Evans method, 298 K): *μ*_eff_ = 1.85 *μ*_B_; EPR: *g* = 2.0000. Anal. calcd for C_33_H_52_IP_2_Pd (744.04 g mol^–1^): C, 53.27; H, 7.04. Found: C, 53.05; H, 7.24.

### Reduction of **4** with KC_8_

KC_8_ (2.3 mg, 0.016 mmol) was mixed with **4** (12 mg, 0.016 mmol) and PMe_3_ (32 μL, 0.032 mmol, 1 M in THF) in 1 mL THF at room temperature. The mixture turned dark-brown immediately. After stirring the reaction mixture for about 5 min, the volatiles were removed under reduced pressure and the residue was extracted with 1 mL of *n*-pentane. The solution was filtered through celite. Compound **3** was obtained as dark brown crystalline solid from this *n*-pentane solution at –35 °C. ^1^H and ^31^P{^1^H} NMR spectra were identical with the previously reported data.^9*b*^ Yield 9 mg (80%).

### Synthesis of [{PC(sp^2^)P}^*t*Bu^PdI][BAr^F^_4_] (**5**)

[Cp_2_Fe][BAr^F^_4_] (83.2 mg, 0.079 mmol) in 3 mL of diethyl ether was slowly added to a dark-green solution of **4** (59 mg, 0.079 mmol) in 2 mL of diethyl ether at –35 °C. Upon addition, a dark-red solution was formed immediately that was stirred at room temperature for 15 min. After reducing the volume of the ether solution to about 1 mL, *n*-pentane (about 8 mL) was layered and the mixture was stored at –35 °C overnight to afford compound **5** as a dark-red crystalline solid, which was washed with *n*-pentane and dried under vacuum; yield 119 mg (94%). ^1^H NMR (500 MHz, CDCl_3_, 25 °C): *δ* = 7.93 (td, ^4^ *J*_HH_ = 3.8 Hz, ^3^ *J*_HP_ = 1.5 Hz, 2H, Ar*H*), 7.73 (m, 4H, Ar*H*), 7.64 (s, 8H, *ortho*-Ar^F^ *H*), 7.46 (s, 4H, *para*-Ar^F^ *H*), 2.99 (m, 4H, C*H*(CH_3_)_2_), 1.39 (s, 18H, C(C*H*_3_)_3_), 1.36 (dt, ^3^ *J*_HH_ = 9.0 Hz, ^3^ *J*_HP_ = 9.5 Hz, 12H, CH(C*H*_3_)_2_), 1.26 (dt, ^3^ *J*_HH_ = 9.0 Hz, ^3^ *J*_HP_ = 8.0 Hz, 12H, CH(C*H*_3_)_2_) ppm; ^13^C{^1^H} NMR (126 MHz, CDCl_3_, 25 °C): *δ* = 284.47 (s, *C*_carbene_), 169.05 (s, Ar*C*), 161.81 (q, ^1^ *J*_CB_ = 49.94 Hz, *ipso*-Ar^F^ *C*), 156.24 (t, *J*_CP_ = 15.0 Hz, Ar*C*), 148.03 (t, *J*_CP_ = 14.6 Hz, Ar*C*), 136.30 (t, *J*_CP_ = 7.0 Hz, Ar*C*), 134.89 (s, *ortho*-Ar^F^ *C*), 132.11 (s, Ar*C*), 131.50 (s, Ar*C*), 129.00 (q, ^2^ *J*_FC_ = 30.3 Hz, *meta*-Ar^F^ *C*), 124.58 (q, ^2^ *J*_FC_ = 273.1 Hz, *C*F_3_), 117.55 (s, *para*-Ar_F_ *C*), 37.26 (s, *C*(CH_3_)_3_), 30.34 (s, C(*C*H_3_)_3_), 26.66 (d, ^1^ *J*_CP_ = 11.6 Hz, *C*H(CH_3_)_2_), 26.57 (d, ^1^ *J*_CP_ = 11.5 Hz, *C*H(CH_3_)_2_), 19.34 (s, CH(*C*H_3_)_2_), 18.62 (s, CH(*C*H_3_)_2_) ppm; ^31^P {^1^H} NMR (202 MHz, CDCl_3_, 25 °C): *δ* = 72.04 (s) ppm; ^11^B{^1^H} NMR (160 MHz, CDCl_3_, 25 °C): *δ* = –6.62 (s) ppm, ^19^F{^1^H} NMR (470 MHz, CDCl_3_, 25 °C): *δ* = –65.79 (s) ppm. Anal. calcd for C_65_H_64_BF_24_IP_2_Pd (1607.25 g mol^–1^): C, 48.57; H, 4.01. Found: C, 48.37; H, 3.91.

### Reduction of **5** with KC_8_

KC_8_ (2.6 mg, 0.018 mmol) and **5** (30 mg, 0.018 mmol) were mixed in 2 mL of THF at room temperature, which formed a dark-green solution immediately. After stirring the reaction mixture for about 5 min, all volatiles were removed under reduced pressure. The residue was extracted with 4 mL of *n*-pentane, filtered, and the pentane was removed under reduced pressure. The resulted green solid was mixed with 0.5 equiv. of 9,10-dihydroanthracene (2 mg, 0.010 mmol) in C_6_D_6_ and heated at 80 °C for 7 d to afford compound **6** (*vide infra*). Yield 12 mg (92%).

### Synthesis of [{PC(sp^3^)HP}^*t*Bu^PdI] (**6**)

9,10-dihydroanthracene (3.6 mg, 0.020 mmol) and **4** (30 mg, 0.040 mmol) were mixed in 0.6 mL of C_6_D_6_ and heated at 80 °C for 7 d, during which time the dark-green solution slowly turned to bright yellow. All volatiles were removed under reduced pressure and the residue was extracted with 5 mL of *n*-pentane. After reducing the volume of the pentane solution to about 1 mL, the by-product anthracene precipitated as a white crystalline solid, which was filtered, and the solution stored at –35 °C to give orange crystals. Further recrystallization from *n*-pentane at –35 °C afforded **6** as analytically pure orange crystals; yield 24 mg (81%). ^1^H NMR (500 MHz, C_6_D_6_, 25 °C): *δ* = 7.46 (td, ^4^ *J*_HH_ = 4.3 Hz, ^3^ *J*_HP_ = 2.0 Hz, 2H, Ar*H*), 7.42 (dm, ^3^ *J*_HH_ = 8.0 Hz, 2H, Ar*H*), 7.22 (dm, ^3^ *J*_HH_ = 8.0 Hz, 2H, Ar*H*), 6.41 (s, 1H, C*H*_backbone_), 2.78 (m, 2H, C*H*(CH_3_)_2_), 2.60 (m, 2H, C*H*(CH_3_)_2_), 1.44 (dt, ^3^ *J*_HH_ = 7.0 Hz, ^3^ *J*_HP_ = 7.8 Hz, 6H, CH(C*H*_3_)_2_), 1.41 (dt, ^3^ *J*_HH_ = 7.5 Hz, ^3^ *J*_HP_ = 7.8 Hz, 6H, CH(C*H*_3_)_2_), 1.23 (s, 18H, C(C*H*_3_)_3_), 1.16 (dt, ^3^ *J*_HH_ = 7.5 Hz, ^3^ *J*_HP_ = 7.5 Hz, 6H, CH(C*H*_3_)_2_), 1.12 (dt, ^3^ *J*_HH_ = 7.5 Hz, ^3^ *J*_HP_ = 7.3 Hz, 6H, CH(C*H*_3_)_2_) ppm; ^13^C{^1^H} NMR (126 MHz, C_6_D_6_, 25 °C): *δ* = 155.62 (t, *J*_CP_ = 14.7 Hz, Ar*C*), 148.28 (t, *J*_CP_ = 3.0 Hz, Ar*C*), 134.78 (t, *J*_CP_ = 16.6 Hz, Ar*C*), 128.71 (s, Ar*C*), 127.68 (s, Ar*C*), 127.04 (t, *J*_CP_ = 9.6 Hz, Ar*C*), 58.35 (t, ^2^ *J*_CP_ = 4.4 Hz, *C*H_backbone_), 34.44 (s, *C*(CH_3_)_3_), 31.48 (s, C(*C*H_3_)_3_), 26.91 (d, ^1^ *J*_CP_ = 9.1 Hz, *C*H(CH_3_)_2_), 26.82 (d, ^1^ *J*_CP_ = 7.2 Hz, *C*H(CH_3_)_2_), 26.73 (d, ^1^ *J*_CP_ = 5.2 Hz, *C*H(CH_3_)_2_), 19.95 (t, ^2^ *J*_CP_ = 2.6 Hz, CH(*C*H_3_)_2_), 19.42 (t, ^2^ *J*_CP_ = 1.8 Hz, CH(*C*H_3_)_2_), 18.67 (s, CH(*C*H_3_)_2_), 18.50 (s, CH(*C*H_3_)_2_) ppm; ^31^P {^1^H} NMR (202 MHz, C_6_D_6_, 25 °C): *δ* = 51.25 (s) ppm. Anal. calcd for C_33_H_53_IP_2_Pd (745.05 g mol^–1^): C, 53.20; H, 7.17. Found: C, 53.04; H, 7.23.

### Synthesis of [{PC(sp^3^)(SPh)P}^*t*Bu^PdI] (**7**)

PhSSPh (4.4 mg, 0.020 mmol) and **4** (30 mg, 0.040 mmol) were mixed in 0.6 mL of C_6_D_6_ and heated at 80 °C for 7 d, during which time the dark-green solution slowly turned to bright yellow. The volatiles were removed under reduced pressure and the residue was extracted with 5 mL of *n*-pentane and filtered. Removal of the volatiles under reduced pressure gave an yellow waxy solid; yield 32 mg (94%). ^1^H NMR (500 MHz, C_6_D_6_, 25 °C): *δ* = 7.52 (m, 2H, Ar*H*), 7.27 (d, ^3^ *J*_HH_ = 8.5 Hz, 2H, Ar*H*), 7.07 (dm, ^3^ *J*_HH_ = 8.5 Hz, 2H, Ar*H*), 7.01 (d, ^3^ *J*_HH_ = 8.0 Hz, 2H, Ar*H*), 6.97 (tt, ^3^ *J*_HH_ = 7.3 Hz, ^4^ *J*_HH_ = 1.3 Hz, 1H, Ar*H*), 6.91 (tt, ^3^ *J*_HH_ = 7.0 Hz, ^4^ *J*_HH_ = 1.2 Hz, 2H, Ar*H*), 3.11 (m, 2H, C*H*(CH_3_)_2_), 2.76 (m, 2H, C*H*(CH_3_)_2_), 1.51 (dt, ^3^ *J*_HH_ = 7.5 Hz, ^3^ *J*_HP_ = 7.5 Hz, 6H, CH(C*H*_3_)_2_), 1.44 (m, 12H, CH(C*H*_3_)_2_), 1.21 (s, 18H, C(C*H*_3_)_3_), 1.06 (dt, ^3^ *J*_HH_ = 7.5 Hz, ^3^ *J*_HP_ = 7.0 Hz, 6H, CH(C*H*_3_)_2_) ppm; ^13^C{^1^H} NMR (126 MHz, C_6_D_6_, 25 °C): *δ* = 156.63 (t, *J*_CP_ = 12.7 Hz, Ar*C*), 149.44 (t, *J*_CP_ = 2.9 Hz, Ar*C*), 136.58 (t, *J*_CP_ = 3.2 Hz, Ar*C*), 136.34 (s, Ar*C*), 136.23 (t, *J*_CP_ = 16.1 Hz, Ar*C*), 129.60 (s, Ar*C*), 128.84 (t, *J*_CP_ = 9.0 Hz, Ar*C*), 128.57 (s, Ar*C*), 128.52 (s, Ar*C*), 126.41 (s, Ar*C*), 79.58 (t, ^2^ *J*_CP_ = 6.1 Hz, *C*H_backbone_), 34.46 (s, *C*(CH_3_)_3_), 31.41 (s, C(*C*H_3_)_3_), 28.19 (t, ^1^ *J*_CP_ = 11.7 Hz, *C*H(CH_3_)_2_), 27.36 (d, ^1^ *J*_CP_ = 11.1 Hz, *C*H(CH_3_)_2_), 20.00 (s, CH(*C*H_3_)_2_), 19.90 (s, CH(*C*H_3_)_2_), 19.20 (s, CH(*C*H_3_)_2_) ppm; ^31^P{^1^H} NMR (202 MHz, C_6_D_6_, 25 °C): *δ* = 48.59 (s) ppm. Anal. calcd for C_39_H_57_IP_2_PdS (853.21 g mol^–1^): C, 54.90; H, 6.73. Found: C, 54.82; H, 6.65.

### Synthesis of [{PC(sp^3^) (SPh)P}^*t*Bu^PdOTf] (**8**)

AgOTf (9.6 mg, 0.038 mmol) in 1 mL of THF was added to **7** (32 mg, 0.038 mmol) in 1 mL of THF at room temperature. The resulting orange slurry was stirred for 4 h, slowly turning to a pale yellow slurry. The volatiles were removed under reduced pressure and the residue was extracted with 5 mL of benzene and filtered. Removal of volatiles under reduced pressure led to a yellow solid, which was recrystallized from diethyl ether at –35 °C to give **8** as yellow blocks; yield 20 mg (61%). ^1^H NMR (500 MHz, C_6_D_6_, 25 °C): *δ* = 7.89 (dd, ^3^ *J*_HH_ = 8.0 Hz, ^4^ *J*_HH_ = 1.3 Hz, 2H, Ar*H*), 7.55 (d, ^3^ *J*_HH_ = 8.5 Hz, 2H, Ar*H*), 7.37 (m, 2H, Ar*H*), 7.11 (m, 4H, Ar*H*), 6.94 (tt, ^3^ *J*_HH_ = 7.5 Hz, ^4^ *J*_HH_ = 1.3 Hz, 1H, Ar*H*), 3.09 (m, 2H, C*H*(CH_3_)_2_), 2.35 (m, 2H, C*H*(CH_3_)_2_), 1.37 (dt, ^3^ *J*_HH_ = 7.5 Hz, ^3^ *J*_HP_ = 7.8 Hz, 6H, CH(C*H*_3_)_2_), 1.31 (dt, ^3^ *J*_HH_ = 7.5 Hz, ^3^ *J*_HP_ = 7.5 Hz, 6H, CH(C*H*_3_)_2_), 1.16 (s, C(C*H*_3_)_3_), 1.12 (dt, ^3^ *J*_HH_ = 7.5 Hz, ^3^ *J*_HP_ = 7.5 Hz, 6H, CH(C*H*_3_)_2_), 0.93 (dt, ^3^ *J*_HH_ = 7.0 Hz, ^3^ *J*_HP_ = 8.0 Hz, 6H, CH(C*H*_3_)_2_) ppm; ^13^C{^1^H} NMR (126 MHz, C_6_D_6_, 25 °C): *δ* = 154.67 (t, *J*_CP_ = 13.5 Hz, Ar*C*), 150.66 (t, *J*_CP_ = 2.5 Hz, Ar*C*), 137.07 (s, Ar*C*), 134.85 (s, Ar*C*), 134.08 (t, *J*_CP_ = 16.9 Hz, Ar*C*), 129.64 (s, Ar*C*), 129.55 (s, Ar*C*), 128.97 (s, Ar*C*), 128.82 (t, *J*_CP_ = 8.6 Hz, Ar*C*), 127.03 (s, Ar*C*), 121.24 (q, ^1^ *J*_FC_ = 320.3 Hz, *C*F_3_), 76.10 (t, ^2^ *J*_CP_ = 6.6 Hz, *C*_backbone_), 34.54 (s, *C*(CH_3_)_3_), 31.22 (s, C(*C*H_3_)_3_), 26.52 (t, ^1^ *J*_CP_ = 9.6 Hz, *C*H(CH_3_)_2_), 25.88 (t, ^1^ *J*_CP_ = 10.2 Hz, *C*H(CH_3_)_2_), 19.71 (s, CH(*C*H_3_)_2_), 19.19 (s, CH(*C*H_3_)_2_), 18.70 (t, ^2^ *J*_CP_ = 3.0 Hz, CH(*C*H_3_)_2_), 18.29 (s, CH(*C*H_3_)_2_) ppm; ^31^P {^1^H} NMR (202 MHz, C_6_D_6_, 25 °C): *δ* = 49.44 (s) ppm; ^19^F{^1^H} NMR (470 MHz, C_6_D_6_, 25 °C): *δ* = –80.11 (s) ppm. Calcd for C_40_H_57_F_3_O_3_P_2_PdS_2_ (875.37 g mol^–1^): C, 54.88; H, 6.56. Found: C, 54.97; H, 6.67.

### Synthesis of [{PC(sp^3^)(SPh)P}^*t*Bu^PdSPh] (**9**)

PhSSPh (9.4 mg, 0.043 mmol) in 1 mL of benzene was added slowly to a dark-brown solution of **3** (30 mg, 0.043 mmol) in 1 mL of benzene at room temperature. The resulting bright-yellow solution was then stirred for 30 min. The volatiles were removed under reduced pressure and the residue was extracted with *n*-pentane (about 5 mL). After reducing the volume of the solution to about 0.5 mL, storing the concentrated solution at –35 °C afforded compound **9** as yellow blocks; yield 36 mg (100%). ^1^H NMR (500 MHz, C_6_D_6_, 25 °C): *δ* = 8.22 (dd, ^3^ *J*_HH_ = 8.5 Hz, ^4^ *J*_HH_ = 1.0 Hz, 2H, Ar*H*), 7.52 (m, 2H, Ar*H*), 7.19–7.13 (m, 5H, Ar*H*), 7.04–6.93 (m, 5H, Ar*H*), 6.80 (dd, ^3^ *J*_HH_ = 8.0 Hz, ^4^ *J*_HH_ = 1.3 Hz, 2H, Ar*H*), 2.76 (m, 4H, C*H*(CH_3_)_2_), 1.41 (dt, ^3^ *J*_HH_ = 8.0 Hz, ^3^ *J*_HP_ = 8.0 Hz, 6H, CH(C*H*_3_)_2_), 1.40 (dt, ^3^ *J*_HH_ = 8.0 Hz, ^3^ *J*_HP_ = 8.0 Hz, 6H, CH(C*H*_3_)_2_), 1.35 (dt, ^3^ *J*_HH_ = 8.0 Hz, ^3^ *J*_HP_ = 8.0 Hz, 6H, CH(C*H*_3_)_2_), 1.22 (s, 18H, C(C*H*_3_)_3_), 1.10 (dt, ^3^ *J*_HH_ = 7.0 Hz, ^3^ *J*_HP_ = 7.0 Hz, 6H, CH(C*H*_3_)_2_) ppm; ^13^C{^1^H} NMR (126 MHz, C_6_D_6_, 25 °C): *δ* = 157.33 (t, *J*_CP_ = 12.1 Hz, Ar*C*), 150.83 (s, Ar*C*), 148.88 (t, *J*_CP_ = 3.0 Hz, Ar*C*), 137.96 (t, *J*_CP_ = 2.5 Hz, Ar*C*), 136.07 (t, *J*_CP_ = 16.1 Hz, Ar*C*), 135.67 (s, Ar*C*), 133.91 (s, Ar*C*), 129.44 (s, Ar*C*), 129.02 (t, *J*_CP_ = 8.6 Hz, Ar*C*), 128.48 (s, Ar*C*), 127.72 (s, Ar*C*), 126.13 (s, Ar*C*), 121.31 (s, Ar*C*), 74.78 (t, ^2^ *J*_CP_ = 3.2 Hz, *C*_backbone_), 34.40 (s, *C*(CH_3_)_3_), 31.45 (s, C(*C*H_3_)_3_), 26.83 (t, ^1^ *J*_CP_ = 10.7 Hz, *C*H(CH_3_)_2_), 26.72 (t, ^1^ *J*_CP_ = 12.0 Hz, *C*H(CH_3_)_2_), 19.50 (s, CH(*C*H_3_)_2_), 19.40 (s, CH(*C*H_3_)_2_), 19.35 (t, ^2^ *J*_CP_ = 1.9 Hz, CH(*C*H_3_)_2_), 18.97 (s, CH(*C*H_3_)_2_) ppm; ^31^P {^1^H} NMR (202 MHz, C_6_D_6_, 25 °C): *δ* = 44.26 (s) ppm. Anal. calcd for C_45_H_62_P_2_PdS_2_ (835.47 g mol^–1^): C, 64.69; H, 7.48. Found: C, 64.38; H, 7.54.

### Reaction of **5** with NaH

NaH (0.6 mg, 0.024 mmol) and **5** (32 mg, 0.020 mmol) were mixed in 1 mL of THF at room temperature. The dark-red mixture was allowed to stir for 24 h, during which time a yellow solution was slowly formed. Removal of volatiles under reduced pressure led to a yellow solid. The ^1^H and ^31^P{^1^H} NMR of the crude yellow solid in C_6_D_6_ showed clean conversion to **6** and NaBAr^F^_4_.

### Synthesis of [{PC(sp^3^)(NH^*p*^Tol)P}^*t*Bu^PdI} (**10**)

^*p*^TolNHLi (3.3 mg, 0.029 mmol) in 1 mL of THF was slowly added to a dark-red solution of **5** (46 mg, 0.029 mmol) in 1 mL of THF at –35 °C. The resulting brownish-green solution was then stirred at room temperature for 15 min. The volatiles were removed under reduced pressure and the residue was extracted with 5 mL of benzene and filtered. Removal of volatiles under reduced pressure gave a greenish-yellow residue, which was extracted with *n*-pentane (8 mL), filtered, and the volume of the pentane solution reduced to about 1 mL. Storing this solution at –35 °C gave compound **10** as yellow crystals; yield 12 mg (49%). ^1^H NMR (500 MHz, C_6_D_6_, 25 °C): *δ* = 7.52 (m, 2H, Ar*H*), 7.14 (d, ^3^ *J*_HH_ = 8.5 Hz, 2H, Ar*H*), 7.10 (d, ^3^ *J*_HH_ = 8.0 Hz, 2H, Ar*H*), 7.03 (d, ^3^ *J*_HH_ = 8.0 Hz, 2H, Ar*H*), 6.79 (d, ^3^ *J*_HH_ = 8.0 Hz, 2H, Ar*H*), 4.22 (t, *J*_HP_ = 2.8 Hz, 1H, ArN*H*), 2.98 (m, 2H, C*H*(CH_3_)_2_), 2.55 (m, 2H, C*H*(CH_3_)_2_), 1.95 (s, 3H, ArC*H*_3_), 1.53 (dt, ^3^ *J*_HH_ = 8.0 Hz, ^3^ *J*_HP_ = 7.8 Hz, 6H, CH(C*H*_3_)_2_), 1.47 (dt, ^3^ *J*_HH_ = 7.5 Hz, ^3^ *J*_HP_ = 7.5 Hz, 6H, CH(C*H*_3_)_2_), 1.34 (dt, ^3^ *J*_HH_ = 8.0 Hz, ^3^ *J*_HP_ = 7.8 Hz, 6H, CH(C*H*_3_)_2_), 1.17 (s, 18H, C(C*H*_3_)_3_), 1.09 (dt, ^3^ *J*_HH_ = 7.5 Hz, ^3^ *J*_HP_ = 7.3 Hz, 6H, CH(C*H*_3_)_2_) ppm; ^13^C{^1^H} NMR (126 MHz, C_6_D_6_, 25 °C): *δ* = 157.90 (t, *J*_CP_ = 14.6 Hz, Ar*C*), 150.08 (s, Ar*C*), 143.11 (s, Ar*C*), 138.33 (m, Ar*C*), 129.64 (s, Ar*C*), 128.73 (s, Ar*C*), 128.35 (s, Ar*C*), 127.16 (s, Ar*C*), 126.85 (br s, Ar*C*), 116.76 (s, Ar*C*), 87.41 (t, ^2^ *J*_CP_ = 9.5 Hz, *C*_backbone_), 34.55 (s, *C*(CH_3_)_3_), 31.37 (s, C(*C*H_3_)_3_), 27.28 (d, ^1^ *J*_CP_ = 10.7 Hz, *C*H(CH_3_)_2_), 27.19 (d, ^1^ *J*_CP_ = 10.7 Hz, *C*H(CH_3_)_2_), 27.11 (d, ^1^ *J*_CP_ = 10.2 Hz, *C*H(CH_3_)_2_), 27.03 (d, ^1^ *J*_CP_ = 10.2 Hz, *C*H(CH_3_)_2_), 20.54 (s, CH(*C*H_3_)_2_), 20.09 (s, CH(*C*H_3_)_2_), 19.90 (s, Ar*C*H_3_), 19.60 (s, CH(*C*H_3_)_2_), 19.08 (s, CH(*C*H_3_)_2_) ppm; ^31^P {^1^H} NMR (202 MHz, C_6_D_6_, 25 °C): *δ* = 49.89 (br s) ppm. Anal. calcd for C_40_H_60_INP_2_Pd (850.18 g mol^–1^): C, 56.51; H, 7.11; N, 1.65. Found: C, 56.42; H, 7.10; N, 1.61.

### Synthesis of [{PC(sp^3^)(OPh)P}^*t*Bu^PdI] (**11**)

PhONa (5.4 mg, 0.046 mmol) in 1 mL of THF was slowly added to a dark-red solution of **5** (71 mg, 0.044 mmol) in 1 mL of THF at –35 °C. The resulting bright yellow solution was then stirred at room temperature for 15 min. All volatiles were removed under reduced pressure and the residue was extracted with benzene (2 mL × 6) and filtered. Removal of volatiles under reduced pressure gave an yellow residue, which was then extracted with *n*-pentane (6 × 5 mL) and filtered. The solution was reduced to about 1 mL and stored at –35 °C. An yellow oil was obtained that was discarded and the solution carefully transferred to another vial. Slow evaporation at room temperature affords compound **11** as yellow crystals; yield 19 mg (53%). ^1^H NMR (500 MHz, C_6_D_6_, 25 °C): *δ* = 7.95 (dd, ^3^ *J*_HH_ = 7.5 Hz, ^4^ *J*_HH_ = 1.0 Hz, 2H, Ar*H*), 7.49 (m, 2H, Ar*H*), 7.27 (d, ^3^ *J*_HH_ = 8.5 Hz, 2H, Ar*H*), 7.10 (d, ^3^ *J*_HH_ = 8.5 Hz, 2H, Ar*H*), 7.02 (dd, ^3^ *J*_HP_ = 8.5 Hz, ^3^ *J*_HH_ = 7.5 Hz, 2H, Ar*H*), 6.66 (tt, ^3^ *J*_HH_ = 7.5 Hz, ^4^ *J*_HH_ = 1.0 Hz, 1H, Ar*H*), 3.03 (m, 2H, C*H*(CH_3_)_2_), 2.54 (m, 2H, C*H*(CH_3_)_2_), 1.50 (dt, ^3^ *J*_HH_ = 7.5 Hz, ^3^ *J*_HP_ = 7.5 Hz, 6H, CH(C*H*_3_)_2_), 1.42 (dt, ^3^ *J*_HH_ = 7.5 Hz, ^3^ *J*_HP_ = 7.5 Hz, 6H, CH(C*H*_3_)_2_), 1.31 (dt, ^3^ *J*_HH_ = 8.0 Hz, ^3^ *J*_HP_ = 7.8 Hz, 6H, CH(C*H*_3_)_2_), 1.13 (s, 18H, C(C*H*_3_)_3_), 1.09 (dt, ^3^ *J*_HH_ = 7.5 Hz, ^3^ *J*_HP_ = 7.5 Hz, 6H, CH(C*H*_3_)_2_) ppm; ^13^C{^1^H} NMR (126 MHz, C_6_D_6_, 25 °C): *δ* = 157.17 (s, Ar*C*), 155.55 (t, *J*_CP_ = 14.7 Hz, Ar*C*), 150.87 (s, Ar*C*), 137.73 (t, *J*_CP_ = 14.6 Hz, Ar*C*), 129.21 (s, Ar*C*), 128.15 (s, Ar*C*), 127.36 (s, Ar*C*), 121.91 (s, Ar*C*), 120.40 (s, Ar*C*), 113.46 (t, *J*_CP_ = 8.1 Hz, Ar*C*), 58.35 (t, ^2^ *J*_CP_ = 4.6 Hz, *C*_backbone_) 34.58 (s, *C*(CH_3_)_3_), 31.30 (C(*C*H_3_)_3_), 27.42 (t, ^1^ *J*_CP_ = 10.6 Hz, *C*H(CH_3_)_2_), 26.68 (t, ^1^ *J*_CP_ = 11.0 Hz, *C*H(CH_3_)_2_), 20.02 (s, CH(*C*H_3_)_2_), 19.73 (s, CH(*C*H_3_)_2_), 19.43 (s, CH(*C*H_3_)_2_), 18.64 (s, CH(*C*H_3_)_2_) ppm; ^31^P {^1^H} NMR (202 MHz, C_6_D_6_, 25 °C): *δ* = 51.09 (br s) ppm. Anal. calcd for C_39_H_57_IOP_2_Pd (837.14 g mol^–1^): C, 55.95; H, 6.86. Found: C, 55.61; H, 6.61.

### Synthesis of [{PC(sp^2^)(PMe_3_)P}^*t*Bu^PdI][BAr^F^_4_] (**12**)

PMe_3_ (3.6 mg, 0.047 mmol) in 1 mL of diethyl ether was added to a dark-red solution of **5** (50 mg, 0.031 mmol) in 2 mL of diethyl ether at room temperature. The resulting bright yellow solution was stirred for 5 min. The volatiles were removed under reduced pressure and the residue was crystallized by layering *n*-pentane to a fluorobenzene solution at room temperature, affording compound **12** as yellow blocks; yield 52 mg (100%). ^1^H NMR (500 MHz, CDCl_3_, 25 °C): *δ* = 7.81 (d, ^3^ *J*_HH_ = 8.5 Hz, 2H, Ar*H*), 7.66–7.63 (m, 13H, Ar*H* and Ar_F_ *H*), 7.51 (s, 3H, Ar*H*), 3.02 (m, 2H, C*H*(CH_3_)_2_), 2.67 (m, 2H, C*H*(CH_3_)_2_), 1.63 (dt, ^3^ *J*_HH_ = 7.5 Hz, ^3^ *J*_HP_ = 7.5 Hz, 12H, C*H*(CH_3_)_2_), 1.36 (s, 18H, C(C*H*_3_)_3_), 1.31 (d, ^2^ *J*_HP_ = 11.5 Hz, 9H, P(C*H*_3_)_3_), 1.20 (dt, ^3^ *J*_HH_ = 8.5 Hz, ^3^ *J*_HP_ = 8.5 Hz, 6H, C*H*(CH_3_)_2_), 0.92 (dt, ^3^ *J*_HH_ = 8.0 Hz, ^3^ *J*_HP_ = 8.0 Hz, 6H, C*H*(CH_3_)_2_) ppm; ^13^C{^1^H} NMR (126 MHz, CDCl_3_, 25 °C): *δ* = 161.83 (q, ^1^ *J*_CB_ = 49.94 Hz, *ipso*-Ar_F_ *C*), 153.43 (m, Ar*C*), 146.01 (td, *J*_CP_ = 4.8 Hz, *J*_CP_ = 12.6 Hz, Ar*C*), 137.97 (td, *J*_CP_ = 6.4 Hz, *J*_CP_ = 15.1 Hz, Ar*C*), 134.91 (s, *ortho*-Ar_F_ *C*), 131.27 (d, *J*_CP_ = 3.4 Hz, Ar*C*), 129.59 (d, *J*_CP_ = 3.7 Hz, Ar*C*), 129.19 (d, *J*_CP_ = 6.3 Hz, Ar*C*), 129.07 (qm, ^2^ *J*_FC_ = 28.9 Hz, *meta*-Ar_F_ *C*), 124.68 (q, ^2^ *J*_FC_ = 273.2 Hz, *C*F_3_), 117.62 (m, *para*-Ar_F_ *C*), 63.65 (dt, ^1^ *J*_CP_ = 21.5 Hz, ^2^ *J*_CP_ = 2.4 Hz, *C*_backbone_) 34.91 (s, *C*(CH_3_)_3_), 31.08 (s, C(*C*H_3_)_3_), 30.77 (t, *J*_CP_ = 13.0 Hz, *C*H(CH_3_)_2_), 28.55 (t, *J*_CP_ = 10.8 Hz, *C*H(CH_3_)_2_), 20.88 (s, CH(*C*H_3_)_2_), 20.74 (s, CH(*C*H_3_)_2_), 20.15 (s, CH(*C*H_3_)_2_), 19.00 (s, CH(*C*H_3_)_2_), 13.86 (d, ^1^ *J*_CP_ = 54.3 Hz, P(*C*H_3_)_3_) ppm; ^31^P {^1^H} NMR (202 MHz, CDCl_3_, 25 °C): *δ* = 42.17 (s, *P*CH(CH_3_)_2_), 31.01 (s, *P*(CH_3_)_3_) ppm; ^11^B{^1^H} NMR (160 MHz, CDCl_3_, 25 °C): *δ* = –6.64 (s) ppm, ^19^F{^1^H} NMR (470 MHz, CDCl_3_, 25 °C): *δ* = –65.48 (s) ppm. Anal. calcd for C_68_H_73_BF_24_IP_3_Pd (1683.33 g mol^–1^): C, 48.52; H, 4.37. Found: C, 48.84; H, 4.56.

### Comproportionation reaction between **3** and **5**

A solution of [{PC(sp^2^)P}^*t*Bu^PPMe_3_ (**3**, 16 mg, 0.022 mmol) in 2 mL of diethyl ether was added to a solution of [{PC(sp^2^)P}^*t*Bu^PdI][BAr^F^_4_] (**5**, 36 mg, 0.022 mmol) in 1 mL of diethyl ether at –35 °C, which immediately generated a dark green solution. After stirring the mixture at room temperature for 15 min, all volatiles were removed under reduced pressure to give a green oil, which was then mixed with 9,10-dihydroanthracene (4 mg, 0.022 mmol) in C_6_D_6_ (with 10% of THF-d_8_) and heated at 80 °C for 7 days. The ^1^H and ^31^P NMR spectra showed the clean transformation to compound **6** as well as the cationic complex [{PC(sp^3^)HP}^*t*Bu^Pd(PMe_3_)][BAr^F^_4_] in about 1 : 1 ratio.
