# Supplementary material for: Redox-induced umpolung of transition metal carbenes
Source: Chem Sci. 2015 Sep 25;6(12):7343–54. doi: 10.1039/c5sc02859k (PMC5633845; doi:10.1039/c5sc02859k)
Supplement: Supplementary file 2 [file SC-006-C5SC02859K-s002.pdf]

Electronic Supplementary Material (ESI) for Chemical Science.
This journal is © The Royal Society of Chemistry 2015

Electronic Supporting information for:

Redox-Induced Umpolung of Transition Metal Carbenes

*Peng Cui and Vlad M. Iluc**

Department of Chemistry and Biochemistry, University of Notre Dame, Notre Dame, IN 46556

*Corresponding author: viluc@nd.edu

1 X-ray data for compounds 4-6, 8-12 S4

2 EPR Spectra S6
Figure S1. EPR spectrum of [{PC•(sp^2)P}^{tBu}PdI] (**4**) . . . S6

3 DFT Results S7
Figure S2. Computed molecules . . . S7
3.1 [{PC•(sp^2)P}^{tBu}PdI] (**4**) . . . S7
Table S1. Optimized coordinates for [{PC•(sp^2)P}^{tBu}PdI] (**4**) . . . S7
Figure S3. Overlaid structures for [{PC•(sp^2)P}^{tBu}PdI] (**4**) (red: X-ray, blue: optimized) . . . S9
Table S2. Selected distances (Å) and angles (°) for the optimized geometry and the crystal structure of [{PC•(sp^2)P}^{tBu}PdI] (**4**) . . . S10
Figure S4. Optimized geometry for [{PC•(sp^2)P}^{tBu}PdI] (**4**) . . . S10
3.2 [{PC(sp^2)P}^{tBu}PdI][BAr$^F_4$] (**5**) . . . S11
Table S3. Optimized coordinates for the complex cation of [{PC(sp^2)P}^{tBu}PdI][BAr$^F_4$] (**5**) . . . S11
Figure S5. Overlaid structures for the complex cation of [{PC(sp^2)P}^{tBu}PdI][BAr$^F_4$] (**5**) (red: X-ray, blue: optimized) . . . S13
Table S4. Selected distances (Å) and angles (°) for the optimized geometry and the crystal structure of the complex cation of [{PC(sp^2)P}^{tBu}PdI][BAr$^F_4$] (**5**) . . . S14
Figure S6. Optimized geometry for the complex cation of [{PC(sp^2)P}^{tBu}PdI][BAr$^F_4$] (**5**) . . . S14
3.3 [{PC(sp^2)P}^{Me}PdI]$^+$ (**5'**) . . . S15
Table S5. Optimized coordinates for [{PC(sp^2)P}^{Me}PdI]$^+$ (**5'**) . . . S15
Figure S7. Optimized geometry for [{PC(sp^2)P}^{Me}PdI]$^+$ (**5'**) . . . S16

4 NMR Spectra S17
4.1 NMR Spectra for [{PC(sp^2)P}^{tBu}PdI][BAr$^F_4$] (**5**) . . . S17
Figure S8. ^{1}H NMR spectrum for [{PC(sp^2)P}^{tBu}PdI][BAr$^F_4$] (**5**) . . . S17
Figure S9. ^{31}P{^{1}H} NMR spectrum for [{PC(sp^2)P}^{tBu}PdI][BAr$^F_4$] (**5**) . . . S18
Figure S10. ^{13}C{^{1}H} NMR spectrum for [{PC(sp^2)P}^{tBu}PdI][BAr$^F_4$] (**5**) . . . S19
Figure S11. ^{19}F NMR spectrum for [{PC(sp^2)P}^{tBu}PdI][BAr$^F_4$] (**5**) . . . S20
Figure S12. ^{11}B NMR spectrum for [{PC(sp^2)P}^{tBu}PdI][BAr$^F_4$] (**5**) . . . S21
4.2 NMR Spectra for [{PC(sp^3)HP}^{tBu}PdI] (**6**) . . . S22
Figure S13. ^{1}H NMR spectrum for [{PC(sp^3)HP}^{tBu}PdI] (**6**) . . . S22
Figure S14. ^{31}P{^{1}H} NMR spectrum for [{PC(sp^3)HP}^{tBu}PdI] (**6**) . . . S23
Figure S15. ^{13}C{^{1}H} NMR spectrum for [{PC(sp^3)HP}^{tBu}PdI] (**6**) . . . S24
4.3 NMR Spectra for [{PC(sp^3)(SPh)P}^{tBu}PdI] (**7**) . . . S25
Figure S16. ^{1}H NMR spectrum for [{PC(sp^3)(SPh)P}^{tBu}PdI] (**7**) . . . S25
Figure S17. ^{31}P{^{1}H} NMR spectrum for [{PC(sp^3)(SPh)P}^{tBu}PdI] (**7**) . . . S26
Figure S18. ^{13}C{^{1}H} NMR spectrum for [{PC(sp^3)(SPh)P}^{tBu}PdI] (**7**) . . . S27
4.4 NMR Spectra for [{PC(sp^3)(SPh)P}^{tBu}PdOTf] (**8**) . . . S28
Figure S19. ^{1}H NMR spectrum for [{PC(sp^3)(SPh)P}^{tBu}PdOTf] (**8**) . . . S28
Figure S20. ^{31}P{^{1}H} NMR spectrum for [{PC(sp^3)(SPh)P}^{tBu}PdOTf] (**8**) . . . S29
Figure S21. ^{13}C{^{1}H} NMR spectrum for [{PC(sp^3)(SPh)P}^{tBu}PdOTf] (**8**) . . . S30

Figure S22. ^{19}F NMR spectrum for [{PC(sp^3)(SPh)P}^{tBu}PdOTf] (**8**) . . . S31
4.5 NMR Spectra for [{PC(sp^3)(SPh)P}^{tBu}PdSPh] (**9**) . . . S32
Figure S23. ^{1}H NMR spectrum for [{PC(sp^3)(SPh)P}^{tBu}PdSPh] (**9**) . . . S32
Figure S24. ^{31}P{^{1}H} NMR spectrum for [{PC(sp^3)(SPh)P}^{tBu}PdSPh] (**9**) . . . S33
Figure S25. ^{13}C{^{1}H} NMR spectrum for [{PC(sp^3)(SPh)P}^{tBu}PdSPh] (**9**) . . . S34
4.6 NMR Spectra for [{PC(sp^3)(NH^{p}Tol)P}^{tBu}PdI] (**10**) . . . S35
Figure S26. ^{1}H NMR spectrum for [{PC(sp^3)(NH^{p}Tol)P}^{tBu}PdI] (**10**) . . . S35
Figure S27. ^{31}P{^{1}H} NMR spectrum for [{PC(sp^3)(NH^{p}Tol)P}^{tBu}PdI] (**10**) . . . S36
Figure S28. ^{13}C{^{1}H} NMR spectrum for [{PC(sp^3)(NH^{p}Tol)P}^{tBu}PdI] (**10**) . . . S37
Figure S29. ^{1}H-^{13}C HSQC NMR spectrum for [{PC(sp^3)(NH^{p}Tol)P}^{tBu}PdI] (**10**) . . . S38
Figure S30. Variable temperature ^{31}P{^{1}H} NMR spectra for [{PC(sp^3)(NH^{p}Tol)P}^{tBu}PdI] (**10**) . . . S39
4.7 NMR Spectra for [{PC(sp^3)(OPh)P}^{tBu}PdI] (**11**) . . . S40
Figure S31. ^{1}H NMR spectrum for [{PC(sp^3)(OPh)P}^{tBu}PdI] (**11**) . . . S40
Figure S32. ^{31}P{^{1}H} NMR spectrum for [{PC(sp^3)(OPh)P}^{tBu}PdI] (**11**) . . . S41
Figure S33. ^{13}C{^{1}H} NMR spectrum for [{PC(sp^3)(OPh)P}^{tBu}PdI] (**11**) . . . S42
Figure S34. Variable temperature ^{31}P{^{1}H} NMR spectra for [{PC(sp^3)(OPh)P}^{tBu}PdI] (**11**) . . . S43
4.8 NMR Spectra for [{PC(sp^3)(PMe$_3$)P}^{tBu}PdI][BAr$^F_4$] (**12**) . . . S44
Figure S35. ^{1}H NMR spectrum for [{PC(sp^3)(PMe$_3$)P}^{tBu}PdI][BAr$^F_4$] (**12**) . . . S44
Figure S36. ^{31}P{^{1}H} NMR spectrum for [{PC(sp^3)(PMe$_3$)P}^{tBu}PdI][BAr$^F_4$] (**12**) . . . S45
Figure S37. ^{13}C{^{1}H} NMR spectrum for [{PC(sp^3)(PMe$_3$)P}^{tBu}PdI][BAr$^F_4$] (**12**) . . . S46
Figure S38. ^{19}F NMR spectrum for [{PC(sp^3)(PMe$_3$)P}^{tBu}PdI][BAr$^F_4$] (**12**) . . . S47
Figure S39. ^{11}B NMR spectrum for [{PC(sp^3)(PMe$_3$)P}^{tBu}PdI][BAr$^F_4$] (**12**) . . . S48
Figure S40. ^{1}H-^{13}C HSQC NMR spectrum for [{PC(sp^3)(PMe$_3$)P}^{tBu}PdI][BAr$^F_4$] (**12**) . . . S49
4.9 NMR Spectra for the comproportionation reaction between **3** and **5** . . . S50
Figure S41. comproportionation reaction between **3** and **5** . . . S50
Figure S42. ^{1}H NMR spectrum for the reaction mixture . . . S51
Figure S43. ^{31}P{^{1}H} NMR spectrum for the reaction mixture . . . S52

5 Crystallographic tables **S53**
5.1 Crystal data for [{PC$^\bullet$(sp^2)P}^{tBu}PdI] (**4**) . . . S53
Figure S44. Thermal-ellipsoid representation of **4** . . . S53
Table S6. Crystal data and structure refinement for **4** . . . S54
Table S7. Atomic coordinates and equivalent isotropic displacement parameters (Å^2) for **4** . . . S55
Table S8. Anisotropic displacement parameters (Å^2) for **4** . . . S58
Table S9. Distances [Å] for **4** . . . S59
Table S10. Angles [°] for **4** . . . S61
5.2 Crystal data for [{PC(sp^2)P}^{tBu}PdI][BAr$^F_4$] (**5**) . . . S64
Figure S45. Thermal-ellipsoid representation of **5** . . . S64
Table S11. Crystal data and structure refinement for **5** . . . S65
Table S12. Atomic coordinates and equivalent isotropic displacement parameters (Å^2) for **5** . . . S66
Table S13. Anisotropic displacement parameters (Å^2) for **5** . . . S70
Table S14. Distances [Å] for **5** . . . S73
Table S15. Angles [°] for **5** . . . S75
5.3 Crystal data for [{PC(sp^3)HP}^{tBu}PdI] (**6**) . . . S80
Figure S46. Thermal-ellipsoid representation of **6** . . . S80
Table S16. Crystal data and structure refinement for **6** . . . S81
Table S17. Atomic coordinates and equivalent isotropic displacement parameters (Å^2) for **6** . . . S82
Table S18. Anisotropic displacement parameters (Å^2) for **6** . . . S84
Table S19. Distances [Å] for **6** . . . S85
Table S20. Angles [°] for **6** . . . S86
5.4 Crystal data for [{PC(sp^3)(SPh)P}^{tBu}PdOTf]·Et$_2$O (**8·Et$_2$O**) . . . S88
Figure S47. Thermal-ellipsoid representation of **8·Et$_2$O** . . . S88
Table S21. Crystal data and structure refinement for **8·Et$_2$O** . . . S89
Table S22. Atomic coordinates and equivalent isotropic displacement parameters (Å^2) for **8·Et$_2$O** . . . S90

Table S23. Anisotropic displacement parameters ($Å^2$) for **8·Et$_2$O** . . . S93
Table S24. Distances [Å] for **8·Et$_2$O** . . . S95
Table S25. Angles [°] for **8·Et$_2$O** . . . S97
5.5 Crystal data for [{PC(sp^3)(SPh)P}^{tBu}PdSPh]·C_5H_{12} (**9·C$_5$H$_{12}$**) . . . S100
Figure S48. Thermal-ellipsoid representation of **9·C$_5$H$_{12}$** . . . S100
Table S26. Crystal data and structure refinement for **9·C$_5$H$_{12}$** . . . S101
Table S27. Atomic coordinates and equivalent isotropic displacement parameters ($Å^2$) for **9·C$_5$H$_{12}$** . . . S102
Table S28. Anisotropic displacement parameters ($Å^2$) for **9·C$_5$H$_{12}$** . . . S105
Table S29. Distances [Å] for **9·C$_5$H$_{12}$** . . . S107
Table S30. Angles [°] for **9·C$_5$H$_{12}$** . . . S109
5.6 Crystal data for [{PC(sp^3)(NH^{p}Tol)P}^{tBu}PdI] (**10**) . . . S112
Figure S49. Thermal-ellipsoid representation of **10** . . . S112
Table S31. Crystal data and structure refinement for **10** . . . S113
Table S32. Atomic coordinates and equivalent isotropic displacement parameters ($Å^2$) for **10** . . . S114
Table S33. Anisotropic displacement parameters ($Å^2$) for **10** . . . S117
Table S34. Distances [Å] for **10** . . . S118
Table S35. Angles [°] for **10** . . . S120
5.7 Crystal data for [{PC(sp^3)(OPhP)}^{tBu}PdI] (**11**) . . . S123
Figure S50. Thermal-ellipsoid representation of **11** . . . S123
Table S36. Crystal data and structure refinement for **11** . . . S124
Table S37. Atomic coordinates and equivalent isotropic displacement parameters ($Å^2$) for **11** . . . S125
Table S38. Anisotropic displacement parameters ($Å^2$) for **11** . . . S130
Table S39. Distances [Å] for **11** . . . S132
Table S40. Angles [°] for **11** . . . S135
5.8 Crystal data for [{PC(sp^3)(PMe$_3$)P}^{tBu}PdI][BAr$^F_4$] (**12**) . . . S140
Figure S51. Thermal-ellipsoid representation of **12** . . . S140
Table S41. Crystal data and structure refinement for **12** . . . S141
Table S42. Atomic coordinates and equivalent isotropic displacement parameters ($Å^2$) for **12** . . . S142
Table S43. Anisotropic displacement parameters ($Å^2$) for **12** . . . S149
Table S44. Distances [Å] for **12** . . . S153
Table S45. Angles [°] for **12** . . . S157

1 X-ray data for compounds 4-6, 8-12

X−Ray crystal structure of [{PC•(sp^2)P}^{tBu}PdI] (4). Single crystals were obtained as dark green blocks from a concentrated *n*-pentane solution at −35 °C in the glovebox. Crystal and refinement data for **4**: $C_{33}H_{52}IP_2Pd$; M_r =743.99; Orthorhombic; space group *Pbca*; *a* = 14.978(2) Å; *b* = 16.438(3) Å; *c* = 29.361(5) Å; α = 90°; β = 90°; γ = 90°; V = 7228.9(19) Å^3; Z = 8; T = 120(2) K; λ = 0.71073 Å; μ = 1.474 mm^{-1}; d_{calc} = 1.367 g·cm^{-3}; 141424 reflections collected; 6340 unique (R_{int} = 0.0644); giving R_1 = 0.0290, wR_2 = 0.0590 for 5847 data with [I>2σ(I)] and R_1 = 0.0343, wR_2 = 0.0613 for all 6340 data. Residual electron density (e^-·Å^{-3}) max/min: 0.850/−0.569.

X−Ray crystal structure of [{PC(sp^2)P}^{tBu}PdI][BAr$^F_4$] (5). Single crystals were obtained as dark red blocks from a concentrated solution of diethyl ether layered with *n*-pentane at −35 °C in the glovebox. Crystal and refinement data for **5**: $C_{65}H_{64}BF_{24}IP_2Pd$; M_r =1607.21; Monoclinic; space group *P*2$_1$/*n*; *a* = 14.0602(6) Å; *b* = 28.6171(12) Å; *c* = 16.9031(7) Å; α = 90°; β = 92.0331(16)°; γ = 90°; V = 6796.9(5) Å^3; Z = 4; T = 120(2) K; λ = 0.71073 Å; μ = 0.879 mm^{-1}; d_{calc} = 1.571 g·cm^{-3}; 119559 reflections collected; 11970 unique (R_{int} = 0.0438); giving R_1 = 0.0320, wR_2 = 0.0725 for 10118 data with [I>2σ(I)] and R_1 = 0.0412, wR_2 = 0.0763 for all 11970 data. Residual electron density (e^-·Å^{-3}) max/min: 1.082/−0.735.

X−Ray crystal structure of [{PC(sp^3)HP}^{tBu}PdI] (6). Single crystals were obtained as pale yellow blocks by slow evaporation from a concentrated solution of *n*-pentane at room temperature in the glovebox. Crystal and refinement data for **6**: $C_{33}H_{53}IP_2Pd$; M_r =744.99; Monoclinic; space group *P*2$_1$/*c*; *a* = 12.8203(13) Å; *b* = 14.8340(15) Å; *c* = 19.1740(19) Å; α = 90°; β = 106.6410(15)°; γ = 90°; V = 3493.7(6) Å^3; Z = 4; T = 120(2) K; λ = 0.71073 Å; μ = 1.525 mm^{-1}; d_{calc} = 1.416 g·cm^{-3}; 63141 reflections collected; 6140 unique (R_{int} = 0.0317); giving R_1 = 0.0245, wR_2 = 0.0495 for 5688 data with [I>2σ(I)] and R_1 = 0.0278, wR_2 = 0.0505 for all 6140 data. Residual electron density (e^-·Å^{-3}) max/min: 1.086/−1.021.

X−Ray crystal structure of [{PC(sp^3)(SPh)P}^{tBu}PdOTf]·Et$_2$O (8·Et$_2$O) Single crystals were obtained as yellow blocks from a concentrated solution of diethyl ether at −35 °C in the glovebox. Crystal and refinement data for **8·Et$_2$O**: $C_{44}H_{67}F_3O_4P_2PdS_2$; M_r =949.44; Orthorhombic; space group *P*2$_1$2$_1$2$_1$; *a* = 14.0266(6) Å; *b* = 15.2323(6) Å; *c* = 21.3735(9) Å; α = 90°; β = 90°; γ = 90°; V = 4566.6(3) Å^3; Z = 4; T = 120(2) K; λ = 0.71073 Å; μ = 0.620 mm^{-1}; d_{calc} = 1.381 g·cm^{-3}; 77120 reflections collected; 9882 unique (R_{int} = 0.0493); giving R_1 = 0.0260, wR_2 = 0.0561 for 8958 data with [I>2σ(I)] and R_1 = 0.0331, wR_2 = 0.0590 for all 9882 data. Residual electron density (e^-·Å^{-3}) max/min: 0.885/−0.528.

X−Ray crystal structure of [{PC(sp^3)(SPh)P}^{tBu}PdSPh]·C$_5$H$_{12}$ (9·C$_5$H$_{12}$). Single crystals were obtained as yellow blocks from a concentrated solution of *n*-pentane at −35 °C in the glovebox. Crystal and refinement data for **9·C$_5$H$_{12}$**: $C_{50}H_{74}P_2PdS_2$; M_r =907.55; Monoclinic; space group *P*2$_1$/*n*; *a* = 11.297(3) Å; *b* = 11.590(3) Å; *c* = 36.768(10) Å; α = 90°; β = 92.926(4)°; γ = 90°; V = 4808(2) Å^3; Z = 4; T = 120(2) K; λ = 0.71073 Å; μ = 0.571 mm^{-1}; d_{calc} = 1.254 g·cm^{-3}; 111329 reflections collected; 12309 unique (R_{int} = 0.0632); giving R_1 = 0.0392, wR_2 = 0.0754 for 10029 data with [I>2σ(I)] and R_1 = 0.0557, wR_2 = 0.0807 for all 12309 data. Residual electron density (e^-·Å^{-3}) max/min: 1.121/−0.773.

X−Ray crystal structure of [{PC(sp^3)(NH^{p}Tol)P}^{tBu}PdI] (10). Single crystals were obtained as yellow blocks from a concentrated solution of *n*-pentane at −35 °C in the glovebox. Crystal and refinement data for **10**: $C_{40}H_{60}INP_2Pd$; M_r =850.13; Monoclinic; space group *P*2$_1$/*n*; *a* = 17.0891(17) Å; *b* = 14.0140(14) Å; *c* = 18.4206(18) Å; α = 90°; β = 100.081(3)°; γ = 90°; V = 4343.4(7) Å^3; Z = 4; T = 120(2) K; λ = 0.71073 Å; μ = 1.237 mm^{-1}; d_{calc} = 1.300 g·cm^{-3}; 67692 reflections collected; 7652 unique (R_{int} = 0.0271); giving R_1 = 0.0300, wR_2 = 0.0772 for 6972 data with [I>2σ(I)] and R_1 = 0.0334, wR_2 = 0.0792 for all 7652 data. Residual electron density (e^-·Å^{-3}) max/min: 1.637/−1.126.

X−Ray crystal structure of [{PC(sp^3)(OPhP)}^{tBu}PdI] (11). Single crystals were obtained as yellow blocks by slow evaporation from a concentrated *n*-pentane solution at room temperature in the glovebox. Crystal and refinement data for **11**: $C_{39}H_{57}IOP_2Pd$; M_r =837.09; Triclinic; space group $P\bar{1}$; *a* = 11.8764(6) Å; *b* = 14.4330(8) Å; *c* = 25.1741(14) Å; α = 101.2966(16)°; β = 93.5784(16)°; γ = 111.2000(15)°; V = 3903.0(4) Å^3; Z = 4; T = 120(2) K; λ = 0.71073 Å; μ = 1.376 mm^{-1}; d_{calc} = 1.425 g·cm^{-3}; 95643 reflections collected; 13721 unique (R_{int} = 0.0511); giving R_1 = 0.0389, wR_2 = 0.0886 for 11059 data with [I>2σ(I)] and R_1 = 0.0556, wR_2 = 0.0934 for all 13721 data. Residual electron density (e^-·Å^{-3}) max/min: 3.446/−2.064.

X−Ray crystal structure of [{PC(sp^3)(PMe$_3$)P}^{tBu}PdI][BAr$^F_4$] (12). Single crystals were obtained as yellow blocks from a concentrated solution of *n*-pentane layered with fluorobenzene at −35 °C in the glovebox. Crystal

and refinement data for **12**: $C_{68}H_{72}BF_{24}IP_3Pd$; M_r =1682.28; Monoclinic; space group $P2_1$; a = 10.0901(7) Å; b = 23.7955(17) Å; c = 31.205(2) Å; α = 90°; β = 90.532(2)°; γ = 90°; V = 7492.0(9) $Å^3$; Z = 4; T = 120(2) K; λ = 0.71073 Å; μ = 0.822 mm^{-1}; d_{calc} = 1.491 $g{\cdot}cm^{-3}$; 134462 reflections collected; 25436 unique (R_{int} = 0.0348); giving R_1 = 0.0632, wR_2 = 0.1651 for 24104 data with [I>2σ(I)] and R_1 = 0.0658, wR_2 = 0.1675 for all 25436 data. Residual electron density ($e^{-}{\cdot}Å^{-3}$) max/min: 2.325/−1.408.

2 EPR Spectra

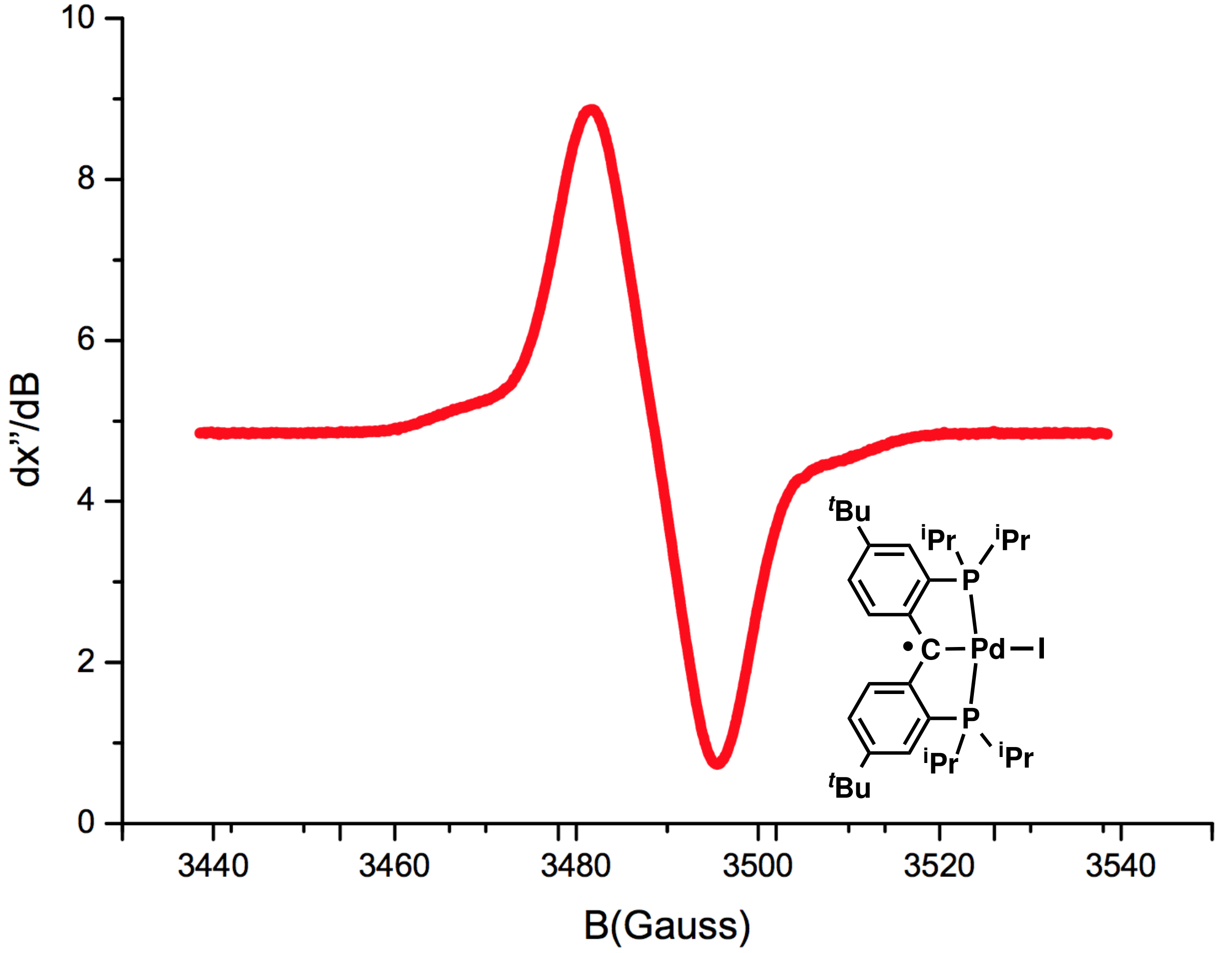

Figure S1. EPR spectrum of [{PC•(sp^2)P}^{tBu}PdI] (**4**) (0.1 mM solution in toluene, 298 K), ΔH_{pp} = 10.0 G; g = 2.000.

3 DFT Results

Gaussian 03 (revision D.02)[1] was used for all reported calculations. The B3LYP (DFT) method was used to carry out the geometry optimizations on model compounds specified in text using the LANL2DZ basis set. The validity of the true minima was checked by the absence of negative frequencies in the energy Hessian. Molecular orbitals as well as Mayer[2] and Wiberg[3] bond orders were obtained from single point computations performed on the optimized molecules using the same level of theory.

1. M. J. Frisch, G. W. Trucks, H. B. Schlegel, G. E. Scuseria, M. A. Robb, J. R. Cheeseman, J. A. Montgomery, T. Vreven, K. N. Kudin, J. C. Burant, J. M. Millam, S. S. Iyengar, J. Tomasi, V. Barone, B. Mennucci, M. Cossi, G. Scalmani, N. Rega, G. A. Petersson, H. Nakatsuji, M. Hada, M. Ehara, K. Toyota, R. Fukuda, J. Hasegawa, M. Ishida, T. Nakajima, Y. Honda, O. Kitao, H. Nakai, M. Klene, X. Li, J. E. Knox, H. P. Hratchian, J. B. Cross, V. Bakken, C. Adamo, J. Jaramillo, R. Gomperts, R. E. Stratmann, O. Yazyev, A. J. Austin, R. Cammi, C. Pomelli, J. W. Ochterski, P. Y. Ayala, K. Morokuma, G. A. Voth, P. Salvador, J. J. Dannenberg, V. G. Zakrzewski, S. Dapprich, A. D. Daniels, M. C. Strain, O. Farkas, D. K. Malick, A. D. Rabuck, K. Raghavachari, J. B. Foresman, J. V. Ortiz, Q. Cui, A. G. Baboul, S. Clifford, J. Cioslowski, B. B. Stefanov, G. Liu, A. Liashenko, P. Piskorz, I. Komaromi, R. L. Martin, D. J. Fox, T. Keith, M. A. Al-Laham, C. Y. Peng, A. Nanayakkara, M. Challacombe, P. M. W. Gill, B. Johnson, W. Chen, M. W. Wong, C. Gonzalez, and J. A. Pople, *Gaussian, Inc.*, Wallingford CT, 2004.
2. I. Mayer, *Chem. Phys. Lett.*, **1983**, *97*, 270-274.
3. K. B. Wiberg, *Tetrahedron*, **1968**, *24*, 1083-1096.

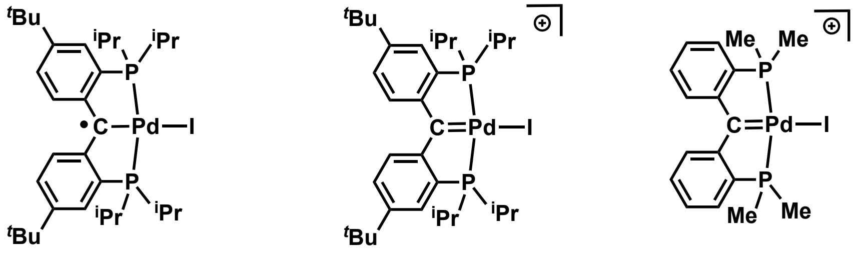

4 5 5'

Figure S2. Computed molecules.

3.1 [{PC•(sp^2)P}^{tBu}PdI] (4)

Table S1. Optimized coordinates for [{PC•(sp^2)P}^{tBu}PdI] (**4**).

atom	x	y	z
C	1.429743	2.912961	−0.666313
C	1.297677	1.578004	−0.162495
C	2.524473	0.893967	0.113058
C	3.770022	1.527973	−0.024852
C	3.886672	2.860039	−0.481347
C	2.677555	3.522182	−0.818607
C	0.017692	0.888742	0.003533
Pd	0.006986	−1.161439	−0.025823
P	2.312060	−0.910798	0.570582
C	2.742706	−1.093763	2.434191
C	1.691938	−0.347617	3.284924

Continued on next page

Table S1. – continued from previous page

atom	x	y	x
C	5.240627	3.583112	−0.651288
C	5.436779	3.973184	−2.147090
C	5.249243	4.874601	0.219717
C	6.440721	2.702604	−0.221453
C	4.177738	−0.647591	2.789701
C	−1.251270	1.595871	0.168582
C	−2.489693	0.935274	−0.116163
C	−3.716955	1.616889	−0.067542
C	−3.808825	2.965522	0.343857
C	−2.595595	3.594451	0.729358
C	−1.362457	2.944956	0.641547
P	−2.311428	−0.886358	−0.505205
C	−3.462908	−1.871774	0.674266
C	−4.978061	−1.674281	0.447680
C	−5.143042	3.739062	0.423056
C	−5.055964	5.015256	−0.466254
C	−5.409455	4.160711	1.899203
C	−6.347087	2.894499	−0.063530
C	−3.004390	−1.116000	−2.278551
C	−3.108728	−2.609414	−2.658333
C	−2.124268	−0.336891	−3.280378
C	−3.063731	−1.588411	2.138963
I	−0.096297	−3.948276	−0.017221
C	3.702698	−1.821718	−0.389449
C	3.915628	−3.261424	0.128116
C	3.376005	−1.804905	−1.899639
H	−4.620609	1.086687	−0.346505
H	−2.614354	4.614101	1.108403
H	−0.470348	3.465696	0.973009
H	−4.605018	4.794320	2.291717
H	−5.489474	3.279079	2.548325
H	−6.348507	4.726391	1.970245
H	−7.265255	3.492465	−0.001516
H	−6.494122	1.999550	0.555350
H	−6.226515	2.577753	−1.107882
H	−4.884128	4.748382	−1.517102
H	−4.239654	5.675598	−0.150103
H	−5.992745	5.585612	−0.404725
H	4.670330	0.968845	0.206672
H	2.711421	4.531643	−1.222926
H	0.542522	3.450524	−0.983250
H	4.436781	5.556528	−0.058463
H	6.198536	5.412495	0.092461
H	5.132656	4.629735	1.283456
H	7.374049	3.266363	−0.346437
H	6.519524	1.794204	−0.833007
H	6.371287	2.405707	0.833395
H	4.633335	4.628157	−2.504931
H	5.450801	3.079748	−2.784646
H	6.388639	4.505493	−2.279389
H	−4.009635	−0.667876	−2.266758

Continued on next page

Table S1. – continued from previous page

**atom**	**x**	**y**	**x**
H	−3.195453	−2.912215	0.445231
H	−2.549171	−0.422610	−4.289808
H	−1.106522	−0.745799	−3.302300
H	−2.059272	0.727937	−3.026379
H	−3.788134	−3.163793	−1.999829
H	−2.128647	−3.097694	−2.611134
H	−3.489164	−2.698762	−3.685274
H	−3.245922	−0.540078	2.409565
H	−2.004693	−1.814103	2.307245
H	−3.657478	−2.222587	2.811174
H	−5.304657	−0.673449	0.753815
H	−5.528607	−2.399795	1.062640
H	−5.276905	−1.835390	−0.595310
H	4.619432	−1.238319	−0.217191
H	2.640545	−2.172154	2.617737
H	2.459962	−2.372819	−2.101066
H	4.198053	−2.270806	−2.460241
H	3.241227	−0.784284	−2.278766
H	4.223351	−3.287177	1.180937
H	4.707868	−3.743425	−0.461396
H	3.001460	−3.855749	0.021685
H	4.304961	0.430832	2.634972
H	4.940224	−1.175654	2.204048
H	4.372374	−0.855298	3.850987
H	1.728489	0.733857	3.099623
H	1.890346	−0.516751	4.352193
H	0.676773	−0.695395	3.062554

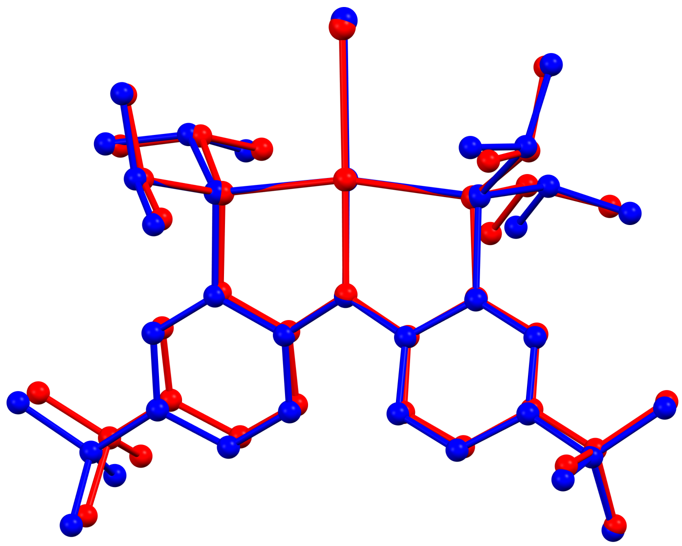

Figure S3. Overlaid structures for [{PC$^{\bullet}$(sp^2)P}$^{t\text{Bu}}$PdI] (**4**) (red: X-ray, blue: optimized).

Table S2. Selected distances (Å) and angles (°) for the optimized geometry and the crystal structure of [{PC•(sp^2)P}^{tBu}PdI] (**4**).

Distance	Calcd.	X-Ray	Angle	Calcd.	X-Ray
Pd – I	2.789	2.675(5)	P(1) – Pd – P(2)	167.05	163.13(3)
Pd – P(1)	2.383	2.2880(7)	I – Pd – C	177.92	175.42(13)
Pd – P(2)	2.394	2.2931(8)	P(1) – Pd – C	83.83	83.32(8)
Pd – C	2.050	2.022(3)	C(21) – C – Pd	118.29	118.8(2)
C – C(11)	1.462	1.441(4)	C(11) – C – Pd	118.73	118.5(2)
C – C(21)	1.463	1.462(4)	C(11) – C – C(21)	122.97	122.5(2)

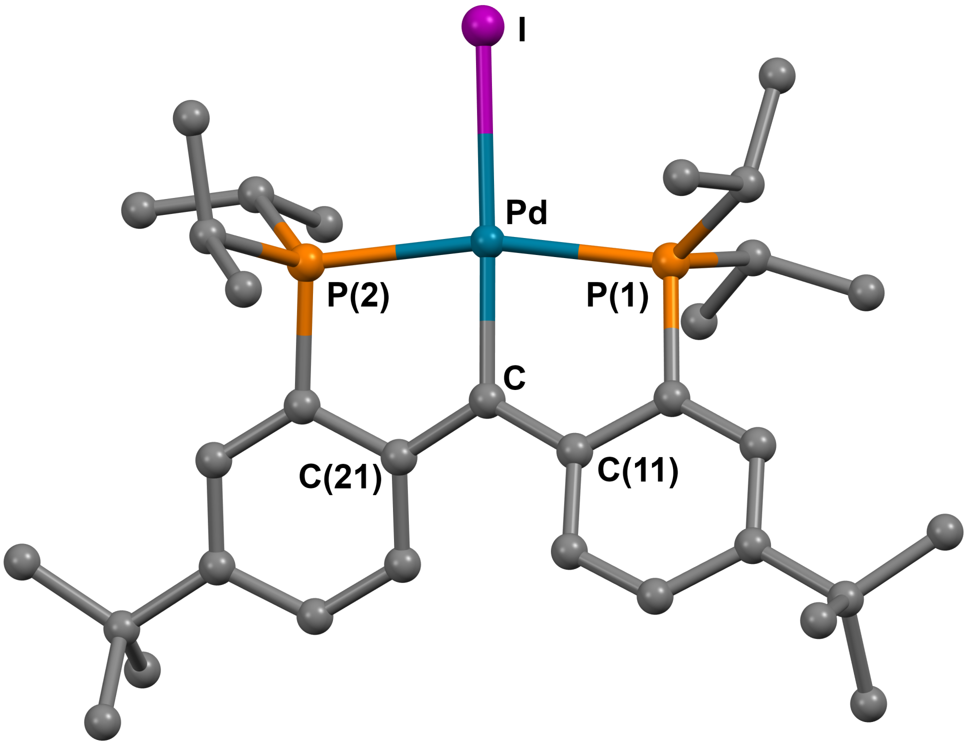

Figure S4. Optimized geometry for [{PC•(sp^2)P}^{tBu}PdI] (**4**).

3.2 [{PC(sp^2)P}^{tBu}PdI][BAr^F_4] (5)

Table S3. Optimized coordinates for the complex cation of [{PC(sp^2)P}^{tBu}PdI][BAr^F_4] (**5**).

atom	x	y	z
C	−3.717499	1.540241	0.018668
C	−2.489342	0.884627	−0.127546
C	−1.262457	1.555793	0.174401
C	−1.353234	2.876650	0.714801
C	−2.588444	3.500665	0.889437
C	−3.803677	2.863896	0.521926
C	0.000010	0.845052	−0.000739
Pd	0.000779	−1.163792	−0.001310
P	−2.306889	−0.920385	−0.638243
C	−3.731784	−1.821408	0.284174
C	−3.992609	−3.230433	−0.292756
C	−5.143704	3.601365	0.706946
C	−5.332090	3.938213	2.218806
C	−2.671483	−1.038965	−2.521108
C	−4.082473	−0.540407	−2.903302
C	−5.112122	4.924828	−0.117451
C	−6.357217	2.761533	0.237887
C	−1.569718	−0.312812	−3.321726
C	−3.427325	−1.866569	1.798033
C	1.262464	1.555977	−0.176018
C	2.489292	0.885490	0.127612
C	3.717356	1.541390	−0.018218
C	3.803537	2.864305	−0.523411
C	2.588363	3.500316	−0.892462
C	1.353185	2.876218	−0.717847
P	2.306520	−0.918939	0.640049
C	3.734547	−1.820788	−0.276731
C	3.994956	−3.228494	0.303481
C	5.143508	3.601917	−0.708456
C	6.356516	2.764999	−0.232928
C	2.664445	−1.035087	2.524333
C	4.073926	−0.535208	2.910685
C	5.334952	3.932265	−2.221464
C	5.109270	4.928908	0.109925
C	1.559897	−0.308462	3.320598
C	3.434132	−1.868989	−1.791334
I	0.001790	−3.883536	−0.002652
H	4.631846	1.021272	0.240567
H	2.612689	4.493176	−1.331281
H	0.453906	3.381666	−1.053191
H	6.279494	4.471483	−2.363081
H	4.529296	4.565434	−2.611273
H	5.371701	3.017009	−2.825707
H	6.051151	5.472679	−0.031679
H	4.989338	4.730970	1.182692
H	4.294116	5.589680	−0.207396
H	6.290100	2.511789	0.833432
H	7.275056	3.346253	−0.372951

Continued on next page

Table S3. – continued from previous page

atom	x	y	x
H	6.468606	1.836979	−0.808715
H	−0.453905	3.382766	1.048964
H	−2.612717	4.493980	1.327242
H	−4.632050	1.019436	−0.238447
H	2.591058	−2.112467	2.725066
H	−4.993608	4.722235	−1.189497
H	−4.297188	5.587844	0.195709
H	−6.054396	5.468155	0.023208
H	−6.293197	2.503836	−0.827532
H	−7.275862	3.342686	0.377702
H	−6.467099	1.835943	0.817979
H	−6.275969	4.478747	2.359884
H	−4.525283	4.572320	2.604676
H	−5.368471	3.025481	2.826904
H	4.623639	−1.196077	−0.107164
H	1.579250	0.774071	3.136766
H	1.720374	−0.466634	4.394534
H	0.562652	−0.687148	3.068052
H	4.176539	0.543330	2.739094
H	4.872962	−1.054718	2.368648
H	4.236188	−0.713407	3.981536
H	4.287705	−2.316618	−2.316109
H	3.266479	−0.870803	−2.215404
H	2.552063	−2.487509	−1.992684
H	4.269304	−3.204017	1.364683
H	4.830562	−3.686223	−0.241685
H	3.120386	−3.876252	0.185977
H	−2.598305	−2.116541	−2.720850
H	−4.621870	−1.197796	0.115619
H	−0.571659	−0.691399	−3.072240
H	−1.588376	0.769824	−3.138490
H	−1.733841	−0.471662	−4.395010
H	−4.184788	0.538521	−2.734115
H	−4.879200	−1.058802	−2.356826
H	−4.248766	−0.721417	−3.973054
H	−3.259867	−0.867441	2.219965
H	−2.543886	−2.483551	1.998191
H	−4.278895	−2.314400	2.325861
H	−3.116953	−3.877059	−0.177125
H	−4.270669	−3.208017	−1.353049
H	−4.825853	−3.688093	0.256063

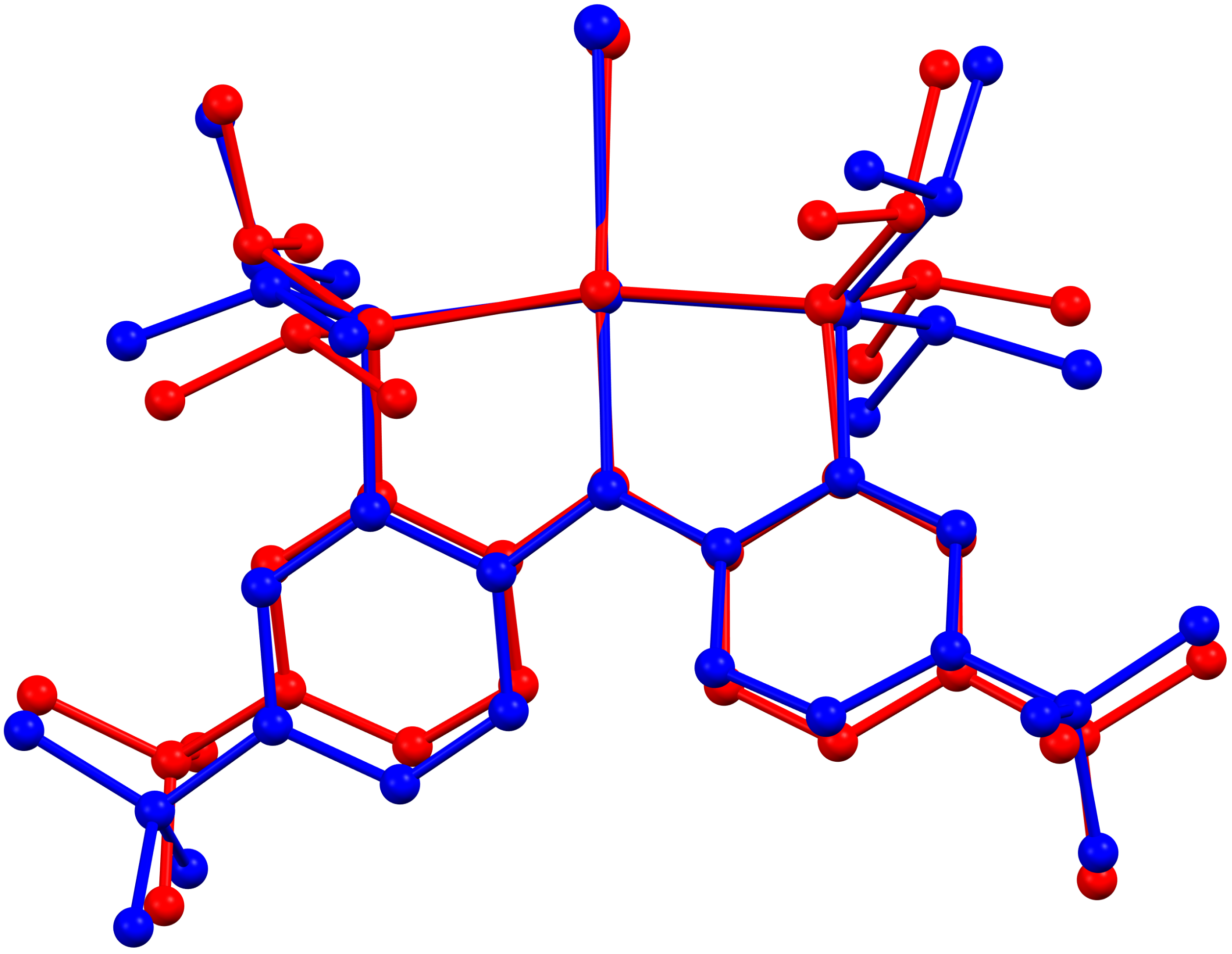

Figure S5. Overlaid structures for the complex cation of [{PC(sp^2)P}^{tBu}PdI][BAr$^F_4$] (**5**) (red: X-ray, blue: optimized).

Table S4. Selected distances (Å) and angles (°) for the optimized geometry and the crystal structure of the complex cation of [{PC(sp^2)P}^{tBu}PdI][BAr$^F_4$] (**5**).

**Distance**	**Calcd.**	**X-Ray**	**Angle**	**Calcd.**	**X-Ray**
Pd – I	2.720	2.6255(3)	P(1) – Pd – P(2)	168.35	164.98(3)
Pd – P(1)	2.406	2.3161(7)	I – Pd – C	179.99	168.44(7)
Pd – P(2)	2.406	2.2884(7)	P(1) – Pd – C	84.18	83.03(8)
Pd – C	2.009	1.968(3)	C(21) – C – Pd	119.13	121.2(2)
C – C(11)	1.459	1.442(4)	C(11) – C – Pd	119.17	117.59(19)
C – C(21)	1.459	1.440(4)	C(11) – C – C(21)	121.70	120.9(2)

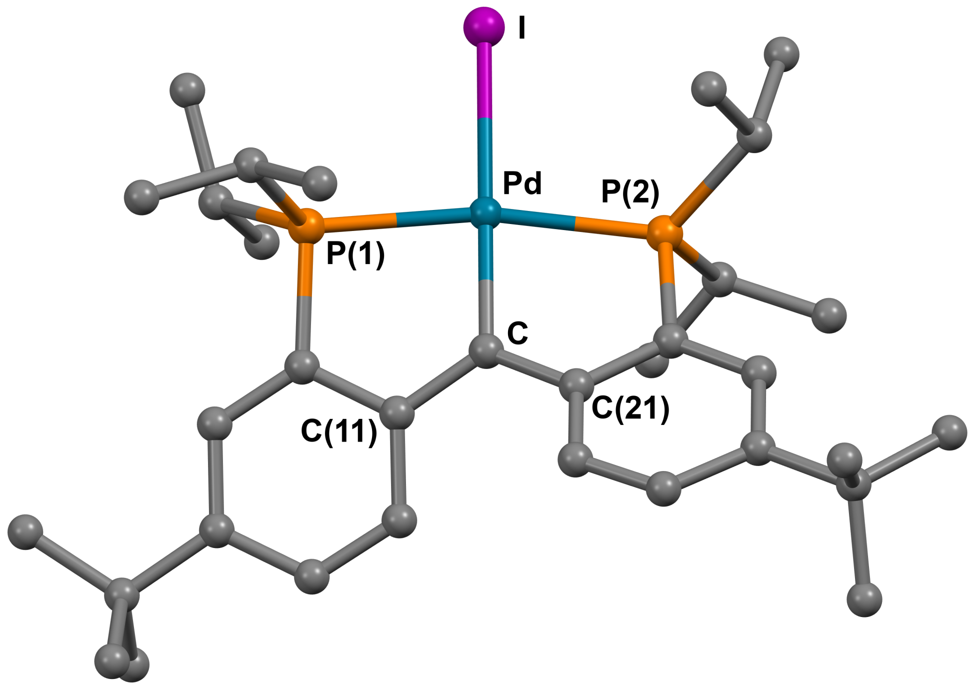

Figure S6. Optimized geometry for [{PC(sp^2)P}^{tBu}PdI][BAr$^F_4$] (**5**).

3.3 [{PC(sp^2)P}^{Me}PdI]$^+$ (5')

Table S5. Optimized coordinates for [{PC(sp^2)P}^{Me}PdI]$^+$ (**5'**).

atom	x	y	z
C	−0.021726	−0.062306	−0.072618
C	−0.006247	−0.068469	1.325822
C	1.235829	−0.059574	2.045266
C	2.445829	0.046025	1.292332
C	2.418601	0.084314	−0.108609
C	1.190679	0.006589	−0.795208
C	1.201547	−0.067629	3.509989
Pd	−0.464898	0.548975	4.443722
P	−1.550644	0.002085	2.397363
C	−2.704000	1.233275	1.580711
H	−3.561853	1.388231	2.241365
C	−2.412649	−1.661146	2.260078
H	−2.718657	−1.853842	1.226331
H	−1.738443	−2.452677	2.597599
H	−2.186734	2.187818	1.451987
C	2.341653	−0.510340	4.316763
C	2.431735	−0.075107	5.681650
C	3.502717	−0.472038	6.488720
C	4.484471	−1.353053	5.981986
C	4.379313	−1.846242	4.666330
C	3.327227	−1.428552	3.839588
P	0.983731	0.961970	6.286712
C	0.550222	0.318876	7.993001
H	−0.363011	0.820654	8.325579
C	1.612099	2.711807	6.554108
H	2.392538	2.729197	7.322238
H	2.011024	3.105673	5.615638
H	0.357243	−0.755920	7.939608
I	−2.703953	1.376844	5.698260
H	3.579837	−0.128528	7.516932
H	5.108482	−2.560359	4.295070
H	3.232638	−1.854867	2.846796
H	3.394276	0.156351	1.806738
H	3.345628	0.185426	−0.665280
H	−0.961902	−0.086435	−0.617276
H	0.772142	3.337110	6.871575
H	1.358084	0.516985	8.705504
H	−3.295253	−1.648618	2.906743
H	−3.050625	0.863937	0.609634
H	5.306450	−1.670261	6.617906
H	1.172477	0.026224	−1.881462

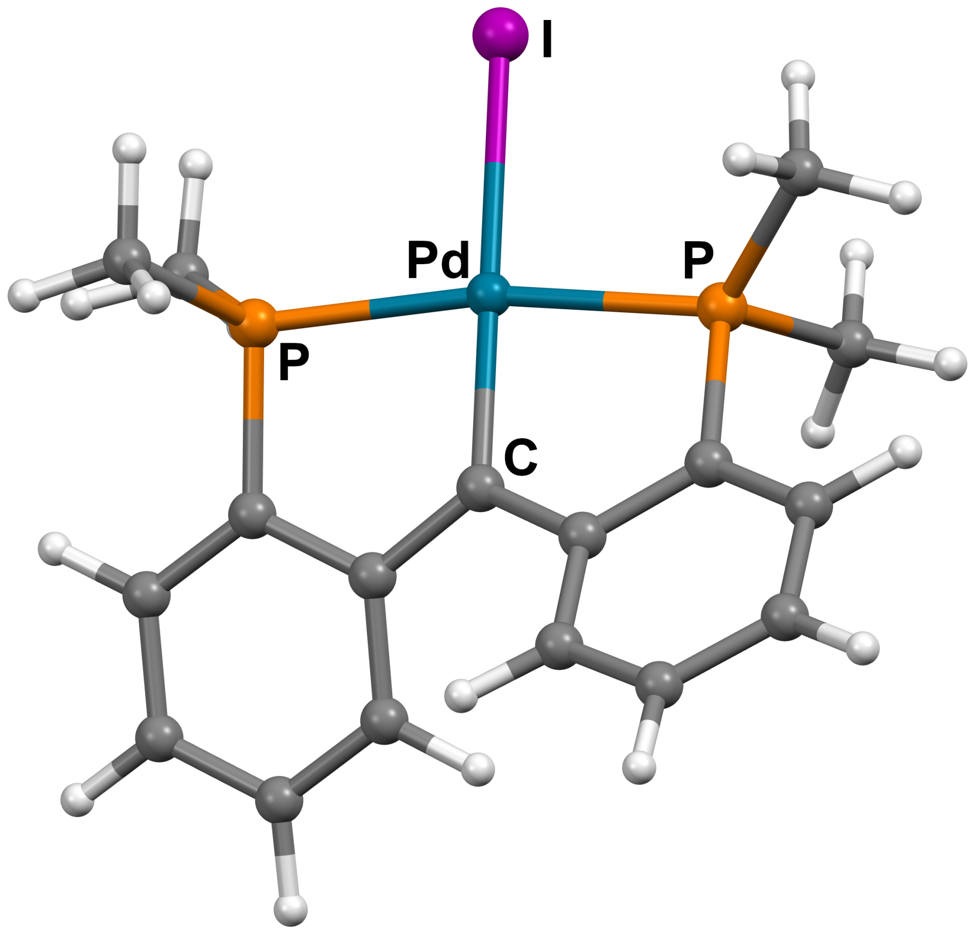

Figure S7. Optimized geometry for $[\{PC(sp^2)P\}^{Me}PdI]^+$ (**5'**).

4 NMR Spectra

4.1 NMR Spectra for [{PC(sp^2)P}^{tBu}PdI][BAr$^F_4$] (5)

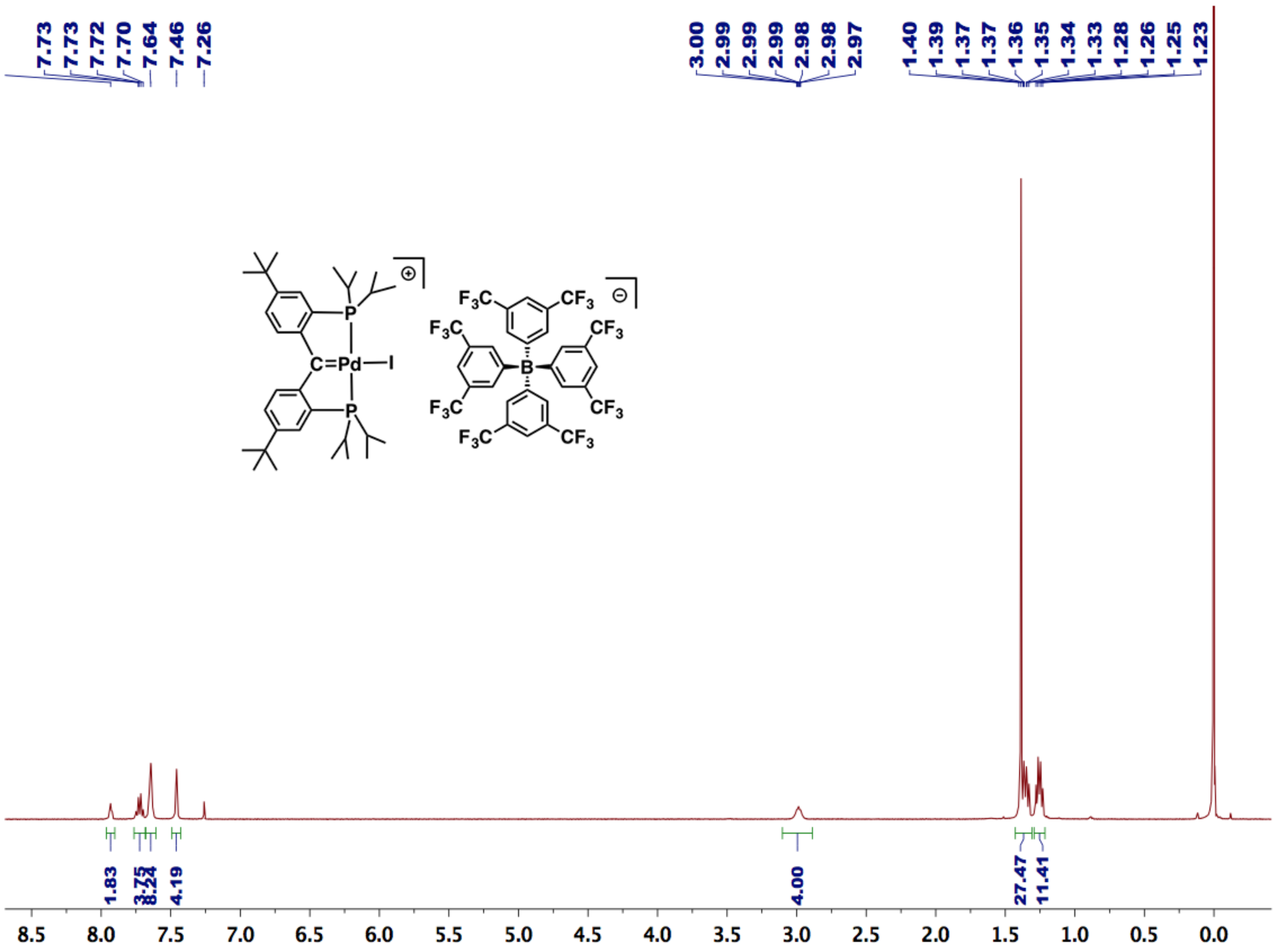

Figure S8. ^{1}H NMR spectrum for [{PC(sp^2)P}^{tBu}PdI][BAr$^F_4$] (**5**).

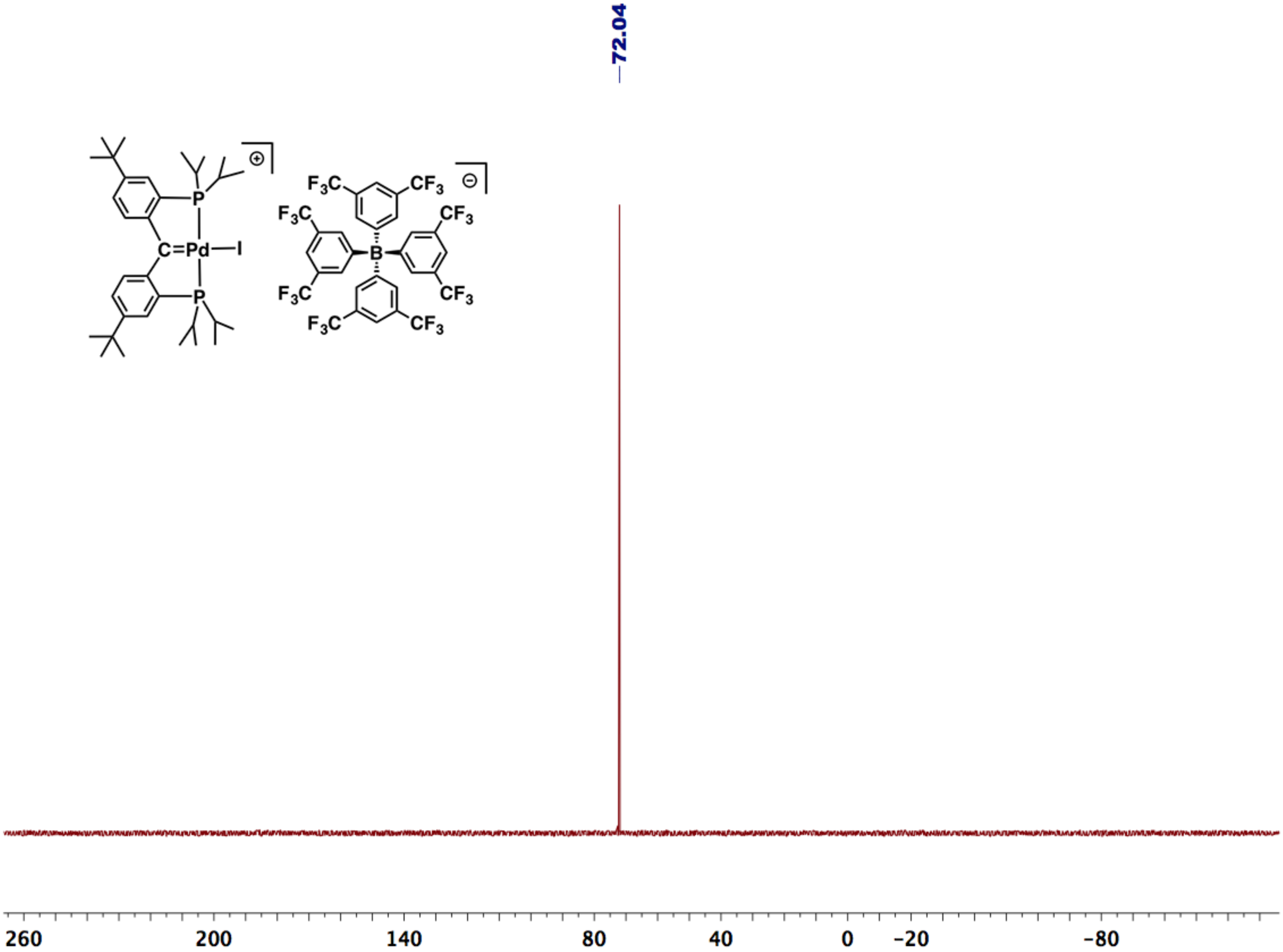

Figure S9. $^{31}P\{^{1}H\}$ NMR spectrum for $[\{PC(sp^2)P\}^{tBu}PdI][BAr^F_4]$ (**5**).

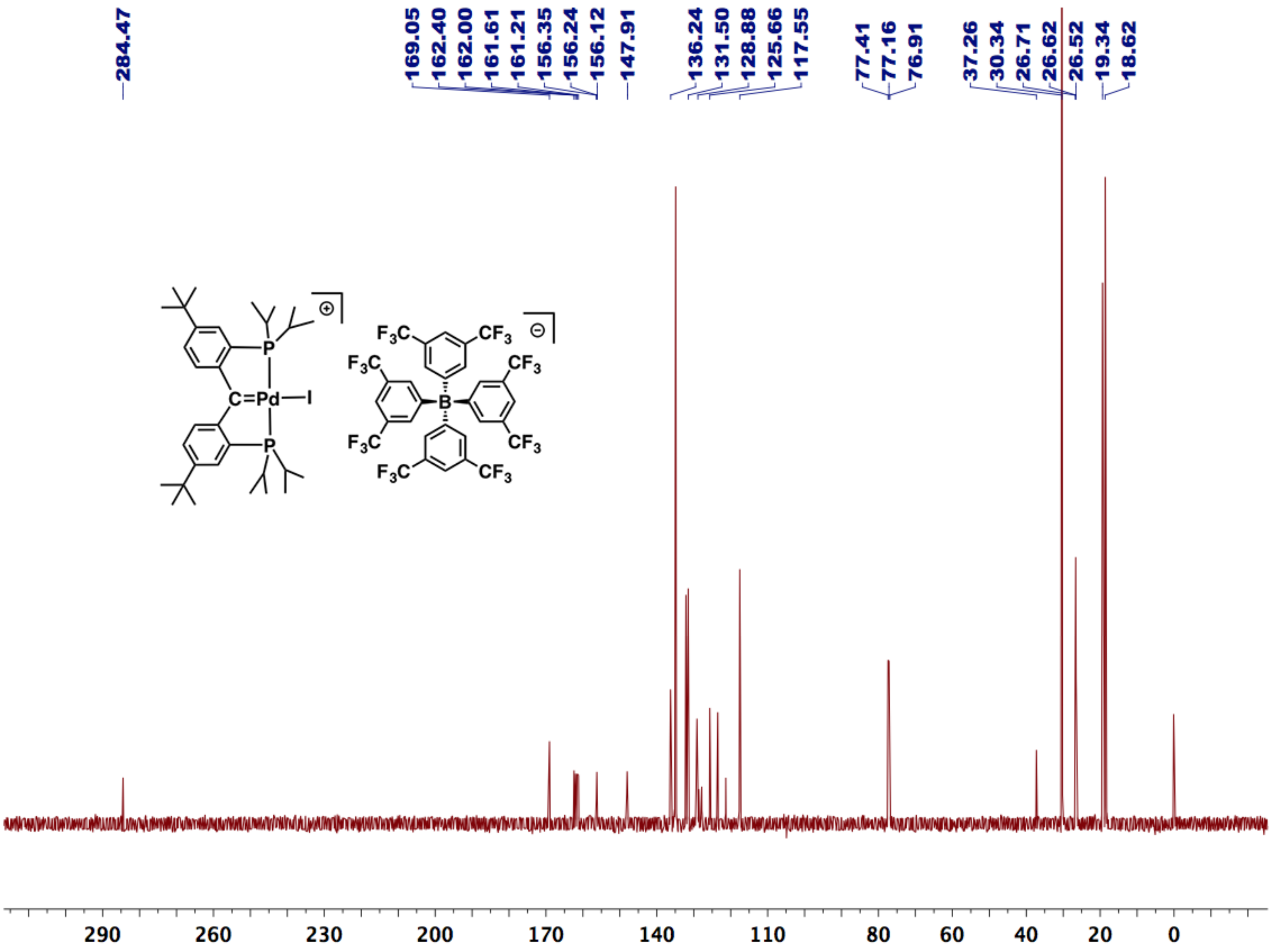

Figure S10. $^{13}C\{^1H\}$ NMR spectrum for $[\{PC(sp^2)P\}^{tBu}PdI][BAr^F_4]$ (**5**).

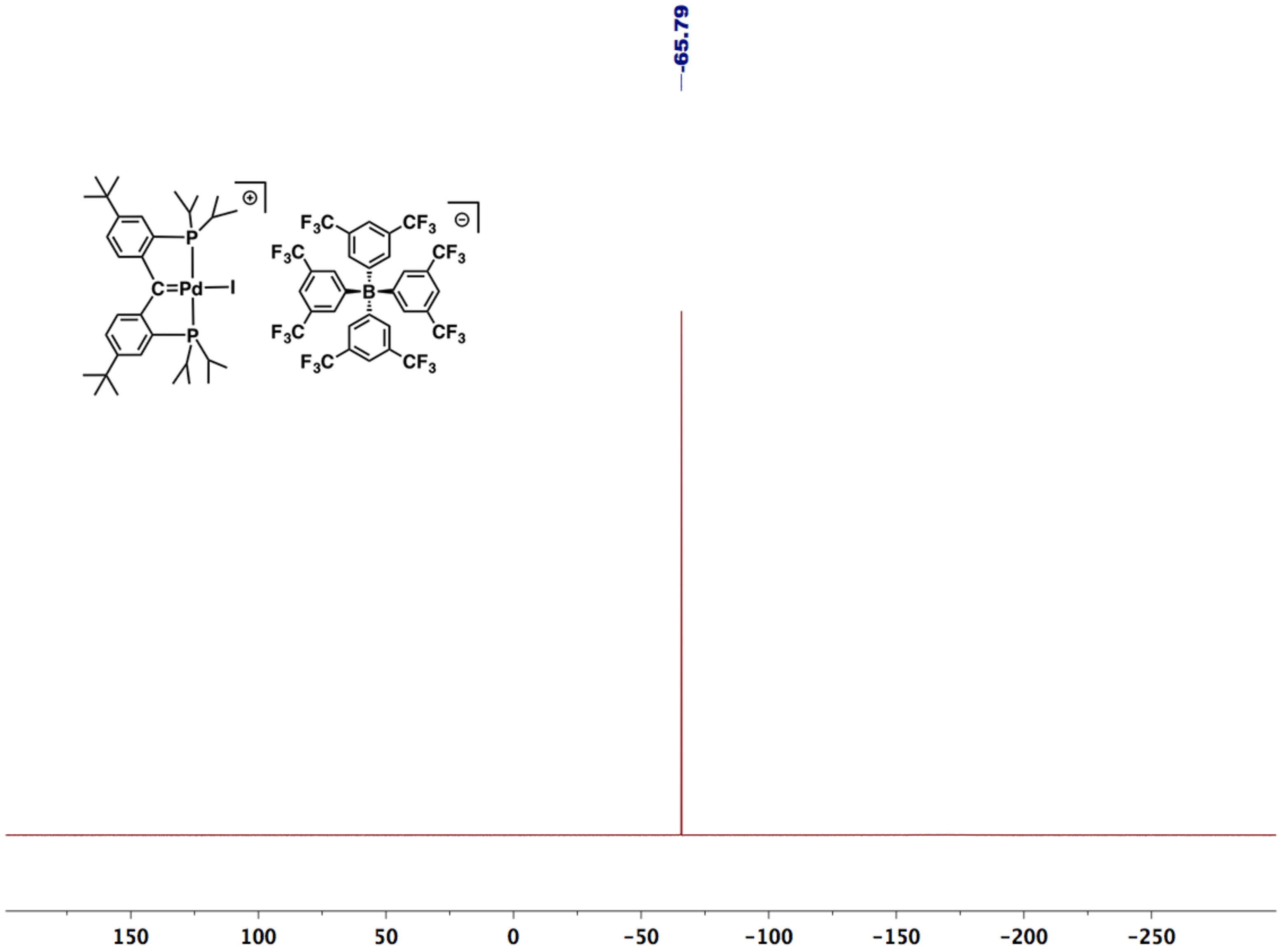

Figure S11. ^{19}F NMR spectrum for [{PC(sp^2)P}^{tBu}PdI][BAr$^F_4$] (**5**).

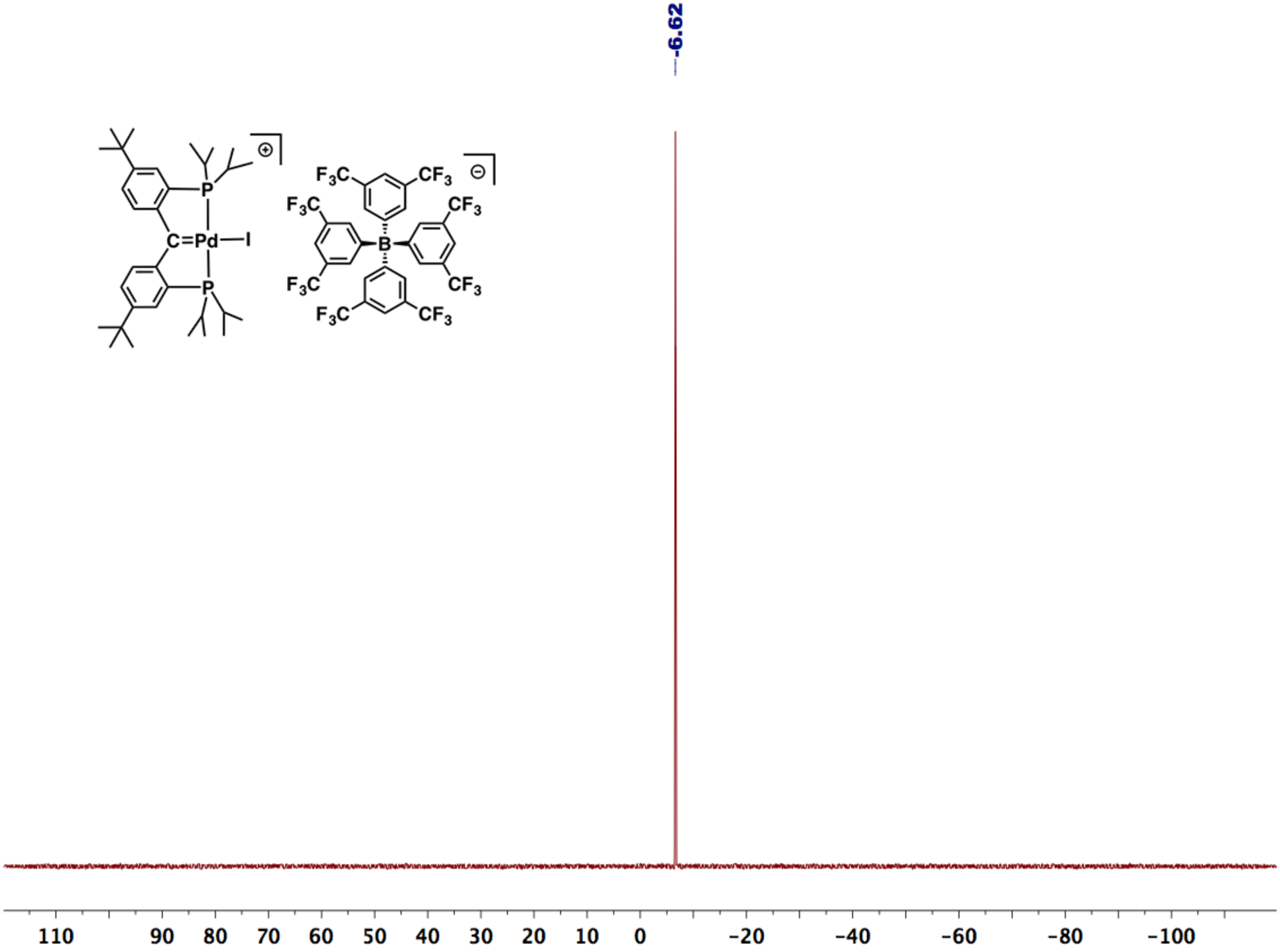

Figure S12. ^{11}B NMR spectrum for [{PC(sp^2)P}^{tBu}PdI][BAr$^F_4$] (**5**).

4.2 NMR Spectra for [{PC(sp^3)HP}^{tBu}PdI] (6)

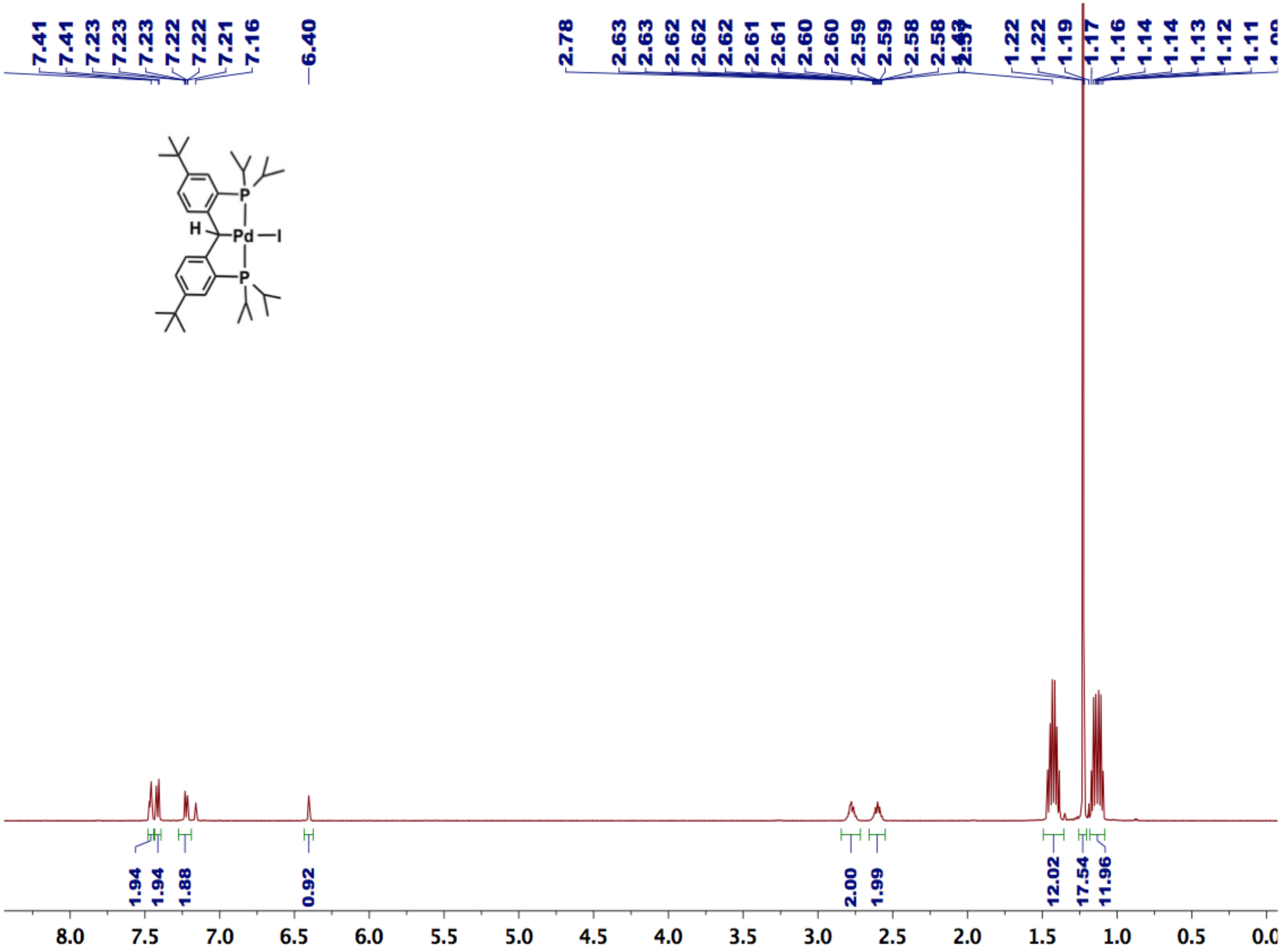

Figure S13. ^{1}H NMR spectrum for [{PC(sp^3)HP}^{tBu}PdI] (**6**).

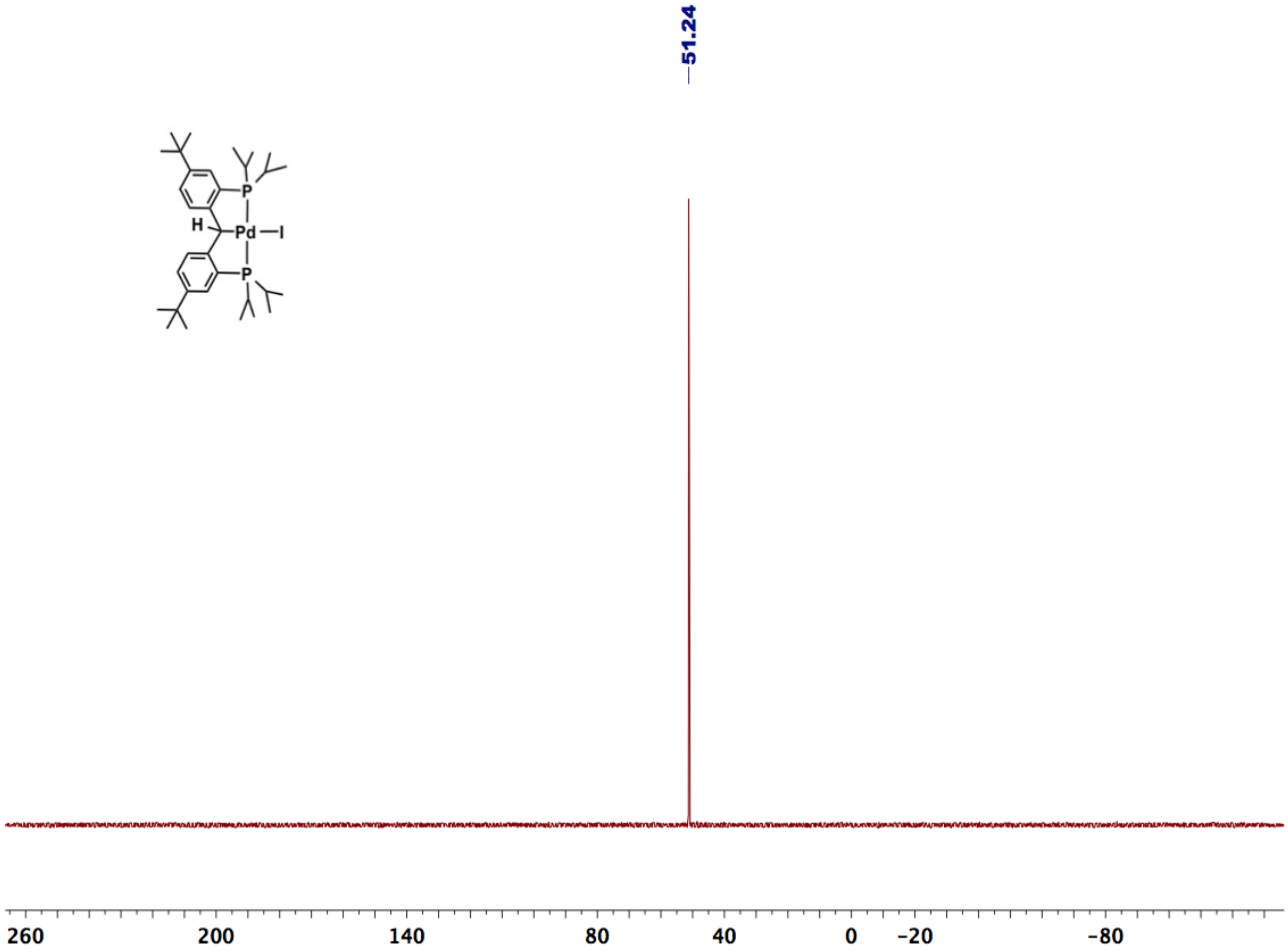

Figure S14. $^{31}P\{^{1}H\}$ NMR spectrum for [{PC(sp^{3})HP}^{tBu}PdI] (**6**).

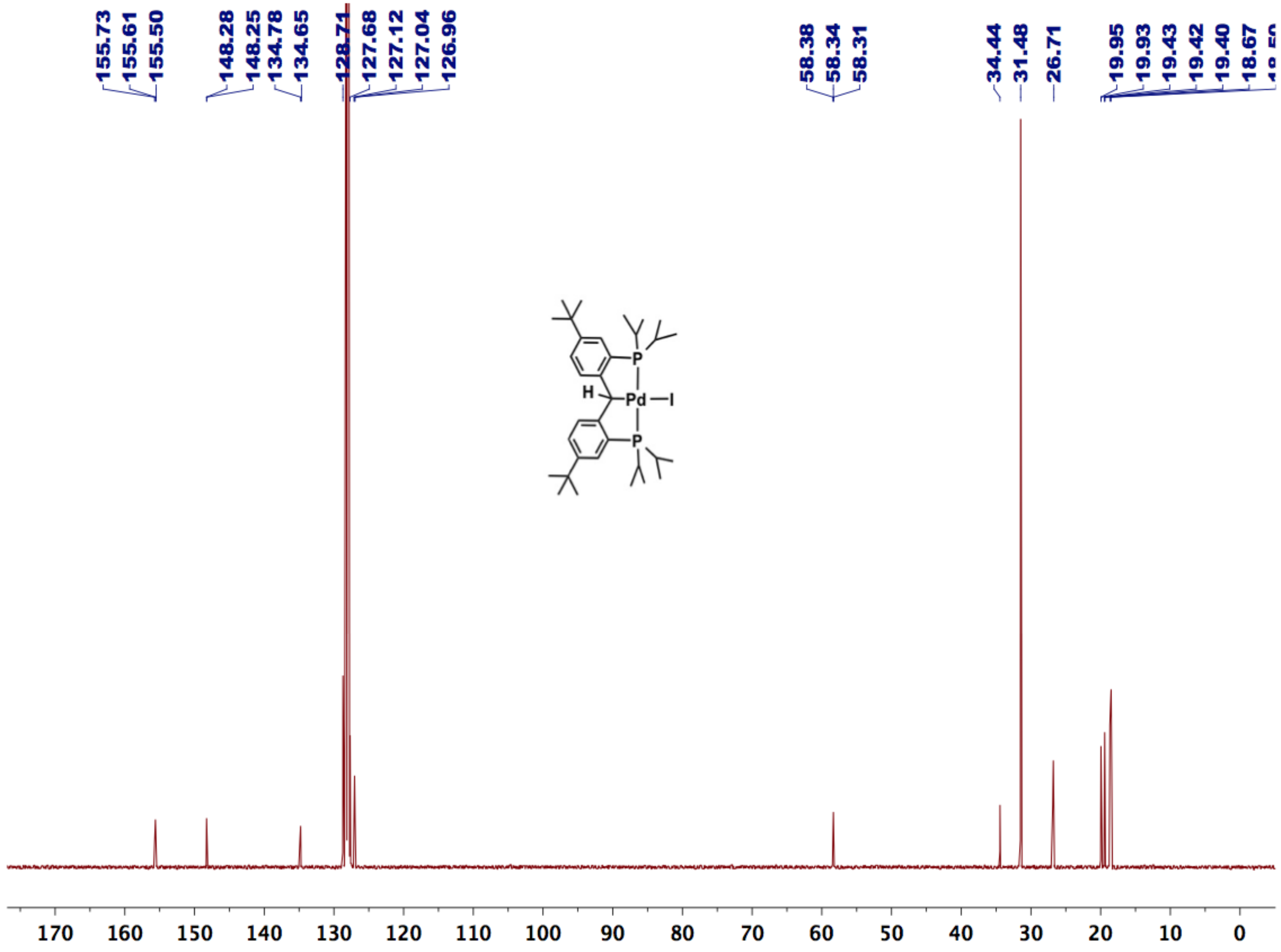

Figure S15. $^{13}C\{^{1}H\}$ NMR spectrum for [{PC(sp^{3})HP}^{tBu}PdI] (**6**).

4.3 NMR Spectra for [{PC(sp^3)(SPh)P}^{tBu}PdI] (7)

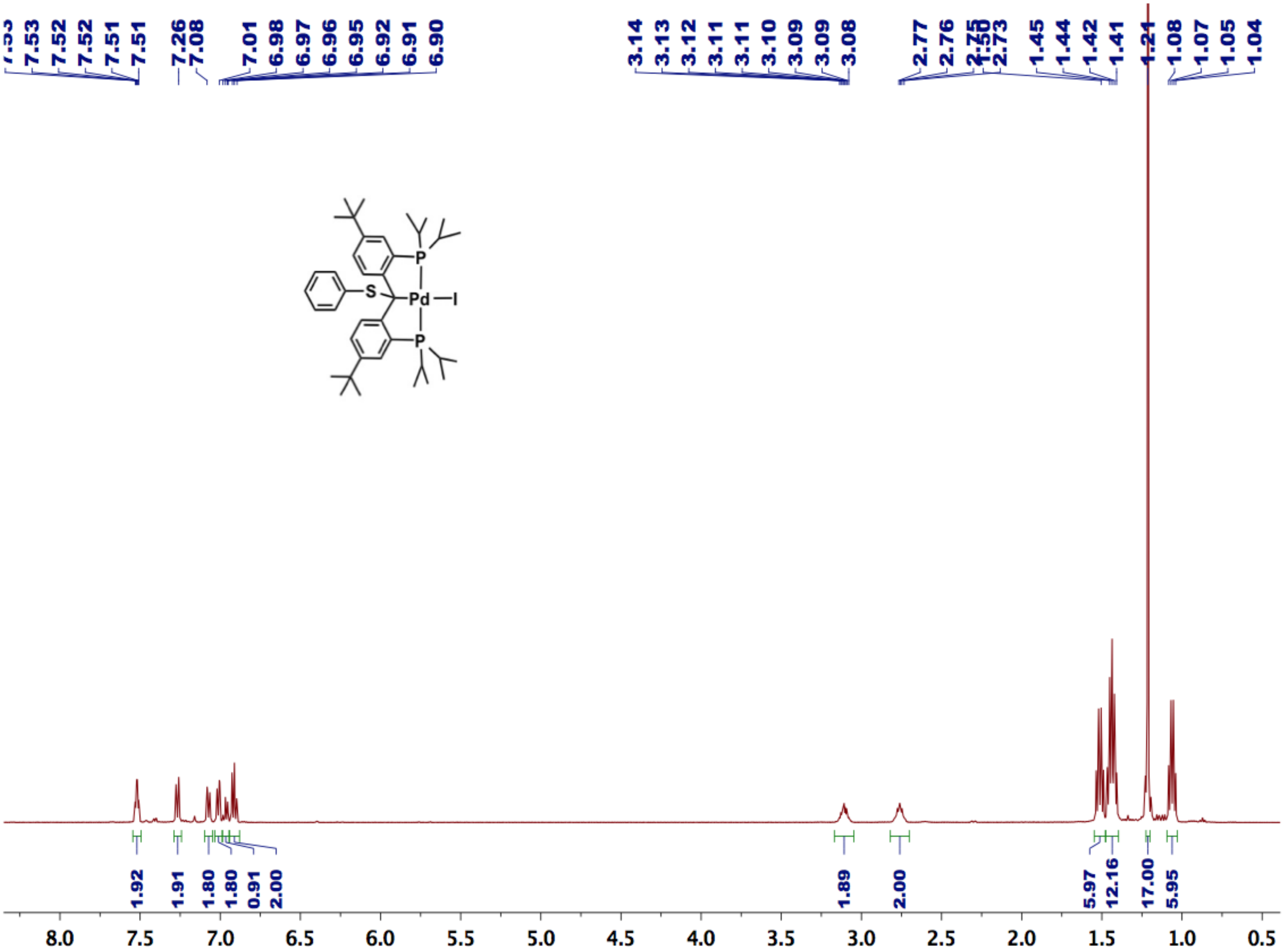

Figure S16. ^{1}H NMR spectrum for [{PC(sp^3)(SPh)P}^{tBu}PdI] (**7**).

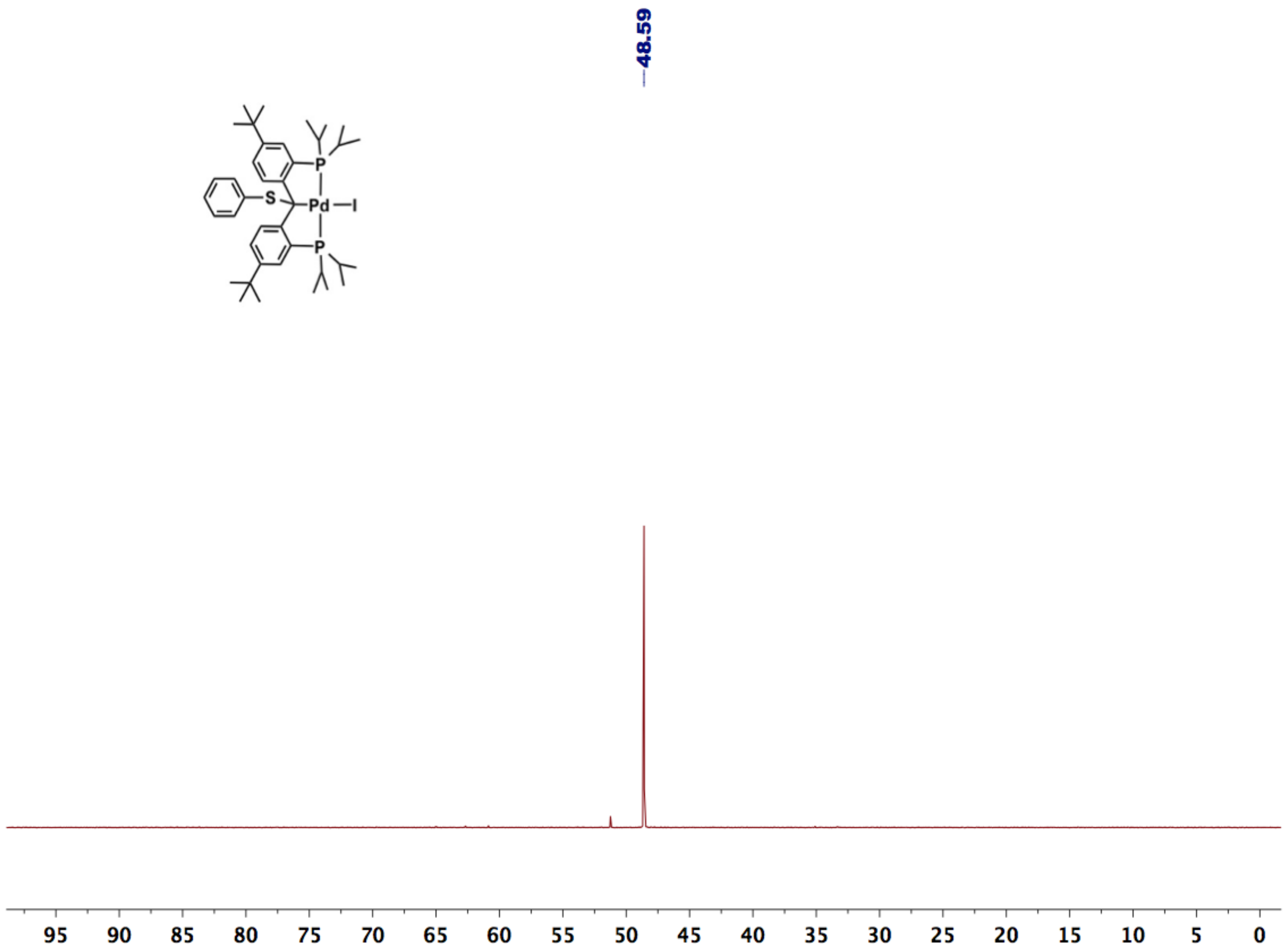

Figure S17. $^{31}P\{^{1}H\}$ NMR spectrum for [{PC(sp^3)(SPh)P}^{tBu}PdI] (**7**).

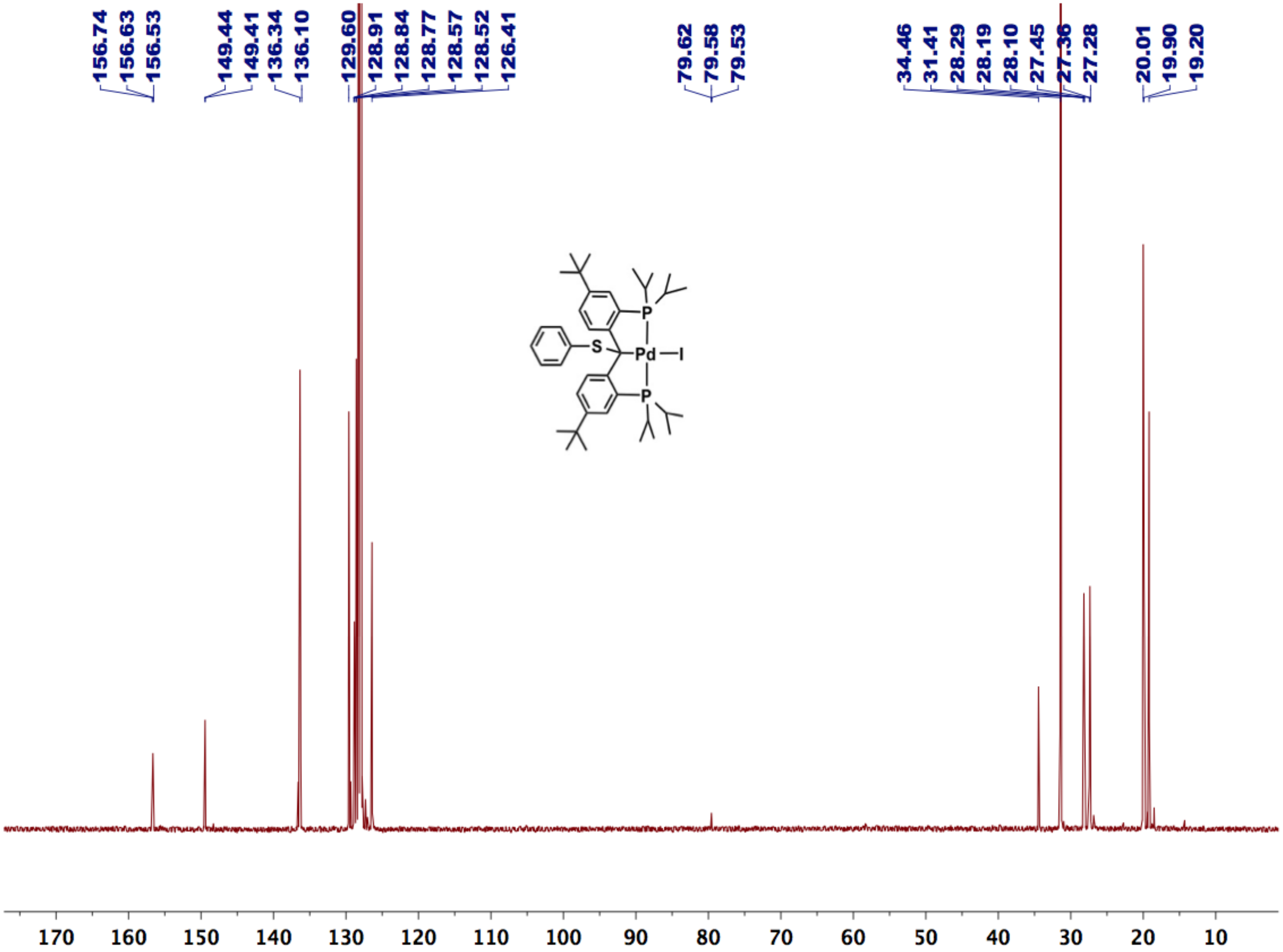

Figure S18. $^{13}C\{^1H\}$ NMR spectrum for [{PC(sp^3)(SPh)P}^{tBu}PdI] (**7**).

4.4 NMR Spectra for [{PC(sp^3)(SPh)P}^{tBu}PdOTf] (8)

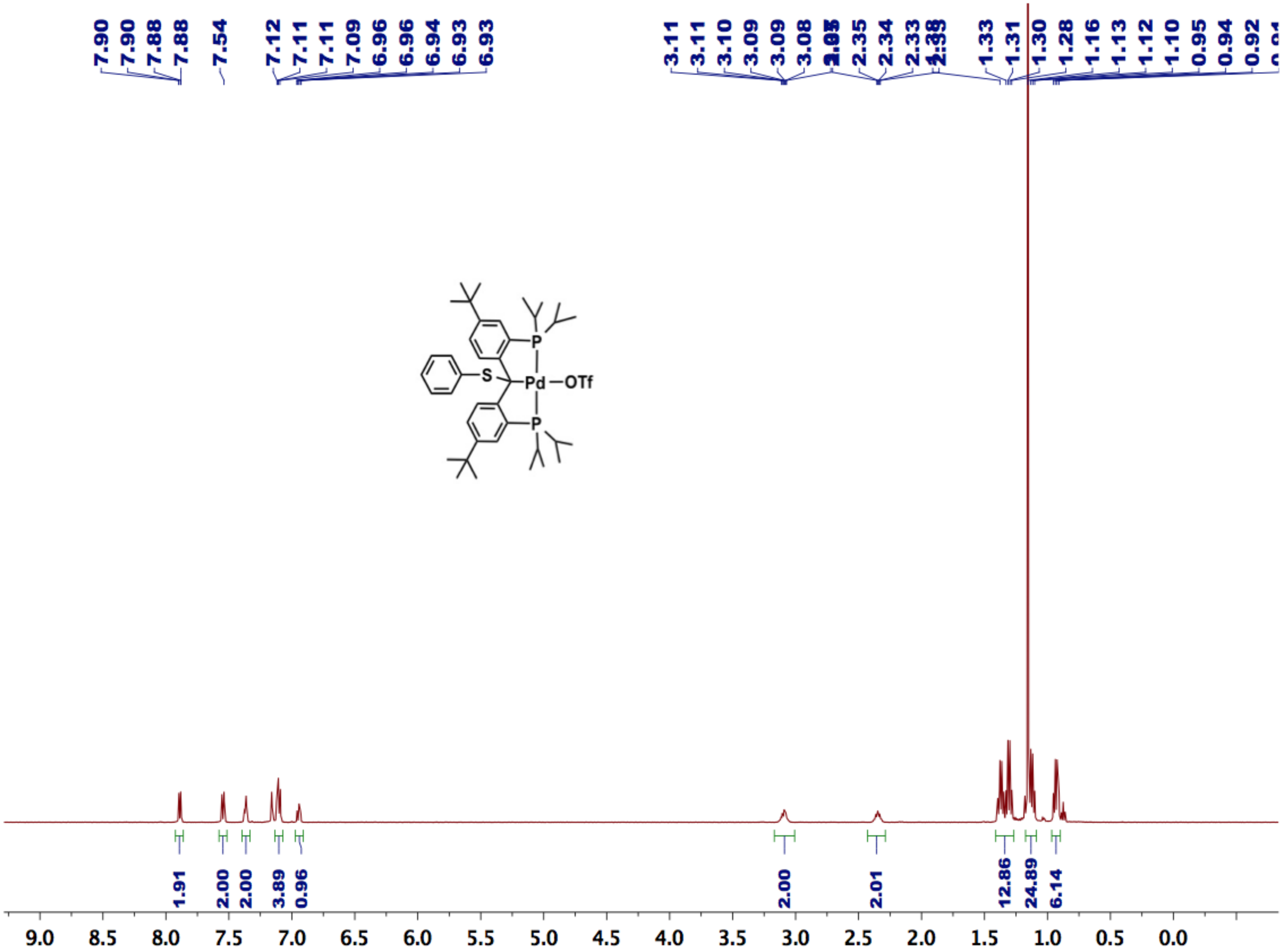

Figure S19. ^{1}H NMR spectrum for [{PC(sp^3)(SPh)P}^{tBu}PdOTf] (**8**).

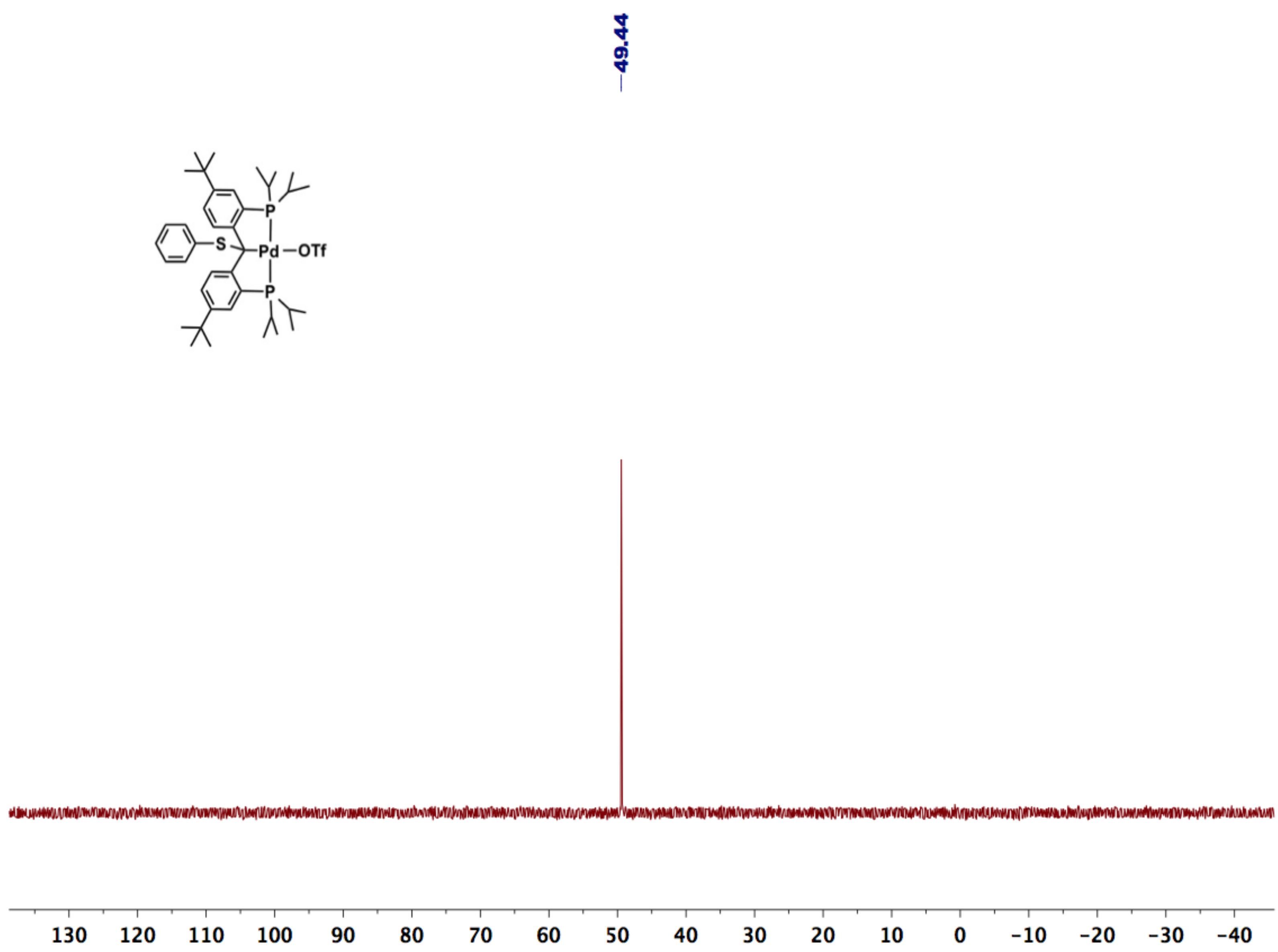

Figure S20. $^{31}P\{^{1}H\}$ NMR spectrum for [{PC(sp^{3})(SPh)P}$^{t\mathrm{Bu}}$PdOTf] (**8**).

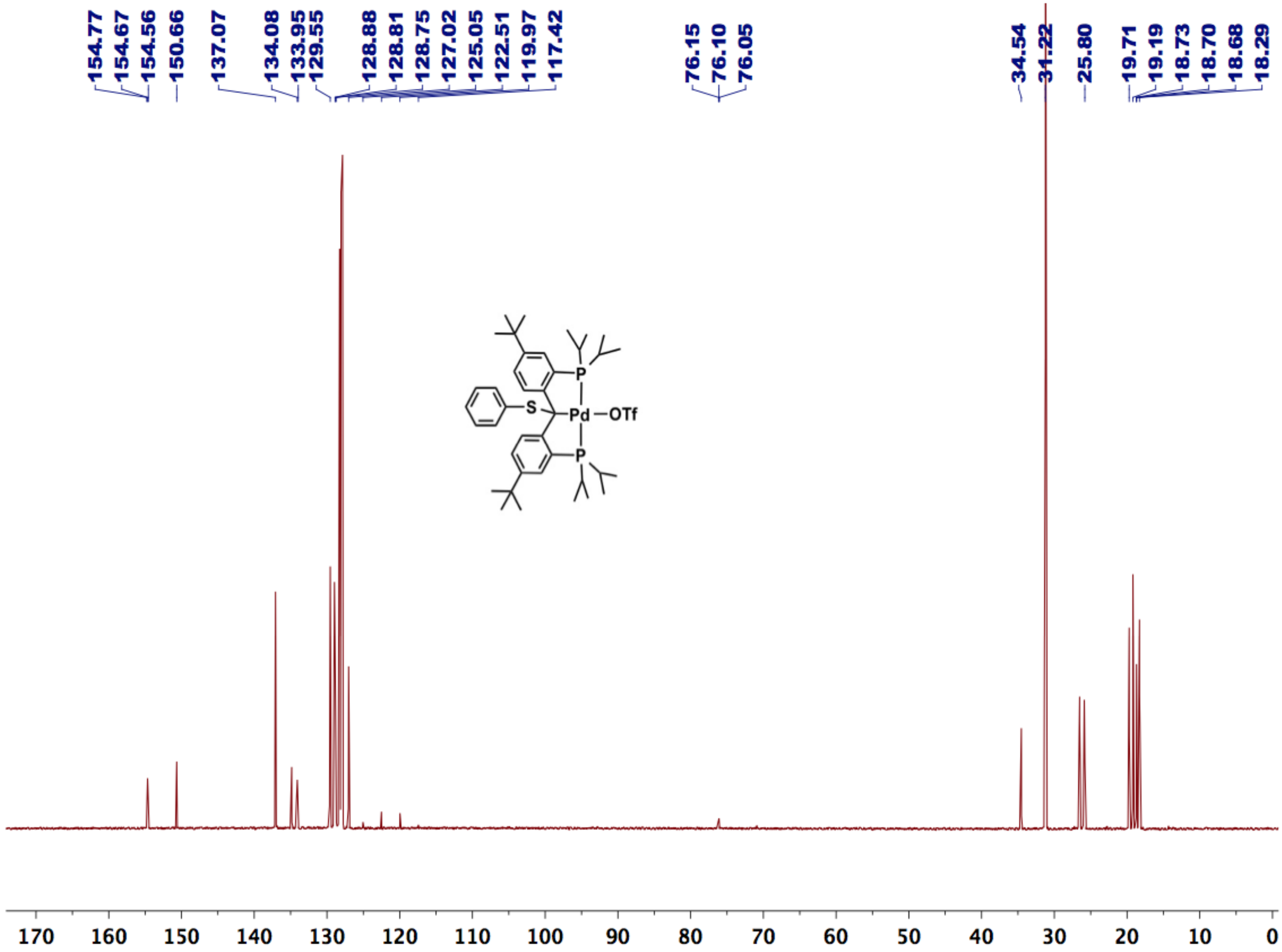

Figure S21. $^{13}C\{^1H\}$ NMR spectrum for [{PC(sp^3)(SPh)P}^{tBu}PdOTf] (**8**).

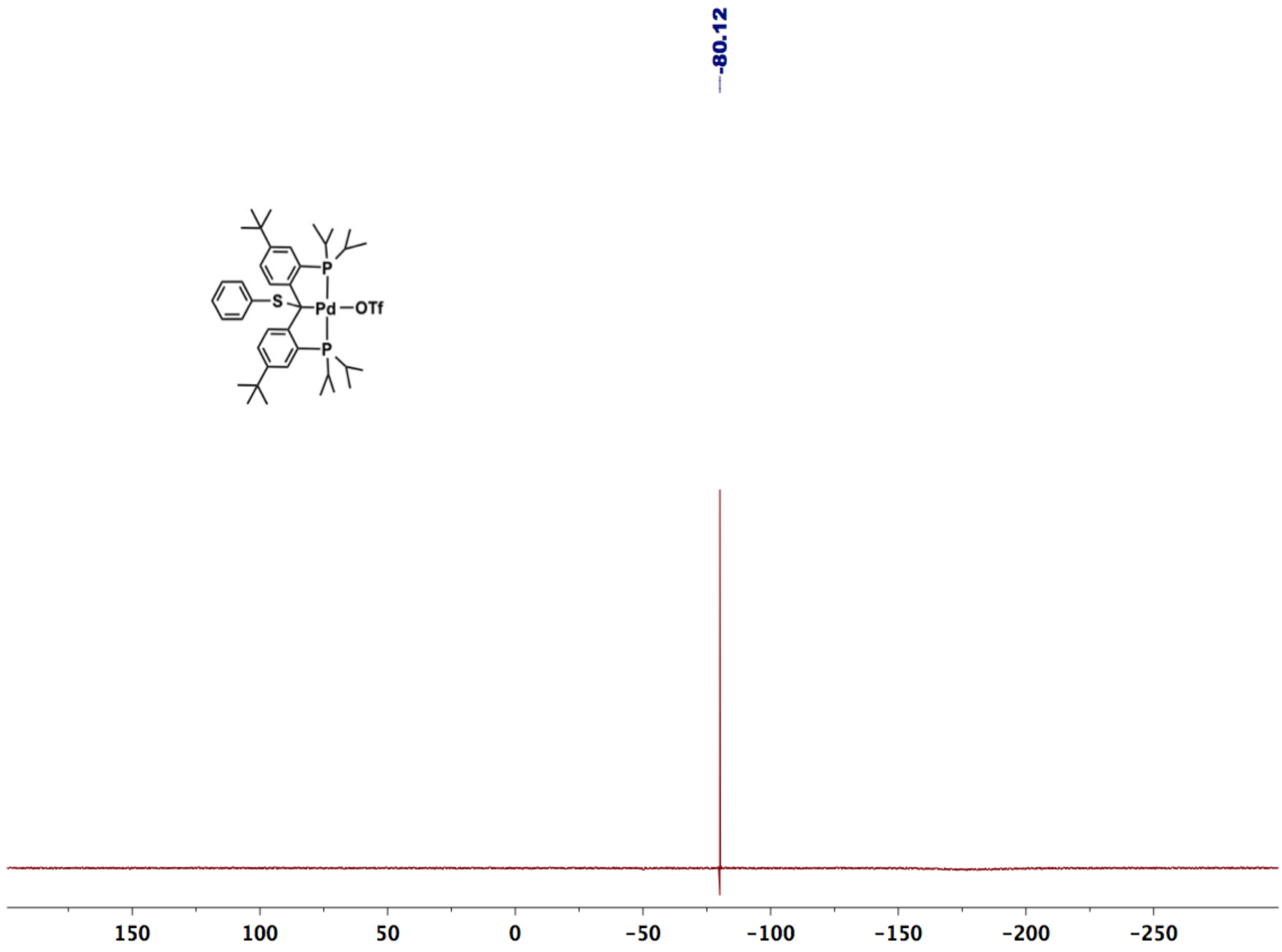

Figure S22. ^{19}F NMR spectrum for [{PC(sp^3)(SPh)P}^{tBu}PdOTf] (**8**).

4.5 NMR Spectra for [{PC(sp³)(SPh)P}^{tBu}PdSPh] (9)

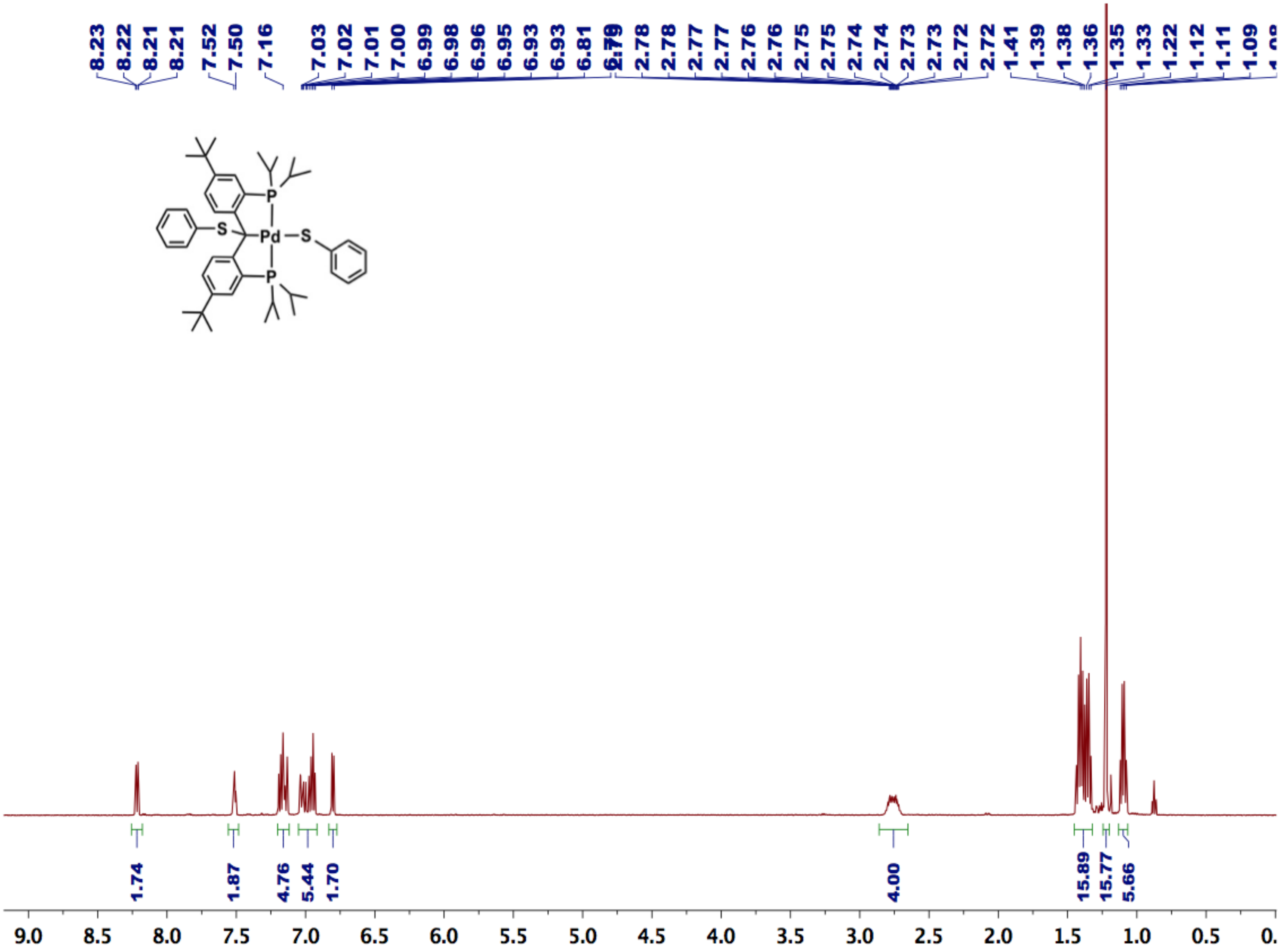

Figure S23. ^{1}H NMR spectrum for [{PC(sp³)(SPh)P}^{tBu}PdSPh] (**9**).

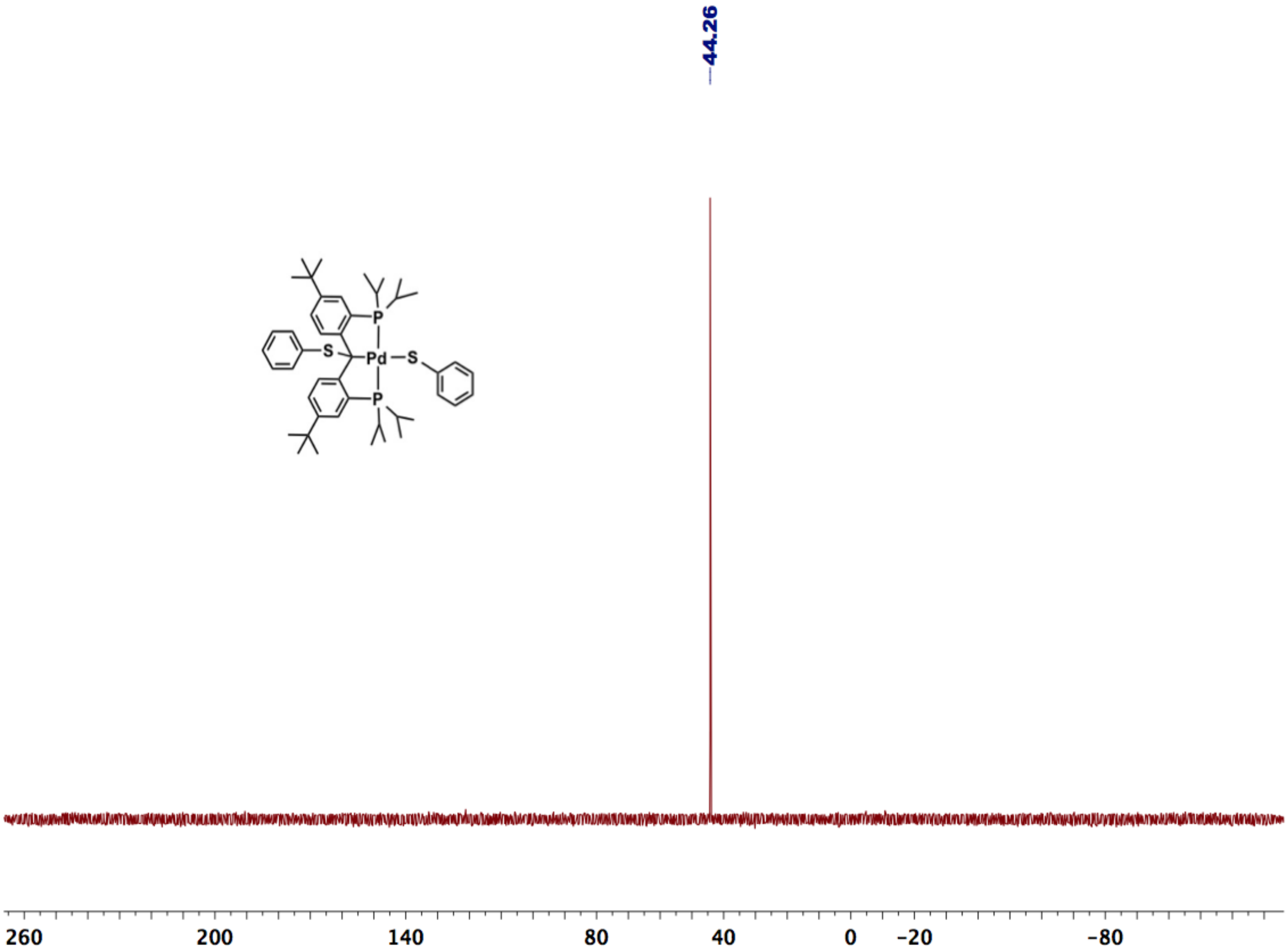

Figure S24. $^{31}P\{^{1}H\}$ NMR spectrum for [{PC(sp^3)(SPh)P}^{tBu}PdSPh] (**9**).

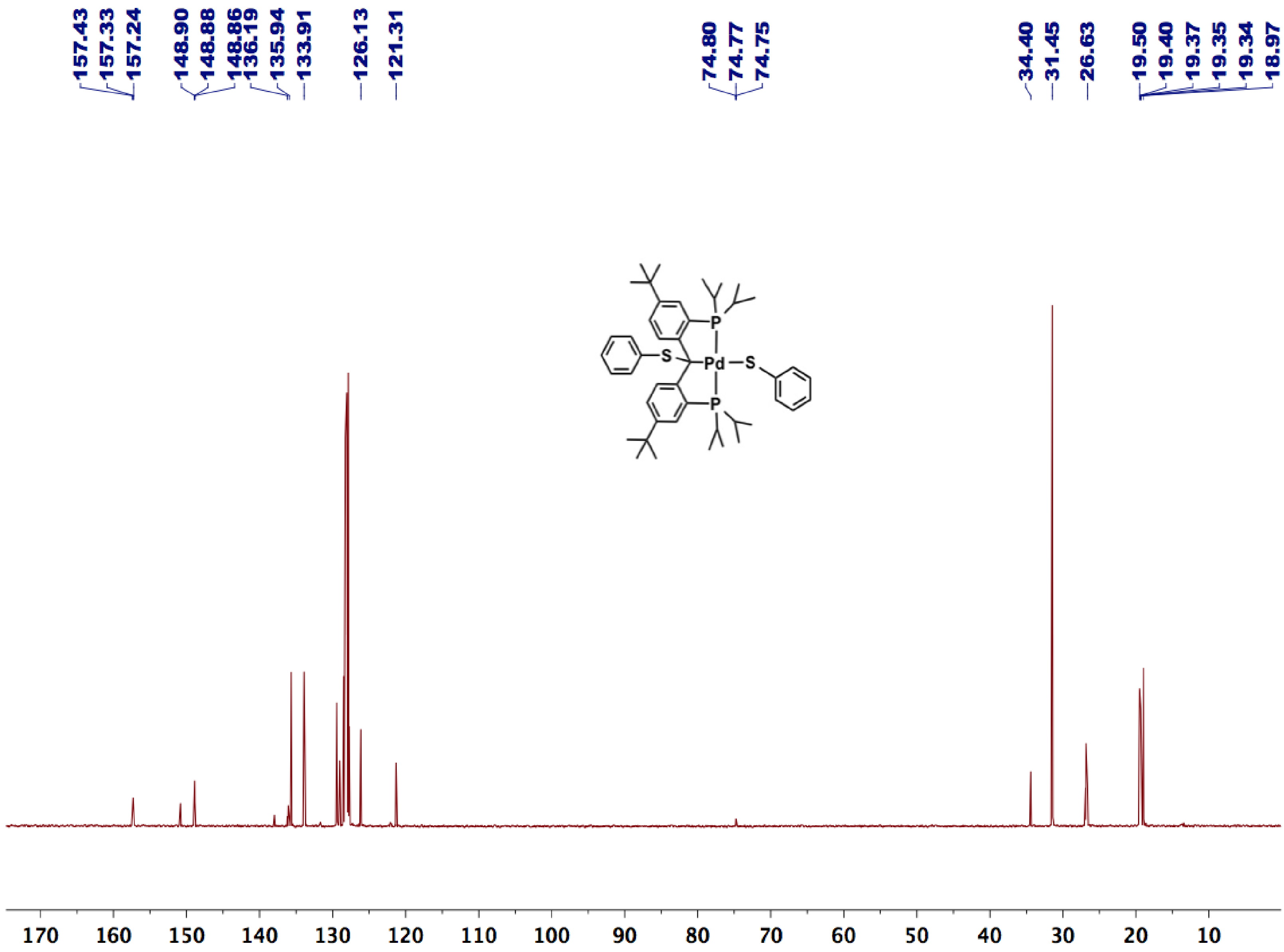

Figure S25. $^{13}C\{^1H\}$ NMR spectrum for [{PC(sp^3)(SPh)P}^{tBu}PdSPh] (**9**).

4.6 NMR Spectra for [{PC(sp^3)(NH^{p}Tol)P}^{tBu}PdI] (10)

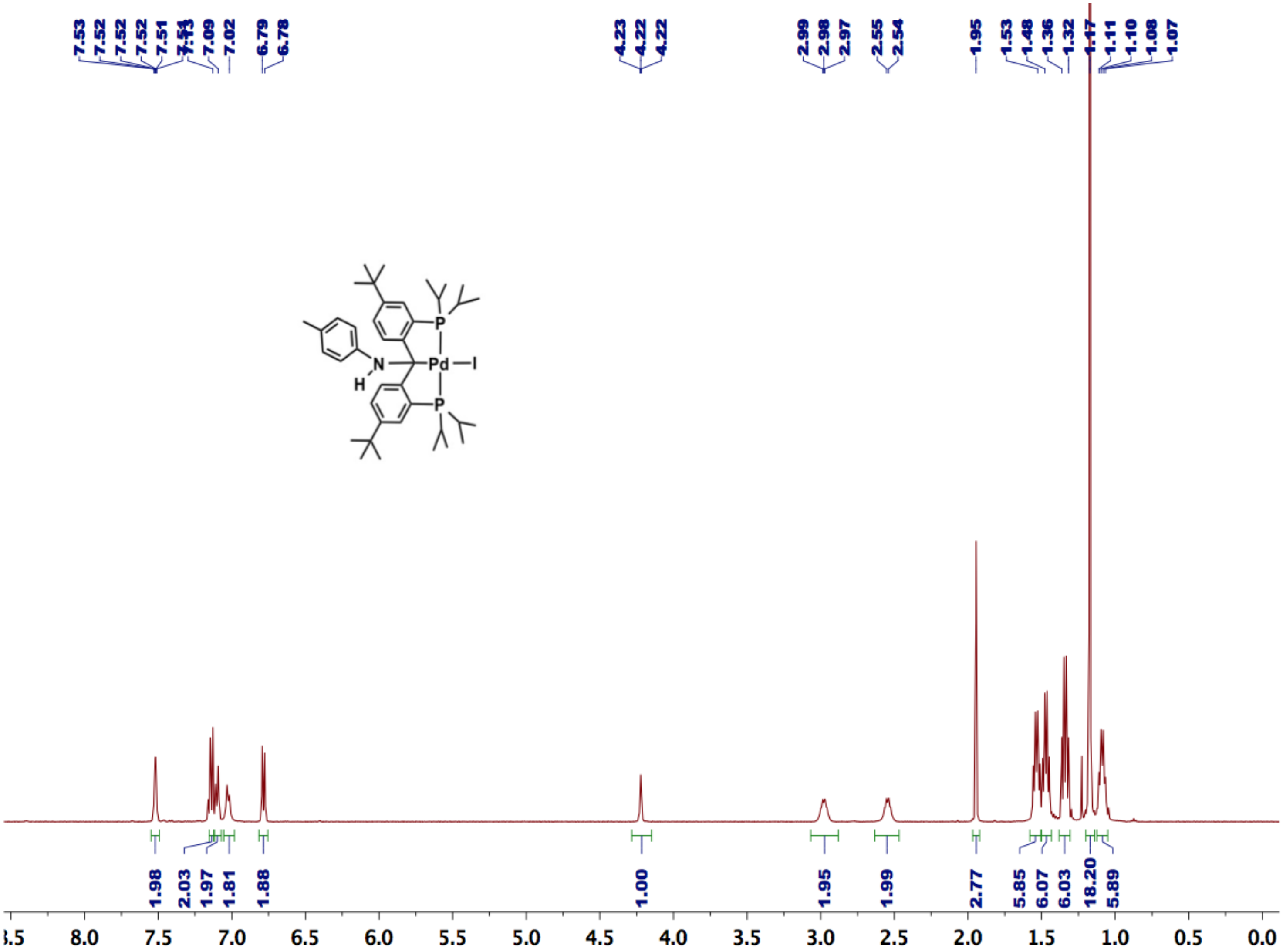

Figure S26. ^{1}H NMR spectrum for [{PC(sp^3)(NH^{p}Tol)P}^{tBu}PdI] (**10**).

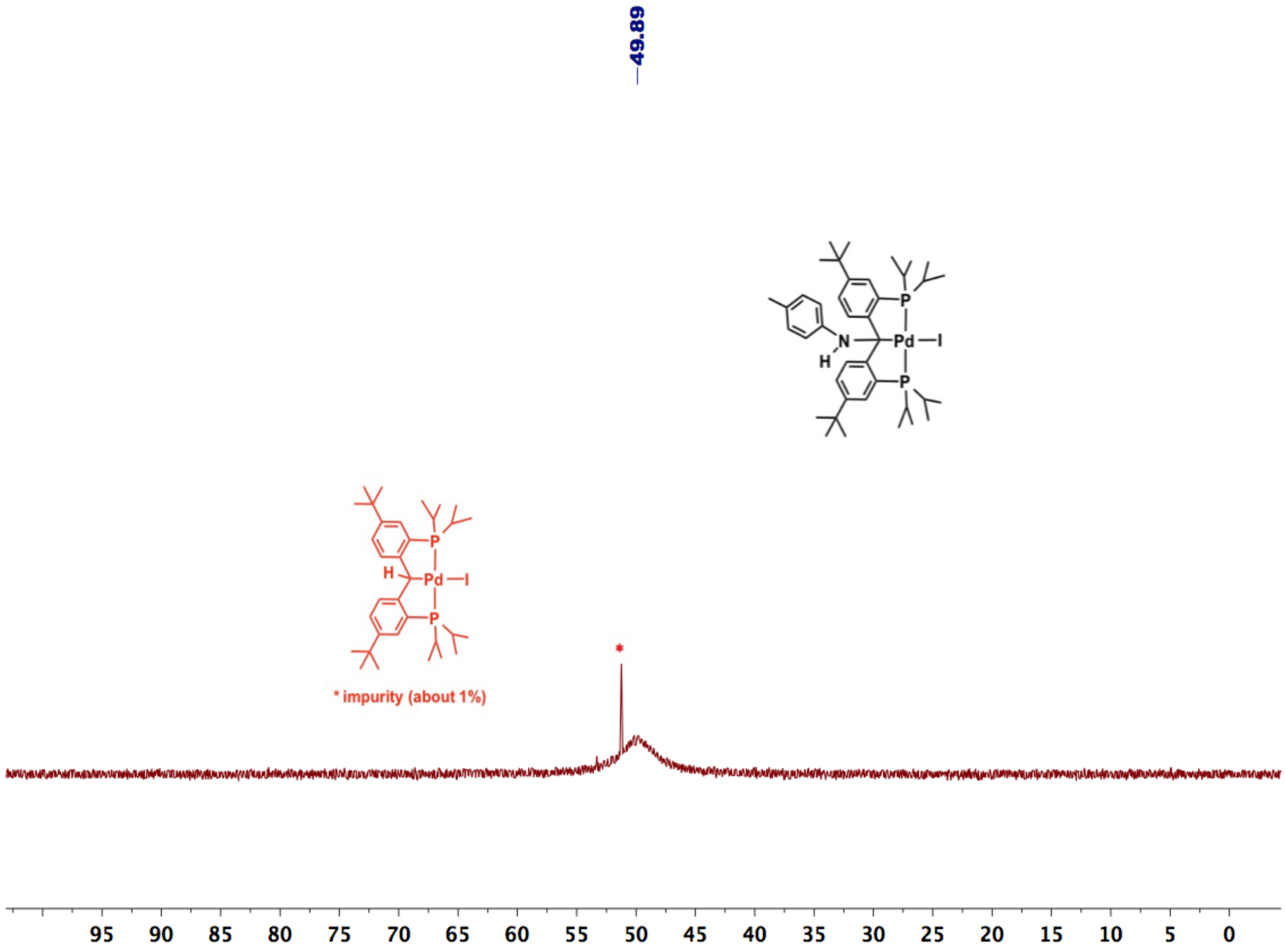

Figure S27. $^{31}P\{^{1}H\}$ NMR spectrum for [{PC(sp^{3})(NH^{p}Tol)P}^{tBu}PdI] (**10**).

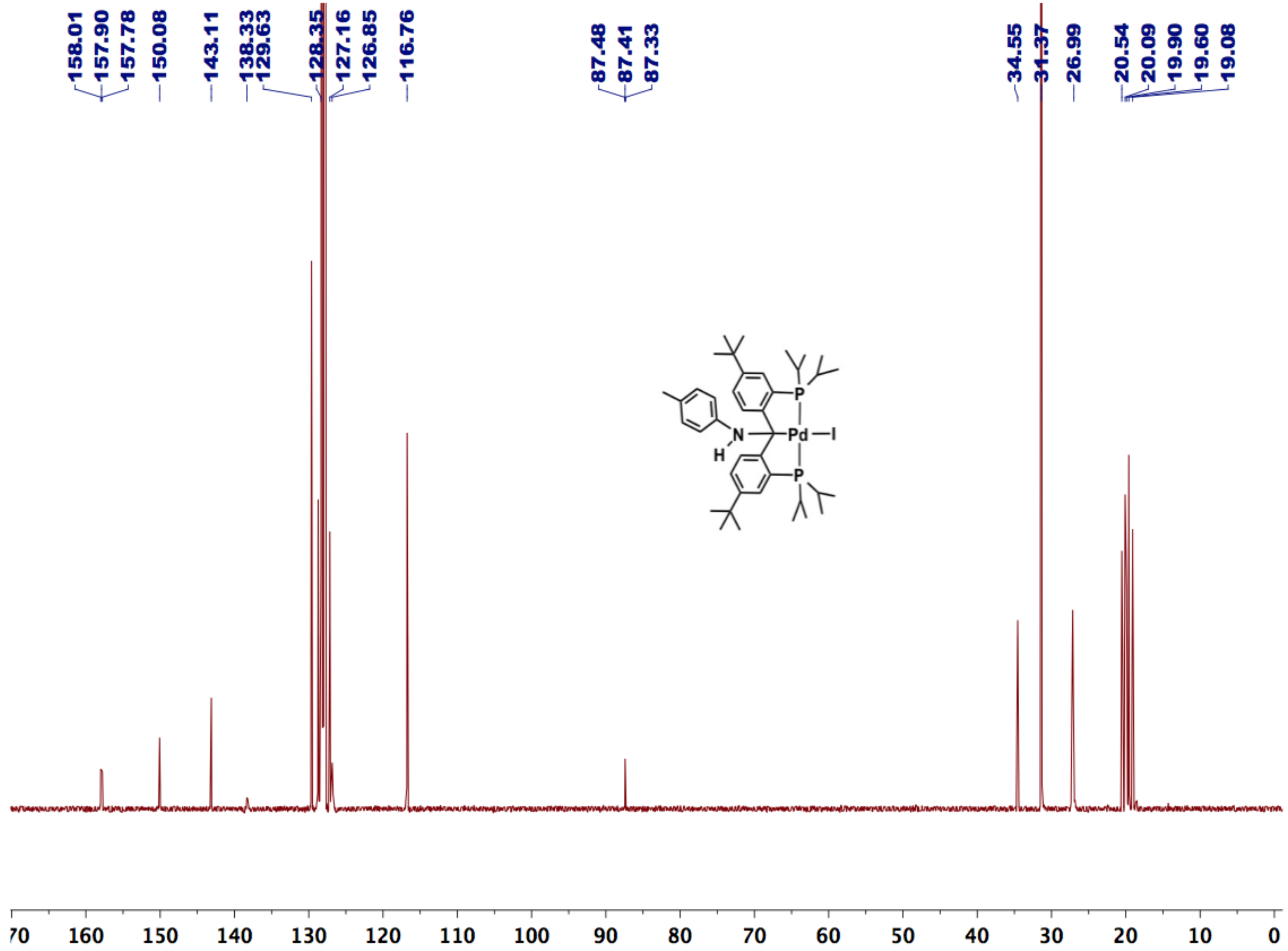

Figure S28. $^{13}C\{^1H\}$ NMR spectrum for [{PC(sp^3)(NH^{p}Tol)P}^{tBu}PdI] (**10**).

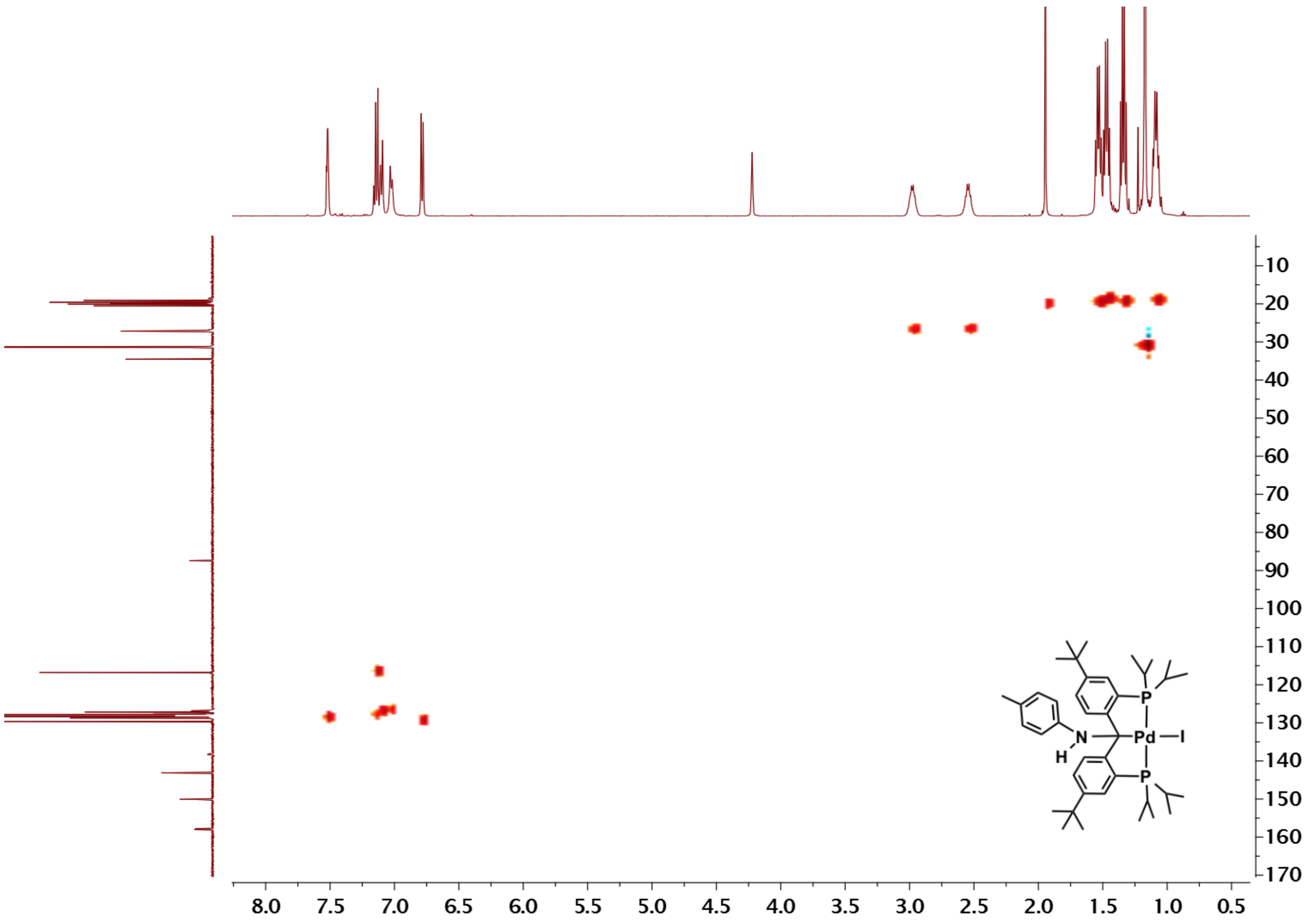

Figure S29. ^{1}H-^{13}C HSQC NMR spectrum for [{PC(sp^{3})(NH^{p}Tol)P}^{tBu}PdI] (**10**).

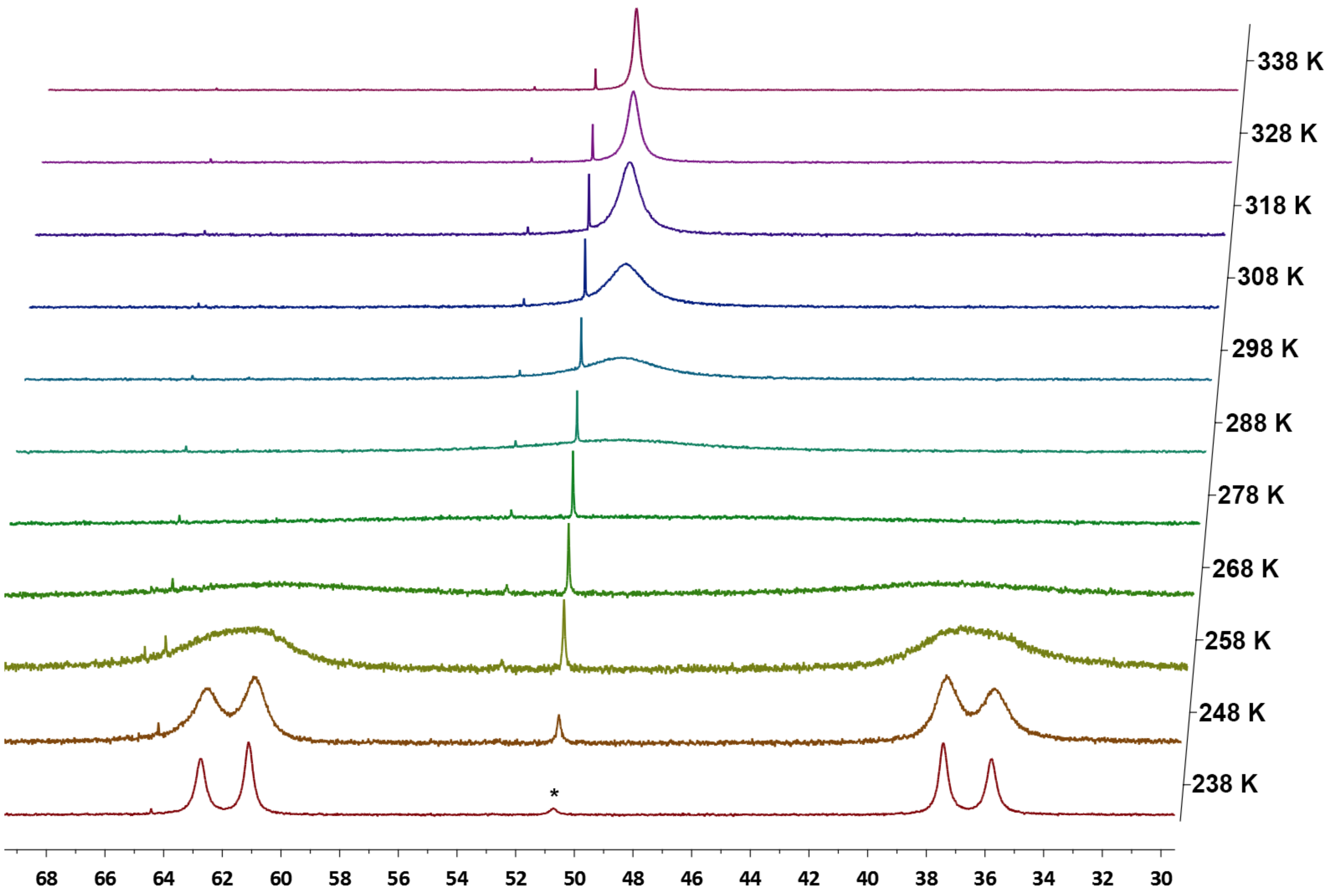

Figure S30. Variable temperature $^{31}P\{^{1}H\}$ NMR spectra for [{PC(sp^3)(NH^{p}Tol)P}^{tBu}PdI] (**10**). (*) designates the small amount of impurity of **6**).

4.7 NMR Spectra for [{PC(sp^3)(OPh)P}^{tBu}PdI] (11)

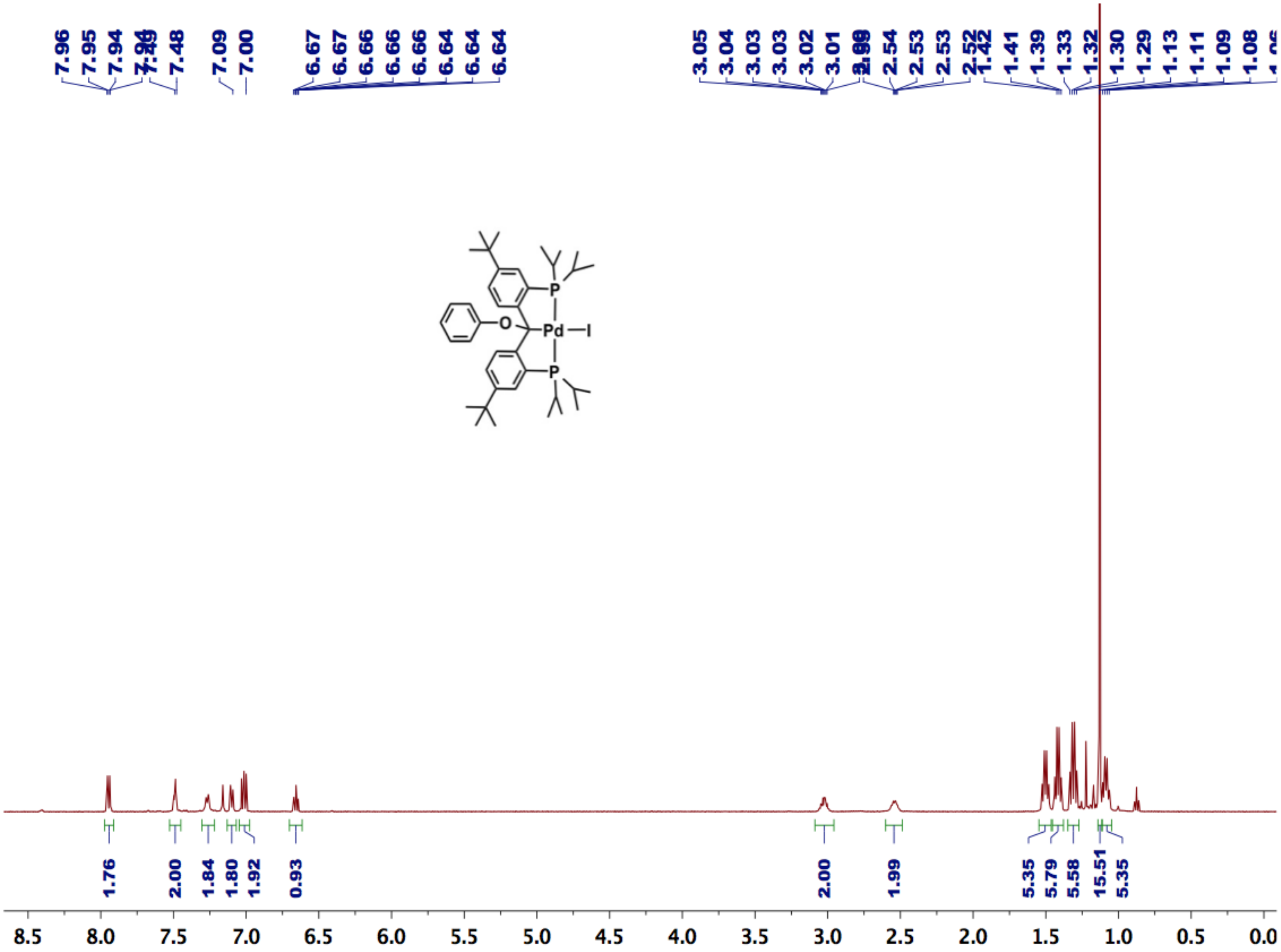

Figure S31. ^{1}H NMR spectrum for [{PC(sp^3)(OPh)P}^{tBu}PdI] (**11**).

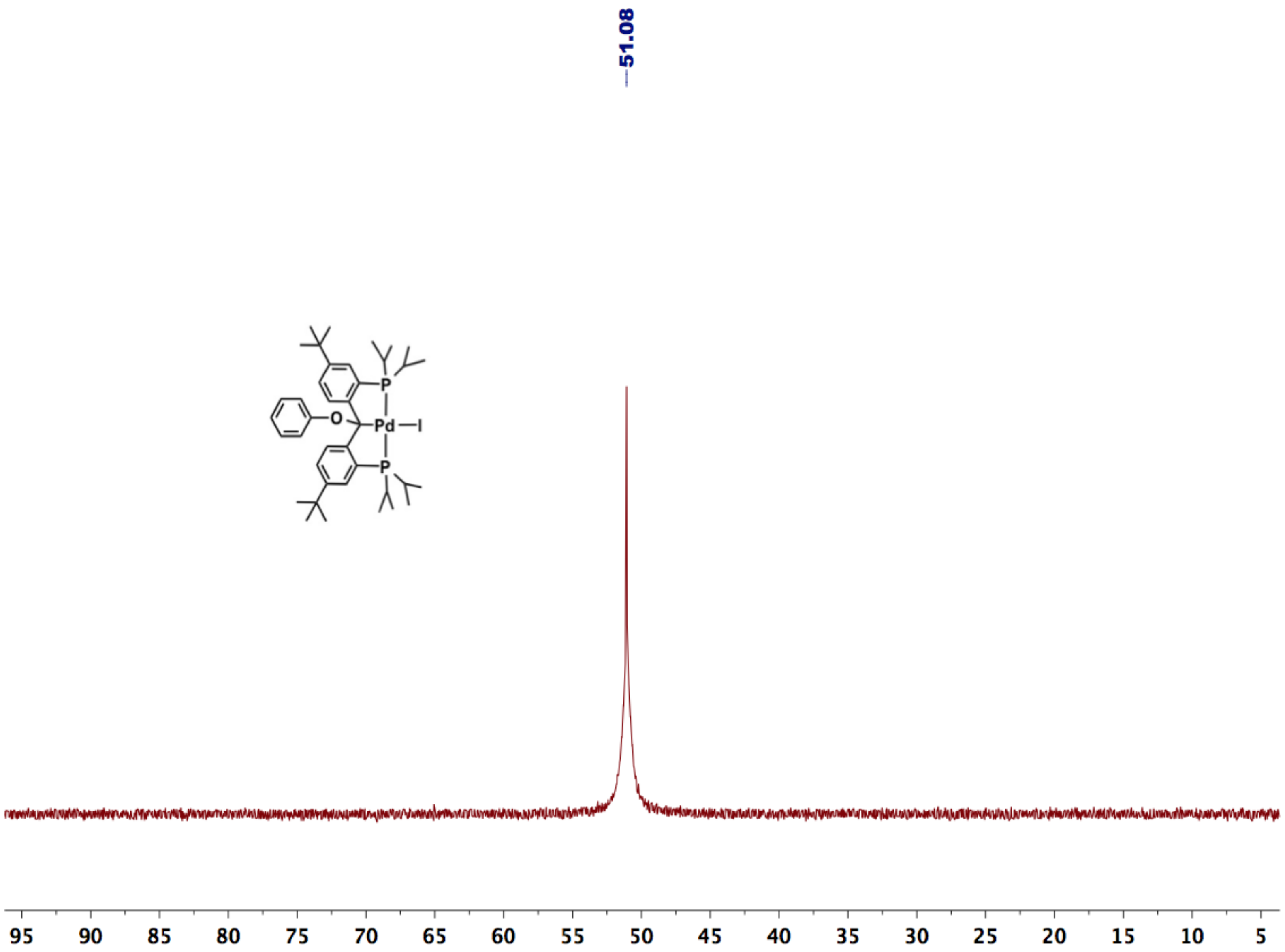

Figure S32. $^{31}P\{^{1}H\}$ NMR spectrum for $[\{PC(sp^3)(OPh)P\}^{tBu}PdI]$ (**11**).

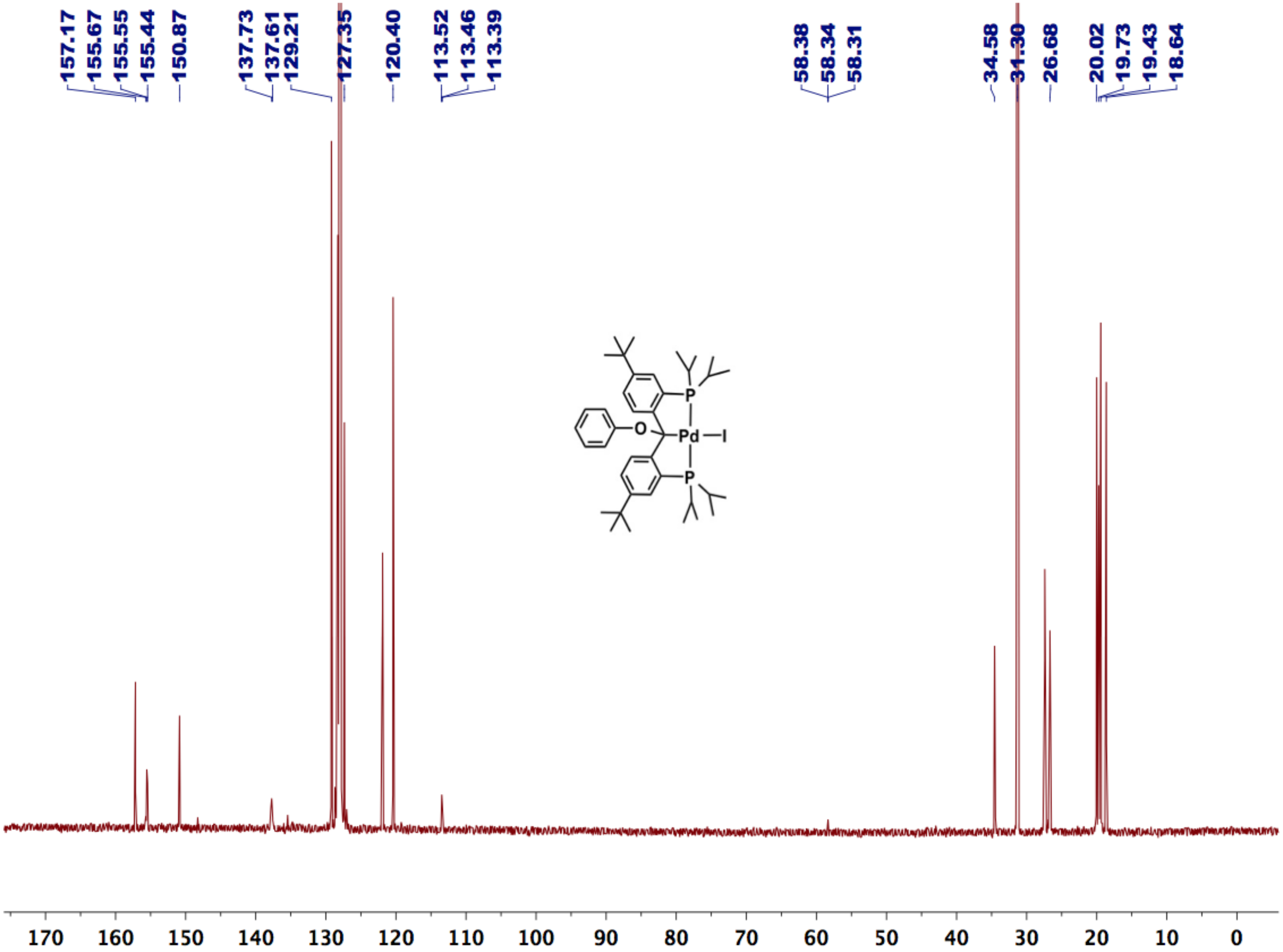

Figure S33. $^{13}C\{^1H\}$ NMR spectrum for [{PC(sp^3)(OPh)P}^{tBu}PdI] (**11**).

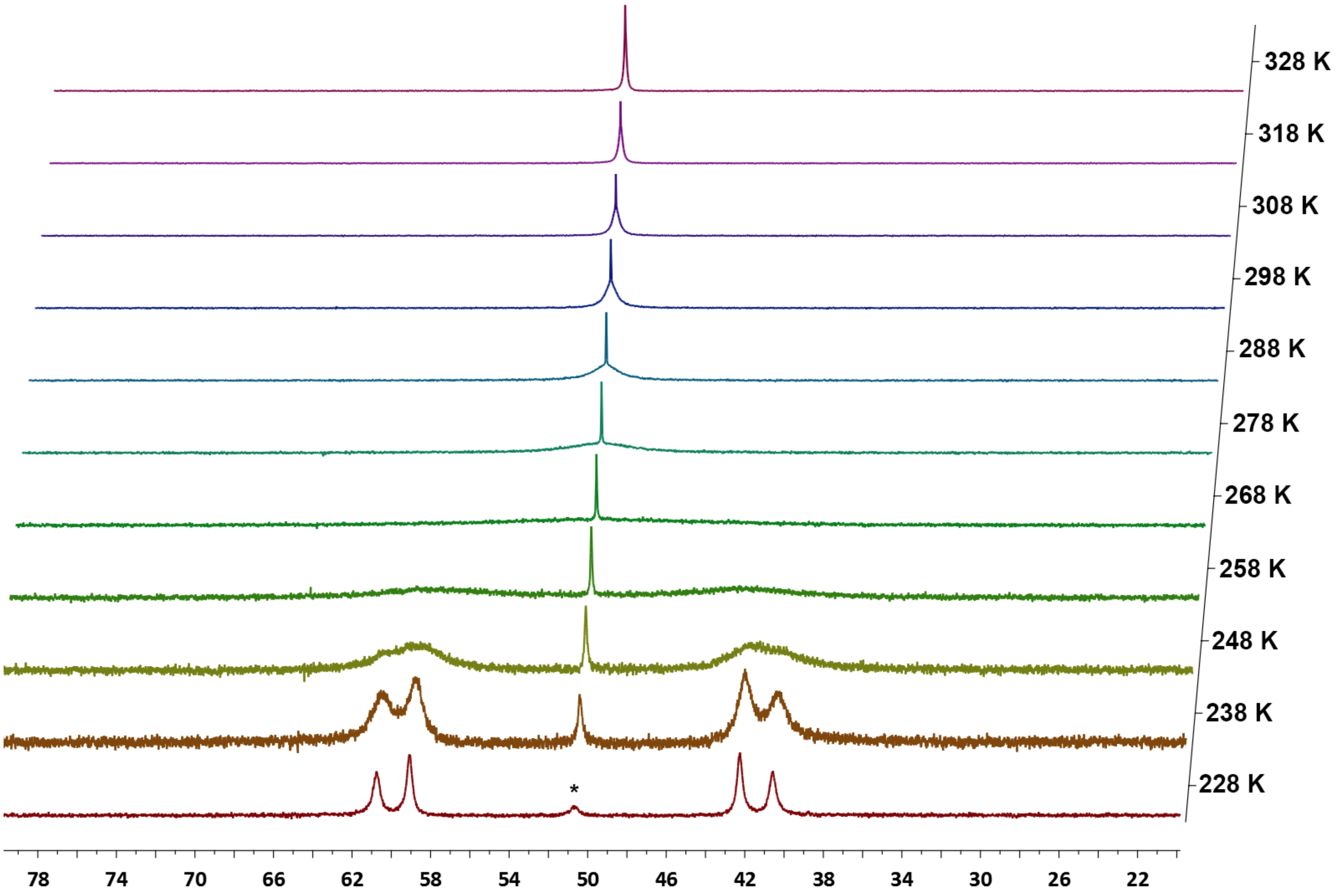

Figure S34. Variable temperature $^{31}P\{^{1}H\}$ NMR spectra for [{PC(sp^3)(OPh)P}^{tBu}PdI] (**11**). (*) designates the small amount of impurity of **6**).

4.8 NMR Spectra for [{PC(sp^3)(PMe_3)P}^{tBu}PdI][BAr^F_4] (12)

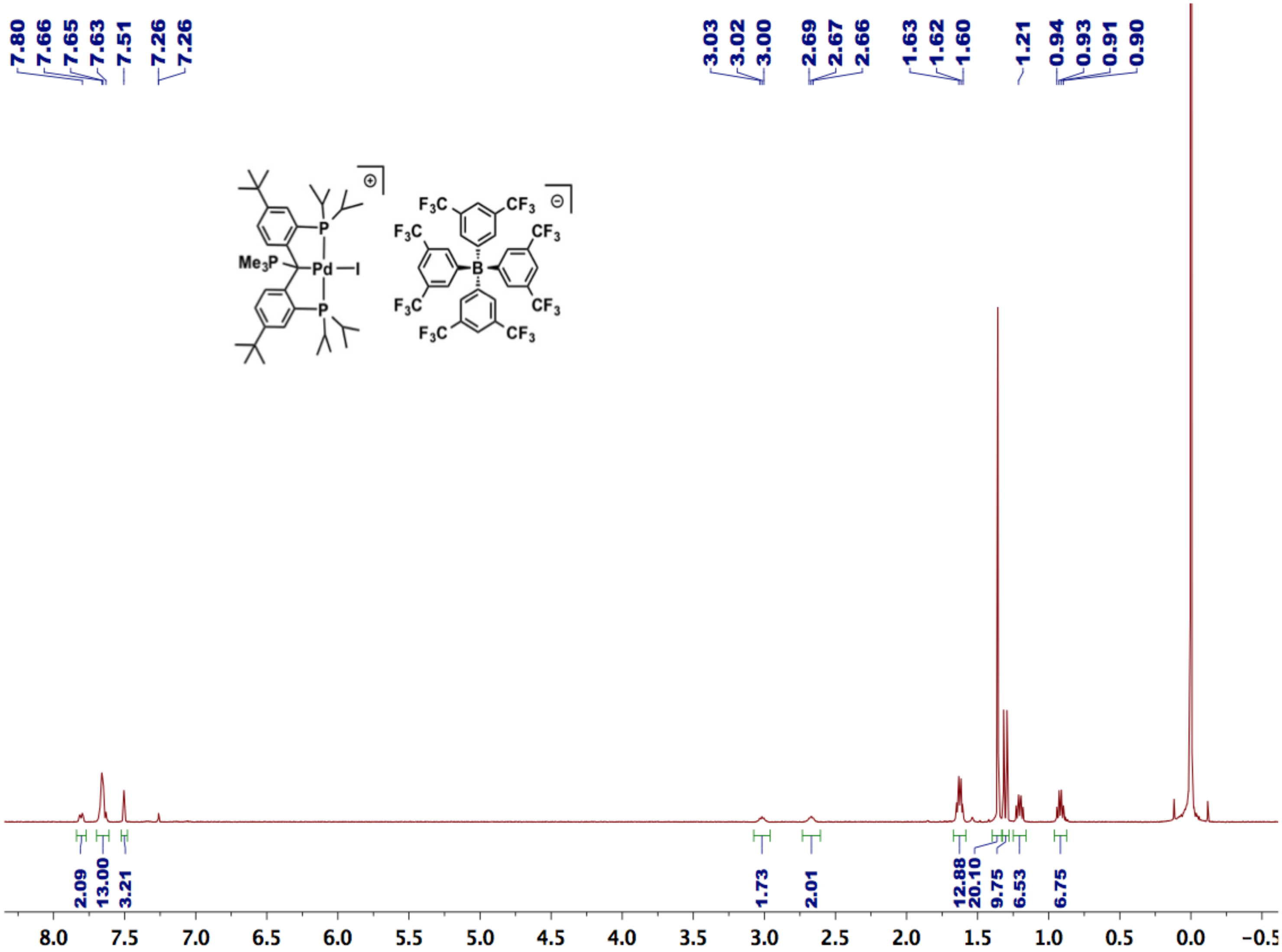

Figure S35. ^{1}H NMR spectrum for [{PC(sp^3)(PMe_3)P}^{tBu}PdI][BAr^F_4] (**12**).

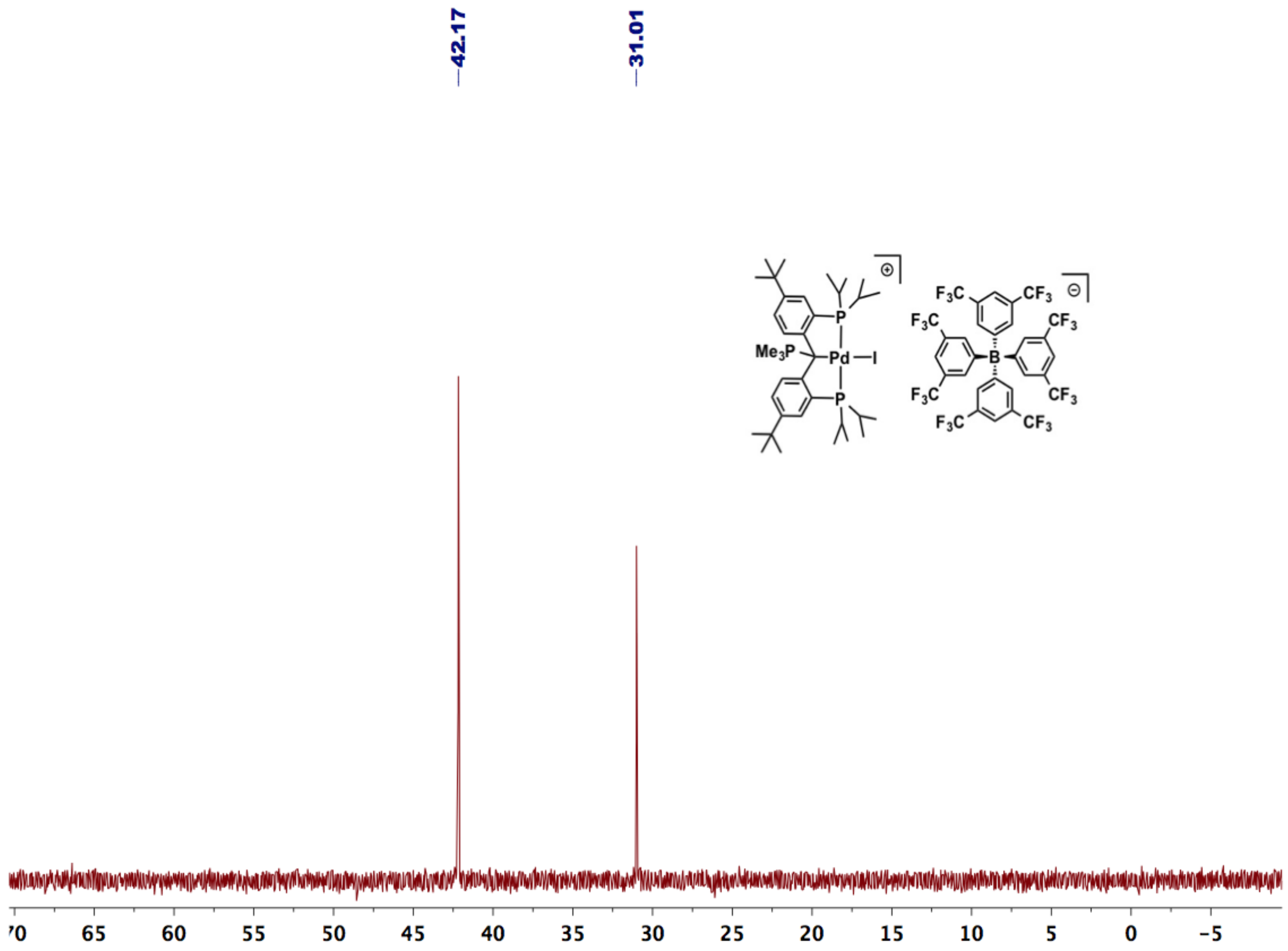

Figure S36. $^{31}P\{^{1}H\}$ NMR spectrum for [{PC(sp^3)(PMe$_3$)P}^{tBu}PdI][BAr$^F_4$] (**12**).

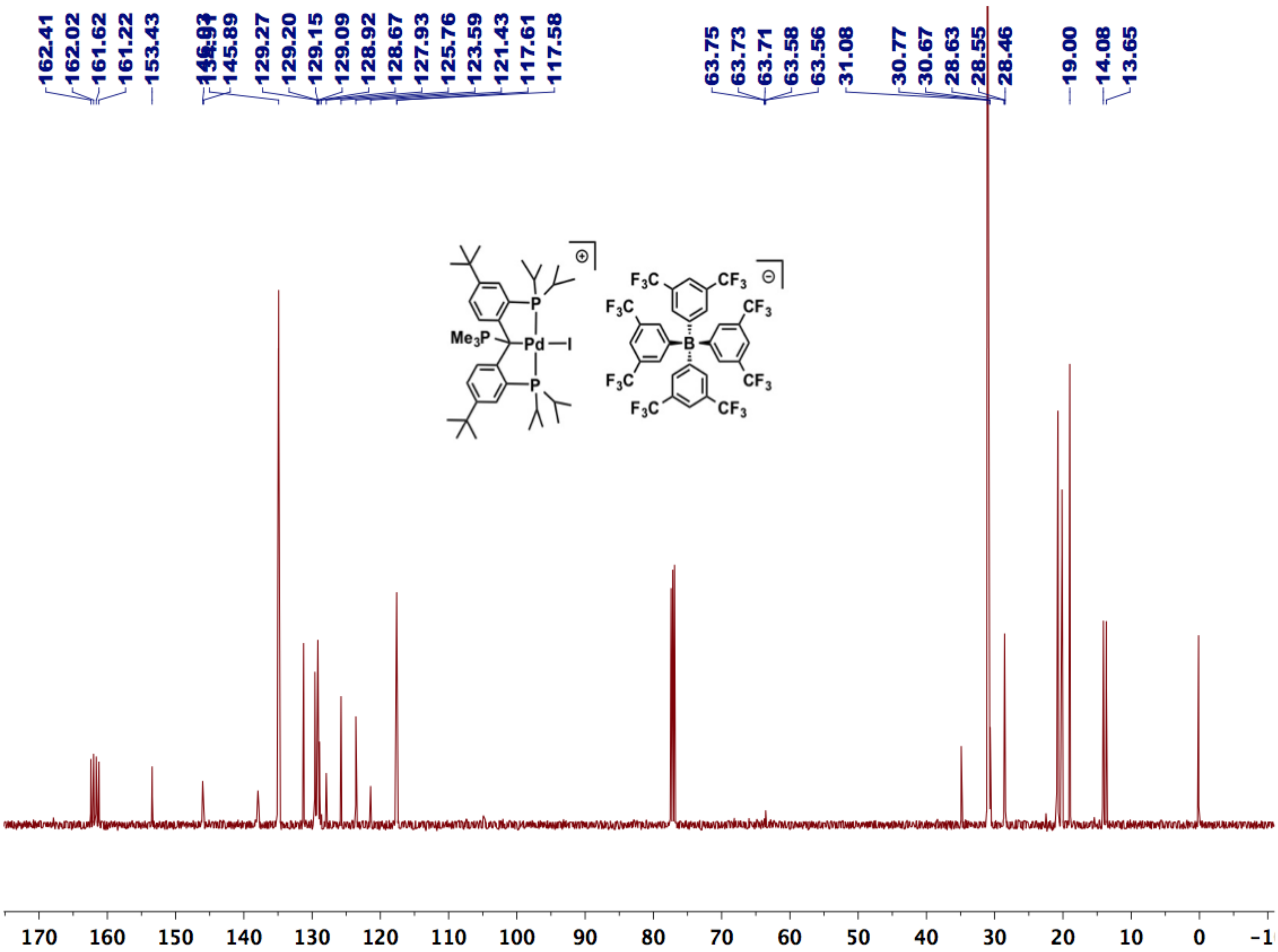

Figure S37. $^{13}C\{^{1}H\}$ NMR spectrum for $[\{PC(sp^3)(PMe_3)P\}^{tBu}PdI][BAr^F_4]$ (**12**).

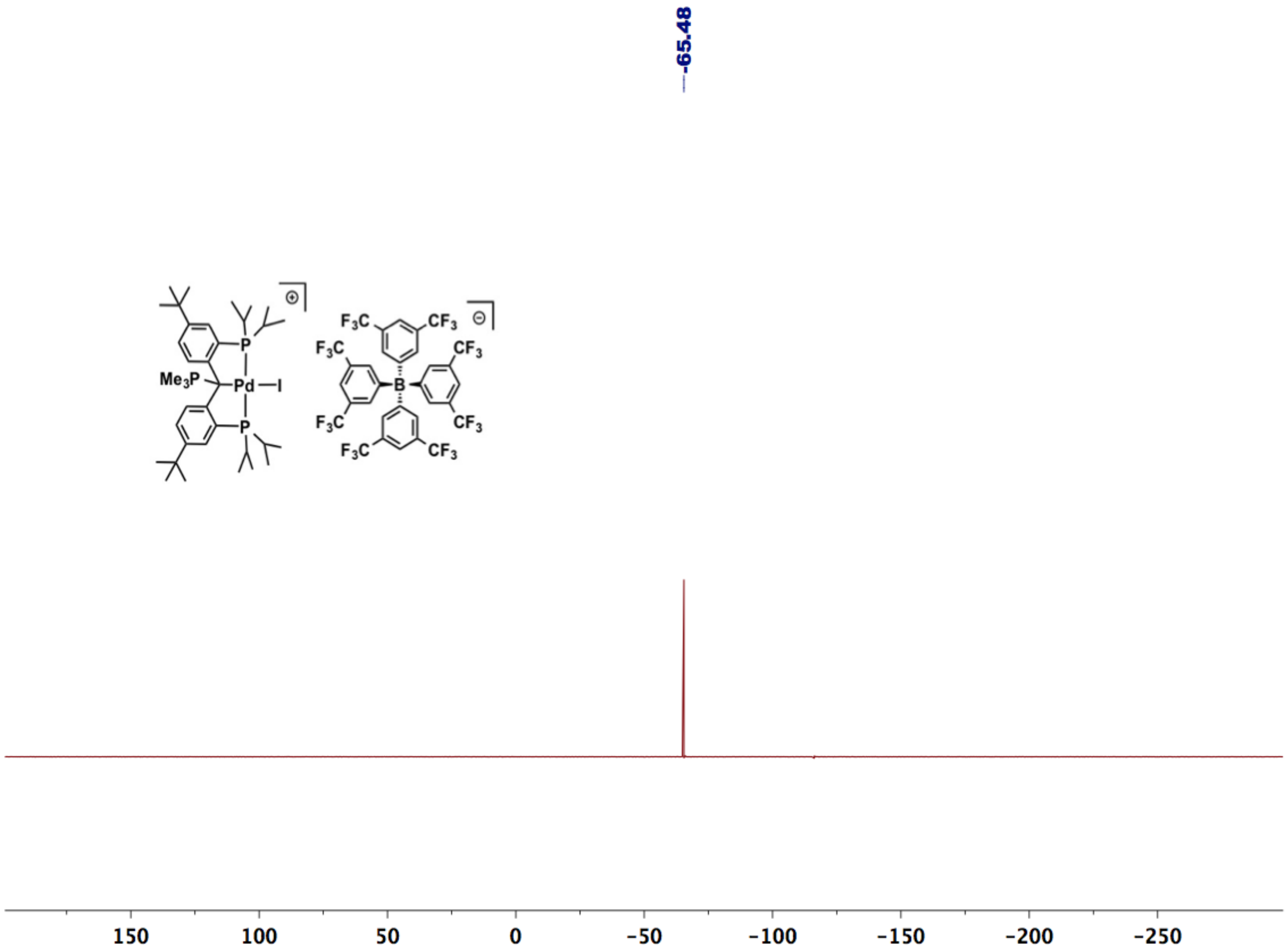

Figure S38. ^{19}F NMR spectrum for [{PC(sp^{3})(PMe$_3$)P}^{tBu}PdI][BAr$^{F}_{4}$] (**12**).

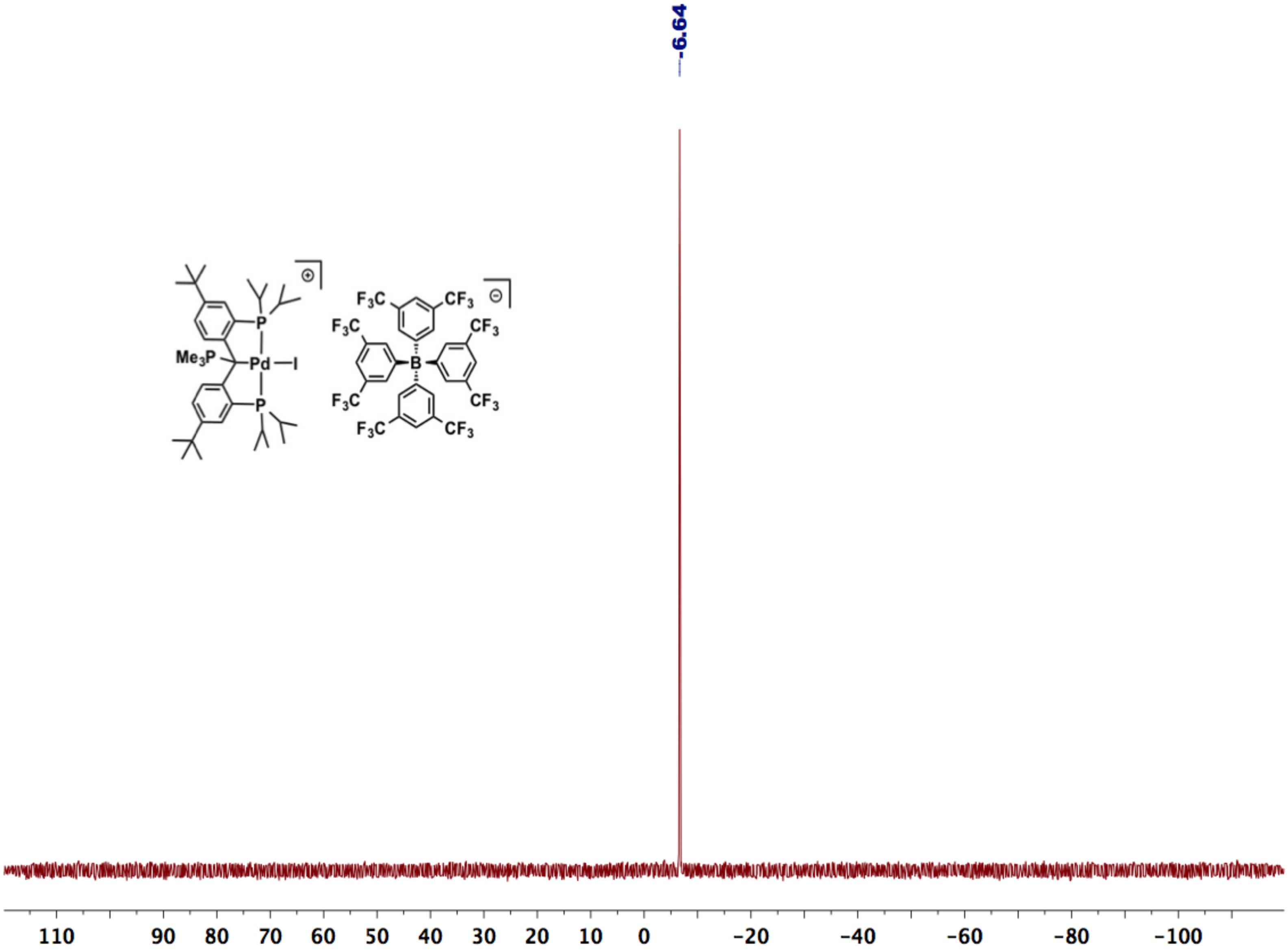

Figure S39. ^{11}B NMR spectrum for [{PC(sp^3)(PMe$_3$)P}^{tBu}PdI][BAr$^F_4$] (**12**).

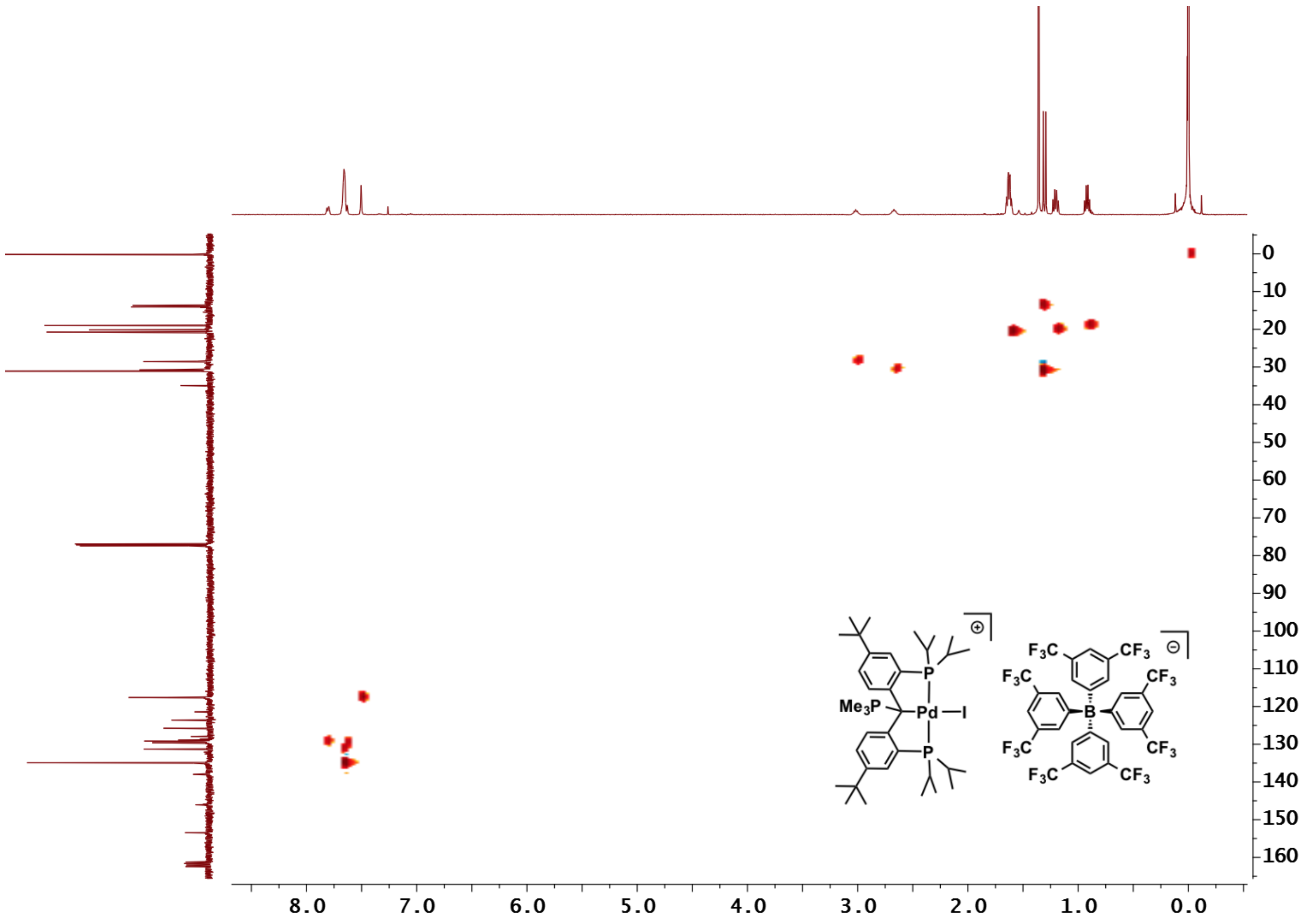

Figure S40. ^{1}H-^{13}C HSQC NMR spectrum for [{PC(sp^3)(PMe$_3$)P}^{tBu}PdI][BAr$^F_4$] (**12**).

4.9 NMR Spectra for the comproportionation reaction between 3 and 5

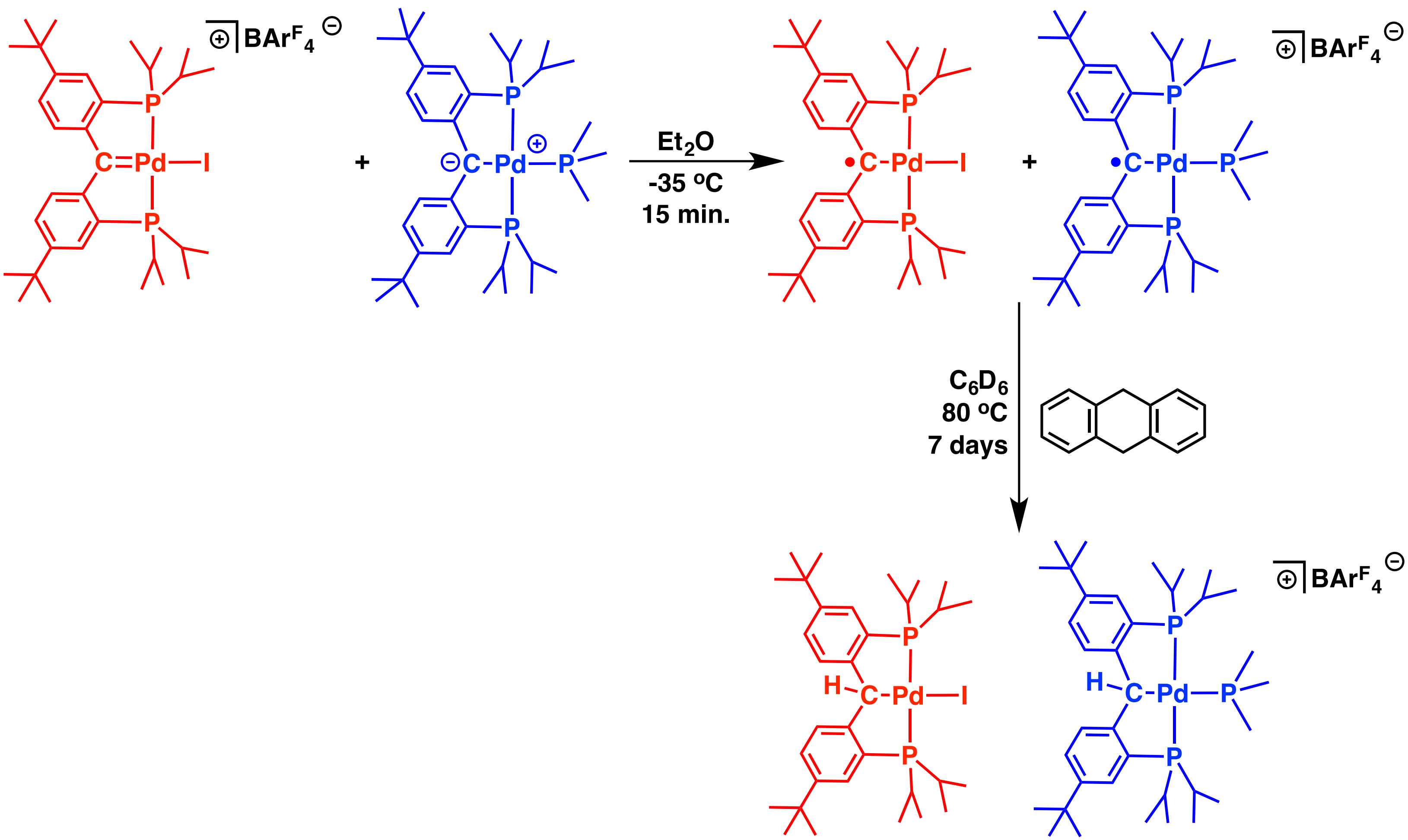

Figure S41. Comproportionation reaction between [{PC(sp^2)P}^{tBu}Pd(PMe$_3$)] (**3**) and [{PC(sp^2)P}^{tBu}PdI][BAr$^F_4$] (**5**).

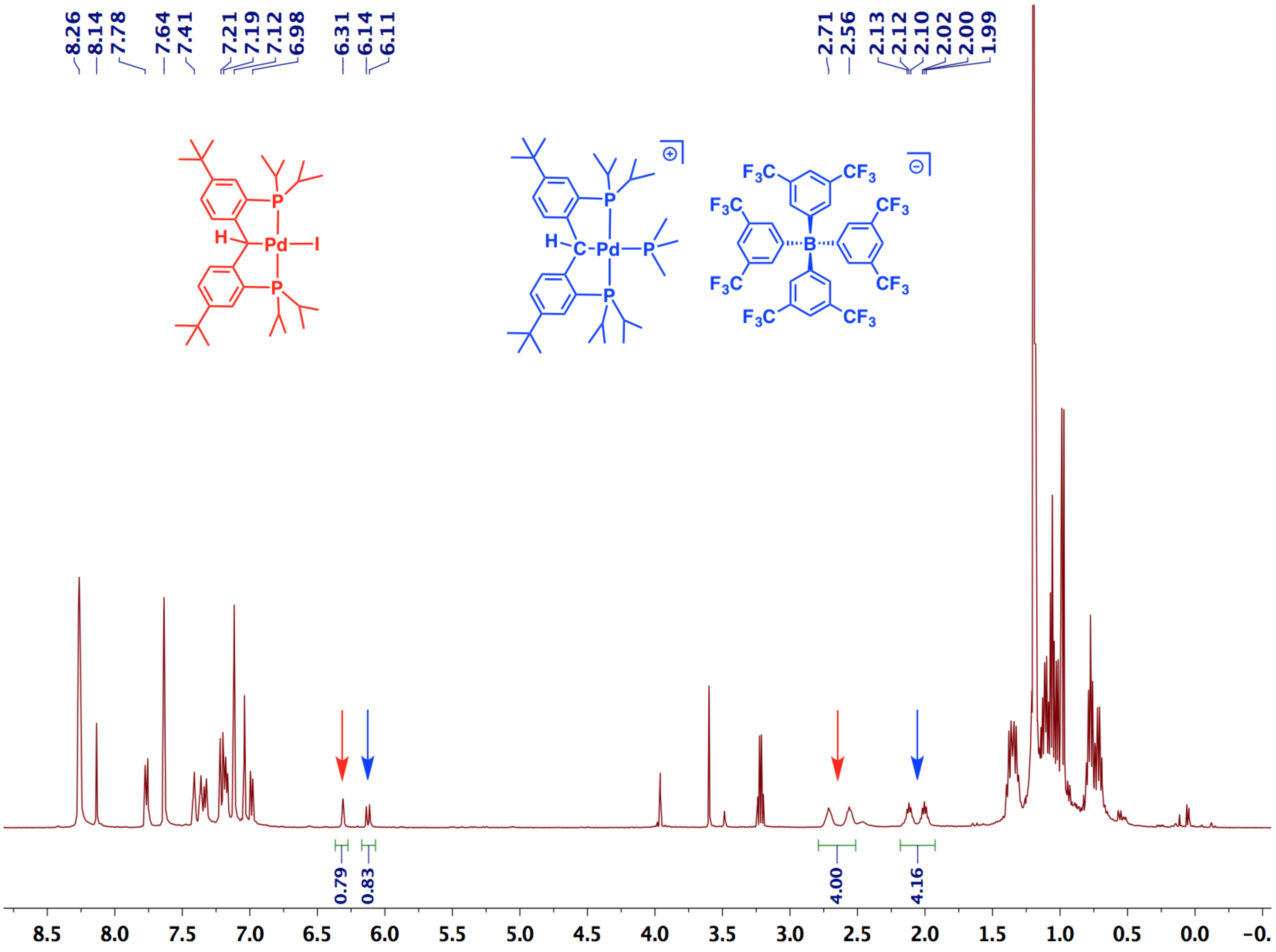

Figure S42. ^{1}H NMR spectrum for the reaction mixture.

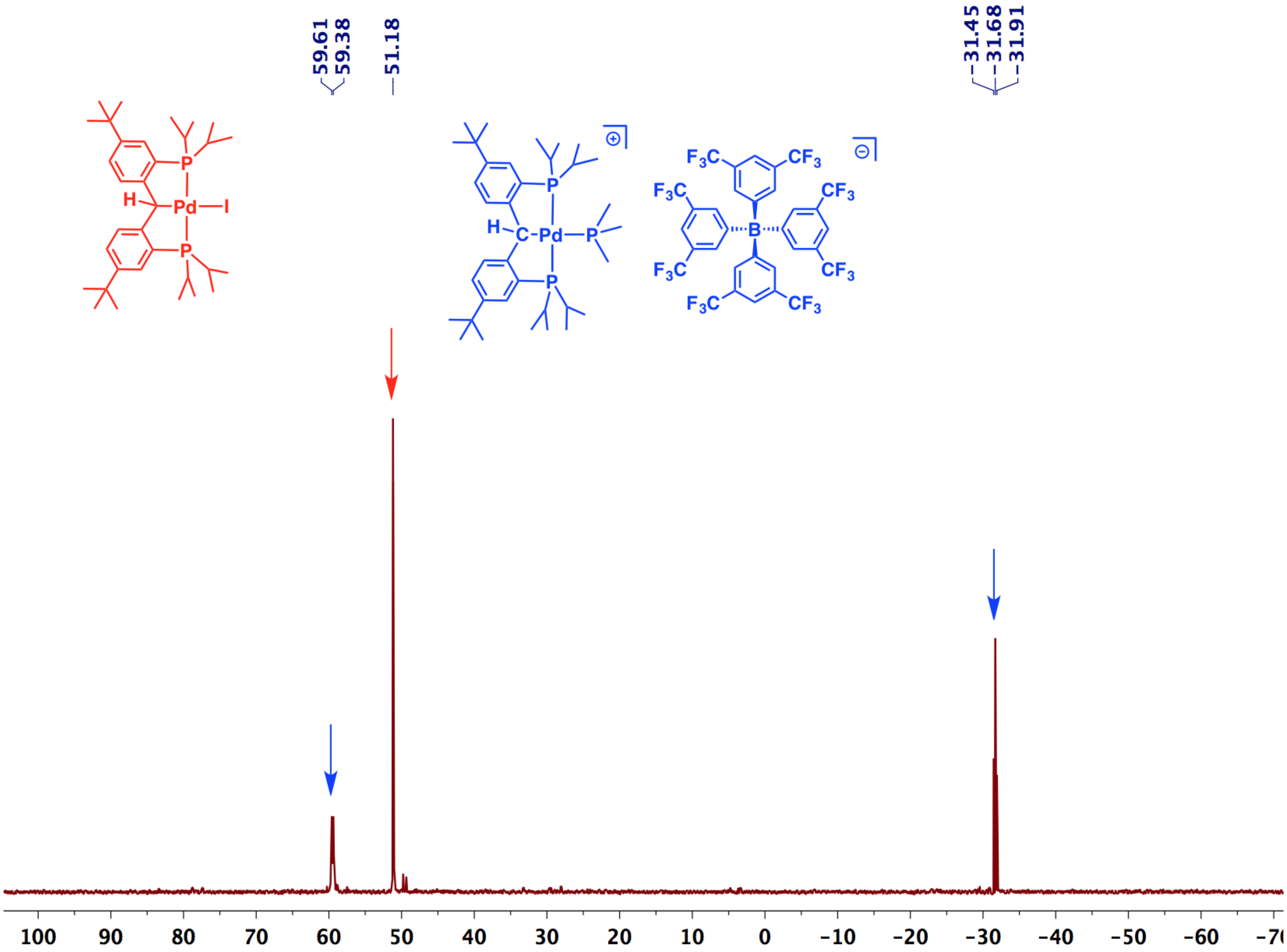

Figure S43. $^{31}P\{^{1}H\}$ NMR spectrum for the reaction mixture.

5 Crystallographic tables

5.1 Crystal data for [{PC•(sp^2)P}^{tBu}PdI] (4)

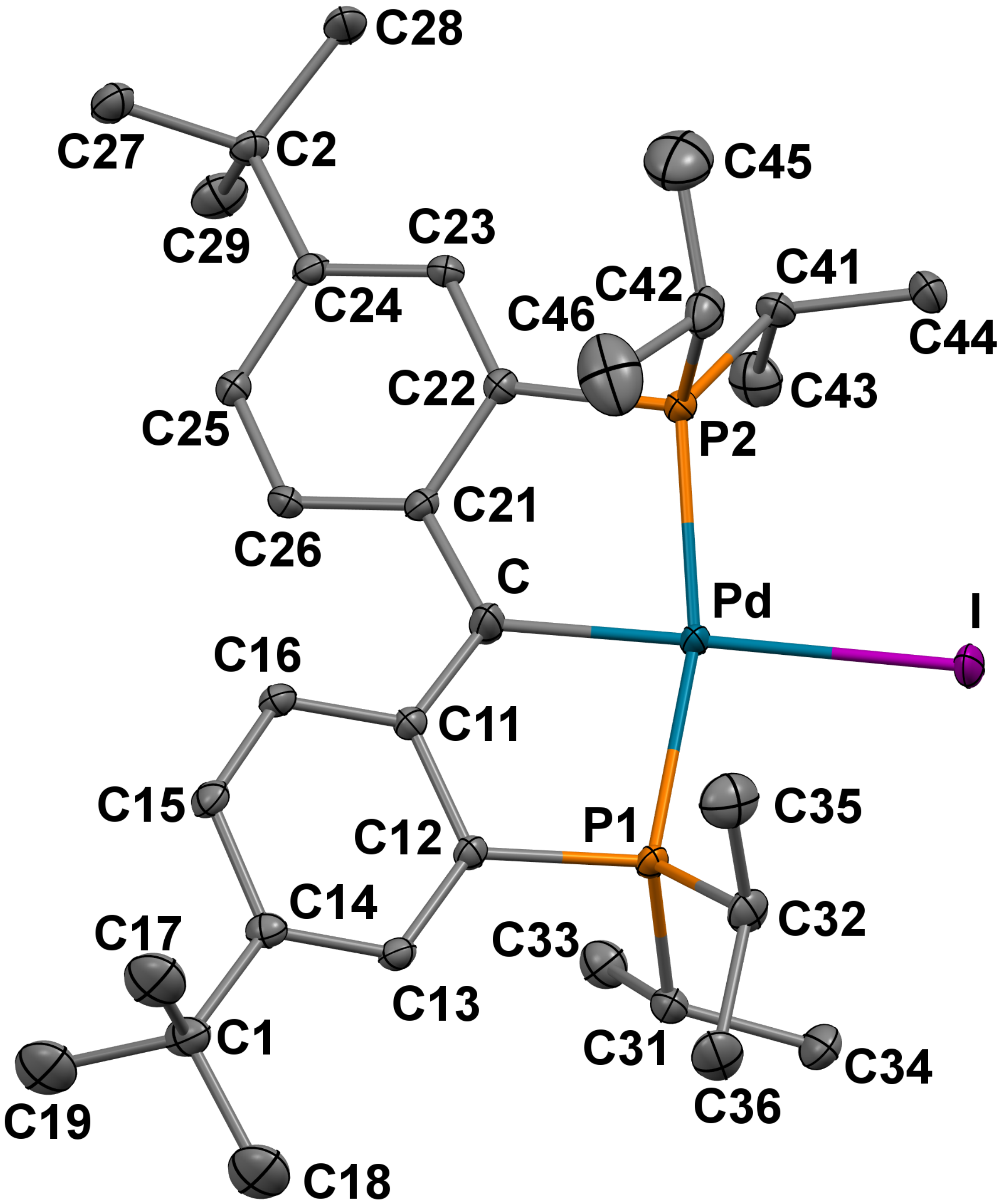

Figure S44. Thermal-ellipsoid representation of [{PC•(sp^2)P}^{tBu}PdI] (**4**) at 50% probability. Hydrogen atoms were omitted for clarity.

Table S6. Crystal data and structure refinement for [{PC•(sp^2)P}^{tBu}PdI] (**4**).

Identification code:	pc27	
Empirical formula:	$C_{33}H_{52}IP_2Pd$	
Formula weight:	743.99	
Temperature:	120(2) K	
Wavelength:	0.71073 Å	
Crystal system:	Orthorhombic	
Space group:	*Pbca*	
Unit cell dimensions:	*a* = 14.978(2) Å	α = 90°
	b = 16.438(3) Å	β = 90°
	c = 29.361(5) Å	γ = 90°
Volume:	7228.9(19) Å^3	
Z:	8	
Density (calculated):	1.367 g·cm^{-3}	
Absorption coefficient (μ):	1.474 mm^{-1}	
F(000):	3032	
Crystal size:	0.09 × 0.08 × 0.07 mm^3	
θ range for data collection:	1.94 to 25.00°	
Index ranges:	−17 ≤ h ≤ 17, −19 ≤ k ≤ 19, −34 ≤ l ≤ 34	
Reflections collected:	141424	
Independent reflections:	6340 [R_{int} = 0.0644]	
Completeness to θ = 25.00°:	99.6 %	
Absorption correction:	Semi-empirical from equivalents	
Max. and min. transmission:	0.8265 and 0.5642	
Refinement method:	Full-matrix least-squares on F^2	
Data / restraints / parameters:	6340 / 0 / 365	
Goodness-of-fit on F^2:	1.079	
Final R indices [I>2σ(I)]:	R_1 = 0.0290, wR_2 = 0.0590	
R indices (all data):	R_1 = 0.0343, wR_2 = 0.0613	
Largest diff. peak and hole:	0.850 and −0.569 e$^-$·Å^{-3}	

Table S7. Atomic coordinates and equivalent isotropic displacement parameters ($Å^2$) for [{PC$^{\bullet}$(sp^2)P}^{tBu}PdI] (**4**). U(eq) is defined as one third of the trace of the orthogonalized U_{ij} tensor

atom	x	y	z	U(eq)
C(1)	0.11287(19)	−0.05689(18)	0.19631(11)	0.031(1)
P(1)	0.13354(4)	0.12092(4)	0.35082(2)	0.019(1)
C(2)	−0.42605(17)	0.24625(17)	0.34417(9)	0.022(1)
C(11)	−0.01718(17)	0.08251(16)	0.30507(9)	0.019(1)
C(12)	0.07690(17)	0.07758(16)	0.30183(9)	0.018(1)
C(13)	0.11786(17)	0.03660(16)	0.26608(9)	0.019(1)
C(14)	0.06934(17)	−0.00712(16)	0.23405(9)	0.021(1)
C(15)	−0.02377(17)	−0.00616(16)	0.23897(9)	0.022(1)
C(16)	−0.06564(17)	0.03778(16)	0.27243(9)	0.021(1)
C(17)	0.0853(4)	−0.1431(3)	0.1999(2)	0.053(1)
C(18)	0.2116(3)	−0.0496(3)	0.19480(18)	0.053(1)
C(19)	0.0781(3)	−0.0234(3)	0.14889(17)	0.053(1)
C(69)	0.0576(10)	−0.1287(8)	0.1797(5)	0.033(2)
C(68)	0.1572(9)	−0.0105(8)	0.1623(4)	0.033(2)
C(67)	0.1978(9)	−0.1081(8)	0.2238(4)	0.033(2)
C(21)	−0.15060(17)	0.15460(17)	0.34044(9)	0.020(1)
C(22)	−0.19448(17)	0.16857(16)	0.38192(9)	0.019(1)
C(23)	−0.28267(17)	0.19512(16)	0.38337(9)	0.020(1)
C(24)	−0.33077(17)	0.21149(16)	0.34389(9)	0.019(1)
C(25)	−0.28633(17)	0.19924(16)	0.30290(9)	0.020(1)
C(26)	−0.19960(17)	0.17122(16)	0.30086(9)	0.021(1)
C(27)	−0.48712(19)	0.1942(2)	0.31416(11)	0.032(1)
I(1)	0.1193(2)	0.1891(7)	0.46881(15)	0.049(1)
C(28)	−0.46523(19)	0.2508(2)	0.39153(10)	0.037(1)
C(29)	−0.4241(2)	0.33273(19)	0.32400(12)	0.037(1)
C(31)	0.20662(18)	0.20190(17)	0.32910(9)	0.024(1)
C(32)	0.20704(19)	0.04128(18)	0.37421(10)	0.028(1)
C(33)	0.1493(2)	0.27262(18)	0.31354(11)	0.032(1)
C(34)	0.2744(2)	0.2305(2)	0.36467(11)	0.034(1)
C(35)	0.1512(2)	−0.0309(2)	0.38978(12)	0.042(1)
C(36)	0.2841(2)	0.0139(2)	0.34324(11)	0.039(1)
C(41)	−0.15972(19)	0.23486(18)	0.47083(9)	0.025(1)
C(42)	−0.1499(2)	0.05715(19)	0.45769(11)	0.032(1)
C(43)	−0.1418(2)	0.31815(19)	0.45010(12)	0.039(1)
C(44)	−0.1186(2)	0.2265(2)	0.51805(10)	0.034(1)
C(45)	−0.2437(3)	0.0512(3)	0.47499(15)	0.063(1)
C(46)	−0.1331(3)	−0.0119(2)	0.42341(14)	0.064(1)
C	−0.05707(19)	0.1291(2)	0.34130(10)	0.032(1)
Pd	0.01629(1)	0.15179(1)	0.39769(1)	0.020(1)
I	0.1176(3)	0.1694(3)	0.47206(16)	0.031(1)
P(2)	−0.12201(4)	0.15570(4)	0.43112(2)	0.019(1)
H(13)	0.1810	0.0386	0.2635	0.023
H(15)	−0.0591	−0.0370	0.2184	0.026
H(16)	−0.1290	0.0381	0.2736	0.025
H(17A)	0.0205	−0.1472	0.1957	0.079
H(17B)	0.1014	−0.1641	0.2300	0.079
H(17C)	0.1157	−0.1751	0.1763	0.079
H(18A)	0.2350	−0.0826	0.1697	0.079
H(18B)	0.2369	−0.0689	0.2236	0.079

Continued on next page

Table S7. – continued from previous page

atom	x	y	x	U(eq)
H(18C)	0.2282	0.0075	0.1901	0.079
H(19A)	0.0932	0.0344	0.1462	0.079
H(19B)	0.0132	−0.0301	0.1471	0.079
H(19C)	0.1065	−0.0538	0.1241	0.079
H(69A)	0.0115	−0.1093	0.1587	0.049
H(69B)	0.0292	−0.1553	0.2058	0.049
H(69C)	0.0964	−0.1676	0.1641	0.049
H(68A)	0.1976	0.0285	0.1768	0.039
H(68B)	0.1129	0.0189	0.1440	0.039
H(68C)	0.1916	−0.0469	0.1425	0.039
H(67A)	0.2291	−0.1431	0.2020	0.049
H(67B)	0.1726	−0.1416	0.2483	0.049
H(67C)	0.2398	−0.0688	0.2368	0.049
H(23)	−0.3108	0.2022	0.4121	0.024
H(25)	−0.3168	0.2106	0.2752	0.024
H(26)	−0.1725	0.1630	0.2720	0.025
H(27A)	−0.4618	0.1903	0.2835	0.047
H(27B)	−0.5464	0.2193	0.3125	0.047
H(27C)	−0.4923	0.1396	0.3273	0.047
H(28A)	−0.5253	0.2743	0.3900	0.055
H(28B)	−0.4272	0.2851	0.4108	0.055
H(28C)	−0.4686	0.1960	0.4046	0.055
H(29A)	−0.4002	0.3307	0.2930	0.056
H(29B)	−0.3860	0.3676	0.3429	0.056
H(29C)	−0.4848	0.3549	0.3234	0.056
H(31)	0.2400	0.1804	0.3022	0.028
H(32)	0.2351	0.0647	0.4022	0.033
H(33A)	0.1869	0.3132	0.2984	0.049
H(33B)	0.1200	0.2974	0.3400	0.049
H(33C)	0.1039	0.2530	0.2922	0.049
H(34A)	0.3154	0.1859	0.3719	0.051
H(34B)	0.2428	0.2474	0.3923	0.051
H(34C)	0.3083	0.2767	0.3525	0.051
H(35A)	0.1210	−0.0550	0.3635	0.063
H(35B)	0.1066	−0.0125	0.4119	0.063
H(35C)	0.1901	−0.0716	0.4040	0.063
H(36A)	0.2603	−0.0186	0.3180	0.058
H(36B)	0.3263	−0.0190	0.3609	0.058
H(36C)	0.3148	0.0618	0.3310	0.058
H(41)	−0.2258	0.2291	0.4742	0.030
H(42)	−0.1087	0.0489	0.4841	0.039
H(43A)	−0.0774	0.3251	0.4455	0.059
H(43B)	−0.1639	0.3605	0.4707	0.059
H(43C)	−0.1725	0.3224	0.4207	0.059
H(44A)	−0.1336	0.1730	0.5307	0.052
H(44B)	−0.1422	0.2692	0.5380	0.052
H(44C)	−0.0536	0.2318	0.5158	0.052
H(45A)	−0.2855	0.0564	0.4495	0.094
H(45B)	−0.2547	0.0949	0.4970	0.094
H(45C)	−0.2524	−0.0016	0.4899	0.094
H(46A)	−0.1757	−0.0075	0.3982	0.096

Continued on next page

Table S7. – continued from previous page

atom	x	y	x	U(eq)
H(46B)	−0.1407	−0.0645	0.4386	0.096
H(46C)	−0.0721	−0.0077	0.4115	0.096

Table S8. Anisotropic displacement parameters ($Å^2$) for [{PC$^{\bullet}$(sp^2)P}^{tBu}PdI] (**4**). The anisotropic displacement factor exponent takes the form: $-2\pi^2[h^2a^{*2}U_{11} + ... + 2hka^*b^*U_{12}]$.

atom	U_{11}	U_{22}	U_{33}	U_{23}	U_{13}	U_{12}
C(1)	0.0233(15)	0.0333(17)	0.0352(17)	−0.0179(14)	0.0056(13)	−0.0040(13)
P(1)	0.0125(3)	0.0275(4)	0.0177(3)	−0.0040(3)	−0.0018(3)	0.0037(3)
C(2)	0.0135(13)	0.0279(15)	0.0234(15)	−0.0051(12)	0.0007(11)	0.0041(11)
C(11)	0.0153(13)	0.0242(14)	0.0159(13)	−0.0001(11)	−0.0003(11)	0.0022(11)
C(12)	0.0145(13)	0.0218(14)	0.0175(13)	−0.0008(11)	−0.0019(11)	0.0027(11)
C(13)	0.0132(13)	0.0217(14)	0.0224(14)	−0.0012(11)	0.0012(11)	0.0000(11)
C(14)	0.0185(14)	0.0189(14)	0.0240(14)	−0.0008(11)	0.0021(11)	−0.0012(11)
C(15)	0.0165(14)	0.0228(14)	0.0261(15)	−0.0043(12)	−0.0024(11)	−0.0032(11)
C(16)	0.0137(13)	0.0270(15)	0.0209(14)	0.0008(12)	0.0009(11)	0.0016(11)
C(17)	0.0452(18)	0.061(2)	0.053(2)	−0.0274(16)	0.0105(15)	0.0088(15)
C(18)	0.0452(18)	0.061(2)	0.053(2)	−0.0274(16)	0.0105(15)	0.0088(15)
C(19)	0.0452(18)	0.061(2)	0.053(2)	−0.0274(16)	0.0105(15)	0.0088(15)
C(69)	0.035(5)	0.031(4)	0.032(5)	−0.021(3)	0.004(3)	0.000(3)
C(68)	0.035(5)	0.031(4)	0.032(5)	−0.021(3)	0.004(3)	0.000(3)
C(67)	0.035(5)	0.031(4)	0.032(5)	−0.021(3)	0.004(3)	0.000(3)
C(21)	0.0124(13)	0.0261(14)	0.0208(14)	−0.0036(12)	−0.0004(11)	−0.0012(11)
C(22)	0.0159(13)	0.0241(15)	0.0170(13)	0.0003(11)	−0.0005(11)	0.0000(11)
C(23)	0.0154(13)	0.0277(15)	0.0169(13)	−0.0018(11)	0.0028(11)	0.0003(11)
C(24)	0.0128(13)	0.0222(14)	0.0218(14)	−0.0021(11)	0.0007(11)	−0.0003(11)
C(25)	0.0163(13)	0.0262(15)	0.0183(14)	−0.0013(11)	−0.0013(11)	0.0005(11)
C(26)	0.0165(13)	0.0290(16)	0.0163(13)	−0.0047(11)	0.0033(11)	−0.0006(11)
C(27)	0.0179(15)	0.0438(19)	0.0329(17)	−0.0095(14)	−0.0009(13)	0.0031(13)
I(1)	0.0179(3)	0.102(2)	0.0263(8)	−0.0262(10)	−0.0040(4)	−0.0008(9)
C(28)	0.0161(15)	0.067(2)	0.0268(17)	−0.0076(16)	0.0005(12)	0.0112(15)
C(29)	0.0252(16)	0.0384(19)	0.048(2)	−0.0011(15)	0.0018(15)	0.0098(14)
C(31)	0.0193(14)	0.0314(16)	0.0201(14)	−0.0046(12)	0.0011(11)	0.0003(12)
C(32)	0.0208(15)	0.0348(17)	0.0272(16)	0.0005(13)	−0.0047(12)	0.0097(13)
C(33)	0.0360(18)	0.0293(17)	0.0319(17)	−0.0049(13)	0.0019(14)	0.0025(14)
C(34)	0.0258(16)	0.044(2)	0.0331(17)	−0.0067(15)	−0.0013(13)	−0.0090(14)
C(35)	0.0387(19)	0.0377(19)	0.050(2)	0.0102(16)	−0.0009(16)	0.0071(15)
C(36)	0.0296(17)	0.049(2)	0.0379(19)	−0.0041(16)	−0.0012(14)	0.0204(15)
C(41)	0.0197(14)	0.0366(17)	0.0198(15)	−0.0067(13)	0.0031(11)	0.0033(12)
C(42)	0.0322(17)	0.0370(18)	0.0279(16)	0.0078(14)	−0.0095(14)	−0.0024(14)
C(43)	0.045(2)	0.0345(18)	0.0387(19)	−0.0090(15)	0.0024(16)	0.0042(15)
C(44)	0.0271(16)	0.058(2)	0.0184(15)	−0.0086(14)	0.0000(13)	0.0020(15)
C(45)	0.058(3)	0.065(3)	0.064(3)	0.016(2)	0.003(2)	−0.015(2)
C(46)	0.100(4)	0.033(2)	0.058(3)	0.0022(19)	−0.009(2)	−0.007(2)
C	0.0184(15)	0.055(2)	0.0218(15)	−0.0105(14)	−0.0038(12)	0.0079(14)
Pd	0.0121(1)	0.0323(1)	0.0142(1)	−0.0038(1)	−0.0013(1)	0.0036(1)
I	0.0210(7)	0.0543(15)	0.0162(6)	−0.0027(9)	−0.0066(4)	0.0070(7)
P(2)	0.0142(3)	0.0283(4)	0.0155(3)	−0.0008(3)	−0.0008(3)	0.0019(3)

Table S9. Distances [Å] for [{PC$^{\bullet}$(sp^2)P}^{tBu}PdI] (**4**).

atom – atom	distance	atom – atom	distance
C(1) – C(68)	1.421(14)	C(1) – C(17)	1.480(6)
C(1) – C(18)	1.485(6)	C(1) – C(69)	1.522(14)
C(1) – C(14)	1.524(4)	C(1) – C(19)	1.585(6)
C(1) – C(67)	1.726(14)	P(1) – C(12)	1.816(3)
P(1) – C(31)	1.838(3)	P(1) – C(32)	1.843(3)
P(1) – Pd	2.2880(7)	C(2) – C(28)	1.511(4)
C(2) – C(27)	1.532(4)	C(2) – C(24)	1.537(4)
C(2) – C(29)	1.540(4)	C(11) – C(16)	1.409(4)
C(11) – C(12)	1.415(4)	C(11) – C	1.441(4)
C(12) – C(13)	1.390(4)	C(13) – C(14)	1.389(4)
C(13) – H(13)	0.9500	C(14) – C(15)	1.402(4)
C(15) – C(16)	1.371(4)	C(15) – H(15)	0.9500
C(16) – H(16)	0.9500	C(17) – H(17A)	0.9800
C(17) – H(17B)	0.9800	C(17) – H(17C)	0.9800
C(18) – H(18A)	0.9800	C(18) – H(18B)	0.9800
C(18) – H(18C)	0.9800	C(19) – H(19A)	0.9800
C(19) – H(19B)	0.9800	C(19) – H(19C)	0.9800
C(69) – H(69A)	0.9800	C(69) – H(69B)	0.9800
C(69) – H(69C)	0.9800	C(68) – H(68A)	0.9800
C(68) – H(68B)	0.9800	C(68) – H(68C)	0.9800
C(67) – H(67A)	0.9800	C(67) – H(67B)	0.9800
C(67) – H(67C)	0.9800	C(21) – C(26)	1.401(4)
C(21) – C(22)	1.403(4)	C(21) – C	1.462(4)
C(22) – C(23)	1.392(4)	C(22) – P(2)	1.819(3)
C(23) – C(24)	1.391(4)	C(23) – H(23)	0.9500
C(24) – C(25)	1.390(4)	C(25) – C(26)	1.380(4)
C(25) – H(25)	0.9500	C(26) – H(26)	0.9500
C(27) – H(27A)	0.9800	C(27) – H(27B)	0.9800
C(27) – H(27C)	0.9800	I(1) – Pd	2.668(3)
C(28) – H(28A)	0.9800	C(28) – H(28B)	0.9800
C(28) – H(28C)	0.9800	C(29) – H(29A)	0.9800
C(29) – H(29B)	0.9800	C(29) – H(29C)	0.9800
C(31) – C(33)	1.516(4)	C(31) – C(34)	1.530(4)
C(31) – H(31)	1.0000	C(32) – C(35)	1.521(4)
C(32) – C(36)	1.537(4)	C(32) – H(32)	1.0000
C(33) – H(33A)	0.9800	C(33) – H(33B)	0.9800
C(33) – H(33C)	0.9800	C(34) – H(34A)	0.9800
C(34) – H(34B)	0.9800	C(34) – H(34C)	0.9800
C(35) – H(35A)	0.9800	C(35) – H(35B)	0.9800
C(35) – H(35C)	0.9800	C(36) – H(36A)	0.9800
C(36) – H(36B)	0.9800	C(36) – H(36C)	0.9800
C(41) – C(43)	1.522(4)	C(41) – C(44)	1.523(4)
C(41) – P(2)	1.836(3)	C(41) – H(41)	1.0000
C(42) – C(45)	1.497(5)	C(42) – C(46)	1.538(5)
C(42) – P(2)	1.846(3)	C(42) – H(42)	1.0000
C(43) – H(43A)	0.9800	C(43) – H(43B)	0.9800
C(43) – H(43C)	0.9800	C(44) – H(44A)	0.9800
C(44) – H(44B)	0.9800	C(44) – H(44C)	0.9800
C(45) – H(45A)	0.9800	C(45) – H(45B)	0.9800
C(45) – H(45C)	0.9800	C(46) – H(46A)	0.9800

Continued on next page

Table S9. – continued from previous page

**atom – atom**	**distance**	**atom – atom**	**distance**
C(46) – H(46B)	0.9800	C(46) – H(46C)	0.9800
C – Pd	2.022(3)	Pd – P(2)	2.2931(8)
Pd – I	2.675(5)		

Table S10. Angles [°] for [{PC$^•$(sp^2)P}^{tBu}PdI] (**4**).

atom – atom – atom	angle	atom – atom – atom	angle
C(68) – C(1) – C(17)	134.0(5)	C(68) – C(1) – C(18)	58.0(6)
C(17) – C(1) – C(18)	110.9(4)	C(68) – C(1) – C(69)	116.5(8)
C(18) – C(1) – C(69)	126.5(6)	C(68) – C(1) – C(14)	115.0(5)
C(17) – C(1) – C(14)	110.1(3)	C(18) – C(1) – C(14)	113.9(3)
C(69) – C(1) – C(14)	114.6(6)	C(68) – C(1) – C(19)	49.4(6)
C(17) – C(1) – C(19)	107.7(4)	C(18) – C(1) – C(19)	105.8(4)
C(69) – C(1) – C(19)	79.0(7)	C(14) – C(1) – C(19)	108.2(3)
C(68) – C(1) – C(67)	104.3(7)	C(17) – C(1) – C(67)	72.8(5)
C(18) – C(1) – C(67)	47.1(5)	C(69) – C(1) – C(67)	99.9(7)
C(14) – C(1) – C(67)	103.7(4)	C(19) – C(1) – C(67)	145.3(5)
C(12) – P(1) – C(31)	106.72(12)	C(12) – P(1) – C(32)	107.19(13)
C(31) – P(1) – C(32)	106.73(13)	C(12) – P(1) – Pd	101.83(9)
C(31) – P(1) – Pd	120.35(9)	C(32) – P(1) – Pd	113.07(10)
C(28) – C(2) – C(27)	109.0(2)	C(28) – C(2) – C(24)	112.6(2)
C(27) – C(2) – C(24)	110.1(2)	C(28) – C(2) – C(29)	108.4(3)
C(27) – C(2) – C(29)	107.8(2)	C(24) – C(2) – C(29)	108.8(2)
C(16) – C(11) – C(12)	115.9(2)	C(16) – C(11) – C	124.5(2)
C(12) – C(11) – C	119.6(2)	C(13) – C(12) – C(11)	121.2(2)
C(13) – C(12) – P(1)	125.6(2)	C(11) – C(12) – P(1)	112.93(19)
C(14) – C(13) – C(12)	122.1(2)	C(14) – C(13) – H(13)	119.0
C(12) – C(13) – H(13)	119.0	C(13) – C(14) – C(15)	116.4(2)
C(13) – C(14) – C(1)	123.1(2)	C(15) – C(14) – C(1)	120.4(2)
C(16) – C(15) – C(14)	122.3(3)	C(16) – C(15) – H(15)	118.8
C(14) – C(15) – H(15)	118.8	C(15) – C(16) – C(11)	121.8(2)
C(15) – C(16) – H(16)	119.1	C(11) – C(16) – H(16)	119.1
C(1) – C(17) – H(17A)	109.5	C(1) – C(17) – H(17B)	109.5
C(1) – C(17) – H(17C)	109.5	C(1) – C(18) – H(18A)	109.5
C(1) – C(18) – H(18B)	109.5	C(1) – C(18) – H(18C)	109.5
C(1) – C(19) – H(19A)	109.5	C(1) – C(19) – H(19B)	109.5
C(1) – C(19) – H(19C)	109.5	C(1) – C(69) – H(69A)	109.5
C(1) – C(69) – H(69B)	109.5	H(69A) – C(69) – H(69B)	109.5
C(1) – C(69) – H(69C)	109.5	H(69A) – C(69) – H(69C)	109.5
H(69B) – C(69) – H(69C)	109.5	C(1) – C(68) – H(68A)	109.5
C(1) – C(68) – H(68B)	109.5	H(68A) – C(68) – H(68B)	109.5
C(1) – C(68) – H(68C)	109.5	H(68A) – C(68) – H(68C)	109.5
H(68B) – C(68) – H(68C)	109.5	C(1) – C(67) – H(67A)	109.5
C(1) – C(67) – H(67B)	109.5	H(67A) – C(67) – H(67B)	109.5
C(1) – C(67) – H(67C)	109.5	H(67A) – C(67) – H(67C)	109.5
H(67B) – C(67) – H(67C)	109.5	C(26) – C(21) – C(22)	116.3(2)
C(26) – C(21) – C	124.9(2)	C(22) – C(21) – C	118.7(2)
C(23) – C(22) – C(21)	121.5(2)	C(23) – C(22) – P(2)	125.3(2)
C(21) – C(22) – P(2)	113.01(19)	C(24) – C(23) – C(22)	121.8(2)
C(24) – C(23) – H(23)	119.1	C(22) – C(23) – H(23)	119.1
C(25) – C(24) – C(23)	116.5(2)	C(25) – C(24) – C(2)	120.2(2)
C(23) – C(24) – C(2)	123.3(2)	C(26) – C(25) – C(24)	122.5(3)
C(26) – C(25) – H(25)	118.8	C(24) – C(25) – H(25)	118.8
C(25) – C(26) – C(21)	121.5(2)	C(25) – C(26) – H(26)	119.3
C(21) – C(26) – H(26)	119.3	C(2) – C(27) – H(27A)	109.5
C(2) – C(27) – H(27B)	109.5	H(27A) – C(27) – H(27B)	109.5
C(2) – C(27) – H(27C)	109.5	H(27A) – C(27) – H(27C)	109.5

Continued on next page

Table S10. – continued from previous page

atom – atom – atom	angle	atom – atom – atom	angle
H(27B) – C(27) – H(27C)	109.5	C(2) – C(28) – H(28A)	109.5
C(2) – C(28) – H(28B)	109.5	H(28A) – C(28) – H(28B)	109.5
C(2) – C(28) – H(28C)	109.5	H(28A) – C(28) – H(28C)	109.5
H(28B) – C(28) – H(28C)	109.5	C(2) – C(29) – H(29A)	109.5
C(2) – C(29) – H(29B)	109.5	H(29A) – C(29) – H(29B)	109.5
C(2) – C(29) – H(29C)	109.5	H(29A) – C(29) – H(29C)	109.5
H(29B) – C(29) – H(29C)	109.5	C(33) – C(31) – C(34)	110.2(2)
C(33) – C(31) – P(1)	108.8(2)	C(34) – C(31) – P(1)	112.4(2)
C(33) – C(31) – H(31)	108.4	C(34) – C(31) – H(31)	108.4
P(1) – C(31) – H(31)	108.4	C(35) – C(32) – C(36)	111.2(3)
C(35) – C(32) – P(1)	109.7(2)	C(36) – C(32) – P(1)	115.9(2)
C(35) – C(32) – H(32)	106.5	C(36) – C(32) – H(32)	106.5
P(1) – C(32) – H(32)	106.5	C(31) – C(33) – H(33A)	109.5
C(31) – C(33) – H(33B)	109.5	H(33A) – C(33) – H(33B)	109.5
C(31) – C(33) – H(33C)	109.5	H(33A) – C(33) – H(33C)	109.5
H(33B) – C(33) – H(33C)	109.5	C(31) – C(34) – H(34A)	109.5
C(31) – C(34) – H(34B)	109.5	H(34A) – C(34) – H(34B)	109.5
C(31) – C(34) – H(34C)	109.5	H(34A) – C(34) – H(34C)	109.5
H(34B) – C(34) – H(34C)	109.5	C(32) – C(35) – H(35A)	109.5
C(32) – C(35) – H(35B)	109.5	H(35A) – C(35) – H(35B)	109.5
C(32) – C(35) – H(35C)	109.5	H(35A) – C(35) – H(35C)	109.5
H(35B) – C(35) – H(35C)	109.5	C(32) – C(36) – H(36A)	109.5
C(32) – C(36) – H(36B)	109.5	H(36A) – C(36) – H(36B)	109.5
C(32) – C(36) – H(36C)	109.5	H(36A) – C(36) – H(36C)	109.5
H(36B) – C(36) – H(36C)	109.5	C(43) – C(41) – C(44)	112.0(3)
C(43) – C(41) – P(2)	109.2(2)	C(44) – C(41) – P(2)	112.9(2)
C(43) – C(41) – H(41)	107.5	C(44) – C(41) – H(41)	107.5
P(2) – C(41) – H(41)	107.5	C(45) – C(42) – C(46)	109.1(3)
C(45) – C(42) – P(2)	114.4(3)	C(46) – C(42) – P(2)	109.5(2)
C(45) – C(42) – H(42)	107.9	C(46) – C(42) – H(42)	107.9
P(2) – C(42) – H(42)	107.9	C(41) – C(43) – H(43A)	109.5
C(41) – C(43) – H(43B)	109.5	H(43A) – C(43) – H(43B)	109.5
C(41) – C(43) – H(43C)	109.5	H(43A) – C(43) – H(43C)	109.5
H(43B) – C(43) – H(43C)	109.5	C(41) – C(44) – H(44A)	109.5
C(41) – C(44) – H(44B)	109.5	H(44A) – C(44) – H(44B)	109.5
C(41) – C(44) – H(44C)	109.5	H(44A) – C(44) – H(44C)	109.5
H(44B) – C(44) – H(44C)	109.5	C(42) – C(45) – H(45A)	109.5
C(42) – C(45) – H(45B)	109.5	H(45A) – C(45) – H(45B)	109.5
C(42) – C(45) – H(45C)	109.5	H(45A) – C(45) – H(45C)	109.5
H(45B) – C(45) – H(45C)	109.5	C(42) – C(46) – H(46A)	109.5
C(42) – C(46) – H(46B)	109.5	H(46A) – C(46) – H(46B)	109.5
C(42) – C(46) – H(46C)	109.5	H(46A) – C(46) – H(46C)	109.5
H(46B) – C(46) – H(46C)	109.5	C(11) – C – C(21)	122.5(2)
C(11) – C – Pd	118.5(2)	C(21) – C – Pd	118.8(2)
C – Pd – P(1)	83.32(8)	C – Pd – P(2)	82.22(9)
P(1) – Pd – P(2)	163.13(3)	C – Pd – I(1)	176.1(2)
P(1) – Pd – I(1)	94.47(7)	P(2) – Pd – I(1)	100.43(8)
C – Pd – I	175.42(13)	P(1) – Pd – I	94.58(11)
P(2) – Pd – I	99.20(11)	I(1) – Pd – I	7.3(2)
C(22) – P(2) – C(41)	103.78(13)	C(22) – P(2) – C(42)	107.62(13)
C(41) – P(2) – C(42)	106.49(14)	C(22) – P(2) – Pd	101.68(9)

Continued on next page

Table S10. – continued from previous page

atom – atom – atom	angle	atom – atom – atom	angle
C(41) – P(2) – Pd	124.71(10)	C(42) – P(2) – Pd	111.14(11)

5.2 Crystal data for [{PC(sp^2)P}^{tBu}PdI][BAr$^F_4$] (**5**)

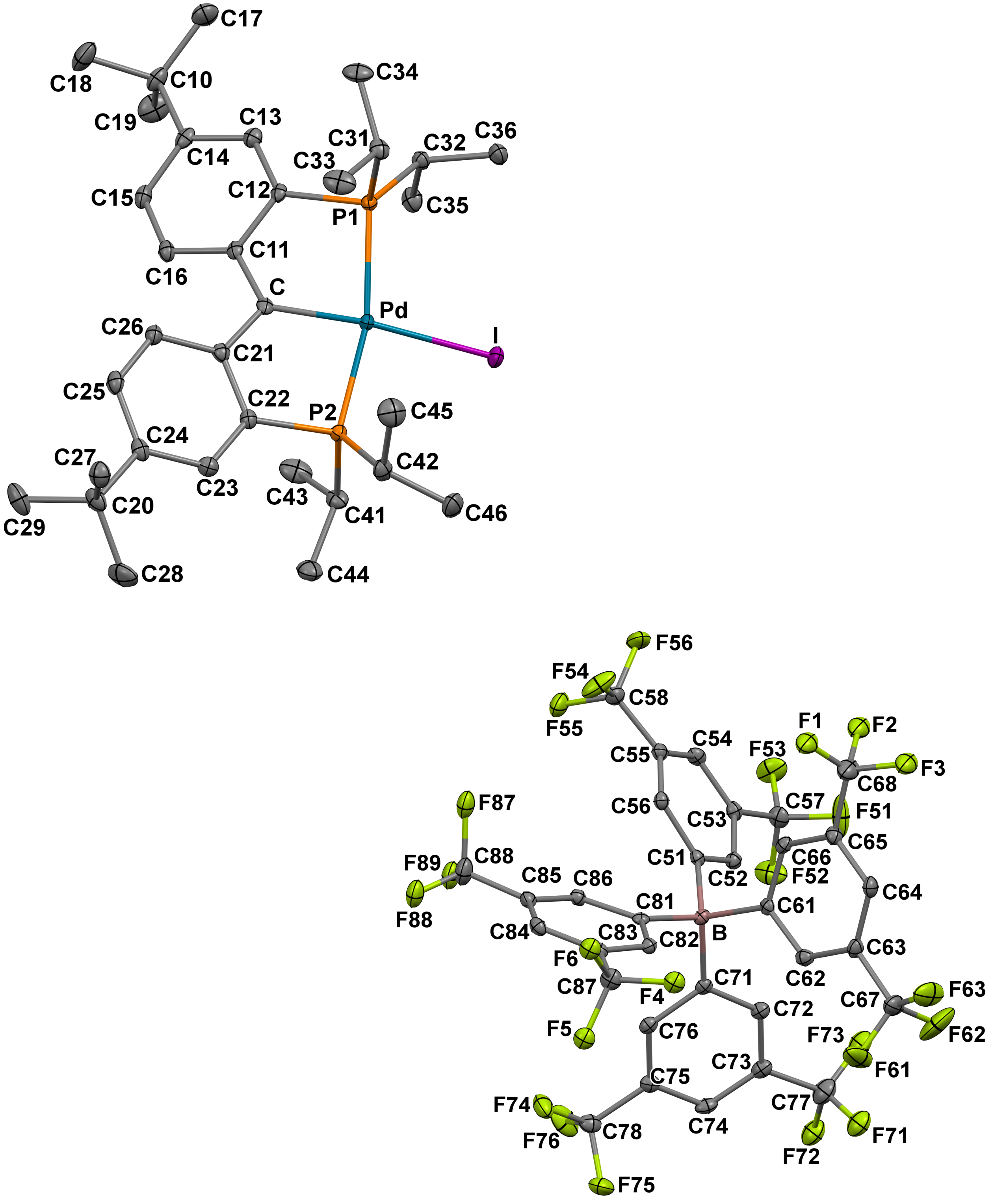

Figure S45. Thermal-ellipsoid representation of [{PC(sp^2)P}^{tBu}PdI][BAr$^F_4$] (**5**) at 50% probability. Hydrogen atoms were omitted for clarity.

Table S11. Crystal data and structure refinement for [{PC(sp^2)P}^{tBu}PdI][BAr$^F_4$] (**5**).

Identification code:	pc28c	
Empirical formula:	$C_{65}H_{64}BF_{24}IP_2Pd$	
Formula weight:	1607.21	
Temperature:	120(2) K	
Wavelength:	0.71073 Å	
Crystal system:	Monoclinic	
Space group:	$P2_1/n$	
Unit cell dimensions:	a = 14.0602(6) Å	α = 90°
	b = 28.6171(12) Å	β = 92.0331(16)°
	c = 16.9031(7) Å	γ = 90°
Volume:	6796.9(5) Å^3	
Z:	4	
Density (calculated):	1.571 g·cm^{-3}	
Absorption coefficient (μ):	0.879 mm^{-1}	
F(000):	3216	
Crystal size:	0.09 × 0.08 × 0.05 mm^3	
θ range for data collection:	1.40 to 25.00°	
Index ranges:	−16 ≤ h ≤ 16, −34 ≤ k ≤ 34, −14 ≤ l ≤ 20	
Reflections collected:	119559	
Independent reflections:	11970 [R_{int} = 0.0438]	
Completeness to θ = 25.00°:	100.0 %	
Absorption correction:	Semi-empirical from equivalents	
Max. and min. transmission:	0.8657 and 0.8133	
Refinement method:	Full-matrix least-squares on F^2	
Data / restraints / parameters:	11970 / 3 / 863	
Goodness-of-fit on F^2:	1.059	
Final R indices [I>2σ(I)]:	R_1 = 0.0320, wR_2 = 0.0725	
R indices (all data):	R_1 = 0.0412, wR_2 = 0.0763	
Largest diff. peak and hole:	1.082 and −0.735 e$^-$·Å^{-3}	

Table S12. Atomic coordinates and equivalent isotropic displacement parameters (Å^2) for [{PC(sp^2)P}^{tBu}PdI][BAr$^F_4$] (**5**). U(eq) is defined as one third of the trace of the orthogonalized U_{ij} tensor

atom	x	y	z	U(eq)
F(86)	−0.1048(4)	0.75394(17)	−0.0477(3)	0.036(1)
F(84)	−0.2358(3)	0.7229(2)	−0.0143(3)	0.036(1)
F(85)	−0.1295(4)	0.68176(19)	−0.0689(3)	0.036(1)
F(89)	−0.1743(6)	0.6766(2)	−0.0497(4)	0.036(1)
F(88)	−0.2045(6)	0.7481(3)	−0.0172(4)	0.036(1)
F(87)	−0.0687(5)	0.7294(3)	−0.0644(3)	0.036(1)
F(93)	−0.0849(7)	0.7019(4)	−0.0704(4)	0.036(1)
F(92)	−0.1598(8)	0.7610(3)	−0.0246(5)	0.036(1)
F(91)	−0.2208(6)	0.6905(3)	−0.0225(4)	0.036(1)
F(72)	−0.1004(3)	0.42844(15)	0.3578(3)	0.039(1)
F(71)	−0.0194(4)	0.47866(13)	0.4270(2)	0.039(1)
F(73)	0.0446(4)	0.44413(16)	0.3310(3)	0.039(1)
F(78)	0.0246(4)	0.4345(2)	0.3190(3)	0.039(1)
F(77)	−0.0980(4)	0.44032(17)	0.3894(3)	0.039(1)
F(79)	0.0272(4)	0.48333(16)	0.4161(3)	0.039(1)
P(1)	0.43254(5)	0.43582(2)	0.34704(4)	0.015(1)
I	0.45934(1)	0.38181(1)	0.55267(1)	0.026(1)
P(2)	0.24496(5)	0.33009(2)	0.47026(4)	0.016(1)
C(11)	0.33757(19)	0.37541(9)	0.24844(16)	0.016(1)
C(10)	0.5072(2)	0.40040(11)	0.04230(17)	0.025(1)
C(12)	0.41046(19)	0.40948(9)	0.25057(16)	0.016(1)
C(14)	0.4471(2)	0.39268(10)	0.11472(16)	0.020(1)
C(13)	0.46169(19)	0.41830(10)	0.18383(16)	0.020(1)
C(15)	0.3787(2)	0.35739(10)	0.11456(16)	0.022(1)
C(16)	0.32412(19)	0.34902(10)	0.17886(16)	0.020(1)
C(21)	0.19279(19)	0.34753(9)	0.31685(16)	0.018(1)
C(20)	−0.0881(2)	0.28365(11)	0.31899(19)	0.026(1)
C(19)	0.5756(3)	0.35883(13)	0.0377(2)	0.041(1)
C(18)	0.4434(3)	0.40311(15)	−0.03279(19)	0.044(1)
C(17)	0.5663(2)	0.44526(12)	0.0482(2)	0.036(1)
C(22)	0.16059(19)	0.32597(9)	0.38607(16)	0.017(1)
C(26)	0.12975(19)	0.34916(10)	0.25022(16)	0.020(1)
C(25)	0.0422(2)	0.32839(10)	0.25225(17)	0.023(1)
C(24)	0.01145(19)	0.30509(10)	0.31908(17)	0.021(1)
C(23)	0.07221(19)	0.30471(10)	0.38631(17)	0.021(1)
C(66)	0.22058(19)	0.63185(9)	0.20521(16)	0.018(1)
C(65)	0.2936(2)	0.64826(10)	0.25474(17)	0.021(1)
C(31)	0.3746(2)	0.49363(10)	0.34450(17)	0.022(1)
C(29)	−0.1116(2)	0.25768(12)	0.2414(2)	0.038(1)
C(28)	−0.1000(2)	0.25007(13)	0.3878(2)	0.043(1)
C(27)	−0.1589(2)	0.32423(11)	0.32646(19)	0.029(1)
C(67)	0.1925(2)	0.62772(13)	0.45347(19)	0.036(1)
F(67)	0.3690(4)	0.6842(3)	0.1474(4)	0.037(1)
F(69)	0.4540(4)	0.6424(2)	0.2272(4)	0.037(1)
F(68)	0.4058(4)	0.7100(2)	0.2634(4)	0.037(1)
F(65)	0.3808(3)	0.71636(17)	0.2286(4)	0.037(1)
F(66)	0.3871(3)	0.6620(2)	0.1424(3)	0.037(1)
F(64)	0.4621(4)	0.6533(2)	0.2560(3)	0.037(1)
F(2)	0.4187(6)	0.6324(4)	0.1679(5)	0.037(1)

Continued on next page

Table S12. – continued from previous page

atom	x	y	x	U(eq)
F(3)	0.4548(7)	0.6718(4)	0.2728(6)	0.037(1)
F(1)	0.3724(6)	0.7037(4)	0.1824(7)	0.037(1)
C(32)	0.56173(19)	0.44602(10)	0.35246(16)	0.019(1)
C(33)	0.2667(2)	0.48824(11)	0.3424(2)	0.036(1)
C(34)	0.4062(2)	0.52450(11)	0.2767(2)	0.033(1)
C(35)	0.6152(2)	0.39965(11)	0.35036(19)	0.027(1)
C(36)	0.5911(2)	0.47483(11)	0.42538(17)	0.025(1)
B	0.0454(2)	0.60198(11)	0.17378(18)	0.016(1)
C(41)	0.2913(2)	0.27148(10)	0.49131(18)	0.024(1)
C(42)	0.1713(2)	0.34836(11)	0.55244(17)	0.024(1)
C(43)	0.3465(2)	0.25450(11)	0.4207(2)	0.038(1)
C(44)	0.2157(2)	0.23622(11)	0.5141(2)	0.033(1)
C(45)	0.1413(3)	0.39911(12)	0.5404(2)	0.038(1)
C(46)	0.2189(2)	0.34095(12)	0.63398(18)	0.034(1)
F(51)	0.24050(17)	0.44774(7)	0.10532(14)	0.057(1)
C(51)	0.08602(18)	0.57470(9)	0.09620(16)	0.016(1)
C(52)	0.10607(19)	0.52708(10)	0.10021(16)	0.019(1)
F(52)	0.09831(15)	0.42709(6)	0.07767(13)	0.047(1)
F(53)	0.19843(15)	0.43079(6)	−0.01352(12)	0.043(1)
C(53)	0.14469(19)	0.50265(10)	0.03824(16)	0.019(1)
F(54)	0.16394(17)	0.64114(7)	−0.11065(11)	0.049(1)
C(54)	0.16168(19)	0.52419(10)	−0.03266(17)	0.021(1)
F(55)	0.07832(14)	0.58839(7)	−0.16554(11)	0.041(1)
C(55)	0.14254(19)	0.57147(10)	−0.03870(16)	0.019(1)
C(57)	0.1703(2)	0.45249(10)	0.05134(18)	0.026(1)
C(56)	0.10747(18)	0.59624(10)	0.02467(16)	0.018(1)
F(56)	0.22710(14)	0.57923(8)	−0.15645(11)	0.045(1)
C(58)	0.1536(2)	0.59502(11)	−0.11670(17)	0.025(1)
C(61)	0.13574(18)	0.61449(9)	0.23421(16)	0.015(1)
C(62)	0.1301(2)	0.61431(9)	0.31637(17)	0.020(1)
C(63)	0.2036(2)	0.63020(10)	0.36643(17)	0.022(1)
F(62)	0.2077(2)	0.58477(10)	0.48182(13)	0.078(1)
F(63)	0.25338(15)	0.65454(9)	0.49435(11)	0.057(1)
F(61)	0.10588(15)	0.63912(11)	0.47494(12)	0.069(1)
C(64)	0.2864(2)	0.64751(10)	0.33606(17)	0.022(1)
C	0.28632(19)	0.36807(9)	0.31980(16)	0.017(1)
Pd	0.35373(1)	0.38105(1)	0.42142(1)	0.015(1)
C(73)	−0.0698(2)	0.50235(9)	0.30162(16)	0.021(1)
C(72)	−0.0044(2)	0.53158(9)	0.26666(16)	0.019(1)
C(71)	−0.03182(19)	0.56843(9)	0.21675(15)	0.016(1)
C(68)	0.3806(2)	0.66843(13)	0.2214(2)	0.037(1)
C(78)	−0.2994(2)	0.55336(11)	0.22561(18)	0.026(1)
C(77)	−0.0341(3)	0.46486(12)	0.3556(2)	0.039(1)
C(76)	−0.12993(19)	0.57447(9)	0.20452(16)	0.018(1)
F(76)	−0.33910(14)	0.52138(8)	0.17673(13)	0.049(1)
C(75)	−0.1954(2)	0.54550(10)	0.23972(16)	0.020(1)
F(75)	−0.34721(13)	0.55060(7)	0.29218(11)	0.040(1)
C(74)	−0.1660(2)	0.50912(10)	0.28824(17)	0.023(1)
F(74)	−0.32043(12)	0.59480(7)	0.19411(12)	0.040(1)
F(81)	−0.0122(8)	0.7534(3)	0.3285(4)	0.026(1)
F(83)	−0.0039(7)	0.8056(3)	0.2308(3)	0.026(1)

Continued on next page

Table S12. – continued from previous page

atom	x	y	x	U(eq)
F(82)	−0.1422(5)	0.7813(4)	0.2761(5)	0.026(1)
F(6)	−0.0311(10)	0.8093(4)	0.2405(7)	0.026(1)
F(5)	−0.1389(8)	0.7703(4)	0.2908(7)	0.026(1)
F(4)	0.0136(9)	0.7589(4)	0.3159(7)	0.026(1)
F(9)	−0.0391(7)	0.7469(3)	0.3328(5)	0.026(1)
F(8)	−0.1346(7)	0.7912(3)	0.2604(6)	0.026(1)
F(7)	0.0094(7)	0.7979(3)	0.2525(7)	0.026(1)
C(81)	−0.00646(18)	0.65221(9)	0.15258(15)	0.014(1)
C(82)	−0.00884(18)	0.68761(9)	0.20958(16)	0.016(1)
C(83)	−0.05384(19)	0.73001(9)	0.19617(16)	0.018(1)
C(87)	−0.0532(2)	0.76645(10)	0.25890(18)	0.026(1)
C(86)	−0.05441(18)	0.66243(10)	0.08085(16)	0.017(1)
C(85)	−0.09696(19)	0.70548(10)	0.06564(17)	0.019(1)
C(84)	−0.09759(19)	0.73977(9)	0.12317(17)	0.019(1)
C(88)	−0.1400(3)	0.71531(12)	−0.0142(2)	0.036(1)
H(13)	0.5080	0.4425	0.1853	0.024
H(15)	0.3695	0.3386	0.0686	0.027
H(16)	0.2770	0.3252	0.1763	0.024
H(19A)	0.6185	0.3636	−0.0060	0.062
H(19B)	0.5391	0.3300	0.0288	0.062
H(19C)	0.6131	0.3563	0.0875	0.062
H(18A)	0.4827	0.4084	−0.0786	0.066
H(18B)	0.3982	0.4289	−0.0281	0.066
H(18C)	0.4084	0.3737	−0.0398	0.066
H(17A)	0.5242	0.4720	0.0566	0.054
H(17B)	0.6001	0.4498	−0.0009	0.054
H(17C)	0.6126	0.4428	0.0928	0.054
H(26)	0.1481	0.3648	0.2036	0.024
H(25)	0.0010	0.3299	0.2065	0.027
H(23)	0.0525	0.2896	0.4330	0.025
H(66)	0.2284	0.6324	0.1496	0.022
H(31)	0.3932	0.5099	0.3950	0.026
H(29A)	−0.0655	0.2324	0.2347	0.056
H(29B)	−0.1083	0.2795	0.1969	0.056
H(29C)	−0.1758	0.2445	0.2430	0.056
H(28A)	−0.0508	0.2258	0.3865	0.064
H(28B)	−0.1630	0.2355	0.3834	0.064
H(28C)	−0.0938	0.2673	0.4378	0.064
H(27A)	−0.2235	0.3117	0.3302	0.044
H(27B)	−0.1559	0.3444	0.2798	0.044
H(27C)	−0.1424	0.3424	0.3741	0.044
H(32)	0.5786	0.4643	0.3046	0.023
H(33A)	0.2458	0.4726	0.2932	0.055
H(33B)	0.2369	0.5192	0.3450	0.055
H(33C)	0.2479	0.4695	0.3878	0.055
H(34A)	0.3893	0.5094	0.2260	0.049
H(34B)	0.4753	0.5290	0.2811	0.049
H(34C)	0.3743	0.5549	0.2794	0.049
H(35A)	0.6838	0.4056	0.3540	0.040
H(35B)	0.5991	0.3834	0.3006	0.040
H(35C)	0.5969	0.3802	0.3951	0.040

Continued on next page

Table S12. – continued from previous page

atom	x	y	x	U(eq)
H(36A)	0.5586	0.5051	0.4231	0.038
H(36B)	0.6601	0.4798	0.4266	0.038
H(36C)	0.5733	0.4581	0.4732	0.038
H(41)	0.3377	0.2742	0.5373	0.028
H(42)	0.1122	0.3289	0.5499	0.029
H(43A)	0.3968	0.2770	0.4094	0.056
H(43B)	0.3031	0.2516	0.3744	0.056
H(43C)	0.3751	0.2240	0.4331	0.056
H(44A)	0.1720	0.2306	0.4686	0.050
H(44B)	0.1800	0.2486	0.5583	0.050
H(44C)	0.2464	0.2068	0.5301	0.050
H(45A)	0.1101	0.4027	0.4879	0.057
H(45B)	0.1976	0.4193	0.5443	0.057
H(45C)	0.0968	0.4080	0.5811	0.057
H(46A)	0.2765	0.3602	0.6389	0.051
H(46B)	0.2359	0.3079	0.6405	0.051
H(46C)	0.1748	0.3500	0.6749	0.051
H(52)	0.0927	0.5107	0.1474	0.022
H(54)	0.1857	0.5072	−0.0759	0.025
H(56)	0.0977	0.6289	0.0192	0.021
H(62)	0.0738	0.6029	0.3390	0.024
H(64)	0.3368	0.6586	0.3700	0.027
H(72)	0.0616	0.5263	0.2771	0.023
H(76)	−0.1524	0.5991	0.1712	0.021
H(74)	−0.2111	0.4892	0.3118	0.027
H(82)	0.0218	0.6823	0.2598	0.019
H(86)	−0.0581	0.6390	0.0410	0.021
H(84)	−0.1271	0.7691	0.1131	0.023

Table S13. Anisotropic displacement parameters (Å^2) for [{PC(sp^2)P}^{tBu}PdI][BAr$^F_4$] (**5**). The anisotropic displacement factor exponent takes the form: $-2\pi^2[h^2a^{*2}U_{11} + ... + 2hka^*b^*U_{12}]$.

atom	U_{11}	U_{22}	U_{33}	U_{23}	U_{13}	U_{12}
F(86)	0.0433(12)	0.0386(11)	0.0260(8)	0.0000(8)	−0.0117(8)	0.0086(9)
F(84)	0.0433(12)	0.0386(11)	0.0260(8)	0.0000(8)	−0.0117(8)	0.0086(9)
F(85)	0.0433(12)	0.0386(11)	0.0260(8)	0.0000(8)	−0.0117(8)	0.0086(9)
F(89)	0.0433(12)	0.0386(11)	0.0260(8)	0.0000(8)	−0.0117(8)	0.0086(9)
F(88)	0.0433(12)	0.0386(11)	0.0260(8)	0.0000(8)	−0.0117(8)	0.0086(9)
F(87)	0.0433(12)	0.0386(11)	0.0260(8)	0.0000(8)	−0.0117(8)	0.0086(9)
F(93)	0.0433(12)	0.0386(11)	0.0260(8)	0.0000(8)	−0.0117(8)	0.0086(9)
F(92)	0.0433(12)	0.0386(11)	0.0260(8)	0.0000(8)	−0.0117(8)	0.0086(9)
F(91)	0.0433(12)	0.0386(11)	0.0260(8)	0.0000(8)	−0.0117(8)	0.0086(9)
F(72)	0.0524(11)	0.0292(9)	0.0349(9)	0.0080(7)	−0.0012(8)	0.0000(7)
F(71)	0.0524(11)	0.0292(9)	0.0349(9)	0.0080(7)	−0.0012(8)	0.0000(7)
F(73)	0.0524(11)	0.0292(9)	0.0349(9)	0.0080(7)	−0.0012(8)	0.0000(7)
F(78)	0.0524(11)	0.0292(9)	0.0349(9)	0.0080(7)	−0.0012(8)	0.0000(7)
F(77)	0.0524(11)	0.0292(9)	0.0349(9)	0.0080(7)	−0.0012(8)	0.0000(7)
F(79)	0.0524(11)	0.0292(9)	0.0349(9)	0.0080(7)	−0.0012(8)	0.0000(7)
P(1)	0.0164(3)	0.0148(3)	0.0148(3)	−0.0006(3)	−0.0009(3)	−0.0005(3)
I	0.0257(1)	0.0332(1)	0.0174(1)	0.0038(1)	−0.0078(1)	−0.0062(1)
P(2)	0.0167(3)	0.0161(3)	0.0153(3)	0.0006(3)	−0.0016(3)	−0.0002(3)
C(11)	0.0169(13)	0.0138(13)	0.0168(14)	0.0004(11)	−0.0048(11)	0.0037(11)
C(10)	0.0269(16)	0.0329(17)	0.0164(15)	0.0004(13)	0.0020(12)	0.0077(13)
C(12)	0.0187(13)	0.0148(13)	0.0150(14)	0.0002(11)	−0.0031(11)	0.0042(11)
C(14)	0.0208(14)	0.0235(15)	0.0168(14)	0.0027(12)	−0.0015(11)	0.0099(12)
C(13)	0.0187(14)	0.0202(14)	0.0205(15)	0.0013(12)	−0.0012(11)	0.0033(11)
C(15)	0.0255(15)	0.0242(15)	0.0163(14)	−0.0045(12)	−0.0042(12)	0.0061(12)
C(16)	0.0194(14)	0.0192(14)	0.0205(15)	−0.0026(12)	−0.0055(11)	0.0023(11)
C(21)	0.0186(14)	0.0156(14)	0.0191(14)	−0.0015(11)	−0.0016(11)	0.0011(11)
C(20)	0.0171(14)	0.0258(16)	0.0357(18)	−0.0026(14)	−0.0043(13)	−0.0020(12)
C(19)	0.038(2)	0.046(2)	0.042(2)	0.0006(17)	0.0151(16)	0.0148(17)
C(18)	0.039(2)	0.072(3)	0.0199(17)	0.0073(17)	0.0003(15)	0.0006(19)
C(17)	0.0376(19)	0.043(2)	0.0278(18)	0.0029(15)	0.0112(15)	−0.0038(16)
C(22)	0.0162(13)	0.0160(14)	0.0184(14)	−0.0027(11)	−0.0023(11)	0.0025(11)
C(26)	0.0215(14)	0.0209(14)	0.0171(14)	−0.0014(12)	−0.0029(11)	0.0011(12)
C(25)	0.0203(14)	0.0248(15)	0.0228(15)	−0.0019(12)	−0.0070(12)	0.0030(12)
C(24)	0.0183(14)	0.0185(14)	0.0262(16)	−0.0059(12)	−0.0035(12)	0.0031(11)
C(23)	0.0195(14)	0.0202(15)	0.0227(15)	−0.0001(12)	0.0022(12)	0.0008(12)
C(66)	0.0228(14)	0.0162(14)	0.0159(14)	0.0023(11)	0.0000(11)	0.0017(11)
C(65)	0.0201(14)	0.0201(15)	0.0226(15)	0.0021(12)	−0.0021(12)	−0.0021(12)
C(31)	0.0241(15)	0.0164(14)	0.0252(16)	−0.0031(12)	−0.0007(12)	0.0027(12)
C(29)	0.0226(16)	0.0366(19)	0.053(2)	−0.0185(17)	−0.0051(15)	−0.0022(14)
C(28)	0.0229(17)	0.043(2)	0.062(3)	0.0144(18)	−0.0070(16)	−0.0132(15)
C(27)	0.0224(16)	0.0356(18)	0.0294(17)	−0.0044(14)	−0.0005(13)	0.0012(13)
C(67)	0.0295(18)	0.058(2)	0.0188(16)	−0.0017(16)	−0.0016(14)	−0.0036(16)
F(67)	0.0293(8)	0.0495(12)	0.0328(11)	0.0077(10)	−0.0028(8)	−0.0135(8)
F(69)	0.0293(8)	0.0495(12)	0.0328(11)	0.0077(10)	−0.0028(8)	−0.0135(8)
F(68)	0.0293(8)	0.0495(12)	0.0328(11)	0.0077(10)	−0.0028(8)	−0.0135(8)
F(65)	0.0293(8)	0.0495(12)	0.0328(11)	0.0077(10)	−0.0028(8)	−0.0135(8)
F(66)	0.0293(8)	0.0495(12)	0.0328(11)	0.0077(10)	−0.0028(8)	−0.0135(8)
F(64)	0.0293(8)	0.0495(12)	0.0328(11)	0.0077(10)	−0.0028(8)	−0.0135(8)
F(2)	0.0293(8)	0.0495(12)	0.0328(11)	0.0077(10)	−0.0028(8)	−0.0135(8)

Continued on next page

Table S13. – continued from previous page

atom	U_{11}	U_{22}	U_{33}	U_{23}	U_{13}	U_{12}
F(3)	0.0293(8)	0.0495(12)	0.0328(11)	0.0077(10)	−0.0028(8)	−0.0135(8)
F(1)	0.0293(8)	0.0495(12)	0.0328(11)	0.0077(10)	−0.0028(8)	−0.0135(8)
C(32)	0.0173(14)	0.0222(15)	0.0185(14)	−0.0008(12)	0.0006(11)	−0.0024(11)
C(33)	0.0259(17)	0.0258(17)	0.058(2)	0.0061(16)	0.0082(16)	0.0110(14)
C(34)	0.0331(18)	0.0206(16)	0.046(2)	0.0107(15)	0.0071(15)	0.0044(13)
C(35)	0.0195(15)	0.0283(16)	0.0321(17)	−0.0023(14)	−0.0042(13)	0.0036(13)
C(36)	0.0230(15)	0.0292(16)	0.0232(16)	−0.0015(13)	−0.0022(12)	−0.0065(13)
B	0.0186(15)	0.0152(15)	0.0138(15)	−0.0001(12)	0.0004(12)	−0.0008(12)
C(41)	0.0214(15)	0.0187(15)	0.0305(17)	0.0043(13)	−0.0038(12)	0.0028(12)
C(42)	0.0239(15)	0.0286(16)	0.0198(15)	−0.0056(13)	0.0045(12)	−0.0023(13)
C(43)	0.0330(18)	0.0224(17)	0.058(2)	0.0003(16)	0.0114(17)	0.0085(14)
C(44)	0.0385(19)	0.0217(16)	0.040(2)	0.0065(14)	−0.0029(15)	−0.0033(14)
C(45)	0.040(2)	0.0335(19)	0.041(2)	−0.0073(16)	0.0094(16)	0.0099(15)
C(46)	0.0391(19)	0.044(2)	0.0191(16)	−0.0026(14)	0.0028(14)	−0.0074(16)
F(51)	0.0675(15)	0.0327(11)	0.0690(15)	−0.0113(11)	−0.0406(12)	0.0251(11)
C(51)	0.0129(13)	0.0178(14)	0.0174(14)	−0.0012(11)	−0.0022(11)	−0.0003(11)
C(52)	0.0182(14)	0.0204(15)	0.0171(14)	0.0001(11)	−0.0021(11)	−0.0006(11)
F(52)	0.0532(13)	0.0200(10)	0.0696(15)	0.0054(10)	0.0240(11)	0.0030(9)
F(53)	0.0666(14)	0.0235(10)	0.0389(11)	−0.0073(8)	0.0166(10)	0.0125(9)
C(53)	0.0175(14)	0.0178(14)	0.0216(15)	−0.0029(12)	−0.0021(11)	0.0009(11)
F(54)	0.0938(17)	0.0288(11)	0.0251(10)	0.0024(9)	0.0109(11)	−0.0133(11)
C(54)	0.0198(14)	0.0238(15)	0.0193(15)	−0.0081(12)	0.0003(12)	0.0018(12)
F(55)	0.0385(11)	0.0590(13)	0.0250(10)	0.0112(9)	−0.0087(8)	−0.0036(10)
C(55)	0.0160(13)	0.0232(15)	0.0173(14)	−0.0015(12)	−0.0006(11)	−0.0008(11)
C(57)	0.0297(16)	0.0199(15)	0.0279(17)	−0.0044(13)	0.0019(13)	0.0058(13)
C(56)	0.0173(13)	0.0170(14)	0.0186(14)	−0.0008(11)	−0.0022(11)	−0.0002(11)
F(56)	0.0425(12)	0.0671(14)	0.0277(10)	0.0136(10)	0.0168(9)	0.0191(10)
C(58)	0.0256(16)	0.0297(17)	0.0191(15)	−0.0028(13)	0.0009(12)	0.0017(13)
C(61)	0.0172(13)	0.0111(13)	0.0175(14)	0.0007(11)	0.0001(11)	0.0036(10)
C(62)	0.0213(14)	0.0186(14)	0.0204(15)	0.0003(12)	0.0033(12)	0.0011(11)
C(63)	0.0239(15)	0.0245(15)	0.0158(14)	−0.0020(12)	−0.0024(12)	0.0044(12)
F(62)	0.129(2)	0.0788(19)	0.0265(12)	0.0219(12)	0.0066(14)	−0.0013(17)
F(63)	0.0458(12)	0.105(2)	0.0201(10)	−0.0165(11)	−0.0031(9)	−0.0233(13)
F(61)	0.0323(12)	0.155(3)	0.0217(11)	−0.0198(14)	0.0043(9)	0.0017(14)
C(64)	0.0216(14)	0.0245(15)	0.0198(15)	−0.0010(12)	−0.0059(12)	0.0007(12)
C	0.0221(14)	0.0092(13)	0.0182(14)	−0.0013(11)	−0.0021(11)	0.0024(11)
Pd	0.0161(1)	0.0153(1)	0.0129(1)	0.0005(1)	−0.0027(1)	−0.0016(1)
C(73)	0.0307(16)	0.0149(14)	0.0178(14)	−0.0018(11)	0.0034(12)	−0.0002(12)
C(72)	0.0232(15)	0.0157(14)	0.0180(14)	−0.0012(11)	0.0023(11)	0.0003(11)
C(71)	0.0200(14)	0.0147(13)	0.0122(13)	−0.0041(11)	0.0008(11)	0.0000(11)
C(68)	0.0293(8)	0.0495(12)	0.0328(11)	0.0077(10)	−0.0028(8)	−0.0135(8)
C(78)	0.0234(15)	0.0274(17)	0.0265(16)	−0.0039(13)	0.0025(13)	−0.0065(13)
C(77)	0.0524(11)	0.0292(9)	0.0349(9)	0.0080(7)	−0.0012(8)	0.0000(7)
C(76)	0.0231(14)	0.0155(14)	0.0151(14)	−0.0023(11)	0.0008(11)	−0.0010(11)
F(76)	0.0305(11)	0.0563(13)	0.0608(14)	−0.0295(11)	−0.0109(10)	−0.0076(9)
C(75)	0.0212(14)	0.0194(14)	0.0189(14)	−0.0053(12)	0.0019(11)	−0.0033(11)
F(75)	0.0253(10)	0.0595(13)	0.0371(11)	0.0045(10)	0.0123(8)	−0.0012(9)
C(74)	0.0294(16)	0.0203(15)	0.0191(15)	−0.0007(12)	0.0061(12)	−0.0086(12)
F(74)	0.0215(9)	0.0417(12)	0.0571(13)	0.0159(10)	−0.0022(9)	−0.0002(8)
F(81)	0.0311(9)	0.0208(8)	0.0259(9)	−0.0043(7)	0.0009(8)	0.0019(7)
F(83)	0.0311(9)	0.0208(8)	0.0259(9)	−0.0043(7)	0.0009(8)	0.0019(7)

Continued on next page

Table S13. – continued from previous page

atom	U_{11}	U_{22}	U_{33}	U_{23}	U_{13}	U_{12}
F(82)	0.0311(9)	0.0208(8)	0.0259(9)	−0.0043(7)	0.0009(8)	0.0019(7)
F(6)	0.0311(9)	0.0208(8)	0.0259(9)	−0.0043(7)	0.0009(8)	0.0019(7)
F(5)	0.0311(9)	0.0208(8)	0.0259(9)	−0.0043(7)	0.0009(8)	0.0019(7)
F(4)	0.0311(9)	0.0208(8)	0.0259(9)	−0.0043(7)	0.0009(8)	0.0019(7)
F(9)	0.0311(9)	0.0208(8)	0.0259(9)	−0.0043(7)	0.0009(8)	0.0019(7)
F(8)	0.0311(9)	0.0208(8)	0.0259(9)	−0.0043(7)	0.0009(8)	0.0019(7)
F(7)	0.0311(9)	0.0208(8)	0.0259(9)	−0.0043(7)	0.0009(8)	0.0019(7)
C(81)	0.0128(12)	0.0135(13)	0.0166(13)	0.0004(11)	0.0029(10)	−0.0045(10)
C(82)	0.0158(13)	0.0164(13)	0.0147(13)	−0.0004(11)	−0.0003(11)	−0.0023(11)
C(83)	0.0172(13)	0.0167(14)	0.0193(14)	−0.0014(11)	0.0013(11)	−0.0015(11)
C(87)	0.0311(9)	0.0208(8)	0.0259(9)	−0.0043(7)	0.0009(8)	0.0019(7)
C(86)	0.0150(13)	0.0191(14)	0.0176(14)	−0.0022(11)	−0.0005(11)	−0.0010(11)
C(85)	0.0146(13)	0.0215(15)	0.0212(15)	0.0010(12)	−0.0011(11)	0.0000(11)
C(84)	0.0161(13)	0.0149(14)	0.0258(15)	−0.0009(12)	0.0003(11)	0.0009(11)
C(88)	0.0433(12)	0.0386(11)	0.0260(8)	0.0000(8)	−0.0117(8)	0.0086(9)

Table S14. Distances [Å] for [{PC(sp^2)P}^{tBu}PdI][BAr$^F_4$] (**5**).

atom – atom	distance	atom – atom	distance
F(86) – C(88)	1.344(6)	F(84) – C(88)	1.364(6)
F(85) – C(88)	1.345(6)	F(89) – C(88)	1.341(7)
F(88) – C(88)	1.305(7)	F(87) – C(88)	1.396(7)
F(93) – C(88)	1.304(9)	F(92) – C(88)	1.347(9)
F(91) – C(88)	1.343(9)	F(72) – C(77)	1.400(5)
F(71) – C(77)	1.281(5)	F(73) – C(77)	1.335(6)
F(78) – C(77)	1.362(7)	F(77) – C(77)	1.290(6)
F(79) – C(77)	1.417(6)	P(1) – C(12)	1.813(3)
P(1) – C(32)	1.839(3)	P(1) – C(31)	1.844(3)
P(1) – Pd	2.3161(7)	I – Pd	2.6255(3)
P(2) – C(22)	1.824(3)	P(2) – C(41)	1.830(3)
P(2) – C(42)	1.838(3)	P(2) – Pd	2.2884(7)
C(11) – C(16)	1.405(4)	C(11) – C(12)	1.414(4)
C(11) – C	1.442(4)	C(10) – C(14)	1.528(4)
C(10) – C(18)	1.530(4)	C(10) – C(17)	1.531(5)
C(10) – C(19)	1.534(4)	C(12) – C(13)	1.383(4)
C(14) – C(13)	1.388(4)	C(14) – C(15)	1.395(4)
C(13) – H(13)	0.9500	C(15) – C(16)	1.373(4)
C(15) – H(15)	0.9500	C(16) – H(16)	0.9500
C(21) – C(26)	1.409(4)	C(21) – C(22)	1.412(4)
C(21) – C	1.440(4)	C(20) – C(28)	1.522(5)
C(20) – C(24)	1.529(4)	C(20) – C(29)	1.533(4)
C(20) – C(27)	1.537(4)	C(19) – H(19A)	0.9800
C(19) – H(19B)	0.9800	C(19) – H(19C)	0.9800
C(18) – H(18A)	0.9800	C(18) – H(18B)	0.9800
C(18) – H(18C)	0.9800	C(17) – H(17A)	0.9800
C(17) – H(17B)	0.9800	C(17) – H(17C)	0.9800
C(22) – C(23)	1.384(4)	C(26) – C(25)	1.369(4)
C(26) – H(26)	0.9500	C(25) – C(24)	1.394(4)
C(25) – H(25)	0.9500	C(24) – C(23)	1.397(4)
C(23) – H(23)	0.9500	C(66) – C(65)	1.384(4)
C(66) – C(61)	1.397(4)	C(66) – H(66)	0.9500
C(65) – C(64)	1.382(4)	C(65) – C(68)	1.482(4)
C(31) – C(33)	1.524(4)	C(31) – C(34)	1.526(4)
C(31) – H(31)	1.0000	C(29) – H(29A)	0.9800
C(29) – H(29B)	0.9800	C(29) – H(29C)	0.9800
C(28) – H(28A)	0.9800	C(28) – H(28B)	0.9800
C(28) – H(28C)	0.9800	C(27) – H(27A)	0.9800
C(27) – H(27B)	0.9800	C(27) – H(27C)	0.9800
C(67) – F(61)	1.323(4)	C(67) – F(63)	1.326(4)
C(67) – F(62)	1.334(4)	C(67) – C(63)	1.487(4)
F(67) – C(68)	1.334(7)	F(69) – C(68)	1.273(8)
F(68) – C(68)	1.424(7)	F(65) – C(68)	1.377(6)
F(66) – C(68)	1.354(6)	F(64) – C(68)	1.339(7)
F(2) – C(68)	1.484(11)	F(3) – C(68)	1.337(11)
F(1) – C(68)	1.209(10)	C(32) – C(35)	1.526(4)
C(32) – C(36)	1.527(4)	C(32) – H(32)	1.0000
C(33) – H(33A)	0.9800	C(33) – H(33B)	0.9800
C(33) – H(33C)	0.9800	C(34) – H(34A)	0.9800
C(34) – H(34B)	0.9800	C(34) – H(34C)	0.9800

Continued on next page

Table S14. – continued from previous page

**atom – atom**	**distance**	**atom – atom**	**distance**
C(35) – H(35A)	0.9800	C(35) – H(35B)	0.9800
C(35) – H(35C)	0.9800	C(36) – H(36A)	0.9800
C(36) – H(36B)	0.9800	C(36) – H(36C)	0.9800
B – C(71)	1.639(4)	B – C(61)	1.641(4)
B – C(51)	1.645(4)	B – C(81)	1.646(4)
C(41) – C(43)	1.525(4)	C(41) – C(44)	1.525(4)
C(41) – H(41)	1.0000	C(42) – C(45)	1.524(4)
C(42) – C(46)	1.525(4)	C(42) – H(42)	1.0000
C(43) – H(43A)	0.9800	C(43) – H(43B)	0.9800
C(43) – H(43C)	0.9800	C(44) – H(44A)	0.9800
C(44) – H(44B)	0.9800	C(44) – H(44C)	0.9800
C(45) – H(45A)	0.9800	C(45) – H(45B)	0.9800
C(45) – H(45C)	0.9800	C(46) – H(46A)	0.9800
C(46) – H(46B)	0.9800	C(46) – H(46C)	0.9800
F(51) – C(57)	1.327(4)	C(51) – C(52)	1.393(4)
C(51) – C(56)	1.400(4)	C(52) – C(53)	1.386(4)
C(52) – H(52)	0.9500	F(52) – C(57)	1.335(4)
F(53) – C(57)	1.332(3)	C(53) – C(54)	1.376(4)
C(53) – C(57)	1.494(4)	F(54) – C(58)	1.331(4)
C(54) – C(55)	1.382(4)	C(54) – H(54)	0.9500
F(55) – C(58)	1.333(3)	C(55) – C(56)	1.389(4)
C(55) – C(58)	1.494(4)	C(56) – H(56)	0.9500
F(56) – C(58)	1.331(3)	C(61) – C(62)	1.394(4)
C(62) – C(63)	1.389(4)	C(62) – H(62)	0.9500
C(63) – C(64)	1.380(4)	C(64) – H(64)	0.9500
C – Pd	1.968(3)	C(73) – C(74)	1.378(4)
C(73) – C(72)	1.390(4)	C(73) – C(77)	1.484(4)
C(72) – C(71)	1.396(4)	C(72) – H(72)	0.9500
C(71) – C(76)	1.398(4)	C(78) – F(74)	1.329(4)
C(78) – F(75)	1.333(3)	C(78) – F(76)	1.341(3)
C(78) – C(75)	1.491(4)	C(76) – C(75)	1.388(4)
C(76) – H(76)	0.9500	C(75) – C(74)	1.379(4)
C(74) – H(74)	0.9500	F(81) – C(87)	1.344(7)
F(83) – C(87)	1.409(7)	F(82) – C(87)	1.363(7)
F(6) – C(87)	1.305(11)	F(5) – C(87)	1.342(12)
F(4) – C(87)	1.340(11)	F(9) – C(87)	1.377(9)
F(8) – C(87)	1.348(10)	F(7) – C(87)	1.266(9)
C(81) – C(86)	1.397(4)	C(81) – C(82)	1.399(4)
C(82) – C(83)	1.383(4)	C(82) – H(82)	0.9500
C(83) – C(84)	1.387(4)	C(83) – C(87)	1.487(4)
C(86) – C(85)	1.389(4)	C(86) – H(86)	0.9500
C(85) – C(84)	1.382(4)	C(85) – C(88)	1.486(4)
C(84) – H(84)	0.9500		

Table S15. Angles [°] for [{PC(sp^2)P}^{tBu}PdI][BAr$^F_4$] (**5**).

atom – atom – atom	angle	atom – atom – atom	angle
C(12) – P(1) – C(32)	104.36(13)	C(12) – P(1) – C(31)	106.90(13)
C(32) – P(1) – C(31)	107.08(13)	C(12) – P(1) – Pd	97.87(9)
C(32) – P(1) – Pd	124.83(9)	C(31) – P(1) – Pd	113.59(10)
C(22) – P(2) – C(41)	108.05(13)	C(22) – P(2) – C(42)	103.87(13)
C(41) – P(2) – C(42)	108.67(14)	C(22) – P(2) – Pd	100.62(9)
C(41) – P(2) – Pd	114.61(10)	C(42) – P(2) – Pd	119.54(10)
C(16) – C(11) – C(12)	117.9(3)	C(16) – C(11) – C	124.4(2)
C(12) – C(11) – C	117.5(2)	C(14) – C(10) – C(18)	110.4(3)
C(14) – C(10) – C(17)	112.5(2)	C(18) – C(10) – C(17)	108.2(3)
C(14) – C(10) – C(19)	107.1(2)	C(18) – C(10) – C(19)	110.4(3)
C(17) – C(10) – C(19)	108.2(3)	C(13) – C(12) – C(11)	120.2(2)
C(13) – C(12) – P(1)	125.5(2)	C(11) – C(12) – P(1)	114.2(2)
C(13) – C(14) – C(15)	117.7(3)	C(13) – C(14) – C(10)	121.9(3)
C(15) – C(14) – C(10)	120.3(3)	C(12) – C(13) – C(14)	121.6(3)
C(12) – C(13) – H(13)	119.2	C(14) – C(13) – H(13)	119.2
C(16) – C(15) – C(14)	121.9(3)	C(16) – C(15) – H(15)	119.0
C(14) – C(15) – H(15)	119.0	C(15) – C(16) – C(11)	120.4(3)
C(15) – C(16) – H(16)	119.8	C(11) – C(16) – H(16)	119.8
C(26) – C(21) – C(22)	117.8(2)	C(26) – C(21) – C	124.2(3)
C(22) – C(21) – C	118.0(2)	C(28) – C(20) – C(24)	112.2(2)
C(28) – C(20) – C(29)	108.8(3)	C(24) – C(20) – C(29)	111.4(3)
C(28) – C(20) – C(27)	109.0(3)	C(24) – C(20) – C(27)	107.0(2)
C(29) – C(20) – C(27)	108.4(2)	C(10) – C(19) – H(19A)	109.5
C(10) – C(19) – H(19B)	109.5	H(19A) – C(19) – H(19B)	109.5
C(10) – C(19) – H(19C)	109.5	H(19A) – C(19) – H(19C)	109.5
H(19B) – C(19) – H(19C)	109.5	C(10) – C(18) – H(18A)	109.5
C(10) – C(18) – H(18B)	109.5	H(18A) – C(18) – H(18B)	109.5
C(10) – C(18) – H(18C)	109.5	H(18A) – C(18) – H(18C)	109.5
H(18B) – C(18) – H(18C)	109.5	C(10) – C(17) – H(17A)	109.5
C(10) – C(17) – H(17B)	109.5	H(17A) – C(17) – H(17B)	109.5
C(10) – C(17) – H(17C)	109.5	H(17A) – C(17) – H(17C)	109.5
H(17B) – C(17) – H(17C)	109.5	C(23) – C(22) – C(21)	120.6(3)
C(23) – C(22) – P(2)	125.8(2)	C(21) – C(22) – P(2)	113.6(2)
C(25) – C(26) – C(21)	120.4(3)	C(25) – C(26) – H(26)	119.8
C(21) – C(26) – H(26)	119.8	C(26) – C(25) – C(24)	122.2(3)
C(26) – C(25) – H(25)	118.9	C(24) – C(25) – H(25)	118.9
C(25) – C(24) – C(23)	117.7(3)	C(25) – C(24) – C(20)	120.1(3)
C(23) – C(24) – C(20)	122.1(3)	C(22) – C(23) – C(24)	121.2(3)
C(22) – C(23) – H(23)	119.4	C(24) – C(23) – H(23)	119.4
C(65) – C(66) – C(61)	122.2(3)	C(65) – C(66) – H(66)	118.9
C(61) – C(66) – H(66)	118.9	C(64) – C(65) – C(66)	121.1(3)
C(64) – C(65) – C(68)	118.4(3)	C(66) – C(65) – C(68)	120.5(3)
C(33) – C(31) – C(34)	110.9(3)	C(33) – C(31) – P(1)	110.4(2)
C(34) – C(31) – P(1)	113.4(2)	C(33) – C(31) – H(31)	107.3
C(34) – C(31) – H(31)	107.3	P(1) – C(31) – H(31)	107.3
C(20) – C(29) – H(29A)	109.5	C(20) – C(29) – H(29B)	109.5
H(29A) – C(29) – H(29B)	109.5	C(20) – C(29) – H(29C)	109.5
H(29A) – C(29) – H(29C)	109.5	H(29B) – C(29) – H(29C)	109.5
C(20) – C(28) – H(28A)	109.5	C(20) – C(28) – H(28B)	109.5
H(28A) – C(28) – H(28B)	109.5	C(20) – C(28) – H(28C)	109.5

Continued on next page

Table S15. – continued from previous page

atom – atom – atom	angle	atom – atom – atom	angle
H(28A) – C(28) – H(28C)	109.5	H(28B) – C(28) – H(28C)	109.5
C(20) – C(27) – H(27A)	109.5	C(20) – C(27) – H(27B)	109.5
H(27A) – C(27) – H(27B)	109.5	C(20) – C(27) – H(27C)	109.5
H(27A) – C(27) – H(27C)	109.5	H(27B) – C(27) – H(27C)	109.5
F(61) – C(67) – F(63)	107.3(3)	F(61) – C(67) – F(62)	105.4(3)
F(63) – C(67) – F(62)	104.7(3)	F(61) – C(67) – C(63)	113.0(3)
F(63) – C(67) – C(63)	113.5(3)	F(62) – C(67) – C(63)	112.2(3)
C(35) – C(32) – C(36)	111.7(2)	C(35) – C(32) – P(1)	110.31(19)
C(36) – C(32) – P(1)	111.33(19)	C(35) – C(32) – H(32)	107.8
C(36) – C(32) – H(32)	107.8	P(1) – C(32) – H(32)	107.8
C(31) – C(33) – H(33A)	109.5	C(31) – C(33) – H(33B)	109.5
H(33A) – C(33) – H(33B)	109.5	C(31) – C(33) – H(33C)	109.5
H(33A) – C(33) – H(33C)	109.5	H(33B) – C(33) – H(33C)	109.5
C(31) – C(34) – H(34A)	109.5	C(31) – C(34) – H(34B)	109.5
H(34A) – C(34) – H(34B)	109.5	C(31) – C(34) – H(34C)	109.5
H(34A) – C(34) – H(34C)	109.5	H(34B) – C(34) – H(34C)	109.5
C(32) – C(35) – H(35A)	109.5	C(32) – C(35) – H(35B)	109.5
H(35A) – C(35) – H(35B)	109.5	C(32) – C(35) – H(35C)	109.5
H(35A) – C(35) – H(35C)	109.5	H(35B) – C(35) – H(35C)	109.5
C(32) – C(36) – H(36A)	109.5	C(32) – C(36) – H(36B)	109.5
H(36A) – C(36) – H(36B)	109.5	C(32) – C(36) – H(36C)	109.5
H(36A) – C(36) – H(36C)	109.5	H(36B) – C(36) – H(36C)	109.5
C(71) – B – C(61)	111.2(2)	C(71) – B – C(51)	109.2(2)
C(61) – B – C(51)	108.5(2)	C(71) – B – C(81)	108.2(2)
C(61) – B – C(81)	105.8(2)	C(51) – B – C(81)	113.9(2)
C(43) – C(41) – C(44)	111.4(3)	C(43) – C(41) – P(2)	109.1(2)
C(44) – C(41) – P(2)	114.1(2)	C(43) – C(41) – H(41)	107.3
C(44) – C(41) – H(41)	107.3	P(2) – C(41) – H(41)	107.3
C(45) – C(42) – C(46)	111.4(3)	C(45) – C(42) – P(2)	109.3(2)
C(46) – C(42) – P(2)	113.7(2)	C(45) – C(42) – H(42)	107.4
C(46) – C(42) – H(42)	107.4	P(2) – C(42) – H(42)	107.4
C(41) – C(43) – H(43A)	109.5	C(41) – C(43) – H(43B)	109.5
H(43A) – C(43) – H(43B)	109.5	C(41) – C(43) – H(43C)	109.5
H(43A) – C(43) – H(43C)	109.5	H(43B) – C(43) – H(43C)	109.5
C(41) – C(44) – H(44A)	109.5	C(41) – C(44) – H(44B)	109.5
H(44A) – C(44) – H(44B)	109.5	C(41) – C(44) – H(44C)	109.5
H(44A) – C(44) – H(44C)	109.5	H(44B) – C(44) – H(44C)	109.5
C(42) – C(45) – H(45A)	109.5	C(42) – C(45) – H(45B)	109.5
H(45A) – C(45) – H(45B)	109.5	C(42) – C(45) – H(45C)	109.5
H(45A) – C(45) – H(45C)	109.5	H(45B) – C(45) – H(45C)	109.5
C(42) – C(46) – H(46A)	109.5	C(42) – C(46) – H(46B)	109.5
H(46A) – C(46) – H(46B)	109.5	C(42) – C(46) – H(46C)	109.5
H(46A) – C(46) – H(46C)	109.5	H(46B) – C(46) – H(46C)	109.5
C(52) – C(51) – C(56)	115.1(2)	C(52) – C(51) – B	120.1(2)
C(56) – C(51) – B	124.8(2)	C(53) – C(52) – C(51)	122.7(3)
C(53) – C(52) – H(52)	118.6	C(51) – C(52) – H(52)	118.6
C(54) – C(53) – C(52)	121.1(3)	C(54) – C(53) – C(57)	120.7(3)
C(52) – C(53) – C(57)	118.1(3)	C(53) – C(54) – C(55)	117.6(3)
C(53) – C(54) – H(54)	121.2	C(55) – C(54) – H(54)	121.2
C(54) – C(55) – C(56)	121.0(3)	C(54) – C(55) – C(58)	118.7(3)
C(56) – C(55) – C(58)	120.1(3)	F(51) – C(57) – F(53)	106.4(2)

Continued on next page

Table S15. – continued from previous page

atom – atom – atom	angle	atom – atom – atom	angle
F(51) – C(57) – F(52)	105.6(3)	F(53) – C(57) – F(52)	105.7(2)
F(51) – C(57) – C(53)	111.7(2)	F(53) – C(57) – C(53)	113.8(3)
F(52) – C(57) – C(53)	113.0(2)	C(55) – C(56) – C(51)	122.3(3)
C(55) – C(56) – H(56)	118.8	C(51) – C(56) – H(56)	118.8
F(54) – C(58) – F(56)	106.9(3)	F(54) – C(58) – F(55)	105.7(2)
F(56) – C(58) – F(55)	104.6(2)	F(54) – C(58) – C(55)	113.2(2)
F(56) – C(58) – C(55)	113.6(2)	F(55) – C(58) – C(55)	112.2(2)
C(62) – C(61) – C(66)	115.5(2)	C(62) – C(61) – B	123.3(2)
C(66) – C(61) – B	120.7(2)	C(63) – C(62) – C(61)	122.6(3)
C(63) – C(62) – H(62)	118.7	C(61) – C(62) – H(62)	118.7
C(64) – C(63) – C(62)	120.7(3)	C(64) – C(63) – C(67)	120.3(3)
C(62) – C(63) – C(67)	119.0(3)	C(63) – C(64) – C(65)	117.9(3)
C(63) – C(64) – H(64)	121.0	C(65) – C(64) – H(64)	121.0
C(21) – C – C(11)	120.9(2)	C(21) – C – Pd	121.2(2)
C(11) – C – Pd	117.59(19)	C – Pd – P(2)	83.52(8)
C – Pd – P(1)	83.03(8)	P(2) – Pd – P(1)	164.98(3)
C – Pd – I	168.44(7)	P(2) – Pd – I	93.767(19)
P(1) – Pd – I	100.717(19)	C(74) – C(73) – C(72)	120.4(3)
C(74) – C(73) – C(77)	120.8(3)	C(72) – C(73) – C(77)	118.8(3)
C(73) – C(72) – C(71)	122.6(3)	C(73) – C(72) – H(72)	118.7
C(71) – C(72) – H(72)	118.7	C(72) – C(71) – C(76)	115.6(2)
C(72) – C(71) – B	122.5(2)	C(76) – C(71) – B	121.9(2)
F(1) – C(68) – F(69)	126.2(6)	F(69) – C(68) – F(67)	110.0(5)
F(1) – C(68) – F(3)	110.6(7)	F(69) – C(68) – F(3)	51.8(5)
F(67) – C(68) – F(3)	130.6(6)	F(1) – C(68) – F(64)	124.9(6)
F(67) – C(68) – F(64)	126.4(4)	F(1) – C(68) – F(66)	65.4(6)
F(69) – C(68) – F(66)	85.0(4)	F(3) – C(68) – F(66)	124.9(6)
F(64) – C(68) – F(66)	107.5(4)	F(69) – C(68) – F(65)	125.2(4)
F(67) – C(68) – F(65)	75.3(4)	F(3) – C(68) – F(65)	82.6(6)
F(64) – C(68) – F(65)	106.5(4)	F(66) – C(68) – F(65)	102.8(4)
F(1) – C(68) – F(68)	65.9(7)	F(69) – C(68) – F(68)	105.3(4)
F(67) – C(68) – F(68)	101.8(5)	F(3) – C(68) – F(68)	56.0(6)
F(64) – C(68) – F(68)	81.8(4)	F(66) – C(68) – F(68)	125.5(4)
F(1) – C(68) – C(65)	118.0(5)	F(69) – C(68) – C(65)	114.9(4)
F(67) – C(68) – C(65)	114.5(4)	F(3) – C(68) – C(65)	114.6(5)
F(64) – C(68) – C(65)	114.3(3)	F(66) – C(68) – C(65)	114.1(3)
F(65) – C(68) – C(65)	110.7(3)	F(68) – C(68) – C(65)	109.2(3)
F(1) – C(68) – F(2)	106.1(7)	F(69) – C(68) – F(2)	48.0(5)
F(67) – C(68) – F(2)	72.5(5)	F(3) – C(68) – F(2)	99.0(7)
F(64) – C(68) – F(2)	73.7(5)	F(65) – C(68) – F(2)	138.3(5)
F(68) – C(68) – F(2)	142.7(5)	C(65) – C(68) – F(2)	106.4(4)
F(74) – C(78) – F(75)	106.2(2)	F(74) – C(78) – F(76)	106.3(3)
F(75) – C(78) – F(76)	105.6(2)	F(74) – C(78) – C(75)	113.7(2)
F(75) – C(78) – C(75)	112.2(3)	F(76) – C(78) – C(75)	112.3(2)
F(71) – C(77) – F(77)	80.9(3)	F(71) – C(77) – F(73)	108.8(4)
F(77) – C(77) – F(73)	119.6(4)	F(71) – C(77) – F(78)	123.0(4)
F(77) – C(77) – F(78)	107.3(4)	F(71) – C(77) – F(72)	106.8(3)
F(73) – C(77) – F(72)	103.8(3)	F(78) – C(77) – F(72)	87.2(3)
F(77) – C(77) – F(79)	107.4(4)	F(73) – C(77) – F(79)	84.3(3)
F(78) – C(77) – F(79)	101.7(4)	F(72) – C(77) – F(79)	130.3(4)
F(71) – C(77) – C(73)	113.4(3)	F(77) – C(77) – C(73)	116.1(4)

Continued on next page

Table S15. – continued from previous page

atom – atom – atom	angle	atom – atom – atom	angle
F(73) – C(77) – C(73)	113.4(3)	F(78) – C(77) – C(73)	112.3(4)
F(72) – C(77) – C(73)	110.1(3)	F(79) – C(77) – C(73)	111.0(3)
C(75) – C(76) – C(71)	121.9(3)	C(75) – C(76) – H(76)	119.1
C(71) – C(76) – H(76)	119.1	C(74) – C(75) – C(76)	121.1(3)
C(74) – C(75) – C(78)	118.7(3)	C(76) – C(75) – C(78)	120.2(3)
C(73) – C(74) – C(75)	118.4(3)	C(73) – C(74) – H(74)	120.8
C(75) – C(74) – H(74)	120.8	C(86) – C(81) – C(82)	115.1(2)
C(86) – C(81) – B	124.6(2)	C(82) – C(81) – B	120.2(2)
C(83) – C(82) – C(81)	123.0(2)	C(83) – C(82) – H(82)	118.5
C(81) – C(82) – H(82)	118.5	C(82) – C(83) – C(84)	120.5(3)
C(82) – C(83) – C(87)	120.5(2)	C(84) – C(83) – C(87)	119.0(2)
F(7) – C(87) – F(4)	72.8(6)	F(6) – C(87) – F(4)	99.0(7)
F(7) – C(87) – F(5)	127.8(6)	F(6) – C(87) – F(5)	104.0(6)
F(4) – C(87) – F(5)	109.9(6)	F(7) – C(87) – F(81)	89.7(4)
F(6) – C(87) – F(81)	111.8(7)	F(5) – C(87) – F(81)	92.0(5)
F(7) – C(87) – F(8)	102.8(5)	F(6) – C(87) – F(8)	73.7(6)
F(4) – C(87) – F(8)	130.3(6)	F(81) – C(87) – F(8)	117.9(5)
F(7) – C(87) – F(82)	116.3(5)	F(6) – C(87) – F(82)	89.2(5)
F(4) – C(87) – F(82)	121.5(5)	F(81) – C(87) – F(82)	105.6(4)
F(7) – C(87) – F(9)	106.7(5)	F(6) – C(87) – F(9)	124.7(7)
F(5) – C(87) – F(9)	76.7(6)	F(8) – C(87) – F(9)	106.9(5)
F(82) – C(87) – F(9)	92.1(5)	F(4) – C(87) – F(83)	91.8(5)
F(5) – C(87) – F(83)	121.9(5)	F(81) – C(87) – F(83)	108.4(4)
F(8) – C(87) – F(83)	90.9(4)	F(82) – C(87) – F(83)	106.9(3)
F(9) – C(87) – F(83)	125.0(4)	F(7) – C(87) – C(83)	115.1(5)
F(6) – C(87) – C(83)	119.0(6)	F(4) – C(87) – C(83)	112.7(5)
F(5) – C(87) – C(83)	111.2(5)	F(81) – C(87) – C(83)	114.9(3)
F(8) – C(87) – C(83)	113.5(4)	F(82) – C(87) – C(83)	112.8(4)
F(9) – C(87) – C(83)	111.1(4)	F(83) – C(87) – C(83)	107.9(3)
C(85) – C(86) – C(81)	122.5(3)	C(85) – C(86) – H(86)	118.7
C(81) – C(86) – H(86)	118.7	C(84) – C(85) – C(86)	120.8(3)
C(84) – C(85) – C(88)	119.5(3)	C(86) – C(85) – C(88)	119.7(3)
C(85) – C(84) – C(83)	118.0(3)	C(85) – C(84) – H(84)	121.0
C(83) – C(84) – H(84)	121.0	F(93) – C(88) – F(88)	127.8(5)
F(93) – C(88) – F(89)	69.2(5)	F(88) – C(88) – F(89)	109.8(5)
F(93) – C(88) – F(91)	106.9(6)	F(88) – C(88) – F(91)	78.0(5)
F(93) – C(88) – F(86)	72.4(5)	F(88) – C(88) – F(86)	70.0(4)
F(89) – C(88) – F(86)	128.5(4)	F(91) – C(88) – F(86)	135.5(4)
F(88) – C(88) – F(85)	125.5(4)	F(91) – C(88) – F(85)	70.4(5)
F(86) – C(88) – F(85)	104.3(4)	F(93) – C(88) – F(92)	108.4(6)
F(89) – C(88) – F(92)	132.3(5)	F(91) – C(88) – F(92)	109.2(6)
F(85) – C(88) – F(92)	129.1(5)	F(93) – C(88) – F(84)	131.1(5)
F(89) – C(88) – F(84)	78.0(4)	F(86) – C(88) – F(84)	104.3(4)
F(85) – C(88) – F(84)	104.2(4)	F(92) – C(88) – F(84)	69.3(5)
F(88) – C(88) – F(87)	106.3(5)	F(89) – C(88) – F(87)	102.7(5)
F(91) – C(88) – F(87)	135.4(5)	F(85) – C(88) – F(87)	71.9(4)
F(92) – C(88) – F(87)	77.8(5)	F(84) – C(88) – F(87)	133.1(4)
F(93) – C(88) – C(85)	111.9(4)	F(88) – C(88) – C(85)	115.6(4)
F(89) – C(88) – C(85)	112.3(4)	F(91) – C(88) – C(85)	108.1(4)
F(86) – C(88) – C(85)	113.2(3)	F(85) – C(88) – C(85)	116.0(3)
F(92) – C(88) – C(85)	112.2(4)	F(84) – C(88) – C(85)	113.7(3)

Continued on next page

Table S15. – continued from previous page

atom – atom – atom	angle	atom – atom – atom	angle
F(87) – C(88) – C(85)	109.2(3)		

5.3 Crystal data for [{PC(sp^3)HP}^{tBu}PdI] (6)

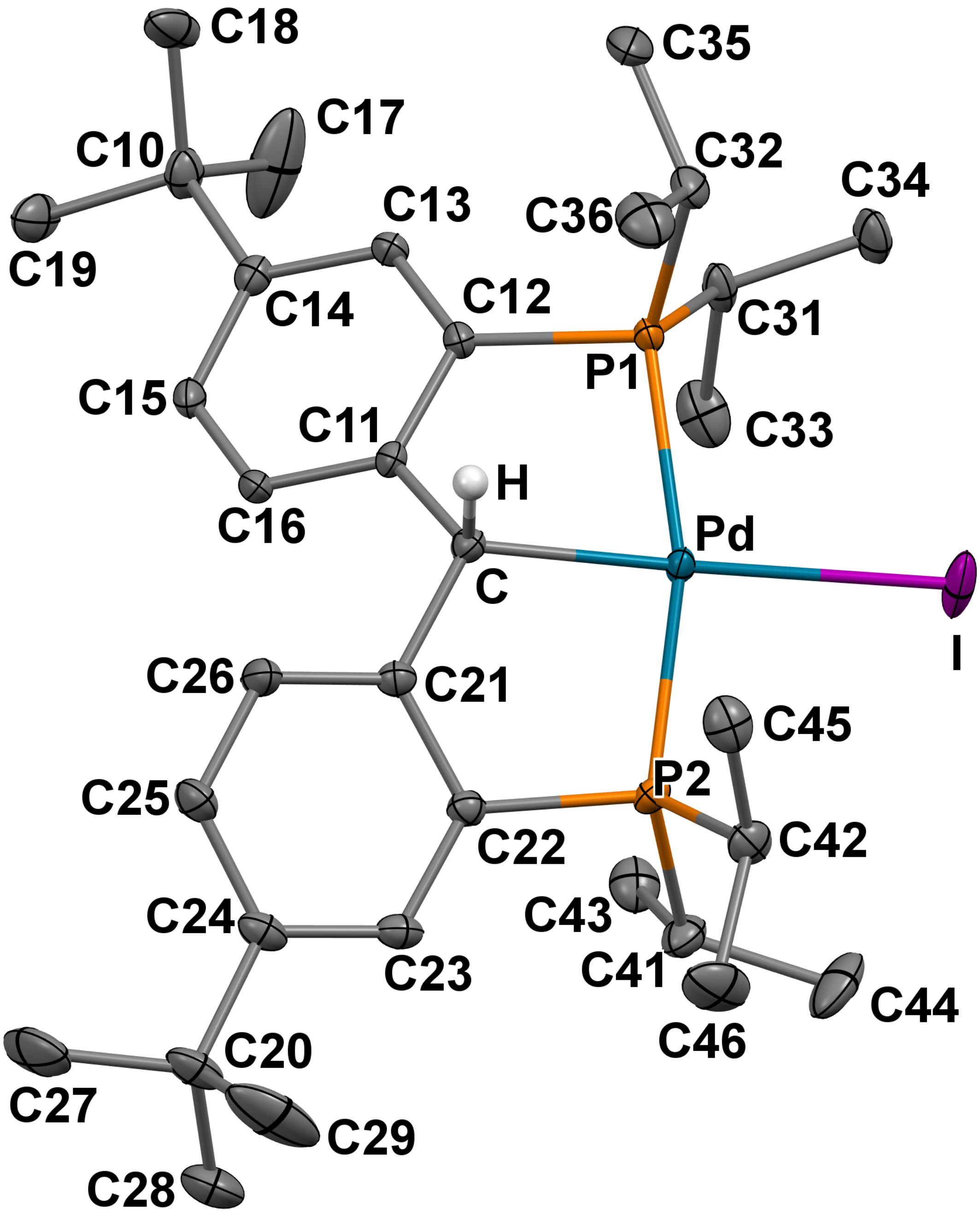

Figure S46. Thermal-ellipsoid representation of [{PC(sp^3)HP}^{tBu}PdI] (**6**) at 50% probability. Most hydrogen atoms were omitted for clarity.

Table S16. Crystal data and structure refinement for [{PC(sp^3)HP}^{tBu}PdI] (**6**).

Identification code:	pc30	
Empirical formula:	$C_{33}H_{53}IP_2Pd$	
Formula weight:	744.99	
Temperature:	120(2) K	
Wavelength:	0.71073 Å	
Crystal system:	Monoclinic	
Space group:	$P2_1/c$	
Unit cell dimensions:	a = 12.8203(13) Å	α = 90°
	b = 14.8340(15) Å	β = 106.6410(15)°
	c = 19.1740(19) Å	γ = 90°
Volume:	3493.7(6) Å^3	
Z:	4	
Density (calculated):	1.416 g·cm^{-3}	
Absorption coefficient (μ):	1.525 mm^{-1}	
F(000):	1520	
Crystal size:	0.12 × 0.11 × 0.09 mm^3	
θ range for data collection:	1.66 to 25.00°	
Index ranges:	$-15 \leq h \leq 15$, $-17 \leq k \leq 17$, $-22 \leq l \leq 22$	
Reflections collected:	63141	
Independent reflections:	6140 [R_{int} = 0.0317]	
Completeness to θ = 25.00°:	99.7 %	
Absorption correction:	Semi-empirical from equivalents	
Max. and min. transmission:	0.8942 and 0.8032	
Refinement method:	Full-matrix least-squares on F^2	
Data / restraints / parameters:	6140 / 0 / 348	
Goodness-of-fit on F^2:	1.168	
Final R indices [I>2σ(I)]:	R_1 = 0.0245, wR_2 = 0.0495	
R indices (all data):	R_1 = 0.0278, wR_2 = 0.0505	
Largest diff. peak and hole:	1.086 and −1.021 e$^-$·Å^{-3}	

Table S17. Atomic coordinates and equivalent isotropic displacement parameters (Å^2) for [{PC(sp^3)HP}^{tBu}PdI] (**6**). U(eq) is defined as one third of the trace of the orthogonalized U_{ij} tensor

atom	x	y	z	U(eq)
P(1)	0.42889(5)	0.14297(4)	0.12336(3)	0.018(1)
P(2)	0.24559(6)	0.38154(4)	−0.00302(4)	0.019(1)
C(10)	0.1998(2)	−0.16310(18)	0.09453(15)	0.028(1)
C(11)	0.2559(2)	0.10051(16)	0.00822(13)	0.018(1)
C(12)	0.32810(19)	0.06351(16)	0.07120(13)	0.018(1)
C(13)	0.3098(2)	−0.02112(17)	0.09627(14)	0.020(1)
C(14)	0.2166(2)	−0.07127(17)	0.06313(14)	0.021(1)
C(15)	0.1426(2)	−0.03196(17)	0.00351(14)	0.023(1)
C(16)	0.1621(2)	0.05099(17)	−0.02391(14)	0.023(1)
I	0.45837(2)	0.39132(1)	0.17099(1)	0.041(1)
Pd	0.35350(2)	0.27546(1)	0.06763(1)	0.017(1)
C(22)	0.1695(2)	0.31944(17)	−0.08287(14)	0.020(1)
C(21)	0.2041(2)	0.23041(17)	−0.08501(13)	0.021(1)
C(20)	−0.0339(2)	0.3547(2)	−0.27156(16)	0.035(1)
C(19)	0.0939(2)	−0.20840(19)	0.05210(18)	0.036(1)
C(18)	0.2928(2)	−0.2257(2)	0.0904(2)	0.048(1)
C(17)	0.1999(5)	−0.1509(3)	0.1738(2)	0.084(2)
C(23)	0.0919(2)	0.35691(18)	−0.14234(14)	0.024(1)
C(24)	0.0480(2)	0.30948(19)	−0.20648(14)	0.026(1)
C(25)	0.0872(2)	0.22233(19)	−0.20927(15)	0.029(1)
C(26)	0.1635(2)	0.18379(18)	−0.15031(14)	0.027(1)
C(27)	−0.0878(3)	0.2874(3)	−0.33105(18)	0.062(1)
C(28)	−0.1242(3)	0.4012(2)	−0.24750(19)	0.049(1)
C(29)	0.0261(3)	0.4284(3)	−0.3015(2)	0.077(2)
C(31)	0.4240(2)	0.12277(18)	0.21734(14)	0.027(1)
C(33)	0.3173(3)	0.1609(2)	0.22513(16)	0.040(1)
C(32)	0.5653(2)	0.10729(19)	0.12100(16)	0.029(1)
C(34)	0.5230(2)	0.1613(2)	0.27462(14)	0.034(1)
C(35)	0.5918(2)	0.0091(2)	0.14267(19)	0.039(1)
C(36)	0.5808(3)	0.1272(2)	0.04752(18)	0.044(1)
C(41)	0.1422(2)	0.44127(18)	0.02958(16)	0.029(1)
C(42)	0.3240(2)	0.46742(18)	−0.03598(16)	0.028(1)
C(43)	0.0943(2)	0.3754(2)	0.07316(17)	0.035(1)
C(44)	0.1860(3)	0.5258(2)	0.0733(2)	0.054(1)
C(45)	0.4121(2)	0.4190(2)	−0.06081(16)	0.033(1)
C(46)	0.2589(3)	0.5316(2)	−0.0953(2)	0.049(1)
C	0.2872(2)	0.18880(16)	−0.02011(13)	0.019(1)
H(13)	0.3623	−0.0457	0.1373	0.024
H(15)	0.0766	−0.0627	−0.0192	0.027
H(16)	0.1101	0.0746	−0.0657	0.027
H(19A)	0.0321	−0.1707	0.0542	0.055
H(19B)	0.0880	−0.2675	0.0736	0.055
H(19C)	0.0934	−0.2161	0.0013	0.055
H(18A)	0.2909	−0.2343	0.0393	0.072
H(18B)	0.2843	−0.2842	0.1120	0.072
H(18C)	0.3626	−0.1988	0.1171	0.072
H(17A)	0.1395	−0.1115	0.1758	0.127
H(17B)	0.2689	−0.1237	0.2016	0.127
H(17C)	0.1914	−0.2098	0.1948	0.127

Continued on next page

Table S17. – continued from previous page

atom	x	y	x	U(eq)
H(23)	0.0684	0.4170	−0.1388	0.029
H(25)	0.0610	0.1883	−0.2528	0.035
H(26)	0.1885	0.1243	−0.1546	0.033
H(27A)	−0.1432	0.3183	−0.3696	0.093
H(27B)	−0.1223	0.2394	−0.3103	0.093
H(27C)	−0.0328	0.2612	−0.3514	0.093
H(28A)	−0.1775	0.4270	−0.2901	0.073
H(28B)	−0.0927	0.4494	−0.2129	0.073
H(28C)	−0.1602	0.3571	−0.2241	0.073
H(29A)	0.0790	0.4003	−0.3229	0.116
H(29B)	0.0641	0.4687	−0.2619	0.116
H(29C)	−0.0267	0.4632	−0.3390	0.116
H(31)	0.4233	0.0561	0.2249	0.032
H(33A)	0.2567	0.1400	0.1845	0.060
H(33B)	0.3201	0.2269	0.2248	0.060
H(33C)	0.3066	0.1403	0.2711	0.060
H(32)	0.6190	0.1452	0.1573	0.035
H(34A)	0.5893	0.1322	0.2701	0.051
H(34B)	0.5155	0.1501	0.3233	0.051
H(34C)	0.5274	0.2264	0.2671	0.051
H(35A)	0.5369	−0.0302	0.1109	0.059
H(35B)	0.5918	0.0003	0.1933	0.059
H(35C)	0.6638	−0.0059	0.1377	0.059
H(36A)	0.5278	0.0927	0.0101	0.066
H(36B)	0.6547	0.1101	0.0476	0.066
H(36C)	0.5701	0.1917	0.0371	0.066
H(41)	0.0823	0.4601	−0.0141	0.035
H(42)	0.3619	0.5054	0.0069	0.033
H(43A)	0.0328	0.4038	0.0854	0.053
H(43B)	0.1502	0.3589	0.1180	0.053
H(43C)	0.0692	0.3211	0.0441	0.053
H(44A)	0.2144	0.5672	0.0433	0.081
H(44B)	0.2445	0.5093	0.1168	0.081
H(44C)	0.1271	0.5554	0.0879	0.081
H(45A)	0.3782	0.3746	−0.0982	0.050
H(45B)	0.4620	0.3884	−0.0192	0.050
H(45C)	0.4526	0.4630	−0.0810	0.050
H(46A)	0.2291	0.4979	−0.1406	0.074
H(46B)	0.3069	0.5795	−0.1032	0.074
H(46C)	0.1992	0.5583	−0.0798	0.074
H	0.3501	0.1730	−0.0388	0.023

Table S18. Anisotropic displacement parameters (Å^2) for [{PC(sp^3)HP}^{tBu}PdI] (**6**). The anisotropic displacement factor exponent takes the form: $-2\pi^2[h^2a^{*2}U_{11} + ... + 2hka^*b^*U_{12}]$.

**atom**	**U$_{11}$**	**U$_{22}$**	**U$_{33}$**	**U$_{23}$**	**U$_{13}$**	**U$_{12}$**
P(1)	0.0167(3)	0.0159(3)	0.0162(3)	−0.0006(2)	−0.0013(3)	−0.0020(3)
P(2)	0.0235(3)	0.0128(3)	0.0225(3)	0.0006(3)	0.0072(3)	−0.0002(3)
C(10)	0.0334(16)	0.0236(14)	0.0251(14)	0.0014(11)	0.0047(12)	−0.0097(12)
C(11)	0.0203(13)	0.0146(12)	0.0180(12)	−0.0042(10)	0.0020(10)	0.0008(10)
C(12)	0.0161(13)	0.0178(12)	0.0175(12)	−0.0027(10)	0.0006(10)	−0.0005(10)
C(13)	0.0205(13)	0.0181(12)	0.0185(13)	0.0009(10)	0.0006(10)	0.0014(10)
C(14)	0.0232(14)	0.0178(12)	0.0233(13)	−0.0027(11)	0.0076(11)	−0.0017(11)
C(15)	0.0171(13)	0.0197(13)	0.0282(14)	−0.0071(11)	0.0016(11)	−0.0021(10)
C(16)	0.0206(13)	0.0178(13)	0.0224(13)	−0.0034(11)	−0.0047(11)	0.0043(11)
I	0.0739(2)	0.0235(1)	0.0193(1)	−0.0051(1)	0.0025(1)	−0.0213(1)
Pd	0.0207(1)	0.0129(1)	0.0145(1)	−0.0014(1)	0.0027(1)	−0.0029(1)
C(22)	0.0195(13)	0.0181(12)	0.0214(13)	0.0026(10)	0.0049(11)	0.0003(10)
C(21)	0.0215(13)	0.0182(12)	0.0205(13)	0.0022(10)	0.0041(11)	0.0010(11)
C(20)	0.0277(16)	0.0439(18)	0.0277(16)	0.0160(14)	−0.0006(12)	0.0082(14)
C(19)	0.0267(16)	0.0217(14)	0.059(2)	0.0047(14)	0.0091(14)	−0.0075(12)
C(18)	0.0297(17)	0.0231(15)	0.076(3)	0.0209(16)	−0.0079(16)	−0.0047(13)
C(17)	0.165(5)	0.058(3)	0.042(2)	−0.0106(19)	0.049(3)	−0.064(3)
C(23)	0.0224(14)	0.0214(13)	0.0287(15)	0.0069(11)	0.0070(11)	0.0056(11)
C(24)	0.0225(14)	0.0314(15)	0.0226(14)	0.0087(12)	0.0040(11)	0.0040(12)
C(25)	0.0286(15)	0.0326(15)	0.0199(14)	0.0010(12)	−0.0020(11)	0.0042(13)
C(26)	0.0327(16)	0.0207(13)	0.0225(14)	−0.0018(11)	−0.0010(12)	0.0061(12)
C(27)	0.067(3)	0.075(3)	0.0273(18)	−0.0010(18)	−0.0149(17)	0.035(2)
C(28)	0.0346(18)	0.051(2)	0.050(2)	0.0084(17)	−0.0051(16)	0.0159(16)
C(29)	0.044(2)	0.109(4)	0.069(3)	0.068(3)	0.002(2)	0.006(2)
C(31)	0.0321(15)	0.0251(14)	0.0166(13)	0.0032(11)	−0.0029(11)	−0.0099(12)
C(33)	0.0381(18)	0.061(2)	0.0239(15)	0.0013(15)	0.0131(14)	−0.0114(16)
C(32)	0.0181(14)	0.0292(15)	0.0352(16)	−0.0071(13)	0.0009(12)	−0.0014(12)
C(34)	0.0418(18)	0.0350(16)	0.0164(14)	−0.0004(12)	−0.0053(12)	−0.0078(14)
C(35)	0.0232(16)	0.0326(16)	0.053(2)	−0.0084(15)	−0.0042(14)	0.0071(13)
C(36)	0.0330(18)	0.056(2)	0.0449(19)	−0.0057(16)	0.0151(15)	0.0044(16)
C(41)	0.0283(15)	0.0221(14)	0.0394(17)	−0.0061(12)	0.0123(13)	0.0023(12)
C(42)	0.0294(15)	0.0214(14)	0.0315(15)	0.0056(12)	0.0072(12)	−0.0046(12)
C(43)	0.0295(16)	0.0429(18)	0.0375(17)	−0.0071(14)	0.0153(14)	−0.0035(14)
C(44)	0.056(2)	0.0314(18)	0.086(3)	−0.0284(18)	0.038(2)	−0.0060(16)
C(45)	0.0328(16)	0.0394(17)	0.0287(16)	0.0032(13)	0.0113(13)	−0.0082(13)
C(46)	0.045(2)	0.0354(18)	0.064(2)	0.0288(17)	0.0089(18)	−0.0028(15)
C	0.0219(13)	0.0158(12)	0.0174(12)	−0.0018(10)	0.0037(10)	0.0025(10)

Table S19. Distances [Å] for [{PC(sp^3)HP}^{tBu}PdI] (**6**).

atom – atom	distance	atom – atom	distance
P(1) – C(12)	1.822(2)	P(1) – C(32)	1.840(3)
P(1) – C(31)	1.845(3)	P(1) – Pd	2.3123(7)
P(2) – C(22)	1.813(3)	P(2) – C(42)	1.843(3)
P(2) – C(41)	1.846(3)	P(2) – Pd	2.2698(7)
C(10) – C(19)	1.525(4)	C(10) – C(14)	1.529(4)
C(10) – C(17)	1.530(4)	C(10) – C(18)	1.531(4)
C(11) – C(16)	1.393(3)	C(11) – C(12)	1.406(3)
C(11) – C	1.516(3)	C(12) – C(13)	1.388(3)
C(13) – C(14)	1.396(4)	C(13) – H(13)	0.9500
C(14) – C(15)	1.388(4)	C(15) – C(16)	1.389(4)
C(15) – H(15)	0.9500	C(16) – H(16)	0.9500
I – Pd	2.6763(3)	Pd – C	2.094(2)
C(22) – C(23)	1.396(4)	C(22) – C(21)	1.397(4)
C(21) – C(26)	1.394(4)	C(21) – C	1.518(3)
C(20) – C(27)	1.525(5)	C(20) – C(28)	1.528(5)
C(20) – C(24)	1.536(4)	C(20) – C(29)	1.539(5)
C(19) – H(19A)	0.9800	C(19) – H(19B)	0.9800
C(19) – H(19C)	0.9800	C(18) – H(18A)	0.9800
C(18) – H(18B)	0.9800	C(18) – H(18C)	0.9800
C(17) – H(17A)	0.9800	C(17) – H(17B)	0.9800
C(17) – H(17C)	0.9800	C(23) – C(24)	1.389(4)
C(23) – H(23)	0.9500	C(24) – C(25)	1.394(4)
C(25) – C(26)	1.388(4)	C(25) – H(25)	0.9500
C(26) – H(26)	0.9500	C(27) – H(27A)	0.9800
C(27) – H(27B)	0.9800	C(27) – H(27C)	0.9800
C(28) – H(28A)	0.9800	C(28) – H(28B)	0.9800
C(28) – H(28C)	0.9800	C(29) – H(29A)	0.9800
C(29) – H(29B)	0.9800	C(29) – H(29C)	0.9800
C(31) – C(33)	1.527(4)	C(31) – C(34)	1.531(4)
C(31) – H(31)	1.0000	C(33) – H(33A)	0.9800
C(33) – H(33B)	0.9800	C(33) – H(33C)	0.9800
C(32) – C(36)	1.507(4)	C(32) – C(35)	1.526(4)
C(32) – H(32)	1.0000	C(34) – H(34A)	0.9800
C(34) – H(34B)	0.9800	C(34) – H(34C)	0.9800
C(35) – H(35A)	0.9800	C(35) – H(35B)	0.9800
C(35) – H(35C)	0.9800	C(36) – H(36A)	0.9800
C(36) – H(36B)	0.9800	C(36) – H(36C)	0.9800
C(41) – C(44)	1.523(4)	C(41) – C(43)	1.525(4)
C(41) – H(41)	1.0000	C(42) – C(45)	1.525(4)
C(42) – C(46)	1.533(4)	C(42) – H(42)	1.0000
C(43) – H(43A)	0.9800	C(43) – H(43B)	0.9800
C(43) – H(43C)	0.9800	C(44) – H(44A)	0.9800
C(44) – H(44B)	0.9800	C(44) – H(44C)	0.9800
C(45) – H(45A)	0.9800	C(45) – H(45B)	0.9800
C(45) – H(45C)	0.9800	C(46) – H(46A)	0.9800
C(46) – H(46B)	0.9800	C(46) – H(46C)	0.9800
C – H	1.0000		

Table S20. Angles [°] for [{PC(sp^3)HP}^{tBu}PdI] (**6**).

atom – atom – atom	angle	atom – atom – atom	angle
C(12) – P(1) – C(32)	109.27(12)	C(12) – P(1) – C(31)	102.36(12)
C(32) – P(1) – C(31)	105.98(13)	C(12) – P(1) – Pd	99.17(8)
C(32) – P(1) – Pd	120.50(10)	C(31) – P(1) – Pd	117.56(10)
C(22) – P(2) – C(42)	106.02(12)	C(22) – P(2) – C(41)	105.37(13)
C(42) – P(2) – C(41)	107.41(13)	C(22) – P(2) – Pd	103.90(8)
C(42) – P(2) – Pd	112.64(9)	C(41) – P(2) – Pd	120.31(10)
C(19) – C(10) – C(14)	112.8(2)	C(19) – C(10) – C(17)	109.2(3)
C(14) – C(10) – C(17)	109.0(2)	C(19) – C(10) – C(18)	107.0(2)
C(14) – C(10) – C(18)	108.7(2)	C(17) – C(10) – C(18)	110.1(3)
C(16) – C(11) – C(12)	116.5(2)	C(16) – C(11) – C	125.5(2)
C(12) – C(11) – C	117.9(2)	C(13) – C(12) – C(11)	121.0(2)
C(13) – C(12) – P(1)	123.98(19)	C(11) – C(12) – P(1)	114.09(18)
C(12) – C(13) – C(14)	122.1(2)	C(12) – C(13) – H(13)	118.9
C(14) – C(13) – H(13)	118.9	C(15) – C(14) – C(13)	116.4(2)
C(15) – C(14) – C(10)	123.9(2)	C(13) – C(14) – C(10)	119.6(2)
C(14) – C(15) – C(16)	122.0(2)	C(14) – C(15) – H(15)	119.0
C(16) – C(15) – H(15)	119.0	C(15) – C(16) – C(11)	121.7(2)
C(15) – C(16) – H(16)	119.1	C(11) – C(16) – H(16)	119.1
C – Pd – P(2)	84.74(7)	C – Pd – P(1)	82.49(7)
P(2) – Pd – P(1)	165.40(2)	C – Pd – I	172.41(7)
P(2) – Pd – I	95.04(2)	P(1) – Pd – I	98.557(18)
C(23) – C(22) – C(21)	120.7(2)	C(23) – C(22) – P(2)	124.9(2)
C(21) – C(22) – P(2)	113.96(18)	C(26) – C(21) – C(22)	117.0(2)
C(26) – C(21) – C	121.9(2)	C(22) – C(21) – C	121.0(2)
C(27) – C(20) – C(28)	107.5(3)	C(27) – C(20) – C(24)	112.3(3)
C(28) – C(20) – C(24)	110.6(2)	C(27) – C(20) – C(29)	110.7(3)
C(28) – C(20) – C(29)	107.1(3)	C(24) – C(20) – C(29)	108.4(2)
C(10) – C(19) – H(19A)	109.5	C(10) – C(19) – H(19B)	109.5
H(19A) – C(19) – H(19B)	109.5	C(10) – C(19) – H(19C)	109.5
H(19A) – C(19) – H(19C)	109.5	H(19B) – C(19) – H(19C)	109.5
C(10) – C(18) – H(18A)	109.5	C(10) – C(18) – H(18B)	109.5
H(18A) – C(18) – H(18B)	109.5	C(10) – C(18) – H(18C)	109.5
H(18A) – C(18) – H(18C)	109.5	H(18B) – C(18) – H(18C)	109.5
C(10) – C(17) – H(17A)	109.5	C(10) – C(17) – H(17B)	109.5
H(17A) – C(17) – H(17B)	109.5	C(10) – C(17) – H(17C)	109.5
H(17A) – C(17) – H(17C)	109.5	H(17B) – C(17) – H(17C)	109.5
C(24) – C(23) – C(22)	122.3(2)	C(24) – C(23) – H(23)	118.8
C(22) – C(23) – H(23)	118.8	C(23) – C(24) – C(25)	116.4(2)
C(23) – C(24) – C(20)	120.5(3)	C(25) – C(24) – C(20)	123.0(3)
C(26) – C(25) – C(24)	121.9(3)	C(26) – C(25) – H(25)	119.1
C(24) – C(25) – H(25)	119.1	C(25) – C(26) – C(21)	121.5(2)
C(25) – C(26) – H(26)	119.3	C(21) – C(26) – H(26)	119.3
C(20) – C(27) – H(27A)	109.5	C(20) – C(27) – H(27B)	109.5
H(27A) – C(27) – H(27B)	109.5	C(20) – C(27) – H(27C)	109.5
H(27A) – C(27) – H(27C)	109.5	H(27B) – C(27) – H(27C)	109.5
C(20) – C(28) – H(28A)	109.5	C(20) – C(28) – H(28B)	109.5
H(28A) – C(28) – H(28B)	109.5	C(20) – C(28) – H(28C)	109.5
H(28A) – C(28) – H(28C)	109.5	H(28B) – C(28) – H(28C)	109.5
C(20) – C(29) – H(29A)	109.5	C(20) – C(29) – H(29B)	109.5
H(29A) – C(29) – H(29B)	109.5	C(20) – C(29) – H(29C)	109.5

Continued on next page

Table S20. – continued from previous page

**atom – atom – atom**	**angle**	**atom – atom – atom**	**angle**
H(29A) – C(29) – H(29C)	109.5	H(29B) – C(29) – H(29C)	109.5
C(33) – C(31) – C(34)	111.9(2)	C(33) – C(31) – P(1)	108.49(18)
C(34) – C(31) – P(1)	112.78(19)	C(33) – C(31) – H(31)	107.8
C(34) – C(31) – H(31)	107.8	P(1) – C(31) – H(31)	107.8
C(31) – C(33) – H(33A)	109.5	C(31) – C(33) – H(33B)	109.5
H(33A) – C(33) – H(33B)	109.5	C(31) – C(33) – H(33C)	109.5
H(33A) – C(33) – H(33C)	109.5	H(33B) – C(33) – H(33C)	109.5
C(36) – C(32) – C(35)	111.2(3)	C(36) – C(32) – P(1)	110.3(2)
C(35) – C(32) – P(1)	114.0(2)	C(36) – C(32) – H(32)	106.9
C(35) – C(32) – H(32)	106.9	P(1) – C(32) – H(32)	106.9
C(31) – C(34) – H(34A)	109.5	C(31) – C(34) – H(34B)	109.5
H(34A) – C(34) – H(34B)	109.5	C(31) – C(34) – H(34C)	109.5
H(34A) – C(34) – H(34C)	109.5	H(34B) – C(34) – H(34C)	109.5
C(32) – C(35) – H(35A)	109.5	C(32) – C(35) – H(35B)	109.5
H(35A) – C(35) – H(35B)	109.5	C(32) – C(35) – H(35C)	109.5
H(35A) – C(35) – H(35C)	109.5	H(35B) – C(35) – H(35C)	109.5
C(32) – C(36) – H(36A)	109.5	C(32) – C(36) – H(36B)	109.5
H(36A) – C(36) – H(36B)	109.5	C(32) – C(36) – H(36C)	109.5
H(36A) – C(36) – H(36C)	109.5	H(36B) – C(36) – H(36C)	109.5
C(44) – C(41) – C(43)	111.6(3)	C(44) – C(41) – P(2)	113.2(2)
C(43) – C(41) – P(2)	108.6(2)	C(44) – C(41) – H(41)	107.7
C(43) – C(41) – H(41)	107.7	P(2) – C(41) – H(41)	107.7
C(45) – C(42) – C(46)	110.8(3)	C(45) – C(42) – P(2)	107.88(19)
C(46) – C(42) – P(2)	116.7(2)	C(45) – C(42) – H(42)	107.0
C(46) – C(42) – H(42)	107.0	P(2) – C(42) – H(42)	107.0
C(41) – C(43) – H(43A)	109.5	C(41) – C(43) – H(43B)	109.5
H(43A) – C(43) – H(43B)	109.5	C(41) – C(43) – H(43C)	109.5
H(43A) – C(43) – H(43C)	109.5	H(43B) – C(43) – H(43C)	109.5
C(41) – C(44) – H(44A)	109.5	C(41) – C(44) – H(44B)	109.5
H(44A) – C(44) – H(44B)	109.5	C(41) – C(44) – H(44C)	109.5
H(44A) – C(44) – H(44C)	109.5	H(44B) – C(44) – H(44C)	109.5
C(42) – C(45) – H(45A)	109.5	C(42) – C(45) – H(45B)	109.5
H(45A) – C(45) – H(45B)	109.5	C(42) – C(45) – H(45C)	109.5
H(45A) – C(45) – H(45C)	109.5	H(45B) – C(45) – H(45C)	109.5
C(42) – C(46) – H(46A)	109.5	C(42) – C(46) – H(46B)	109.5
H(46A) – C(46) – H(46B)	109.5	C(42) – C(46) – H(46C)	109.5
H(46A) – C(46) – H(46C)	109.5	H(46B) – C(46) – H(46C)	109.5
C(11) – C – C(21)	116.7(2)	C(11) – C – Pd	109.32(16)
C(21) – C – Pd	115.91(16)	C(11) – C – H	104.4
C(21) – C – H	104.4	Pd – C – H	104.4

5.4 Crystal data for [{PC(sp^3)(SPh)P}^{tBu}PdOTf]·Et$_2$O (8·Et$_2$O)

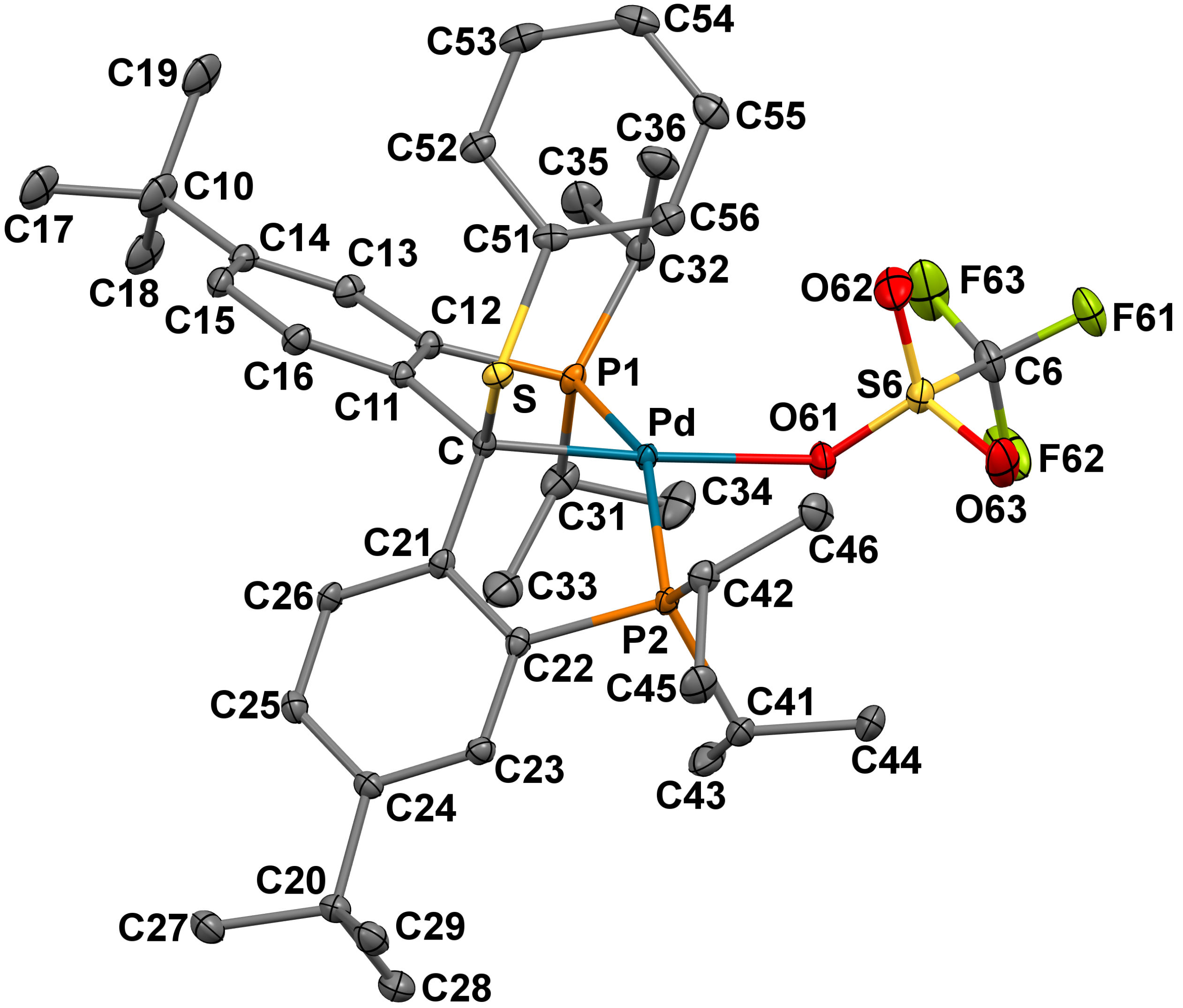

Figure S47. Thermal-ellipsoid representation of [{PC(sp^3)(SPh)P}^{tBu}PdOTf]·Et$_2$O (**8·Et$_2$O**) at 50% probability. Hydrogen atoms and the solvent were omitted for clarity.

Table S21. Crystal data and structure refinement for [{PC(sp^3)(SPh)P}^{tBu}PdOTf]·Et_2O (**8·Et₂O**).

Identification code:	pc39a	
Empirical formula:	$C_{44}H_{67}F_3O_4P_2PdS_2$	
Formula weight:	949.44	
Temperature:	120(2) K	
Wavelength:	0.71073 Å	
Crystal system:	Orthorhombic	
Space group:	$P2_12_12_1$	
Unit cell dimensions:	*a* = 14.0266(6) Å	α = 90°
	b = 15.2323(6) Å	β = 90°
	c = 21.3735(9) Å	γ = 90°
Volume:	4566.6(3) $Å^3$	
Z:	4	
Density (calculated):	1.381 $g·cm^{-3}$	
Absorption coefficient (μ):	0.620 mm^{-1}	
F(000):	1992	
Crystal size:	0.09 × 0.08 × 0.02 mm^3	
θ range for data collection:	1.64 to 26.91°	
Index ranges:	−13 ≤ h ≤ 17, −19 ≤ k ≤ 18, −27 ≤ l ≤ 27	
Reflections collected:	77120	
Independent reflections:	9882 [R_{int} = 0.0493]	
Completeness to θ = 25.00°:	100.0 %	
Absorption correction:	Semi-empirical from equivalents	
Max. and min. transmission:	1.0000 and 0.9527	
Refinement method:	Full-matrix least-squares on F^2	
Data / restraints / parameters:	9882 / 0 / 516	
Goodness-of-fit on F^2:	1.012	
Final R indices [I>2σ(I)]:	R_1 = 0.0260, wR_2 = 0.0561	
R indices (all data):	R_1 = 0.0331, wR_2 = 0.0590	
Absolute structure parameter:	−0.022(15)	
Largest diff. peak and hole:	0.885 and −0.528 $e^-·Å^{-3}$	

Table S22. Atomic coordinates and equivalent isotropic displacement parameters (Å^2) for [{PC(sp^3)(SPh)P}^{tBu}PdOTf]·Et$_2$O (**8·Et$_2$O**). U(eq) is defined as one third of the trace of the orthogonalized U_{ij} tensor

atom	x	y	z	U(eq)
Pd	0.41412(1)	0.49818(1)	0.47367(1)	0.014(1)
P(1)	0.40919(5)	0.63744(4)	0.51194(3)	0.016(1)
S	0.36480(4)	0.50739(4)	0.33191(2)	0.017(1)
P(2)	0.49064(4)	0.37483(4)	0.43015(3)	0.016(1)
C(13)	0.42178(18)	0.79545(14)	0.44103(12)	0.020(1)
C(20)	0.82267(18)	0.42296(16)	0.31278(12)	0.021(1)
C(19)	0.3181(4)	0.9682(3)	0.4004(3)	0.035(1)
C(18)	0.4863(4)	0.9812(3)	0.4289(3)	0.035(1)
C(17)	0.4416(4)	0.9789(3)	0.3175(3)	0.035(1)
C(22)	0.57058(17)	0.43737(14)	0.38026(11)	0.016(1)
C(21)	0.54495(17)	0.52493(14)	0.36924(11)	0.015(1)
C(23)	0.65872(18)	0.40638(16)	0.36027(11)	0.018(1)
C(24)	0.72394(18)	0.45989(16)	0.32961(12)	0.017(1)
C(25)	0.69844(19)	0.54656(16)	0.31983(13)	0.021(1)
C(26)	0.61033(18)	0.57858(15)	0.33895(12)	0.019(1)
C(27)	0.88033(18)	0.48744(19)	0.27385(12)	0.028(1)
C(28)	0.87611(19)	0.40438(18)	0.37382(13)	0.027(1)
C(29)	0.81115(19)	0.33739(17)	0.27569(13)	0.026(1)
C(31)	0.5110(2)	0.66463(17)	0.56221(12)	0.026(1)
C(32)	0.30344(19)	0.67426(16)	0.55516(12)	0.022(1)
C(33)	0.60362(19)	0.63806(18)	0.53013(16)	0.033(1)
C(34)	0.5015(2)	0.6218(2)	0.62614(13)	0.039(1)
C(35)	0.3041(2)	0.77016(17)	0.57625(15)	0.034(1)
C(36)	0.21363(18)	0.65254(18)	0.51810(14)	0.030(1)
C(41)	0.57276(18)	0.31416(15)	0.48191(12)	0.021(1)
C(42)	0.43322(18)	0.29394(15)	0.37851(12)	0.021(1)
C(43)	0.6265(2)	0.37898(18)	0.52301(16)	0.030(1)
C(44)	0.52182(19)	0.24488(17)	0.52167(13)	0.028(1)
C(45)	0.5023(2)	0.22624(16)	0.35127(13)	0.028(1)
C(46)	0.3452(2)	0.25127(17)	0.40834(13)	0.028(1)
C(51)	0.24957(18)	0.54088(16)	0.35665(11)	0.018(1)
C(52)	0.20881(18)	0.61731(17)	0.33375(11)	0.022(1)
C(53)	0.11612(19)	0.63889(18)	0.35044(12)	0.027(1)
C(54)	0.06470(19)	0.58479(18)	0.38934(13)	0.028(1)
C(55)	0.10513(17)	0.50864(19)	0.41262(11)	0.026(1)
C(56)	0.19774(17)	0.48723(17)	0.39648(11)	0.022(1)
C	0.44611(17)	0.55644(14)	0.38973(11)	0.015(1)
C(14)	0.42412(18)	0.84165(15)	0.38536(11)	0.018(1)
C(16)	0.43488(17)	0.70222(15)	0.33139(12)	0.020(1)
C(15)	0.42920(18)	0.79270(15)	0.33061(12)	0.021(1)
C(11)	0.43422(16)	0.65597(15)	0.38803(11)	0.016(1)
C(10)	0.4191(2)	0.94215(16)	0.38394(14)	0.035(1)
O(7)	0.17564(14)	0.25158(13)	0.26152(9)	0.033(1)
C(12)	0.42323(18)	0.70360(14)	0.44253(11)	0.017(1)
F(61)	0.18489(12)	0.38273(11)	0.67857(7)	0.037(1)
O(61)	0.36997(13)	0.43521(11)	0.56108(8)	0.024(1)
F(62)	0.33225(13)	0.41445(16)	0.69062(8)	0.055(1)
O(62)	0.20182(14)	0.41398(14)	0.53911(9)	0.039(1)

Continued on next page

Table S22. – continued from previous page

atom	x	y	x	U(eq)
F(63)	0.23599(15)	0.51005(13)	0.65394(8)	0.055(1)
C(71)	0.3237(2)	0.3077(2)	0.22396(14)	0.035(1)
C(72)	0.2321(2)	0.32779(18)	0.25740(14)	0.032(1)
C(73)	0.0930(2)	0.2671(2)	0.29785(14)	0.037(1)
C(74)	0.0387(2)	0.1834(2)	0.30582(16)	0.043(1)
C(91)	0.5072(5)	0.9749(3)	0.3518(3)	0.035(1)
C(93)	0.3288(5)	0.9727(4)	0.3523(3)	0.035(1)
C(96)	0.4181(5)	0.9817(3)	0.4509(3)	0.035(1)
O(63)	0.29479(16)	0.29528(12)	0.58236(11)	0.041(1)
C(6)	0.2578(2)	0.42570(18)	0.65383(12)	0.026(1)
S(6)	0.28217(5)	0.38745(4)	0.57483(3)	0.021(1)
H(13)	0.4191	0.8270	0.4793	0.024
H(19A)	0.2733	0.9278	0.3802	0.052
H(19B)	0.3060	1.0282	0.3857	0.052
H(19C)	0.3097	0.9656	0.4459	0.052
H(18A)	0.5518	0.9689	0.4155	0.052
H(18B)	0.4755	0.9558	0.4704	0.052
H(18C)	0.4764	1.0448	0.4306	0.052
H(17A)	0.5091	0.9693	0.3079	0.052
H(17B)	0.4278	1.0419	0.3164	0.052
H(17C)	0.4021	0.9486	0.2865	0.052
H(23)	0.6749	0.3467	0.3678	0.021
H(25)	0.7420	0.5850	0.2996	0.025
H(26)	0.5946	0.6382	0.3311	0.023
H(27A)	0.9420	0.4612	0.2632	0.042
H(27B)	0.8907	0.5414	0.2979	0.042
H(27C)	0.8455	0.5013	0.2354	0.042
H(28A)	0.9408	0.3842	0.3642	0.041
H(28B)	0.8423	0.3589	0.3975	0.041
H(28C)	0.8794	0.4582	0.3988	0.041
H(29A)	0.7758	0.3492	0.2370	0.040
H(29B)	0.7761	0.2945	0.3010	0.040
H(29C)	0.8742	0.3138	0.2653	0.040
H(31)	0.5120	0.7297	0.5683	0.031
H(32)	0.3007	0.6380	0.5941	0.026
H(33A)	0.6056	0.5741	0.5255	0.050
H(33B)	0.6579	0.6574	0.5555	0.050
H(33C)	0.6070	0.6656	0.4888	0.050
H(34A)	0.4914	0.5586	0.6210	0.058
H(34B)	0.4472	0.6474	0.6484	0.058
H(34C)	0.5599	0.6318	0.6503	0.058
H(35A)	0.2991	0.8085	0.5396	0.051
H(35B)	0.3636	0.7826	0.5985	0.051
H(35C)	0.2499	0.7808	0.6042	0.051
H(36A)	0.2154	0.6830	0.4777	0.046
H(36B)	0.1574	0.6716	0.5417	0.046
H(36C)	0.2103	0.5890	0.5110	0.046
H(41)	0.6204	0.2833	0.4549	0.025
H(42)	0.4088	0.3281	0.3419	0.025
H(43A)	0.6605	0.4212	0.4965	0.046
H(43B)	0.6724	0.3472	0.5491	0.046

Continued on next page

Table S22. – continued from previous page

atom	x	y	x	U(eq)
H(43C)	0.5813	0.4103	0.5499	0.046
H(44A)	0.4941	0.2001	0.4943	0.042
H(44B)	0.4711	0.2729	0.5461	0.042
H(44C)	0.5677	0.2173	0.5501	0.042
H(45A)	0.4682	0.1882	0.3218	0.041
H(45B)	0.5289	0.1906	0.3852	0.041
H(45C)	0.5540	0.2566	0.3293	0.041
H(46A)	0.3098	0.2186	0.3764	0.042
H(46B)	0.3042	0.2970	0.4261	0.042
H(46C)	0.3654	0.2110	0.4415	0.042
H(52)	0.2441	0.6547	0.3068	0.026
H(53)	0.0880	0.6913	0.3349	0.033
H(54)	0.0011	0.5998	0.4003	0.033
H(55)	0.0695	0.4713	0.4395	0.031
H(56)	0.2260	0.4354	0.4128	0.026
H(16)	0.4393	0.6710	0.2930	0.024
H(15)	0.4288	0.8224	0.2915	0.025
H(71A)	0.3101	0.2929	0.1803	0.052
H(71B)	0.3554	0.2580	0.2443	0.052
H(71C)	0.3655	0.3593	0.2255	0.052
H(72A)	0.2462	0.3500	0.2999	0.038
H(72B)	0.1969	0.3740	0.2345	0.038
H(73A)	0.0523	0.3113	0.2769	0.044
H(73B)	0.1115	0.2905	0.3394	0.044
H(74A)	0.0173	0.1623	0.2648	0.064
H(74B)	−0.0168	0.1938	0.3326	0.064
H(74C)	0.0800	0.1392	0.3252	0.064
H(91A)	0.5132	0.9466	0.3108	0.052
H(91B)	0.5632	0.9609	0.3773	0.052
H(91C)	0.5028	1.0387	0.3462	0.052
H(93A)	0.3306	0.9564	0.3080	0.052
H(93B)	0.3235	1.0366	0.3560	0.052
H(93C)	0.2737	0.9449	0.3723	0.052
H(96A)	0.4204	1.0459	0.4482	0.052
H(96B)	0.4737	0.9605	0.4742	0.052
H(96C)	0.3597	0.9637	0.4725	0.052

Table S23. Anisotropic displacement parameters ($Å^2$) for [{PC(sp^3)(SPh)P}^{tBu}PdOTf]·Et_2O (**8·Et$_2$O**). The anisotropic displacement factor exponent takes the form: $-2\pi^2[h^2a^{*2}U_{11} + ... + 2hka^*b^*U_{12}]$.

atom	U_{11}	U_{22}	U_{33}	U_{23}	U_{13}	U_{12}
Pd	0.0159(1)	0.0101(1)	0.0145(1)	0.0012(1)	0.0013(1)	0.0000(1)
P(1)	0.0214(3)	0.0118(3)	0.0156(3)	−0.0007(2)	−0.0011(3)	−0.0003(2)
S	0.0182(3)	0.0150(3)	0.0168(2)	−0.0024(3)	−0.0002(2)	0.0018(3)
P(2)	0.0171(3)	0.0114(3)	0.0184(3)	0.0019(2)	0.0030(3)	0.0002(2)
C(13)	0.0216(13)	0.0135(10)	0.0245(12)	−0.0018(9)	−0.0024(11)	−0.0022(10)
C(20)	0.0161(13)	0.0214(13)	0.0249(13)	0.0022(10)	0.0022(11)	0.0020(10)
C(19)	0.0470(12)	0.0109(7)	0.0471(11)	0.0023(7)	−0.0045(10)	−0.0007(8)
C(18)	0.0470(12)	0.0109(7)	0.0471(11)	0.0023(7)	−0.0045(10)	−0.0007(8)
C(17)	0.0470(12)	0.0109(7)	0.0471(11)	0.0023(7)	−0.0045(10)	−0.0007(8)
C(22)	0.0182(13)	0.0135(11)	0.0161(11)	0.0009(9)	−0.0018(10)	−0.0028(10)
C(21)	0.0198(12)	0.0115(12)	0.0133(11)	−0.0011(8)	0.0010(9)	0.0005(9)
C(23)	0.0168(13)	0.0146(11)	0.0223(12)	−0.0002(9)	0.0000(10)	0.0016(9)
C(24)	0.0157(13)	0.0193(12)	0.0173(12)	−0.0004(10)	0.0016(11)	0.0000(10)
C(25)	0.0191(14)	0.0196(13)	0.0230(13)	0.0025(10)	0.0029(11)	−0.0037(10)
C(26)	0.0227(15)	0.0110(11)	0.0240(13)	0.0027(10)	0.0004(11)	−0.0002(9)
C(27)	0.0197(12)	0.0311(16)	0.0332(13)	0.0014(12)	0.0060(10)	−0.0010(12)
C(28)	0.0190(13)	0.0311(15)	0.0318(15)	0.0036(12)	−0.0005(12)	0.0002(11)
C(29)	0.0224(14)	0.0245(13)	0.0326(15)	−0.0026(11)	0.0067(12)	0.0006(11)
C(31)	0.0336(16)	0.0188(12)	0.0248(14)	−0.0019(11)	−0.0106(12)	−0.0006(11)
C(32)	0.0302(15)	0.0152(12)	0.0198(12)	−0.0004(10)	0.0082(11)	0.0009(11)
C(33)	0.0264(16)	0.0293(14)	0.0444(17)	0.0019(14)	−0.0091(15)	−0.0057(11)
C(34)	0.0492(19)	0.0400(17)	0.0266(15)	0.0062(13)	−0.0174(14)	−0.0065(15)
C(35)	0.0426(18)	0.0209(14)	0.0391(16)	−0.0088(12)	0.0132(15)	0.0023(12)
C(36)	0.0233(14)	0.0256(14)	0.0422(17)	−0.0068(13)	0.0060(13)	0.0023(11)
C(41)	0.0215(13)	0.0172(11)	0.0243(13)	0.0049(9)	0.0030(11)	0.0029(10)
C(42)	0.0229(15)	0.0152(11)	0.0245(13)	−0.0024(10)	−0.0005(11)	−0.0015(10)
C(43)	0.0280(15)	0.0274(14)	0.0359(15)	0.0080(13)	−0.0108(14)	0.0011(12)
C(44)	0.0300(15)	0.0242(13)	0.0300(14)	0.0118(12)	0.0021(13)	0.0029(11)
C(45)	0.0347(16)	0.0151(12)	0.0331(15)	−0.0052(11)	0.0035(13)	0.0011(11)
C(46)	0.0262(15)	0.0169(13)	0.0413(16)	−0.0017(11)	0.0021(12)	−0.0057(11)
C(51)	0.0166(13)	0.0193(12)	0.0175(12)	−0.0058(9)	−0.0048(10)	0.0019(10)
C(52)	0.0247(14)	0.0211(12)	0.0202(12)	−0.0018(10)	−0.0036(11)	0.0022(11)
C(53)	0.0284(15)	0.0245(14)	0.0293(14)	−0.0038(11)	−0.0081(12)	0.0106(11)
C(54)	0.0176(14)	0.0330(15)	0.0320(14)	−0.0095(12)	−0.0008(12)	0.0027(11)
C(55)	0.0227(13)	0.0263(13)	0.0297(12)	−0.0057(12)	0.0015(10)	−0.0052(12)
C(56)	0.0231(13)	0.0213(14)	0.0205(11)	−0.0016(10)	−0.0014(10)	0.0007(11)
C	0.0185(13)	0.0123(11)	0.0148(11)	0.0026(9)	0.0009(10)	−0.0006(9)
C(14)	0.0149(12)	0.0137(10)	0.0267(12)	0.0015(9)	−0.0020(11)	−0.0007(10)
C(16)	0.0218(14)	0.0171(11)	0.0199(12)	0.0008(9)	−0.0002(10)	0.0021(10)
C(15)	0.0208(14)	0.0172(11)	0.0238(13)	0.0064(9)	−0.0019(11)	−0.0011(10)
C(11)	0.0137(13)	0.0136(11)	0.0205(12)	0.0033(9)	−0.0016(10)	−0.0012(9)
C(10)	0.0470(12)	0.0109(7)	0.0471(11)	0.0023(7)	−0.0045(10)	−0.0007(8)
O(7)	0.0299(12)	0.0331(11)	0.0371(11)	−0.0085(9)	0.0049(9)	0.0011(9)
C(12)	0.0153(12)	0.0135(10)	0.0216(12)	0.0007(9)	−0.0008(10)	−0.0009(10)
F(61)	0.0366(10)	0.0402(10)	0.0352(9)	−0.0018(8)	0.0174(8)	−0.0115(8)
O(61)	0.0252(10)	0.0259(9)	0.0206(9)	0.0057(7)	0.0049(8)	−0.0034(8)
F(62)	0.0379(11)	0.1066(17)	0.0205(8)	0.0057(10)	−0.0049(8)	−0.0171(11)
O(62)	0.0332(12)	0.0525(13)	0.0312(12)	0.0125(10)	−0.0111(9)	−0.0087(10)
F(63)	0.0781(13)	0.0265(10)	0.0592(11)	−0.0134(9)	0.0357(10)	−0.0056(10)

Continued on next page

Table S23. – continued from previous page

atom	U_{11}	U_{22}	U_{33}	U_{23}	U_{13}	U_{12}
C(71)	0.0313(17)	0.0409(17)	0.0317(16)	−0.0080(13)	0.0010(14)	−0.0004(13)
C(72)	0.0328(17)	0.0271(15)	0.0352(16)	−0.0047(12)	0.0005(13)	0.0003(12)
C(73)	0.0314(17)	0.0420(17)	0.0378(16)	−0.0055(13)	0.0078(15)	0.0064(14)
C(74)	0.0438(19)	0.0420(19)	0.0428(19)	−0.0100(15)	0.0068(16)	−0.0029(15)
C(91)	0.0470(12)	0.0109(7)	0.0471(11)	0.0023(7)	−0.0045(10)	−0.0007(8)
C(93)	0.0470(12)	0.0109(7)	0.0471(11)	0.0023(7)	−0.0045(10)	−0.0007(8)
C(96)	0.0470(12)	0.0109(7)	0.0471(11)	0.0023(7)	−0.0045(10)	−0.0007(8)
O(63)	0.0549(15)	0.0152(9)	0.0535(14)	0.0003(9)	0.0244(12)	−0.0002(9)
C(6)	0.0277(16)	0.0285(15)	0.0225(13)	−0.0020(11)	0.0067(12)	−0.0086(12)
S(6)	0.0270(3)	0.0173(3)	0.0181(3)	0.0019(2)	0.0034(3)	−0.0010(2)

Table S24. Distances [Å] for [{PC(sp^3)(SPh)P}^{tBu}PdOTf]·Et_2O (**8·Et_2O**).

atom – atom	distance	atom – atom	distance
Pd – C	2.051(2)	Pd – O(61)	2.1895(17)
Pd – P(1)	2.2745(6)	Pd – P(2)	2.3554(6)
P(1) – C(12)	1.804(2)	P(1) – C(31)	1.835(3)
P(1) – C(32)	1.835(3)	S – C(51)	1.776(3)
S – C	1.840(2)	P(2) – C(22)	1.817(2)
P(2) – C(42)	1.840(2)	P(2) – C(41)	1.845(2)
C(13) – C(14)	1.383(3)	C(13) – C(12)	1.400(3)
C(13) – H(13)	0.9500	C(20) – C(27)	1.520(4)
C(20) – C(28)	1.531(4)	C(20) – C(29)	1.534(4)
C(20) – C(24)	1.537(3)	C(19) – C(10)	1.512(7)
C(19) – H(19A)	0.9800	C(19) – H(19B)	0.9800
C(19) – H(19C)	0.9800	C(18) – C(10)	1.471(6)
C(18) – H(18A)	0.9800	C(18) – H(18B)	0.9800
C(18) – H(18C)	0.9800	C(17) – C(10)	1.558(6)
C(17) – H(17A)	0.9800	C(17) – H(17B)	0.9800
C(17) – H(17C)	0.9800	C(22) – C(23)	1.391(3)
C(22) – C(21)	1.401(3)	C(21) – C(26)	1.389(3)
C(21) – C	1.531(3)	C(23) – C(24)	1.389(3)
C(23) – H(23)	0.9500	C(24) – C(25)	1.384(3)
C(25) – C(26)	1.390(4)	C(25) – H(25)	0.9500
C(26) – H(26)	0.9500	C(27) – H(27A)	0.9800
C(27) – H(27B)	0.9800	C(27) – H(27C)	0.9800
C(28) – H(28A)	0.9800	C(28) – H(28B)	0.9800
C(28) – H(28C)	0.9800	C(29) – H(29A)	0.9800
C(29) – H(29B)	0.9800	C(29) – H(29C)	0.9800
C(31) – C(34)	1.520(4)	C(31) – C(33)	1.524(4)
C(31) – H(31)	1.0000	C(32) – C(36)	1.524(4)
C(32) – C(35)	1.529(3)	C(32) – H(32)	1.0000
C(33) – H(33A)	0.9800	C(33) – H(33B)	0.9800
C(33) – H(33C)	0.9800	C(34) – H(34A)	0.9800
C(34) – H(34B)	0.9800	C(34) – H(34C)	0.9800
C(35) – H(35A)	0.9800	C(35) – H(35B)	0.9800
C(35) – H(35C)	0.9800	C(36) – H(36A)	0.9800
C(36) – H(36B)	0.9800	C(36) – H(36C)	0.9800
C(41) – C(43)	1.522(4)	C(41) – C(44)	1.532(3)
C(41) – H(41)	1.0000	C(42) – C(45)	1.530(3)
C(42) – C(46)	1.534(4)	C(42) – H(42)	1.0000
C(43) – H(43A)	0.9800	C(43) – H(43B)	0.9800
C(43) – H(43C)	0.9800	C(44) – H(44A)	0.9800
C(44) – H(44B)	0.9800	C(44) – H(44C)	0.9800
C(45) – H(45A)	0.9800	C(45) – H(45B)	0.9800
C(45) – H(45C)	0.9800	C(46) – H(46A)	0.9800
C(46) – H(46B)	0.9800	C(46) – H(46C)	0.9800
C(51) – C(56)	1.386(3)	C(51) – C(52)	1.386(3)
C(52) – C(53)	1.388(4)	C(52) – H(52)	0.9500
C(53) – C(54)	1.375(4)	C(53) – H(53)	0.9500
C(54) – C(55)	1.384(4)	C(54) – H(54)	0.9500
C(55) – C(56)	1.383(3)	C(55) – H(55)	0.9500
C(56) – H(56)	0.9500	C – C(11)	1.526(3)
C(14) – C(15)	1.389(3)	C(14) – C(10)	1.533(3)

Continued on next page

Table S24. – continued from previous page

atom – atom	distance	atom – atom	distance
C(16) – C(15)	1.381(3)	C(16) – C(11)	1.401(3)
C(16) – H(16)	0.9500	C(15) – H(15)	0.9500
C(11) – C(12)	1.381(3)	C(10) – C(91)	1.500(7)
C(10) – C(93)	1.509(7)	C(10) – C(96)	1.553(7)
O(7) – C(72)	1.408(3)	O(7) – C(73)	1.415(3)
F(61) – C(6)	1.324(3)	O(61) – S(6)	1.4603(18)
F(62) – C(6)	1.318(3)	O(62) – S(6)	1.420(2)
F(63) – C(6)	1.321(3)	C(71) – C(72)	1.502(4)
C(71) – H(71A)	0.9800	C(71) – H(71B)	0.9800
C(71) – H(71C)	0.9800	C(72) – H(72A)	0.9900
C(72) – H(72B)	0.9900	C(73) – C(74)	1.495(4)
C(73) – H(73A)	0.9900	C(73) – H(73B)	0.9900
C(74) – H(74A)	0.9800	C(74) – H(74B)	0.9800
C(74) – H(74C)	0.9800	C(91) – H(91A)	0.9800
C(91) – H(91B)	0.9800	C(91) – H(91C)	0.9800
C(93) – H(93A)	0.9800	C(93) – H(93B)	0.9800
C(93) – H(93C)	0.9800	C(96) – H(96A)	0.9800
C(96) – H(96B)	0.9800	C(96) – H(96C)	0.9800
O(63) – S(6)	1.424(2)	C(6) – S(6)	1.818(3)

Table S25. Angles [°] for [{PC(sp^3)(SPh)P}^{tBu}PdOTf]·Et$_2$O (**8·Et$_2$O**).

atom – atom – atom	angle	atom – atom – atom	angle
C – Pd – O(61)	176.11(8)	C – Pd – P(1)	85.28(6)
O(61) – Pd – P(1)	95.34(5)	C – Pd – P(2)	84.26(6)
O(61) – Pd – P(2)	96.68(5)	P(1) – Pd – P(2)	154.14(2)
C(12) – P(1) – C(31)	105.69(12)	C(12) – P(1) – C(32)	109.35(11)
C(31) – P(1) – C(32)	105.39(12)	C(12) – P(1) – Pd	102.82(7)
C(31) – P(1) – Pd	113.42(9)	C(32) – P(1) – Pd	119.38(8)
C(51) – S – C	104.33(11)	C(22) – P(2) – C(42)	105.62(11)
C(22) – P(2) – C(41)	103.25(11)	C(42) – P(2) – C(41)	107.31(11)
C(22) – P(2) – Pd	95.43(8)	C(42) – P(2) – Pd	124.87(8)
C(41) – P(2) – Pd	116.56(8)	C(14) – C(13) – C(12)	121.9(2)
C(14) – C(13) – H(13)	119.1	C(12) – C(13) – H(13)	119.1
C(27) – C(20) – C(28)	109.0(2)	C(27) – C(20) – C(29)	108.8(2)
C(28) – C(20) – C(29)	109.6(2)	C(27) – C(20) – C(24)	111.8(2)
C(28) – C(20) – C(24)	108.0(2)	C(29) – C(20) – C(24)	109.7(2)
C(10) – C(19) – H(19A)	109.5	C(10) – C(19) – H(19B)	109.5
H(19A) – C(19) – H(19B)	109.5	C(10) – C(19) – H(19C)	109.5
H(19A) – C(19) – H(19C)	109.5	H(19B) – C(19) – H(19C)	109.5
C(10) – C(18) – H(18A)	109.5	C(10) – C(18) – H(18B)	109.5
H(18A) – C(18) – H(18B)	109.5	C(10) – C(18) – H(18C)	109.5
H(18A) – C(18) – H(18C)	109.5	H(18B) – C(18) – H(18C)	109.5
C(10) – C(17) – H(17A)	109.5	C(10) – C(17) – H(17B)	109.5
H(17A) – C(17) – H(17B)	109.5	C(10) – C(17) – H(17C)	109.5
H(17A) – C(17) – H(17C)	109.5	H(17B) – C(17) – H(17C)	109.5
C(23) – C(22) – C(21)	120.0(2)	C(23) – C(22) – P(2)	123.43(18)
C(21) – C(22) – P(2)	116.06(18)	C(26) – C(21) – C(22)	118.0(2)
C(26) – C(21) – C	123.2(2)	C(22) – C(21) – C	118.9(2)
C(24) – C(23) – C(22)	122.1(2)	C(24) – C(23) – H(23)	119.0
C(22) – C(23) – H(23)	119.0	C(25) – C(24) – C(23)	117.4(2)
C(25) – C(24) – C(20)	123.1(2)	C(23) – C(24) – C(20)	119.3(2)
C(24) – C(25) – C(26)	121.3(2)	C(24) – C(25) – H(25)	119.3
C(26) – C(25) – H(25)	119.3	C(21) – C(26) – C(25)	121.2(2)
C(21) – C(26) – H(26)	119.4	C(25) – C(26) – H(26)	119.4
C(20) – C(27) – H(27A)	109.5	C(20) – C(27) – H(27B)	109.5
H(27A) – C(27) – H(27B)	109.5	C(20) – C(27) – H(27C)	109.5
H(27A) – C(27) – H(27C)	109.5	H(27B) – C(27) – H(27C)	109.5
C(20) – C(28) – H(28A)	109.5	C(20) – C(28) – H(28B)	109.5
H(28A) – C(28) – H(28B)	109.5	C(20) – C(28) – H(28C)	109.5
H(28A) – C(28) – H(28C)	109.5	H(28B) – C(28) – H(28C)	109.5
C(20) – C(29) – H(29A)	109.5	C(20) – C(29) – H(29B)	109.5
H(29A) – C(29) – H(29B)	109.5	C(20) – C(29) – H(29C)	109.5
H(29A) – C(29) – H(29C)	109.5	H(29B) – C(29) – H(29C)	109.5
C(34) – C(31) – C(33)	111.4(2)	C(34) – C(31) – P(1)	111.2(2)
C(33) – C(31) – P(1)	109.89(19)	C(34) – C(31) – H(31)	108.1
C(33) – C(31) – H(31)	108.1	P(1) – C(31) – H(31)	108.1
C(36) – C(32) – C(35)	111.4(2)	C(36) – C(32) – P(1)	109.88(17)
C(35) – C(32) – P(1)	115.84(19)	C(36) – C(32) – H(32)	106.4
C(35) – C(32) – H(32)	106.4	P(1) – C(32) – H(32)	106.4
C(31) – C(33) – H(33A)	109.5	C(31) – C(33) – H(33B)	109.5
H(33A) – C(33) – H(33B)	109.5	C(31) – C(33) – H(33C)	109.5
H(33A) – C(33) – H(33C)	109.5	H(33B) – C(33) – H(33C)	109.5

Continued on next page

Table S25. – continued from previous page

atom – atom – atom	angle	atom – atom – atom	angle
C(31) – C(34) – H(34A)	109.5	C(31) – C(34) – H(34B)	109.5
H(34A) – C(34) – H(34B)	109.5	C(31) – C(34) – H(34C)	109.5
H(34A) – C(34) – H(34C)	109.5	H(34B) – C(34) – H(34C)	109.5
C(32) – C(35) – H(35A)	109.5	C(32) – C(35) – H(35B)	109.5
H(35A) – C(35) – H(35B)	109.5	C(32) – C(35) – H(35C)	109.5
H(35A) – C(35) – H(35C)	109.5	H(35B) – C(35) – H(35C)	109.5
C(32) – C(36) – H(36A)	109.5	C(32) – C(36) – H(36B)	109.5
H(36A) – C(36) – H(36B)	109.5	C(32) – C(36) – H(36C)	109.5
H(36A) – C(36) – H(36C)	109.5	H(36B) – C(36) – H(36C)	109.5
C(43) – C(41) – C(44)	111.0(2)	C(43) – C(41) – P(2)	109.30(16)
C(44) – C(41) – P(2)	112.74(17)	C(43) – C(41) – H(41)	107.9
C(44) – C(41) – H(41)	107.9	P(2) – C(41) – H(41)	107.9
C(45) – C(42) – C(46)	112.5(2)	C(45) – C(42) – P(2)	113.73(18)
C(46) – C(42) – P(2)	112.75(18)	C(45) – C(42) – H(42)	105.7
C(46) – C(42) – H(42)	105.7	P(2) – C(42) – H(42)	105.7
C(41) – C(43) – H(43A)	109.5	C(41) – C(43) – H(43B)	109.5
H(43A) – C(43) – H(43B)	109.5	C(41) – C(43) – H(43C)	109.5
H(43A) – C(43) – H(43C)	109.5	H(43B) – C(43) – H(43C)	109.5
C(41) – C(44) – H(44A)	109.5	C(41) – C(44) – H(44B)	109.5
H(44A) – C(44) – H(44B)	109.5	C(41) – C(44) – H(44C)	109.5
H(44A) – C(44) – H(44C)	109.5	H(44B) – C(44) – H(44C)	109.5
C(42) – C(45) – H(45A)	109.5	C(42) – C(45) – H(45B)	109.5
H(45A) – C(45) – H(45B)	109.5	C(42) – C(45) – H(45C)	109.5
H(45A) – C(45) – H(45C)	109.5	H(45B) – C(45) – H(45C)	109.5
C(42) – C(46) – H(46A)	109.5	C(42) – C(46) – H(46B)	109.5
H(46A) – C(46) – H(46B)	109.5	C(42) – C(46) – H(46C)	109.5
H(46A) – C(46) – H(46C)	109.5	H(46B) – C(46) – H(46C)	109.5
C(56) – C(51) – C(52)	119.7(2)	C(56) – C(51) – S	119.41(19)
C(52) – C(51) – S	120.8(2)	C(51) – C(52) – C(53)	119.6(2)
C(51) – C(52) – H(52)	120.2	C(53) – C(52) – H(52)	120.2
C(54) – C(53) – C(52)	120.3(2)	C(54) – C(53) – H(53)	119.8
C(52) – C(53) – H(53)	119.8	C(53) – C(54) – C(55)	120.3(2)
C(53) – C(54) – H(54)	119.8	C(55) – C(54) – H(54)	119.8
C(56) – C(55) – C(54)	119.5(3)	C(56) – C(55) – H(55)	120.2
C(54) – C(55) – H(55)	120.2	C(55) – C(56) – C(51)	120.5(3)
C(55) – C(56) – H(56)	119.8	C(51) – C(56) – H(56)	119.8
C(11) – C – C(21)	113.81(19)	C(11) – C – S	108.65(16)
C(21) – C – S	104.00(15)	C(11) – C – Pd	115.27(16)
C(21) – C – Pd	108.22(15)	S – C – Pd	106.03(11)
C(13) – C(14) – C(15)	116.9(2)	C(13) – C(14) – C(10)	121.6(2)
C(15) – C(14) – C(10)	121.4(2)	C(15) – C(16) – C(11)	120.8(2)
C(15) – C(16) – H(16)	119.6	C(11) – C(16) – H(16)	119.6
C(16) – C(15) – C(14)	121.9(2)	C(16) – C(15) – H(15)	119.0
C(14) – C(15) – H(15)	119.0	C(12) – C(11) – C(16)	117.7(2)
C(12) – C(11) – C	120.9(2)	C(16) – C(11) – C	121.3(2)
C(18) – C(10) – C(91)	68.7(4)	C(18) – C(10) – C(93)	134.9(3)
C(91) – C(10) – C(93)	112.5(4)	C(18) – C(10) – C(19)	110.0(4)
C(91) – C(10) – C(19)	142.3(4)	C(18) – C(10) – C(14)	111.2(3)
C(91) – C(10) – C(14)	107.7(3)	C(93) – C(10) – C(14)	110.8(3)
C(19) – C(10) – C(14)	107.5(3)	C(91) – C(10) – C(96)	107.5(4)
C(93) – C(10) – C(96)	106.6(4)	C(19) – C(10) – C(96)	71.1(4)

Continued on next page

Table S25. – continued from previous page

atom – atom – atom	angle	atom – atom – atom	angle
C(14) – C(10) – C(96)	111.7(3)	C(18) – C(10) – C(17)	108.6(4)
C(91) – C(10) – C(17)	45.2(3)	C(93) – C(10) – C(17)	69.6(4)
C(19) – C(10) – C(17)	107.9(4)	C(14) – C(10) – C(17)	111.6(3)
C(96) – C(10) – C(17)	134.6(3)	C(72) – O(7) – C(73)	110.9(2)
C(11) – C(12) – C(13)	120.5(2)	C(11) – C(12) – P(1)	114.35(16)
C(13) – C(12) – P(1)	125.15(19)	S(6) – O(61) – Pd	128.95(11)
C(72) – C(71) – H(71A)	109.5	C(72) – C(71) – H(71B)	109.5
H(71A) – C(71) – H(71B)	109.5	C(72) – C(71) – H(71C)	109.5
H(71A) – C(71) – H(71C)	109.5	H(71B) – C(71) – H(71C)	109.5
O(7) – C(72) – C(71)	110.1(2)	O(7) – C(72) – H(72A)	109.6
C(71) – C(72) – H(72A)	109.6	O(7) – C(72) – H(72B)	109.6
C(71) – C(72) – H(72B)	109.6	H(72A) – C(72) – H(72B)	108.2
O(7) – C(73) – C(74)	109.7(2)	O(7) – C(73) – H(73A)	109.7
C(74) – C(73) – H(73A)	109.7	O(7) – C(73) – H(73B)	109.7
C(74) – C(73) – H(73B)	109.7	H(73A) – C(73) – H(73B)	108.2
C(73) – C(74) – H(74A)	109.5	C(73) – C(74) – H(74B)	109.5
H(74A) – C(74) – H(74B)	109.5	C(73) – C(74) – H(74C)	109.5
H(74A) – C(74) – H(74C)	109.5	H(74B) – C(74) – H(74C)	109.5
C(10) – C(91) – H(91A)	109.5	C(10) – C(91) – H(91B)	109.5
C(10) – C(91) – H(91C)	109.5	C(10) – C(93) – H(93A)	109.5
C(10) – C(93) – H(93B)	109.5	C(10) – C(93) – H(93C)	109.5
C(10) – C(96) – H(96A)	109.5	C(10) – C(96) – H(96B)	109.5
C(10) – C(96) – H(96C)	109.5	F(62) – C(6) – F(63)	108.0(2)
F(62) – C(6) – F(61)	108.0(2)	F(63) – C(6) – F(61)	107.5(2)
F(62) – C(6) – S(6)	111.30(19)	F(63) – C(6) – S(6)	110.91(19)
F(61) – C(6) – S(6)	110.95(18)	O(62) – S(6) – O(63)	116.10(14)
O(62) – S(6) – O(61)	114.79(11)	O(63) – S(6) – O(61)	114.14(12)
O(62) – S(6) – C(6)	105.01(13)	O(63) – S(6) – C(6)	103.55(13)
O(61) – S(6) – C(6)	100.71(12)		

5.5 Crystal data for [{PC(sp^3)(SPh)P}^{tBu}PdSPh]·C_5H_{12} (9·C_5H_{12})

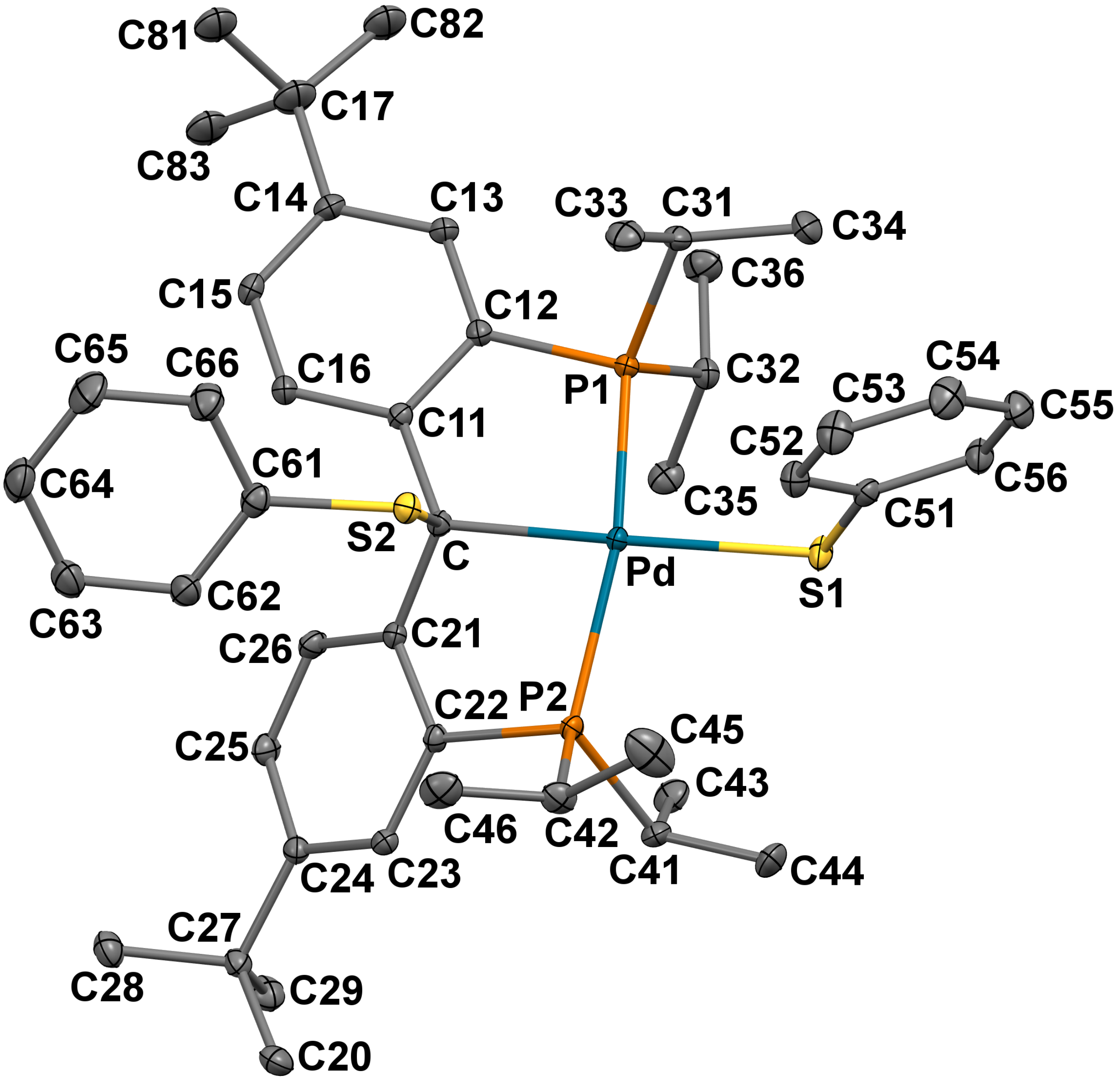

Figure S48. Thermal-ellipsoid representation of [{PC(sp^3)(SPh)P}^{tBu}PdSPh]·C_5H_{12} (**9·C_5H_{12}**) at 50% probability. Hydrogen atoms and the solvent were omitted for clarity.

Table S26. Crystal data and structure refinement for [{PC(sp^3)(SPh)P}^{tBu}PdSPh]·C_5H_{12} (**9·C_5H_{12}**).

Identification code:	pc36	
Empirical formula:	$C_{50}H_{74}P_2PdS_2$	
Formula weight:	907.55	
Temperature:	120(2) K	
Wavelength:	0.71073 Å	
Crystal system:	Monoclinic	
Space group:	$P2_1/n$	
Unit cell dimensions:	a = 11.297(3) Å	α = 90°
	b = 11.590(3) Å	β = 92.926(4)°
	c = 36.768(10) Å	γ = 90°
Volume:	4808(2) Å^3	
Z:	4	
Density (calculated):	1.254 g·cm^{-3}	
Absorption coefficient (μ):	0.571 mm^{-1}	
F(000):	1928	
Crystal size:	0.18 × 0.13 × 0.05 mm^3	
θ range for data collection:	1.84 to 28.81°	
Index ranges:	$-15 \leq h \leq 15$, $-15 \leq k \leq 15$, $-49 \leq l \leq 49$	
Reflections collected:	111329	
Independent reflections:	12309 [R_{int} = 0.0632]	
Completeness to θ = 25.00°:	100.0 %	
Absorption correction:	Semi-empirical from equivalents	
Max. and min. transmission:	0.9737 and 0.9052	
Refinement method:	Full-matrix least-squares on F^2	
Data / restraints / parameters:	12309 / 0 / 507	
Goodness-of-fit on F^2:	1.055	
Final R indices [I>2σ(I)]:	R_1 = 0.0392, wR_2 = 0.0754	
R indices (all data):	R_1 = 0.0557, wR_2 = 0.0807	
Largest diff. peak and hole:	1.121 and −0.773 e$^-$·Å^{-3}	

Table S27. Atomic coordinates and equivalent isotropic displacement parameters (Å^2) for [{PC(sp^3)(SPh)P}^{tBu}PdSPh]·C_5H_{12} (**9·C_5H_{12}**). U(eq) is defined as one third of the trace of the orthogonalized U_{ij} tensor

**atom**	**x**	**y**	**z**	**U(eq)**
Pd	0.66755(1)	0.33226(1)	0.65633(1)	0.013(1)
P(1)	0.60311(5)	0.17718(5)	0.62339(2)	0.015(1)
P(2)	0.67856(5)	0.50065(5)	0.69169(2)	0.015(1)
S(2)	0.62991(5)	0.51062(5)	0.60039(2)	0.018(1)
S(1)	0.80739(5)	0.21561(5)	0.69109(2)	0.020(1)
C	0.52947(18)	0.42195(18)	0.62720(6)	0.014(1)
C(81)	0.2603(14)	0.1856(13)	0.4810(4)	0.038(1)
C(82)	0.2746(14)	0.0154(13)	0.5212(4)	0.038(1)
C(83)	0.1085(14)	0.1602(14)	0.5274(4)	0.038(1)
C(11)	0.45293(18)	0.34964(18)	0.60063(6)	0.015(1)
C(13)	0.41212(19)	0.1660(2)	0.56986(6)	0.019(1)
C(12)	0.47787(19)	0.23220(19)	0.59562(6)	0.015(1)
C(14)	0.3179(2)	0.2125(2)	0.54849(6)	0.020(1)
C(15)	0.2953(2)	0.3298(2)	0.55301(6)	0.020(1)
C(16)	0.36096(19)	0.39710(19)	0.57800(6)	0.018(1)
C(20)	0.3832(2)	0.8244(2)	0.74525(7)	0.026(1)
C(17)	0.2437(3)	0.1355(3)	0.52208(8)	0.038(1)
C(21)	0.46384(18)	0.49475(19)	0.65410(6)	0.015(1)
C(22)	0.52960(18)	0.55551(19)	0.68147(6)	0.015(1)
C(23)	0.47674(19)	0.63820(18)	0.70291(6)	0.015(1)
C(24)	0.35476(19)	0.6583(2)	0.70017(6)	0.017(1)
C(25)	0.28803(19)	0.5872(2)	0.67633(6)	0.020(1)
C(26)	0.34027(19)	0.5081(2)	0.65364(6)	0.019(1)
C(27)	0.2936(2)	0.7492(2)	0.72323(6)	0.019(1)
C(29)	0.2145(2)	0.6876(2)	0.74974(7)	0.027(1)
C(28)	0.2175(2)	0.8283(2)	0.69814(7)	0.026(1)
C(18)	0.1847(3)	0.0398(3)	0.54402(10)	0.038(1)
C(19)	0.1480(3)	0.2012(3)	0.50109(11)	0.038(1)
C(10)	0.3250(3)	0.0780(3)	0.49550(10)	0.038(1)
C(31)	0.6946(2)	0.1043(2)	0.59014(6)	0.020(1)
C(34)	0.7847(2)	0.0192(2)	0.60713(7)	0.029(1)
C(33)	0.7547(2)	0.1963(2)	0.56757(7)	0.026(1)
C(32)	0.5481(2)	0.06348(19)	0.65326(6)	0.019(1)
C(36)	0.4858(2)	−0.0380(2)	0.63387(7)	0.028(1)
C(35)	0.4699(2)	0.1179(2)	0.68125(7)	0.024(1)
C(46)	0.7487(3)	0.7200(2)	0.66445(8)	0.031(1)
C(45)	0.9093(2)	0.5803(3)	0.68443(10)	0.043(1)
C(44)	0.7940(2)	0.4445(2)	0.75947(7)	0.031(1)
C(43)	0.5836(2)	0.3770(2)	0.74820(7)	0.027(1)
C(42)	0.7846(2)	0.6240(2)	0.69101(7)	0.023(1)
C(41)	0.6728(2)	0.4724(2)	0.74121(6)	0.020(1)
C(51)	0.94000(19)	0.2148(2)	0.66798(6)	0.018(1)
C(52)	0.9666(2)	0.2989(2)	0.64256(7)	0.022(1)
C(53)	1.0734(2)	0.2963(2)	0.62527(8)	0.030(1)
C(54)	1.1551(2)	0.2096(3)	0.63303(8)	0.031(1)
C(55)	1.1294(2)	0.1251(2)	0.65777(7)	0.029(1)
C(61)	0.5376(2)	0.6066(2)	0.57342(7)	0.021(1)
C(62)	0.4662(2)	0.6888(2)	0.58922(7)	0.025(1)

Continued on next page

Table S27. – continued from previous page

atom	x	y	x	U(eq)
C(63)	0.3918(2)	0.7582(2)	0.56731(8)	0.031(1)
C(64)	0.3908(3)	0.7490(2)	0.52976(8)	0.036(1)
C(65)	0.4652(3)	0.6715(3)	0.51387(8)	0.038(1)
C(66)	0.5379(2)	0.5993(2)	0.53561(7)	0.030(1)
C(71)	1.0305(5)	0.7891(5)	0.59853(14)	0.093(2)
C(72)	1.0855(4)	0.6757(6)	0.59781(14)	0.097(2)
C(73)	1.0469(4)	0.6008(4)	0.56676(15)	0.081(2)
C(74)	0.9260(4)	0.5569(4)	0.56704(12)	0.070(1)
C(75)	0.8881(4)	0.4721(4)	0.53574(14)	0.087(2)
C(56)	1.0231(2)	0.1271(2)	0.67515(7)	0.023(1)
H(81A)	0.2132	0.1391	0.4633	0.057
H(81B)	0.3441	0.1816	0.4754	0.057
H(81C)	0.2335	0.2660	0.4797	0.057
H(82A)	0.2204	−0.0250	0.5038	0.046
H(82B)	0.2685	−0.0180	0.5455	0.046
H(82C)	0.3561	0.0073	0.5136	0.046
H(83A)	0.0934	0.2432	0.5251	0.057
H(83B)	0.0879	0.1341	0.5516	0.057
H(83C)	0.0602	0.1187	0.5088	0.057
H(13)	0.4322	0.0871	0.5668	0.023
H(15)	0.2331	0.3649	0.5386	0.024
H(16)	0.3434	0.4770	0.5799	0.021
H(20A)	0.4356	0.8630	0.7286	0.039
H(20B)	0.4305	0.7758	0.7623	0.039
H(20C)	0.3407	0.8826	0.7589	0.039
H(23)	0.5249	0.6819	0.7198	0.019
H(25)	0.2041	0.5931	0.6756	0.024
H(26)	0.2915	0.4623	0.6375	0.022
H(29A)	0.1541	0.6424	0.7360	0.040
H(29B)	0.1757	0.7448	0.7647	0.040
H(29C)	0.2630	0.6361	0.7655	0.040
H(28A)	0.2678	0.8659	0.6807	0.039
H(28B)	0.1796	0.8871	0.7128	0.039
H(28C)	0.1565	0.7824	0.6849	0.039
H(18A)	0.1391	−0.0112	0.5273	0.057
H(18B)	0.1315	0.0749	0.5611	0.057
H(18C)	0.2459	−0.0049	0.5575	0.057
H(19A)	0.1839	0.2630	0.4871	0.057
H(19B)	0.0935	0.2348	0.5181	0.057
H(19C)	0.1043	0.1487	0.4844	0.057
H(10A)	0.2786	0.0250	0.4796	0.057
H(10B)	0.3873	0.0349	0.5092	0.057
H(10C)	0.3613	0.1372	0.4806	0.057
H(31)	0.6398	0.0597	0.5732	0.024
H(34A)	0.8293	−0.0165	0.5879	0.043
H(34B)	0.7431	−0.0408	0.6203	0.043
H(34C)	0.8395	0.0602	0.6241	0.043
H(33A)	0.7952	0.1591	0.5478	0.039
H(33B)	0.8124	0.2388	0.5832	0.039
H(33C)	0.6947	0.2499	0.5573	0.039
H(32)	0.6189	0.0311	0.6671	0.022

Continued on next page

Table S27. – continued from previous page

**atom**	**x**	**y**	**x**	**U(eq)**
H(36A)	0.4077	−0.0133	0.6240	0.043
H(36B)	0.4765	−0.1011	0.6512	0.043
H(36C)	0.5335	−0.0645	0.6140	0.043
H(35A)	0.4009	0.1542	0.6687	0.035
H(35B)	0.5154	0.1763	0.6952	0.035
H(35C)	0.4432	0.0581	0.6978	0.035
H(46A)	0.7457	0.6898	0.6395	0.047
H(46B)	0.8070	0.7826	0.6666	0.047
H(46C)	0.6705	0.7496	0.6701	0.047
H(45A)	0.9133	0.5561	0.6590	0.064
H(45B)	0.9279	0.5145	0.7005	0.064
H(45C)	0.9668	0.6422	0.6896	0.064
H(44A)	0.8276	0.3771	0.7477	0.046
H(44B)	0.7850	0.4278	0.7853	0.046
H(44C)	0.8470	0.5107	0.7571	0.046
H(43A)	0.5077	0.3951	0.7354	0.041
H(43B)	0.5725	0.3713	0.7744	0.041
H(43C)	0.6135	0.3034	0.7393	0.041
H(42)	0.7877	0.6587	0.7159	0.028
H(41)	0.6436	0.5444	0.7528	0.024
H(52)	0.9112	0.3587	0.6370	0.026
H(53)	1.0902	0.3544	0.6080	0.036
H(54)	1.2283	0.2084	0.6214	0.037
H(55)	1.1849	0.0650	0.6630	0.034
H(62)	0.4684	0.6974	0.6149	0.030
H(63)	0.3412	0.8122	0.5782	0.038
H(64)	0.3391	0.7961	0.5150	0.043
H(65)	0.4669	0.6672	0.4881	0.046
H(66)	0.5878	0.5450	0.5246	0.036
H(71A)	1.0488	0.8318	0.5765	0.139
H(71B)	1.0612	0.8314	0.6201	0.139
H(71C)	0.9444	0.7804	0.5995	0.139
H(72A)	1.0689	0.6351	0.6207	0.116
H(72B)	1.1724	0.6862	0.5975	0.116
H(73A)	1.0550	0.6448	0.5440	0.097
H(73B)	1.1015	0.5342	0.5662	0.097
H(74A)	0.8711	0.6235	0.5660	0.084
H(74B)	0.9163	0.5173	0.5905	0.084
H(75A)	0.8951	0.5106	0.5122	0.130
H(75B)	0.8056	0.4484	0.5383	0.130
H(75C)	0.9395	0.4040	0.5370	0.130
H(56)	1.0066	0.0682	0.6921	0.027

Table S28. Anisotropic displacement parameters (Å^2) for [{PC(sp^3)(SPh)P}^{tBu}PdSPh]·C_5H_{12} (**9·C_5H_{12}**). The anisotropic displacement factor exponent takes the form: $-2\pi^2[h^2a^{*2}U_{11} + ... + 2hka^*b^*U_{12}]$.

atom	U_{11}	U_{22}	U_{33}	U_{23}	U_{13}	U_{12}
Pd	0.0116(1)	0.0139(1)	0.0123(1)	−0.0006(1)	−0.0019(1)	0.0021(1)
P(1)	0.0163(3)	0.0136(3)	0.0134(3)	−0.0018(2)	−0.0019(2)	0.0028(2)
P(2)	0.0124(2)	0.0157(3)	0.0155(3)	−0.0023(2)	−0.0031(2)	0.0012(2)
S(2)	0.0179(3)	0.0185(3)	0.0177(3)	0.0014(2)	0.0006(2)	0.0001(2)
S(1)	0.0160(3)	0.0251(3)	0.0178(3)	0.0055(2)	−0.0003(2)	0.0059(2)
C	0.0130(10)	0.0146(10)	0.0147(11)	0.0002(8)	−0.0020(8)	0.0008(8)
C(81)	0.0439(10)	0.0323(9)	0.0364(10)	−0.0104(7)	−0.0165(8)	−0.0039(7)
C(82)	0.0439(10)	0.0323(9)	0.0364(10)	−0.0104(7)	−0.0165(8)	−0.0039(7)
C(83)	0.0439(10)	0.0323(9)	0.0364(10)	−0.0104(7)	−0.0165(8)	−0.0039(7)
C(11)	0.0146(10)	0.0162(11)	0.0135(10)	−0.0007(8)	−0.0009(8)	0.0003(8)
C(13)	0.0220(11)	0.0171(10)	0.0168(11)	−0.0033(9)	−0.0029(8)	0.0019(9)
C(12)	0.0161(10)	0.0159(10)	0.0143(11)	−0.0002(8)	0.0001(8)	0.0027(8)
C(14)	0.0230(11)	0.0217(11)	0.0141(11)	−0.0034(9)	−0.0034(9)	0.0004(9)
C(15)	0.0207(11)	0.0223(11)	0.0164(11)	0.0013(10)	−0.0053(8)	0.0038(10)
C(16)	0.0184(10)	0.0165(11)	0.0172(11)	−0.0003(9)	−0.0022(9)	0.0037(8)
C(20)	0.0274(12)	0.0241(12)	0.0264(13)	−0.0080(11)	0.0016(10)	0.0064(10)
C(17)	0.0439(10)	0.0323(9)	0.0364(10)	−0.0104(7)	−0.0165(8)	−0.0039(7)
C(21)	0.0160(10)	0.0141(10)	0.0135(10)	0.0007(8)	−0.0022(8)	0.0022(8)
C(22)	0.0119(9)	0.0161(10)	0.0161(11)	0.0006(8)	−0.0016(8)	0.0005(8)
C(23)	0.0163(10)	0.0156(10)	0.0143(11)	−0.0012(8)	−0.0018(8)	0.0004(8)
C(24)	0.0178(10)	0.0180(11)	0.0152(10)	0.0010(9)	0.0003(8)	0.0038(9)
C(25)	0.0126(10)	0.0254(12)	0.0211(12)	−0.0019(10)	−0.0008(9)	0.0028(9)
C(26)	0.0154(10)	0.0223(11)	0.0175(11)	−0.0034(9)	−0.0034(8)	−0.0004(9)
C(27)	0.0186(11)	0.0210(11)	0.0174(12)	−0.0002(9)	0.0017(9)	0.0053(9)
C(29)	0.0288(13)	0.0277(14)	0.0250(13)	0.0010(10)	0.0073(10)	0.0037(10)
C(28)	0.0229(11)	0.0250(12)	0.0298(13)	0.0011(11)	0.0032(10)	0.0076(10)
C(18)	0.0439(10)	0.0323(9)	0.0364(10)	−0.0104(7)	−0.0165(8)	−0.0039(7)
C(19)	0.0439(10)	0.0323(9)	0.0364(10)	−0.0104(7)	−0.0165(8)	−0.0039(7)
C(10)	0.0439(10)	0.0323(9)	0.0364(10)	−0.0104(7)	−0.0165(8)	−0.0039(7)
C(31)	0.0204(11)	0.0221(12)	0.0163(11)	−0.0067(9)	−0.0004(9)	0.0047(9)
C(34)	0.0282(13)	0.0286(14)	0.0283(14)	−0.0065(11)	−0.0031(11)	0.0124(11)
C(33)	0.0274(13)	0.0322(14)	0.0199(13)	−0.0034(10)	0.0062(10)	0.0021(10)
C(32)	0.0199(11)	0.0173(11)	0.0187(12)	0.0017(9)	−0.0011(9)	0.0022(9)
C(36)	0.0374(14)	0.0205(12)	0.0271(14)	0.0002(10)	−0.0005(11)	−0.0063(11)
C(35)	0.0229(12)	0.0268(13)	0.0214(13)	0.0014(10)	0.0032(10)	−0.0026(10)
C(46)	0.0360(15)	0.0251(13)	0.0329(15)	0.0000(11)	−0.0017(12)	−0.0110(11)
C(45)	0.0183(13)	0.0355(16)	0.075(2)	−0.0116(16)	0.0072(14)	−0.0071(11)
C(44)	0.0313(14)	0.0332(14)	0.0255(14)	−0.0043(11)	−0.0148(11)	0.0060(11)
C(43)	0.0327(14)	0.0314(13)	0.0173(12)	0.0033(10)	0.0007(10)	0.0014(11)
C(42)	0.0176(11)	0.0222(12)	0.0302(14)	−0.0051(10)	−0.0006(10)	−0.0040(9)
C(41)	0.0244(12)	0.0227(12)	0.0136(11)	−0.0042(9)	−0.0038(9)	0.0043(9)
C(51)	0.0160(10)	0.0198(11)	0.0169(11)	−0.0026(9)	−0.0039(8)	0.0007(9)
C(52)	0.0187(11)	0.0227(12)	0.0246(13)	0.0030(9)	−0.0013(9)	0.0015(9)
C(53)	0.0249(13)	0.0365(15)	0.0295(14)	0.0086(11)	0.0046(11)	−0.0001(11)
C(54)	0.0164(11)	0.0463(16)	0.0306(15)	0.0018(12)	0.0054(10)	0.0031(11)
C(55)	0.0219(12)	0.0344(14)	0.0293(14)	−0.0010(11)	−0.0001(10)	0.0106(10)
C(61)	0.0226(11)	0.0197(12)	0.0209(12)	0.0039(9)	−0.0012(9)	−0.0022(9)
C(62)	0.0326(13)	0.0184(12)	0.0236(13)	0.0002(9)	−0.0047(10)	0.0002(10)
C(63)	0.0352(15)	0.0206(12)	0.0377(16)	0.0000(11)	−0.0049(12)	0.0045(11)

Continued on next page

Table S28. – continued from previous page

atom	U_{11}	U_{22}	U_{33}	U_{23}	U_{13}	U_{12}
C(64)	0.0415(16)	0.0302(14)	0.0338(16)	0.0094(12)	−0.0115(13)	0.0042(12)
C(65)	0.0478(17)	0.0445(17)	0.0216(14)	0.0097(13)	−0.0020(12)	0.0039(15)
C(66)	0.0323(14)	0.0358(15)	0.0225(14)	0.0047(11)	0.0028(11)	0.0044(12)
C(71)	0.092(4)	0.114(5)	0.069(3)	0.015(3)	−0.016(3)	−0.004(3)
C(72)	0.043(2)	0.174(6)	0.073(3)	0.052(4)	0.004(2)	0.011(3)
C(73)	0.060(3)	0.083(3)	0.101(4)	0.040(3)	0.022(3)	0.014(2)
C(74)	0.060(2)	0.085(3)	0.065(3)	0.024(2)	0.009(2)	0.029(2)
C(75)	0.103(4)	0.051(2)	0.114(4)	0.023(3)	0.074(3)	0.018(2)
C(56)	0.0211(11)	0.0247(12)	0.0223(12)	0.0038(10)	−0.0006(9)	0.0048(9)

Table S29. Distances [Å] for [{PC(sp^3)(SPh)P}^{tBu}PdSPh]·C_5H_{12} (**9·C_5H_{12}**).

atom – atom	distance	atom – atom	distance
Pd – C	2.119(2)	Pd – P(1)	2.2666(7)
Pd – P(2)	2.3449(8)	Pd – S(1)	2.3980(7)
P(1) – C(12)	1.818(2)	P(1) – C(32)	1.844(2)
P(1) – C(31)	1.846(2)	P(2) – C(22)	1.820(2)
P(2) – C(41)	1.854(2)	P(2) – C(42)	1.866(2)
S(2) – C(61)	1.789(2)	S(2) – C	1.852(2)
S(1) – C(51)	1.760(2)	C – C(21)	1.521(3)
C – C(11)	1.523(3)	C(81) – C(17)	1.638(16)
C(81) – H(81A)	0.9800	C(81) – H(81B)	0.9800
C(81) – H(81C)	0.9800	C(82) – C(17)	1.436(15)
C(82) – H(82A)	0.9800	C(82) – H(82B)	0.9800
C(82) – H(82C)	0.9800	C(83) – C(17)	1.575(16)
C(83) – H(83A)	0.9800	C(83) – H(83B)	0.9800
C(83) – H(83C)	0.9800	C(11) – C(12)	1.404(3)
C(11) – C(16)	1.409(3)	C(13) – C(14)	1.399(3)
C(13) – C(12)	1.402(3)	C(13) – H(13)	0.9500
C(14) – C(15)	1.395(3)	C(14) – C(17)	1.536(3)
C(15) – C(16)	1.390(3)	C(15) – H(15)	0.9500
C(16) – H(16)	0.9500	C(20) – C(27)	1.536(3)
C(20) – H(20A)	0.9800	C(20) – H(20B)	0.9800
C(20) – H(20C)	0.9800	C(17) – C(19)	1.503(5)
C(17) – C(10)	1.528(5)	C(17) – C(18)	1.543(5)
C(21) – C(26)	1.404(3)	C(21) – C(22)	1.409(3)
C(22) – C(23)	1.395(3)	C(23) – C(24)	1.396(3)
C(23) – H(23)	0.9500	C(24) – C(25)	1.396(3)
C(24) – C(27)	1.538(3)	C(25) – C(26)	1.391(3)
C(25) – H(25)	0.9500	C(26) – H(26)	0.9500
C(27) – C(29)	1.532(3)	C(27) – C(28)	1.532(3)
C(29) – H(29A)	0.9800	C(29) – H(29B)	0.9800
C(29) – H(29C)	0.9800	C(28) – H(28A)	0.9800
C(28) – H(28B)	0.9800	C(28) – H(28C)	0.9800
C(18) – H(18A)	0.9800	C(18) – H(18B)	0.9800
C(18) – H(18C)	0.9800	C(19) – H(19A)	0.9800
C(19) – H(19B)	0.9800	C(19) – H(19C)	0.9800
C(10) – H(10A)	0.9800	C(10) – H(10B)	0.9800
C(10) – H(10C)	0.9800	C(31) – C(34)	1.528(3)
C(31) – C(33)	1.531(3)	C(31) – H(31)	1.0000
C(34) – H(34A)	0.9800	C(34) – H(34B)	0.9800
C(34) – H(34C)	0.9800	C(33) – H(33A)	0.9800
C(33) – H(33B)	0.9800	C(33) – H(33C)	0.9800
C(32) – C(35)	1.526(3)	C(32) – C(36)	1.528(3)
C(32) – H(32)	1.0000	C(36) – H(36A)	0.9800
C(36) – H(36B)	0.9800	C(36) – H(36C)	0.9800
C(35) – H(35A)	0.9800	C(35) – H(35B)	0.9800
C(35) – H(35C)	0.9800	C(46) – C(42)	1.522(4)
C(46) – H(46A)	0.9800	C(46) – H(46B)	0.9800
C(46) – H(46C)	0.9800	C(45) – C(42)	1.528(4)
C(45) – H(45A)	0.9800	C(45) – H(45B)	0.9800
C(45) – H(45C)	0.9800	C(44) – C(41)	1.528(3)
C(44) – H(44A)	0.9800	C(44) – H(44B)	0.9800

Continued on next page

Table S29. – continued from previous page

**atom – atom**	**distance**	**atom – atom**	**distance**
C(44) – H(44C)	0.9800	C(43) – C(41)	1.526(4)
C(43) – H(43A)	0.9800	C(43) – H(43B)	0.9800
C(43) – H(43C)	0.9800	C(42) – H(42)	1.0000
C(41) – H(41)	1.0000	C(51) – C(52)	1.393(3)
C(51) – C(56)	1.400(3)	C(52) – C(53)	1.392(3)
C(52) – H(52)	0.9500	C(53) – C(54)	1.385(4)
C(53) – H(53)	0.9500	C(54) – C(55)	1.378(4)
C(54) – H(54)	0.9500	C(55) – C(56)	1.389(3)
C(55) – H(55)	0.9500	C(61) – C(66)	1.393(4)
C(61) – C(62)	1.394(3)	C(62) – C(63)	1.390(3)
C(62) – H(62)	0.9500	C(63) – C(64)	1.384(4)
C(63) – H(63)	0.9500	C(64) – C(65)	1.381(4)
C(64) – H(64)	0.9500	C(65) – C(66)	1.395(4)
C(65) – H(65)	0.9500	C(66) – H(66)	0.9500
C(71) – C(72)	1.454(7)	C(71) – H(71A)	0.9800
C(71) – H(71B)	0.9800	C(71) – H(71C)	0.9800
C(72) – C(73)	1.482(7)	C(72) – H(72A)	0.9900
C(72) – H(72B)	0.9900	C(73) – C(74)	1.457(6)
C(73) – H(73A)	0.9900	C(73) – H(73B)	0.9900
C(74) – C(75)	1.557(7)	C(74) – H(74A)	0.9900
C(74) – H(74B)	0.9900	C(75) – H(75A)	0.9800
C(75) – H(75B)	0.9800	C(75) – H(75C)	0.9800
C(56) – H(56)	0.9500		

Table S30. Angles [°] for [{PC(sp^3)(SPh)P}^{tBu}PdSPh]·C_5H_{12} (**9·C_5H_{12}**).

atom – atom – atom	angle	atom – atom – atom	angle
C – Pd – P(1)	84.89(6)	C – Pd – P(2)	83.61(6)
P(1) – Pd – P(2)	164.32(2)	C – Pd – S(1)	173.64(6)
P(1) – Pd – S(1)	91.29(3)	P(2) – Pd – S(1)	99.15(3)
C(12) – P(1) – C(32)	107.99(11)	C(12) – P(1) – C(31)	103.58(10)
C(32) – P(1) – C(31)	106.54(11)	C(12) – P(1) – Pd	103.98(7)
C(32) – P(1) – Pd	110.94(8)	C(31) – P(1) – Pd	122.78(8)
C(22) – P(2) – C(41)	100.72(10)	C(22) – P(2) – C(42)	108.48(11)
C(41) – P(2) – C(42)	101.73(11)	C(22) – P(2) – Pd	98.86(7)
C(41) – P(2) – Pd	113.19(8)	C(42) – P(2) – Pd	130.25(8)
C(61) – S(2) – C	106.53(10)	C(51) – S(1) – Pd	107.43(8)
C(21) – C – C(11)	116.28(18)	C(21) – C – S(2)	111.75(15)
C(11) – C – S(2)	107.88(15)	C(21) – C – Pd	108.31(14)
C(11) – C – Pd	115.69(14)	S(2) – C – Pd	94.94(9)
C(17) – C(81) – H(81A)	109.5	C(17) – C(81) – H(81B)	109.5
H(81A) – C(81) – H(81B)	109.5	C(17) – C(81) – H(81C)	109.5
H(81A) – C(81) – H(81C)	109.5	H(81B) – C(81) – H(81C)	109.5
C(17) – C(82) – H(82A)	109.5	C(17) – C(82) – H(82B)	109.5
H(82A) – C(82) – H(82B)	109.5	C(17) – C(82) – H(82C)	109.5
H(82A) – C(82) – H(82C)	109.5	H(82B) – C(82) – H(82C)	109.5
C(17) – C(83) – H(83A)	109.5	C(17) – C(83) – H(83B)	109.5
H(83A) – C(83) – H(83B)	109.5	C(17) – C(83) – H(83C)	109.5
H(83A) – C(83) – H(83C)	109.5	H(83B) – C(83) – H(83C)	109.5
C(12) – C(11) – C(16)	116.6(2)	C(12) – C(11) – C	120.44(19)
C(16) – C(11) – C	122.78(19)	C(14) – C(13) – C(12)	122.0(2)
C(14) – C(13) – H(13)	119.0	C(12) – C(13) – H(13)	119.0
C(13) – C(12) – C(11)	121.1(2)	C(13) – C(12) – P(1)	123.91(17)
C(11) – C(12) – P(1)	114.97(16)	C(15) – C(14) – C(13)	116.6(2)
C(15) – C(14) – C(17)	123.0(2)	C(13) – C(14) – C(17)	120.4(2)
C(16) – C(15) – C(14)	122.1(2)	C(16) – C(15) – H(15)	119.0
C(14) – C(15) – H(15)	119.0	C(15) – C(16) – C(11)	121.6(2)
C(15) – C(16) – H(16)	119.2	C(11) – C(16) – H(16)	119.2
C(27) – C(20) – H(20A)	109.5	C(27) – C(20) – H(20B)	109.5
H(20A) – C(20) – H(20B)	109.5	C(27) – C(20) – H(20C)	109.5
H(20A) – C(20) – H(20C)	109.5	H(20B) – C(20) – H(20C)	109.5
C(82) – C(17) – C(19)	130.5(6)	C(82) – C(17) – C(10)	53.6(7)
C(19) – C(17) – C(10)	109.4(3)	C(82) – C(17) – C(14)	116.9(6)
C(19) – C(17) – C(14)	112.7(2)	C(10) – C(17) – C(14)	109.4(2)
C(82) – C(17) – C(18)	55.1(7)	C(19) – C(17) – C(18)	108.3(3)
C(10) – C(17) – C(18)	108.1(3)	C(14) – C(17) – C(18)	108.9(2)
C(82) – C(17) – C(83)	114.7(9)	C(19) – C(17) – C(83)	45.2(6)
C(10) – C(17) – C(83)	140.9(6)	C(14) – C(17) – C(83)	108.5(6)
C(18) – C(17) – C(83)	67.6(7)	C(82) – C(17) – C(81)	106.4(9)
C(19) – C(17) – C(81)	57.3(6)	C(10) – C(17) – C(81)	57.6(6)
C(14) – C(17) – C(81)	107.0(5)	C(18) – C(17) – C(81)	144.1(6)
C(83) – C(17) – C(81)	102.0(8)	C(26) – C(21) – C(22)	116.2(2)
C(26) – C(21) – C	124.79(19)	C(22) – C(21) – C	119.01(18)
C(23) – C(22) – C(21)	121.46(19)	C(23) – C(22) – P(2)	122.79(16)
C(21) – C(22) – P(2)	114.91(16)	C(22) – C(23) – C(24)	121.6(2)
C(22) – C(23) – H(23)	119.2	C(24) – C(23) – H(23)	119.2
C(25) – C(24) – C(23)	116.5(2)	C(25) – C(24) – C(27)	120.45(19)

Continued on next page

Table S30. – continued from previous page

atom – atom – atom	angle	atom – atom – atom	angle
C(23) – C(24) – C(27)	123.0(2)	C(26) – C(25) – C(24)	122.3(2)
C(26) – C(25) – H(25)	118.9	C(24) – C(25) – H(25)	118.9
C(25) – C(26) – C(21)	121.2(2)	C(25) – C(26) – H(26)	119.4
C(21) – C(26) – H(26)	119.4	C(29) – C(27) – C(28)	109.54(19)
C(29) – C(27) – C(20)	108.6(2)	C(28) – C(27) – C(20)	108.2(2)
C(29) – C(27) – C(24)	108.92(19)	C(28) – C(27) – C(24)	109.38(19)
C(20) – C(27) – C(24)	112.12(19)	C(27) – C(29) – H(29A)	109.5
C(27) – C(29) – H(29B)	109.5	H(29A) – C(29) – H(29B)	109.5
C(27) – C(29) – H(29C)	109.5	H(29A) – C(29) – H(29C)	109.5
H(29B) – C(29) – H(29C)	109.5	C(27) – C(28) – H(28A)	109.5
C(27) – C(28) – H(28B)	109.5	H(28A) – C(28) – H(28B)	109.5
C(27) – C(28) – H(28C)	109.5	H(28A) – C(28) – H(28C)	109.5
H(28B) – C(28) – H(28C)	109.5	C(17) – C(18) – H(18A)	109.5
C(17) – C(18) – H(18B)	109.5	C(17) – C(18) – H(18C)	109.5
C(17) – C(19) – H(19A)	109.5	C(17) – C(19) – H(19B)	109.5
C(17) – C(19) – H(19C)	109.5	C(17) – C(10) – H(10A)	109.5
C(17) – C(10) – H(10B)	109.5	C(17) – C(10) – H(10C)	109.5
C(34) – C(31) – C(33)	111.6(2)	C(34) – C(31) – P(1)	114.11(17)
C(33) – C(31) – P(1)	108.58(16)	C(34) – C(31) – H(31)	107.4
C(33) – C(31) – H(31)	107.4	P(1) – C(31) – H(31)	107.4
C(31) – C(34) – H(34A)	109.5	C(31) – C(34) – H(34B)	109.5
H(34A) – C(34) – H(34B)	109.5	C(31) – C(34) – H(34C)	109.5
H(34A) – C(34) – H(34C)	109.5	H(34B) – C(34) – H(34C)	109.5
C(31) – C(33) – H(33A)	109.5	C(31) – C(33) – H(33B)	109.5
H(33A) – C(33) – H(33B)	109.5	C(31) – C(33) – H(33C)	109.5
H(33A) – C(33) – H(33C)	109.5	H(33B) – C(33) – H(33C)	109.5
C(35) – C(32) – C(36)	111.4(2)	C(35) – C(32) – P(1)	109.31(16)
C(36) – C(32) – P(1)	115.67(17)	C(35) – C(32) – H(32)	106.7
C(36) – C(32) – H(32)	106.7	P(1) – C(32) – H(32)	106.7
C(32) – C(36) – H(36A)	109.5	C(32) – C(36) – H(36B)	109.5
H(36A) – C(36) – H(36B)	109.5	C(32) – C(36) – H(36C)	109.5
H(36A) – C(36) – H(36C)	109.5	H(36B) – C(36) – H(36C)	109.5
C(32) – C(35) – H(35A)	109.5	C(32) – C(35) – H(35B)	109.5
H(35A) – C(35) – H(35B)	109.5	C(32) – C(35) – H(35C)	109.5
H(35A) – C(35) – H(35C)	109.5	H(35B) – C(35) – H(35C)	109.5
C(42) – C(46) – H(46A)	109.5	C(42) – C(46) – H(46B)	109.5
H(46A) – C(46) – H(46B)	109.5	C(42) – C(46) – H(46C)	109.5
H(46A) – C(46) – H(46C)	109.5	H(46B) – C(46) – H(46C)	109.5
C(42) – C(45) – H(45A)	109.5	C(42) – C(45) – H(45B)	109.5
H(45A) – C(45) – H(45B)	109.5	C(42) – C(45) – H(45C)	109.5
H(45A) – C(45) – H(45C)	109.5	H(45B) – C(45) – H(45C)	109.5
C(41) – C(44) – H(44A)	109.5	C(41) – C(44) – H(44B)	109.5
H(44A) – C(44) – H(44B)	109.5	C(41) – C(44) – H(44C)	109.5
H(44A) – C(44) – H(44C)	109.5	H(44B) – C(44) – H(44C)	109.5
C(41) – C(43) – H(43A)	109.5	C(41) – C(43) – H(43B)	109.5
H(43A) – C(43) – H(43B)	109.5	C(41) – C(43) – H(43C)	109.5
H(43A) – C(43) – H(43C)	109.5	H(43B) – C(43) – H(43C)	109.5
C(46) – C(42) – C(45)	111.0(2)	C(46) – C(42) – P(2)	114.79(17)
C(45) – C(42) – P(2)	110.24(18)	C(46) – C(42) – H(42)	106.8
C(45) – C(42) – H(42)	106.8	P(2) – C(42) – H(42)	106.8
C(43) – C(41) – C(44)	110.9(2)	C(43) – C(41) – P(2)	110.48(16)

Continued on next page

Table S30. – continued from previous page

atom – atom – atom	angle	atom – atom – atom	angle
C(44) – C(41) – P(2)	113.15(18)	C(43) – C(41) – H(41)	107.3
C(44) – C(41) – H(41)	107.3	P(2) – C(41) – H(41)	107.3
C(52) – C(51) – C(56)	117.9(2)	C(52) – C(51) – S(1)	122.58(18)
C(56) – C(51) – S(1)	119.49(18)	C(53) – C(52) – C(51)	120.9(2)
C(53) – C(52) – H(52)	119.6	C(51) – C(52) – H(52)	119.6
C(54) – C(53) – C(52)	120.4(2)	C(54) – C(53) – H(53)	119.8
C(52) – C(53) – H(53)	119.8	C(55) – C(54) – C(53)	119.4(2)
C(55) – C(54) – H(54)	120.3	C(53) – C(54) – H(54)	120.3
C(54) – C(55) – C(56)	120.5(2)	C(54) – C(55) – H(55)	119.7
C(56) – C(55) – H(55)	119.7	C(66) – C(61) – C(62)	119.2(2)
C(66) – C(61) – S(2)	119.00(19)	C(62) – C(61) – S(2)	121.81(19)
C(63) – C(62) – C(61)	120.0(2)	C(63) – C(62) – H(62)	120.0
C(61) – C(62) – H(62)	120.0	C(64) – C(63) – C(62)	120.5(3)
C(64) – C(63) – H(63)	119.8	C(62) – C(63) – H(63)	119.8
C(65) – C(64) – C(63)	119.9(3)	C(65) – C(64) – H(64)	120.1
C(63) – C(64) – H(64)	120.1	C(64) – C(65) – C(66)	120.1(3)
C(64) – C(65) – H(65)	120.0	C(66) – C(65) – H(65)	120.0
C(61) – C(66) – C(65)	120.3(3)	C(61) – C(66) – H(66)	119.8
C(65) – C(66) – H(66)	119.8	C(72) – C(71) – H(71A)	109.5
C(72) – C(71) – H(71B)	109.5	H(71A) – C(71) – H(71B)	109.5
C(72) – C(71) – H(71C)	109.5	H(71A) – C(71) – H(71C)	109.5
H(71B) – C(71) – H(71C)	109.5	C(71) – C(72) – C(73)	115.7(4)
C(71) – C(72) – H(72A)	108.3	C(73) – C(72) – H(72A)	108.3
C(71) – C(72) – H(72B)	108.3	C(73) – C(72) – H(72B)	108.3
H(72A) – C(72) – H(72B)	107.4	C(74) – C(73) – C(72)	116.0(4)
C(74) – C(73) – H(73A)	108.3	C(72) – C(73) – H(73A)	108.3
C(74) – C(73) – H(73B)	108.3	C(72) – C(73) – H(73B)	108.3
H(73A) – C(73) – H(73B)	107.4	C(73) – C(74) – C(75)	116.0(4)
C(73) – C(74) – H(74A)	108.3	C(75) – C(74) – H(74A)	108.3
C(73) – C(74) – H(74B)	108.3	C(75) – C(74) – H(74B)	108.3
H(74A) – C(74) – H(74B)	107.4	C(74) – C(75) – H(75A)	109.5
C(74) – C(75) – H(75B)	109.5	H(75A) – C(75) – H(75B)	109.5
C(74) – C(75) – H(75C)	109.5	H(75A) – C(75) – H(75C)	109.5
H(75B) – C(75) – H(75C)	109.5	C(55) – C(56) – C(51)	120.9(2)
C(55) – C(56) – H(56)	119.6	C(51) – C(56) – H(56)	119.6

5.6 Crystal data for [{PC(sp^3)(NH^{p}Tol)P}^{tBu}PdI] (10)

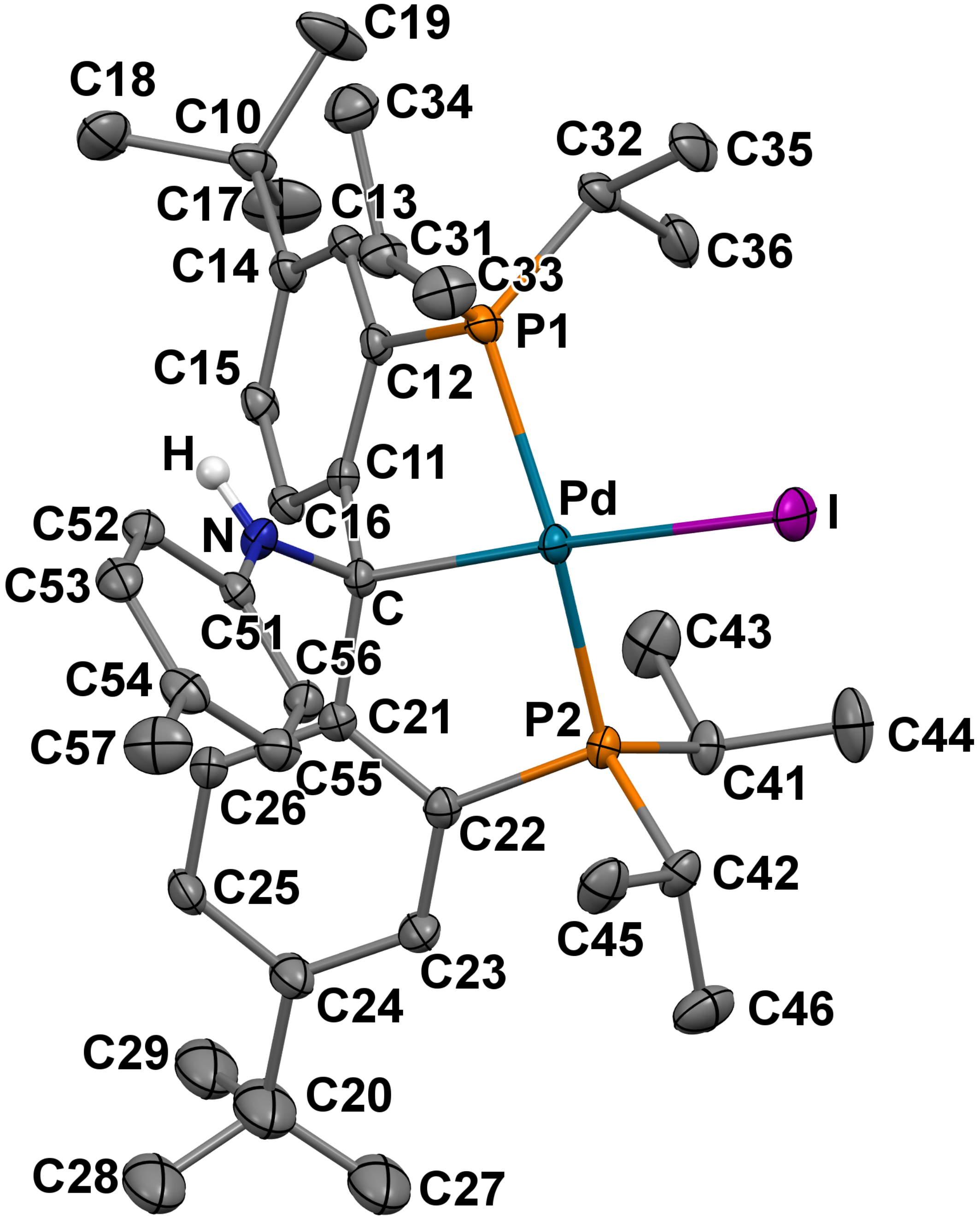

Figure S49. Thermal-ellipsoid representation of [{PC(sp^3)(NH^{p}Tol)P}^{tBu}PdI] (**10**) at 50% probability. Most hydrogen atoms were omitted for clarity.

Table S31. Crystal data and structure refinement for [{PC(sp^3)($NH^{p}Tol$)P}^{tBu}PdI] (**10**).

Identification code:	pc32	
Empirical formula:	$C_{40}H_{60}INP_2Pd$	
Formula weight:	850.13	
Temperature:	120(2) K	
Wavelength:	0.71073 Å	
Crystal system:	Monoclinic	
Space group:	$P2_1/n$	
Unit cell dimensions:	a = 17.0891(17) Å	α = 90°
	b = 14.0140(14) Å	β = 100.081(3)°
	c = 18.4206(18) Å	γ = 90°
Volume:	4343.4(7) $Å^3$	
Z:	4	
Density (calculated):	1.300 $g{\cdot}cm^{-3}$	
Absorption coefficient (μ):	1.237 mm^{-1}	
F(000):	1744	
Crystal size:	0.18 × 0.13 × 0.10 mm^3	
θ range for data collection:	1.50 to 25.00°	
Index ranges:	$-13 \leq h \leq 20$, $-16 \leq k \leq 16$, $-21 \leq l \leq 21$	
Reflections collected:	67692	
Independent reflections:	7652 [R_{int} = 0.0271]	
Completeness to θ = 25.00°:	100.0 %	
Absorption correction:	Semi-empirical from equivalents	
Max. and min. transmission:	0.8863 and 0.8081	
Refinement method:	Full-matrix least-squares on F^2	
Data / restraints / parameters:	7652 / 0 / 408	
Goodness-of-fit on F^2:	1.035	
Final R indices [I>2σ(I)]:	R_1 = 0.0300, wR_2 = 0.0772	
R indices (all data):	R_1 = 0.0334, wR_2 = 0.0792	
Largest diff. peak and hole:	1.637 and −1.126 $e^{-}{\cdot}Å^{-3}$	

Table S32. Atomic coordinates and equivalent isotropic displacement parameters (Å^2) for [{PC(sp^3)(NH^{p}Tol)P}^{tBu}PdI] (**10**). U(eq) is defined as one third of the trace of the orthogonalized U_{ij} tensor

atom	x	y	z	U(eq)
C(24)	0.35252(19)	0.1762(3)	−0.17618(17)	0.037(1)
I	0.13372(1)	0.15340(2)	0.15375(1)	0.041(1)
Pd	0.25344(1)	0.09070(1)	0.08894(1)	0.021(1)
C(17)	0.3410(3)	−0.4063(2)	−0.0267(2)	0.058(1)
C(15)	0.37431(16)	−0.2011(2)	−0.02075(15)	0.026(1)
C(11)	0.35321(15)	−0.05118(19)	0.03694(14)	0.021(1)
C(10)	0.38007(19)	−0.3605(2)	0.04527(17)	0.031(1)
P(2)	0.20394(4)	0.15634(5)	−0.02170(4)	0.022(1)
P(1)	0.30442(4)	−0.03056(5)	0.16928(4)	0.024(1)
C(12)	0.34461(15)	−0.10199(19)	0.10101(15)	0.022(1)
C(13)	0.35399(16)	−0.2000(2)	0.10369(15)	0.025(1)
C(16)	0.36714(16)	−0.10298(19)	−0.02377(15)	0.024(1)
C(14)	0.36884(16)	−0.2522(2)	0.04297(15)	0.025(1)
C	0.35438(15)	0.0568(2)	0.04182(14)	0.022(1)
C(18)	0.4690(2)	−0.3804(3)	0.0558(2)	0.048(1)
C(19)	0.3446(3)	−0.4048(2)	0.1073(2)	0.059(1)
C(20)	0.3518(3)	0.2073(4)	−0.2558(2)	0.073(1)
C(21)	0.35434(16)	0.10589(19)	−0.03202(14)	0.021(1)
C(22)	0.28615(16)	0.14670(19)	−0.07167(15)	0.023(1)
C(23)	0.28508(18)	0.1786(2)	−0.14391(16)	0.030(1)
N	0.42855(14)	0.07706(18)	0.09214(13)	0.025(1)
C(25)	0.42185(18)	0.1417(2)	−0.13336(17)	0.033(1)
C(26)	0.42281(17)	0.1067(2)	−0.06350(16)	0.026(1)
C(29)	0.3661(3)	0.1082(4)	−0.3006(2)	0.073(1)
C(28)	0.4211(3)	0.2674(4)	−0.2637(2)	0.073(1)
C(27)	0.2772(3)	0.2381(4)	−0.2952(2)	0.073(1)
C(31)	0.38589(19)	−0.0049(2)	0.24748(16)	0.034(1)
C(33)	0.3677(2)	0.0857(2)	0.28738(18)	0.044(1)
C(32)	0.23624(19)	−0.1154(2)	0.20272(17)	0.033(1)
C(34)	0.4095(2)	−0.0878(3)	0.29944(19)	0.043(1)
C(36)	0.1711(2)	−0.1443(2)	0.1392(2)	0.043(1)
C(35)	0.2002(2)	−0.0757(3)	0.2667(2)	0.045(1)
C(41)	0.12300(17)	0.0888(2)	−0.07864(17)	0.032(1)
C(43)	0.1454(2)	−0.0166(2)	−0.0782(2)	0.049(1)
C(42)	0.16946(18)	0.2813(2)	−0.02540(17)	0.031(1)
C(45)	0.2318(2)	0.3460(2)	0.0185(2)	0.042(1)
C(46)	0.1402(2)	0.3213(3)	−0.1024(2)	0.049(1)
C(51)	0.45669(16)	0.16396(19)	0.12057(14)	0.023(1)
C(52)	0.52569(17)	0.1667(2)	0.17412(15)	0.028(1)
C(53)	0.55516(18)	0.2527(2)	0.20313(16)	0.033(1)
C(54)	0.51887(19)	0.3388(2)	0.18092(16)	0.033(1)
C(55)	0.45032(18)	0.3343(2)	0.12770(16)	0.030(1)
C(56)	0.41976(17)	0.2497(2)	0.09825(16)	0.027(1)
C(57)	0.5512(2)	0.4325(3)	0.2128(2)	0.051(1)
C(44)	0.0432(2)	0.1027(3)	−0.0549(2)	0.051(1)
H(17A)	0.3656	−0.3817	−0.0672	0.088
H(17B)	0.2841	−0.3911	−0.0361	0.088
H(17C)	0.3480	−0.4756	−0.0234	0.088
H	0.4557(19)	0.030(2)	0.1089(17)	0.029(9)

Continued on next page

Table S32. – continued from previous page

atom	x	y	x	U(eq)
H(15)	0.3832	−0.2347	−0.0634	0.031
H(13)	0.3502	−0.2327	0.1481	0.030
H(16)	0.3718	−0.0704	−0.0681	0.028
H(18A)	0.4779	−0.4494	0.0552	0.072
H(18B)	0.4951	−0.3542	0.1033	0.072
H(18C)	0.4912	−0.3504	0.0159	0.072
H(19A)	0.2892	−0.3846	0.1034	0.089
H(19B)	0.3749	−0.3841	0.1549	0.089
H(19C)	0.3469	−0.4745	0.1039	0.089
H(23)	0.2369	0.2024	−0.1715	0.035
H(25)	0.4698	0.1423	−0.1529	0.040
H(26)	0.4711	0.0825	−0.0361	0.031
H(29A)	0.3684	0.1235	−0.3521	0.109
H(29B)	0.3221	0.0639	−0.2987	0.109
H(29C)	0.4162	0.0786	−0.2774	0.109
H(28A)	0.4208	0.2802	−0.3161	0.109
H(28B)	0.4702	0.2339	−0.2427	0.109
H(28C)	0.4182	0.3279	−0.2376	0.109
H(27A)	0.2813	0.2491	−0.3469	0.087
H(27B)	0.2617	0.2975	−0.2735	0.087
H(27C)	0.2371	0.1889	−0.2923	0.087
H(31)	0.4337	0.0100	0.2250	0.040
H(33A)	0.3578	0.1385	0.2520	0.066
H(33B)	0.4131	0.1014	0.3259	0.066
H(33C)	0.3205	0.0754	0.3098	0.066
H(32)	0.2673	−0.1739	0.2205	0.039
H(34A)	0.4206	−0.1438	0.2711	0.064
H(34B)	0.3659	−0.1023	0.3259	0.064
H(34C)	0.4571	−0.0708	0.3349	0.064
H(36A)	0.1398	−0.0880	0.1208	0.064
H(36B)	0.1365	−0.1918	0.1564	0.064
H(36C)	0.1952	−0.1717	0.0994	0.064
H(35A)	0.1687	−0.0188	0.2504	0.068
H(35B)	0.2429	−0.0588	0.3075	0.068
H(35C)	0.1659	−0.1241	0.2833	0.068
H(41)	0.1181	0.1123	−0.1305	0.038
H(43A)	0.1037	−0.0522	−0.1104	0.074
H(43B)	0.1511	−0.0415	−0.0278	0.074
H(43C)	0.1959	−0.0239	−0.0960	0.074
H(42)	0.1228	0.2828	0.0006	0.037
H(45A)	0.2783	0.3493	−0.0060	0.063
H(45B)	0.2478	0.3202	0.0683	0.063
H(45C)	0.2097	0.4101	0.0213	0.063
H(46A)	0.1847	0.3252	−0.1294	0.073
H(46B)	0.1179	0.3852	−0.0984	0.073
H(46C)	0.0990	0.2793	−0.1290	0.073
H(52)	0.5523	0.1091	0.1905	0.034
H(53)	0.6019	0.2530	0.2396	0.040
H(55)	0.4238	0.3919	0.1112	0.036
H(56)	0.3727	0.2497	0.0621	0.032
H(57A)	0.5453	0.4808	0.1738	0.076

Continued on next page

Table S32. – continued from previous page

atom	x	y	x	U(eq)
H(57B)	0.6076	0.4252	0.2341	0.076
H(57C)	0.5219	0.4525	0.2514	0.076
H(44A)	0.0267	0.1695	−0.0625	0.077
H(44B)	0.0477	0.0865	−0.0026	0.077
H(44C)	0.0036	0.0612	−0.0843	0.077

Table S33. Anisotropic displacement parameters (Å^2) for [{PC(sp^3)(NH^{p}Tol)P}^{tBu}PdI] (**10**). The anisotropic displacement factor exponent takes the form: $-2\pi^2[h^2a^{*2}U_{11} + ... + 2hka^*b^*U_{12}]$.

**atom**	**U$_{11}$**	**U$_{22}$**	**U$_{33}$**	**U$_{23}$**	**U$_{13}$**	**U$_{12}$**
C(24)	0.0310(17)	0.052(2)	0.0281(15)	0.0065(14)	0.0065(13)	−0.0040(15)
I	0.0410(1)	0.0470(1)	0.0415(1)	0.0016(1)	0.0231(1)	0.0196(1)
Pd	0.0210(1)	0.0230(1)	0.0218(1)	−0.0009(1)	0.0080(1)	0.0049(1)
C(17)	0.090(3)	0.0246(17)	0.059(2)	−0.0061(16)	0.007(2)	−0.0089(18)
C(15)	0.0266(15)	0.0278(15)	0.0261(14)	−0.0032(11)	0.0124(12)	0.0013(12)
C(11)	0.0166(13)	0.0235(13)	0.0240(13)	0.0012(11)	0.0060(10)	0.0024(10)
C(10)	0.0423(18)	0.0227(14)	0.0330(16)	0.0036(12)	0.0210(14)	0.0051(13)
P(2)	0.0179(3)	0.0237(4)	0.0246(3)	−0.0021(3)	0.0036(3)	0.0028(3)
P(1)	0.0282(4)	0.0258(4)	0.0218(3)	0.0008(3)	0.0119(3)	0.0057(3)
C(12)	0.0188(13)	0.0267(14)	0.0235(13)	−0.0013(11)	0.0090(11)	0.0028(11)
C(13)	0.0246(14)	0.0274(14)	0.0243(14)	0.0041(11)	0.0107(11)	0.0032(11)
C(16)	0.0240(14)	0.0256(14)	0.0236(13)	0.0022(11)	0.0094(11)	0.0005(11)
C(14)	0.0236(14)	0.0261(14)	0.0294(14)	0.0007(11)	0.0120(12)	0.0018(11)
C	0.0171(13)	0.0261(14)	0.0226(13)	0.0010(11)	0.0062(10)	0.0029(11)
C(18)	0.055(2)	0.0312(17)	0.062(2)	0.0074(16)	0.0223(19)	0.0147(16)
C(19)	0.099(3)	0.0227(17)	0.072(3)	0.0079(16)	0.063(3)	0.0081(18)
C(20)	0.0612(13)	0.116(2)	0.0432(11)	0.0227(12)	0.0151(10)	0.0021(14)
C(21)	0.0215(14)	0.0206(13)	0.0224(13)	−0.0019(10)	0.0045(11)	−0.0008(11)
C(22)	0.0217(14)	0.0230(14)	0.0255(14)	−0.0015(11)	0.0046(11)	−0.0007(11)
C(23)	0.0252(15)	0.0376(16)	0.0247(14)	0.0043(12)	0.0013(12)	0.0003(13)
N	0.0216(12)	0.0243(12)	0.0281(12)	0.0011(10)	0.0027(10)	0.0051(10)
C(25)	0.0251(16)	0.0446(18)	0.0327(16)	0.0041(14)	0.0125(13)	−0.0043(13)
C(26)	0.0213(14)	0.0278(15)	0.0300(15)	0.0033(12)	0.0058(11)	0.0018(11)
C(29)	0.0612(13)	0.116(2)	0.0432(11)	0.0227(12)	0.0151(10)	0.0021(14)
C(28)	0.0612(13)	0.116(2)	0.0432(11)	0.0227(12)	0.0151(10)	0.0021(14)
C(27)	0.0612(13)	0.116(2)	0.0432(11)	0.0227(12)	0.0151(10)	0.0021(14)
C(31)	0.0412(18)	0.0354(17)	0.0257(15)	−0.0006(12)	0.0103(13)	0.0039(14)
C(33)	0.062(2)	0.0393(19)	0.0313(17)	−0.0084(14)	0.0071(16)	0.0048(16)
C(32)	0.0379(17)	0.0310(15)	0.0347(16)	0.0066(13)	0.0220(14)	0.0047(13)
C(34)	0.046(2)	0.046(2)	0.0353(17)	0.0051(15)	0.0059(15)	0.0082(16)
C(36)	0.0311(18)	0.0423(19)	0.060(2)	0.0041(16)	0.0221(16)	−0.0039(14)
C(35)	0.052(2)	0.046(2)	0.047(2)	0.0086(16)	0.0365(17)	0.0115(17)
C(41)	0.0198(14)	0.0390(17)	0.0347(16)	−0.0068(13)	0.0005(12)	−0.0029(12)
C(43)	0.044(2)	0.0361(19)	0.062(2)	−0.0191(17)	−0.0047(17)	−0.0057(16)
C(42)	0.0287(15)	0.0271(15)	0.0360(16)	−0.0008(12)	0.0035(13)	0.0083(12)
C(45)	0.0399(19)	0.0267(16)	0.057(2)	−0.0049(15)	0.0020(16)	0.0054(14)
C(46)	0.061(2)	0.0401(19)	0.044(2)	0.0059(16)	0.0058(17)	0.0219(18)
C(51)	0.0215(14)	0.0273(14)	0.0208(13)	0.0010(11)	0.0094(11)	0.0006(11)
C(52)	0.0266(15)	0.0352(16)	0.0226(14)	0.0031(12)	0.0056(12)	0.0014(12)
C(53)	0.0299(16)	0.0463(18)	0.0215(14)	0.0023(13)	0.0008(12)	−0.0033(14)
C(54)	0.0382(17)	0.0370(17)	0.0238(14)	−0.0036(12)	0.0085(13)	−0.0064(14)
C(55)	0.0343(17)	0.0276(15)	0.0298(15)	0.0016(12)	0.0079(13)	0.0008(13)
C(56)	0.0231(14)	0.0302(15)	0.0277(14)	0.0005(12)	0.0042(11)	0.0015(12)
C(57)	0.063(2)	0.044(2)	0.0395(19)	−0.0074(16)	−0.0036(17)	−0.0105(18)
C(44)	0.0247(17)	0.063(2)	0.065(2)	−0.020(2)	0.0092(16)	−0.0107(16)

Table S34. Distances [Å] for [{PC(sp^3)(NH^{p}Tol)P}^{tBu}PdI] (**10**).

atom – atom	distance	atom – atom	distance
C(24) – C(23)	1.386(4)	C(24) – C(25)	1.390(5)
C(24) – C(20)	1.528(5)	I – Pd	2.6903(3)
Pd – C	2.116(3)	Pd – P(2)	2.2609(8)
Pd – P(1)	2.3206(7)	C(17) – C(10)	1.519(5)
C(17) – H(17A)	0.9800	C(17) – H(17B)	0.9800
C(17) – H(17C)	0.9800	C(15) – C(16)	1.381(4)
C(15) – C(14)	1.392(4)	C(15) – H(15)	0.9500
C(11) – C(16)	1.388(4)	C(11) – C(12)	1.408(4)
C(11) – C	1.515(4)	C(10) – C(19)	1.518(4)
C(10) – C(18)	1.524(5)	C(10) – C(14)	1.529(4)
P(2) – C(22)	1.815(3)	P(2) – C(41)	1.845(3)
P(2) – C(42)	1.845(3)	P(1) – C(12)	1.833(3)
P(1) – C(32)	1.844(3)	P(1) – C(31)	1.855(3)
C(12) – C(13)	1.383(4)	C(13) – C(14)	1.397(4)
C(13) – H(13)	0.9500	C(16) – H(16)	0.9500
C – N	1.461(3)	C – C(21)	1.524(4)
C(18) – H(18A)	0.9800	C(18) – H(18B)	0.9800
C(18) – H(18C)	0.9800	C(19) – H(19A)	0.9800
C(19) – H(19B)	0.9800	C(19) – H(19C)	0.9800
C(20) – C(27)	1.419(6)	C(20) – C(28)	1.482(7)
C(20) – C(29)	1.654(8)	C(21) – C(22)	1.386(4)
C(21) – C(26)	1.394(4)	C(22) – C(23)	1.401(4)
C(23) – H(23)	0.9500	N – C(51)	1.378(4)
N – H	0.84(3)	C(25) – C(26)	1.375(4)
C(25) – H(25)	0.9500	C(26) – H(26)	0.9500
C(29) – H(29A)	0.9800	C(29) – H(29B)	0.9800
C(29) – H(29C)	0.9800	C(28) – H(28A)	0.9800
C(28) – H(28B)	0.9800	C(28) – H(28C)	0.9800
C(27) – H(27A)	0.9800	C(27) – H(27B)	0.9800
C(27) – H(27C)	0.9800	C(31) – C(34)	1.515(4)
C(31) – C(33)	1.526(4)	C(31) – H(31)	1.0000
C(33) – H(33A)	0.9800	C(33) – H(33B)	0.9800
C(33) – H(33C)	0.9800	C(32) – C(36)	1.522(5)
C(32) – C(35)	1.527(4)	C(32) – H(32)	1.0000
C(34) – H(34A)	0.9800	C(34) – H(34B)	0.9800
C(34) – H(34C)	0.9800	C(36) – H(36A)	0.9800
C(36) – H(36B)	0.9800	C(36) – H(36C)	0.9800
C(35) – H(35A)	0.9800	C(35) – H(35B)	0.9800
C(35) – H(35C)	0.9800	C(41) – C(44)	1.516(4)
C(41) – C(43)	1.526(5)	C(41) – H(41)	1.0000
C(43) – H(43A)	0.9800	C(43) – H(43B)	0.9800
C(43) – H(43C)	0.9800	C(42) – C(45)	1.519(4)
C(42) – C(46)	1.527(4)	C(42) – H(42)	1.0000
C(45) – H(45A)	0.9800	C(45) – H(45B)	0.9800
C(45) – H(45C)	0.9800	C(46) – H(46A)	0.9800
C(46) – H(46B)	0.9800	C(46) – H(46C)	0.9800
C(51) – C(56)	1.385(4)	C(51) – C(52)	1.399(4)
C(52) – C(53)	1.377(4)	C(52) – H(52)	0.9500
C(53) – C(54)	1.385(5)	C(53) – H(53)	0.9500
C(54) – C(55)	1.391(4)	C(54) – C(57)	1.504(4)

Continued on next page

Table S34. – continued from previous page

**atom – atom**	**distance**	**atom – atom**	**distance**
C(55) – C(56)	1.369(4)	C(55) – H(55)	0.9500
C(56) – H(56)	0.9500	C(57) – H(57A)	0.9800
C(57) – H(57B)	0.9800	C(57) – H(57C)	0.9800
C(44) – H(44A)	0.9800	C(44) – H(44B)	0.9800
C(44) – H(44C)	0.9800		

Table S35. Angles [°] for [{PC(sp^3)(NH^{p}Tol)P}^{tBu}PdI] (**10**).

atom – atom – atom	angle	atom – atom – atom	angle
C(23) – C(24) – C(25)	117.0(3)	C(23) – C(24) – C(20)	122.5(3)
C(25) – C(24) – C(20)	120.4(3)	C – Pd – P(2)	85.69(7)
C – Pd – P(1)	81.67(7)	P(2) – Pd – P(1)	155.81(3)
C – Pd – I	173.25(8)	P(2) – Pd – I	94.22(2)
P(1) – Pd – I	100.715(19)	C(10) – C(17) – H(17A)	109.5
C(10) – C(17) – H(17B)	109.5	H(17A) – C(17) – H(17B)	109.5
C(10) – C(17) – H(17C)	109.5	H(17A) – C(17) – H(17C)	109.5
H(17B) – C(17) – H(17C)	109.5	C(16) – C(15) – C(14)	121.9(3)
C(16) – C(15) – H(15)	119.1	C(14) – C(15) – H(15)	119.1
C(16) – C(11) – C(12)	117.9(2)	C(16) – C(11) – C	124.6(2)
C(12) – C(11) – C	117.2(2)	C(19) – C(10) – C(17)	108.2(3)
C(19) – C(10) – C(18)	110.4(3)	C(17) – C(10) – C(18)	108.1(3)
C(19) – C(10) – C(14)	111.3(2)	C(17) – C(10) – C(14)	111.2(3)
C(18) – C(10) – C(14)	107.6(3)	C(22) – P(2) – C(41)	104.09(13)
C(22) – P(2) – C(42)	108.81(13)	C(41) – P(2) – C(42)	105.27(14)
C(22) – P(2) – Pd	103.08(9)	C(41) – P(2) – Pd	115.39(11)
C(42) – P(2) – Pd	119.08(10)	C(12) – P(1) – C(32)	102.23(13)
C(12) – P(1) – C(31)	108.57(13)	C(32) – P(1) – C(31)	107.34(15)
C(12) – P(1) – Pd	96.10(9)	C(32) – P(1) – Pd	119.76(11)
C(31) – P(1) – Pd	120.01(10)	C(13) – C(12) – C(11)	120.3(2)
C(13) – C(12) – P(1)	125.0(2)	C(11) – C(12) – P(1)	113.9(2)
C(12) – C(13) – C(14)	121.9(3)	C(12) – C(13) – H(13)	119.1
C(14) – C(13) – H(13)	119.1	C(15) – C(16) – C(11)	121.0(3)
C(15) – C(16) – H(16)	119.5	C(11) – C(16) – H(16)	119.5
C(15) – C(14) – C(13)	117.0(3)	C(15) – C(14) – C(10)	120.5(2)
C(13) – C(14) – C(10)	122.5(2)	N – C – C(11)	103.5(2)
N – C – C(21)	110.1(2)	C(11) – C – C(21)	113.6(2)
N – C – Pd	112.02(17)	C(11) – C – Pd	104.20(17)
C(21) – C – Pd	113.05(17)	C(10) – C(18) – H(18A)	109.5
C(10) – C(18) – H(18B)	109.5	H(18A) – C(18) – H(18B)	109.5
C(10) – C(18) – H(18C)	109.5	H(18A) – C(18) – H(18C)	109.5
H(18B) – C(18) – H(18C)	109.5	C(10) – C(19) – H(19A)	109.5
C(10) – C(19) – H(19B)	109.5	H(19A) – C(19) – H(19B)	109.5
C(10) – C(19) – H(19C)	109.5	H(19A) – C(19) – H(19C)	109.5
H(19B) – C(19) – H(19C)	109.5	C(27) – C(20) – C(28)	116.0(4)
C(27) – C(20) – C(24)	115.6(4)	C(28) – C(20) – C(24)	112.6(4)
C(27) – C(20) – C(29)	101.4(4)	C(28) – C(20) – C(29)	103.8(4)
C(24) – C(20) – C(29)	105.2(4)	C(22) – C(21) – C(26)	117.8(2)
C(22) – C(21) – C	121.7(2)	C(26) – C(21) – C	120.4(2)
C(21) – C(22) – C(23)	120.2(3)	C(21) – C(22) – P(2)	114.7(2)
C(23) – C(22) – P(2)	125.2(2)	C(24) – C(23) – C(22)	121.7(3)
C(24) – C(23) – H(23)	119.1	C(22) – C(23) – H(23)	119.1
C(51) – N – C	128.2(2)	C(51) – N – H	115(2)
C – N – H	116(2)	C(26) – C(25) – C(24)	121.7(3)
C(26) – C(25) – H(25)	119.2	C(24) – C(25) – H(25)	119.2
C(25) – C(26) – C(21)	121.3(3)	C(25) – C(26) – H(26)	119.4
C(21) – C(26) – H(26)	119.4	C(20) – C(29) – H(29A)	109.5
C(20) – C(29) – H(29B)	109.5	H(29A) – C(29) – H(29B)	109.5
C(20) – C(29) – H(29C)	109.5	H(29A) – C(29) – H(29C)	109.5
H(29B) – C(29) – H(29C)	109.5	C(20) – C(28) – H(28A)	109.5

Continued on next page

Table S35. – continued from previous page

atom – atom – atom	angle	atom – atom – atom	angle
C(20) – C(28) – H(28B)	109.5	H(28A) – C(28) – H(28B)	109.5
C(20) – C(28) – H(28C)	109.5	H(28A) – C(28) – H(28C)	109.5
H(28B) – C(28) – H(28C)	109.5	C(20) – C(27) – H(27A)	109.5
C(20) – C(27) – H(27B)	109.5	H(27A) – C(27) – H(27B)	109.5
C(20) – C(27) – H(27C)	109.5	H(27A) – C(27) – H(27C)	109.5
H(27B) – C(27) – H(27C)	109.5	C(34) – C(31) – C(33)	112.8(3)
C(34) – C(31) – P(1)	115.0(2)	C(33) – C(31) – P(1)	110.3(2)
C(34) – C(31) – H(31)	106.0	C(33) – C(31) – H(31)	106.0
P(1) – C(31) – H(31)	106.0	C(31) – C(33) – H(33A)	109.5
C(31) – C(33) – H(33B)	109.5	H(33A) – C(33) – H(33B)	109.5
C(31) – C(33) – H(33C)	109.5	H(33A) – C(33) – H(33C)	109.5
H(33B) – C(33) – H(33C)	109.5	C(36) – C(32) – C(35)	110.6(3)
C(36) – C(32) – P(1)	109.5(2)	C(35) – C(32) – P(1)	112.8(2)
C(36) – C(32) – H(32)	107.9	C(35) – C(32) – H(32)	107.9
P(1) – C(32) – H(32)	107.9	C(31) – C(34) – H(34A)	109.5
C(31) – C(34) – H(34B)	109.5	H(34A) – C(34) – H(34B)	109.5
C(31) – C(34) – H(34C)	109.5	H(34A) – C(34) – H(34C)	109.5
H(34B) – C(34) – H(34C)	109.5	C(32) – C(36) – H(36A)	109.5
C(32) – C(36) – H(36B)	109.5	H(36A) – C(36) – H(36B)	109.5
C(32) – C(36) – H(36C)	109.5	H(36A) – C(36) – H(36C)	109.5
H(36B) – C(36) – H(36C)	109.5	C(32) – C(35) – H(35A)	109.5
C(32) – C(35) – H(35B)	109.5	H(35A) – C(35) – H(35B)	109.5
C(32) – C(35) – H(35C)	109.5	H(35A) – C(35) – H(35C)	109.5
H(35B) – C(35) – H(35C)	109.5	C(44) – C(41) – C(43)	111.2(3)
C(44) – C(41) – P(2)	113.1(2)	C(43) – C(41) – P(2)	109.3(2)
C(44) – C(41) – H(41)	107.7	C(43) – C(41) – H(41)	107.7
P(2) – C(41) – H(41)	107.7	C(41) – C(43) – H(43A)	109.5
C(41) – C(43) – H(43B)	109.5	H(43A) – C(43) – H(43B)	109.5
C(41) – C(43) – H(43C)	109.5	H(43A) – C(43) – H(43C)	109.5
H(43B) – C(43) – H(43C)	109.5	C(45) – C(42) – C(46)	111.1(3)
C(45) – C(42) – P(2)	110.9(2)	C(46) – C(42) – P(2)	115.7(2)
C(45) – C(42) – H(42)	106.1	C(46) – C(42) – H(42)	106.1
P(2) – C(42) – H(42)	106.1	C(42) – C(45) – H(45A)	109.5
C(42) – C(45) – H(45B)	109.5	H(45A) – C(45) – H(45B)	109.5
C(42) – C(45) – H(45C)	109.5	H(45A) – C(45) – H(45C)	109.5
H(45B) – C(45) – H(45C)	109.5	C(42) – C(46) – H(46A)	109.5
C(42) – C(46) – H(46B)	109.5	H(46A) – C(46) – H(46B)	109.5
C(42) – C(46) – H(46C)	109.5	H(46A) – C(46) – H(46C)	109.5
H(46B) – C(46) – H(46C)	109.5	N – C(51) – C(56)	122.9(2)
N – C(51) – C(52)	119.2(3)	C(56) – C(51) – C(52)	118.0(3)
C(53) – C(52) – C(51)	120.2(3)	C(53) – C(52) – H(52)	119.9
C(51) – C(52) – H(52)	119.9	C(52) – C(53) – C(54)	122.2(3)
C(52) – C(53) – H(53)	118.9	C(54) – C(53) – H(53)	118.9
C(53) – C(54) – C(55)	116.5(3)	C(53) – C(54) – C(57)	122.0(3)
C(55) – C(54) – C(57)	121.5(3)	C(56) – C(55) – C(54)	122.3(3)
C(56) – C(55) – H(55)	118.9	C(54) – C(55) – H(55)	118.9
C(55) – C(56) – C(51)	120.7(3)	C(55) – C(56) – H(56)	119.6
C(51) – C(56) – H(56)	119.6	C(54) – C(57) – H(57A)	109.5
C(54) – C(57) – H(57B)	109.5	H(57A) – C(57) – H(57B)	109.5
C(54) – C(57) – H(57C)	109.5	H(57A) – C(57) – H(57C)	109.5
H(57B) – C(57) – H(57C)	109.5	C(41) – C(44) – H(44A)	109.5

Continued on next page

Table S35. – continued from previous page

atom – atom – atom	angle	atom – atom – atom	angle
C(41) – C(44) – H(44B)	109.5	H(44A) – C(44) – H(44B)	109.5
C(41) – C(44) – H(44C)	109.5	H(44A) – C(44) – H(44C)	109.5
H(44B) – C(44) – H(44C)	109.5		

5.7 Crystal data for [{PC(sp^3)(OPhP)}^{tBu}PdI] (11)

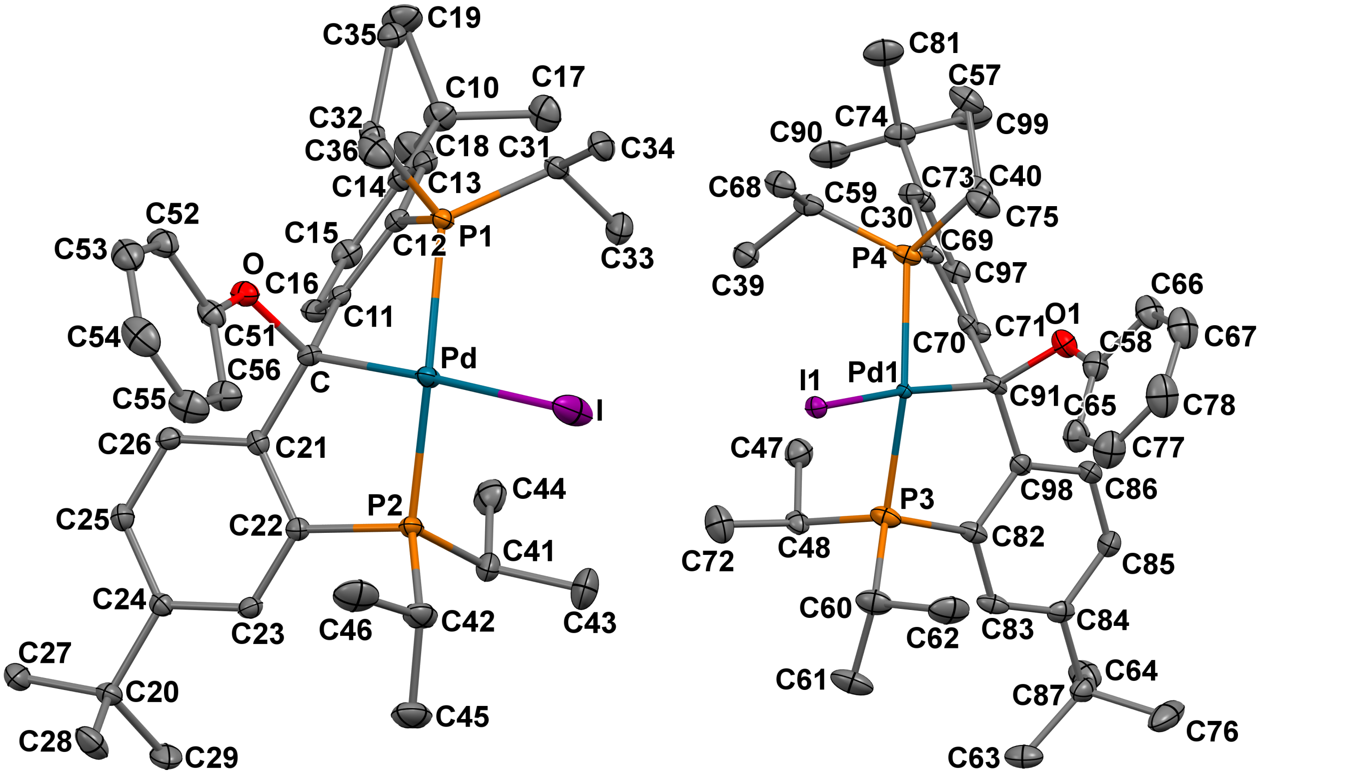

Figure S50. Thermal-ellipsoid representation of the two crystallographically independent molecules of [{PC(sp^3)(OPhP)}^{tBu}PdI] (**11**) at 50% probability. Hydrogen atoms and the solvent were omitted for clarity.

Table S36. Crystal data and structure refinement for [{PC(sp^3)(OPhP)}^{tBu}PdI] (**11**).

Identification code:	pc35b	
Empirical formula:	$C_{39}H_{57}IOP_2Pd$	
Formula weight:	837.09	
Temperature:	120(2) K	
Wavelength:	0.71073 Å	
Crystal system:	Triclinic	
Space group:	$P\bar{1}$	
Unit cell dimensions:	a = 11.8764(6) Å	α = 101.2966(16)°
	b = 14.4330(8) Å	β = 93.5784(16)°
	c = 25.1741(14) Å	γ = 111.2000(15)°
Volume:	3903.0(4) $Å^3$	
Z:	4	
Density (calculated):	1.425 $g{\cdot}cm^{-3}$	
Absorption coefficient (μ):	1.376 mm^{-1}	
F(000):	1712	
Crystal size:	0.09 × 0.08 × 0.05 mm^3	
θ range for data collection:	1.67 to 25.00°	
Index ranges:	−14 ≤ h ≤ 14, −17 ≤ k ≤ 17, −29 ≤ l ≤ 29	
Reflections collected:	95643	
Independent reflections:	13721 [R_{int} = 0.0511]	
Completeness to θ = 25.00°:	100.0 %	
Absorption correction:	Semi-empirical from equivalents	
Max. and min. transmission:	0.9344 and 0.8862	
Refinement method:	Full-matrix least-squares on F^2	
Data / restraints / parameters:	13721 / 0 / 841	
Goodness-of-fit on F^2:	1.031	
Final R indices [I>2σ(I)]:	R_1 = 0.0389, wR_2 = 0.0886	
R indices (all data):	R_1 = 0.0556, wR_2 = 0.0934	
Largest diff. peak and hole:	3.446 and −2.064 $e^{-}{\cdot}Å^{-3}$	

Table S37. Atomic coordinates and equivalent isotropic displacement parameters ($Å^2$) for [{PC(sp^3)(OPhP)}^{tBu}PdI] (**11**). U(eq) is defined as one third of the trace of the orthogonalized U_{ij} tensor

atom	x	y	z	U(eq)
Pd(1)	0.8684(3)	0.1959(2)	0.14206(12)	0.010(1)
I(1)	1.02028(17)	0.32115(14)	0.23212(8)	0.019(1)
P(1)	0.42571(9)	0.59684(8)	0.33646(4)	0.018(1)
O(1)	0.6304(3)	0.1504(2)	0.07849(13)	0.030(1)
C(62)	1.0721(5)	0.4355(4)	0.0911(2)	0.047(1)
C(65)	0.7484(5)	0.3354(4)	0.1010(2)	0.036(1)
C(60)	1.1278(5)	0.3545(4)	0.0912(2)	0.038(1)
C(59)	0.7727(4)	0.0268(3)	0.22811(17)	0.027(1)
C(58)	0.6428(4)	0.2503(4)	0.09767(19)	0.031(1)
C(57)	0.5002(4)	0.0237(4)	0.2253(2)	0.037(1)
C(56)	0.6881(4)	0.8358(3)	0.4752(2)	0.031(1)
C(55)	0.7103(5)	0.9380(4)	0.4983(2)	0.039(1)
C(54)	0.6248(5)	0.9649(4)	0.5248(2)	0.043(1)
C(53)	0.5161(5)	0.8892(4)	0.5286(2)	0.035(1)
C(61)	1.2237(5)	0.3638(4)	0.0523(2)	0.049(2)
I	0.67166(3)	0.77566(3)	0.26906(2)	0.051(1)
O	0.5443(2)	0.6575(2)	0.45674(12)	0.023(1)
Pd	0.63714(3)	0.68113(2)	0.35155(1)	0.019(1)
P(2)	0.83558(9)	0.69929(8)	0.36686(4)	0.020(1)
P(4)	0.71833(10)	0.08513(8)	0.17798(4)	0.021(1)
C(10)	0.2721(4)	0.1929(3)	0.32749(19)	0.028(1)
C(11)	0.5314(3)	0.5068(3)	0.39824(16)	0.017(1)
C	0.6169(3)	0.6177(3)	0.41989(16)	0.018(1)
C(12)	0.4264(4)	0.4864(3)	0.36140(16)	0.017(1)
C(13)	0.3428(4)	0.3865(3)	0.34114(17)	0.021(1)
C(16)	0.5489(4)	0.4241(3)	0.41210(17)	0.023(1)
C(15)	0.4670(4)	0.3244(3)	0.38942(18)	0.024(1)
C(14)	0.3624(4)	0.3027(3)	0.35429(17)	0.022(1)
C(20)	1.0815(4)	0.6877(3)	0.55111(17)	0.024(1)
C(19)	0.1482(4)	0.1778(4)	0.3464(2)	0.040(1)
C(18)	0.3151(5)	0.1130(3)	0.3428(2)	0.040(1)
C(17)	0.2578(5)	0.1779(4)	0.26515(19)	0.036(1)
C(21)	0.7366(4)	0.6336(3)	0.45367(16)	0.018(1)
C(22)	0.8474(4)	0.6695(3)	0.43364(16)	0.018(1)
C(23)	0.9552(4)	0.6823(3)	0.46460(16)	0.019(1)
C(24)	0.9586(4)	0.6641(3)	0.51679(16)	0.019(1)
C(25)	0.8467(4)	0.6293(3)	0.53626(17)	0.022(1)
C(26)	0.7384(4)	0.6138(3)	0.50555(17)	0.022(1)
C(27)	1.0660(4)	0.6515(4)	0.60411(18)	0.031(1)
C(28)	1.1524(4)	0.8036(4)	0.5650(2)	0.038(1)
C(29)	1.1551(4)	0.6348(4)	0.5188(2)	0.036(1)
C(30)	0.6010(4)	−0.1223(3)	0.11641(17)	0.022(1)
C(31)	0.3448(4)	0.5423(3)	0.26611(17)	0.022(1)
C(32)	0.3241(4)	0.6457(3)	0.37583(17)	0.022(1)
C(33)	0.4162(4)	0.4923(4)	0.23023(18)	0.033(1)
C(81)	0.4538(17)	−0.3291(12)	0.1233(7)	0.043(1)
C(90)	0.5903(15)	−0.3677(11)	0.0610(7)	0.043(1)
C(99)	0.3953(15)	−0.3649(10)	0.0229(6)	0.043(1)
C(35)	0.1891(4)	0.5767(4)	0.35983(18)	0.028(1)

Continued on next page

Table S37. – continued from previous page

atom	x	y	x	U(eq)
C(34)	0.3163(4)	0.6202(4)	0.24028(19)	0.032(1)
C(39)	0.8789(4)	−0.0008(4)	0.20992(19)	0.032(1)
C(36)	0.3518(5)	0.7580(4)	0.3764(2)	0.039(1)
C(40)	0.5847(4)	0.1090(3)	0.20154(18)	0.025(1)
C(41)	0.8760(4)	0.6027(4)	0.32121(18)	0.032(1)
C(42)	0.9564(4)	0.8240(4)	0.3667(2)	0.033(1)
C(43)	0.8964(5)	0.6242(5)	0.2651(2)	0.051(2)
C(44)	0.7794(5)	0.4961(4)	0.3159(2)	0.038(1)
C(45)	1.0876(4)	0.8317(4)	0.3753(2)	0.044(1)
C(46)	0.9405(5)	0.9114(4)	0.4055(2)	0.046(1)
C(47)	0.9942(5)	0.0295(4)	0.0676(2)	0.036(1)
C(48)	1.0860(4)	0.1381(3)	0.07468(18)	0.025(1)
C(51)	0.5785(4)	0.7606(3)	0.47889(18)	0.025(1)
C(52)	0.4922(4)	0.7873(4)	0.50570(18)	0.030(1)
C(63)	1.0546(5)	0.2494(4)	−0.1315(2)	0.042(1)
C(64)	0.8720(5)	0.0997(4)	−0.1777(2)	0.042(1)
C(66)	0.5384(5)	0.2619(4)	0.1146(2)	0.040(1)
C(67)	0.5418(5)	0.3593(4)	0.1363(2)	0.049(1)
C(72)	1.1857(5)	0.1661(4)	0.1227(2)	0.047(1)
C(71)	0.6569(4)	−0.0662(3)	0.01992(16)	0.020(1)
C(70)	0.6847(3)	0.0103(3)	0.06733(16)	0.018(1)
C(69)	0.6585(4)	−0.0197(3)	0.11627(16)	0.019(1)
C(68)	0.8066(4)	0.0920(4)	0.28660(18)	0.035(1)
C(75)	0.6250(5)	0.2148(4)	0.2408(2)	0.040(1)
C(74)	0.5087(4)	−0.3125(3)	0.06905(19)	0.028(1)
C(73)	0.5704(4)	−0.1991(3)	0.06871(17)	0.022(1)
C(76)	0.8628(6)	0.2719(5)	−0.1586(3)	0.063(2)
C(77)	0.7503(5)	0.4319(4)	0.1236(2)	0.046(1)
C(78)	0.6475(6)	0.4445(5)	0.1416(2)	0.052(2)
C(97)	0.6018(4)	−0.1684(3)	0.02116(17)	0.023(1)
C(91)	0.7375(3)	0.1245(3)	0.06987(17)	0.019(1)
C(98)	0.7832(4)	0.1508(3)	0.01749(18)	0.022(1)
C(82)	0.9084(4)	0.1959(3)	0.01577(18)	0.024(1)
C(83)	0.9504(4)	0.2108(3)	−0.03393(18)	0.026(1)
C(84)	0.8715(4)	0.1862(3)	−0.08212(19)	0.025(1)
C(85)	0.7467(4)	0.1464(4)	−0.0781(2)	0.032(1)
C(86)	0.7029(4)	0.1281(4)	−0.03007(19)	0.031(1)
C(87)	0.9163(4)	0.2028(4)	−0.13642(19)	0.031(1)
C(92)	0.4174(10)	−0.3263(7)	0.1068(4)	0.043(1)
C(79)	0.4549(10)	−0.3787(6)	0.0112(4)	0.043(1)
C(80)	0.6097(9)	−0.3458(7)	0.0906(5)	0.043(1)
I(3)	1.0096(6)	0.3292(4)	0.2262(3)	0.066(1)
Pd(91)	0.8595(6)	0.2025(5)	0.1361(3)	0.037(1)
P(3)	1.00566(10)	0.22812(9)	0.08173(5)	0.027(1)
H(62A)	1.1377	0.5034	0.0981	0.071
H(62B)	1.0198	0.4338	0.1198	0.071
H(62C)	1.0234	0.4210	0.0554	0.071
H(65)	0.8187	0.3278	0.0880	0.043
H(60)	1.1728	0.3707	0.1289	0.046
H(59)	0.7041	−0.0385	0.2283	0.032
H(57A)	0.4263	0.0364	0.2323	0.056

Continued on next page

Table S37. – continued from previous page

atom	x	y	x	U(eq)
H(57B)	0.5425	0.0223	0.2596	0.056
H(57C)	0.4779	−0.0422	0.1990	0.056
H(56)	0.7478	0.8180	0.4571	0.037
H(55)	0.7854	0.9897	0.4958	0.046
H(54)	0.6404	1.0346	0.5404	0.051
H(53)	0.4569	0.9071	0.5470	0.042
H(61A)	1.2396	0.3007	0.0447	0.073
H(61B)	1.2994	0.4217	0.0694	0.073
H(61C)	1.1933	0.3748	0.0180	0.073
H(13)	0.2705	0.3746	0.3178	0.026
H(16)	0.6179	0.4357	0.4375	0.027
H(15)	0.4840	0.2694	0.3985	0.029
H(19A)	0.1185	0.2277	0.3358	0.060
H(19B)	0.1568	0.1875	0.3863	0.060
H(19C)	0.0899	0.1084	0.3291	0.060
H(18A)	0.2572	0.0445	0.3238	0.060
H(18B)	0.3201	0.1196	0.3825	0.060
H(18C)	0.3959	0.1234	0.3320	0.060
H(17A)	0.2010	0.1081	0.2479	0.054
H(17B)	0.3374	0.1894	0.2529	0.054
H(17C)	0.2259	0.2267	0.2548	0.054
H(23)	1.0292	0.7042	0.4496	0.023
H(25)	0.8453	0.6159	0.5717	0.027
H(26)	0.6640	0.5893	0.5200	0.026
H(27A)	1.0191	0.5776	0.5956	0.046
H(27B)	1.0224	0.6867	0.6266	0.046
H(27C)	1.1464	0.6671	0.6242	0.046
H(28A)	1.1687	0.8266	0.5311	0.057
H(28B)	1.2300	0.8206	0.5881	0.057
H(28C)	1.1041	0.8377	0.5846	0.057
H(29A)	1.1077	0.5610	0.5080	0.054
H(29B)	1.2317	0.6483	0.5417	0.054
H(29C)	1.1732	0.6613	0.4860	0.054
H(30)	0.5820	−0.1405	0.1500	0.027
H(31)	0.2651	0.4873	0.2676	0.026
H(32)	0.3457	0.6444	0.4146	0.027
H(33A)	0.3674	0.4570	0.1941	0.049
H(33B)	0.4341	0.4430	0.2475	0.049
H(33C)	0.4929	0.5450	0.2261	0.049
H(81A)	0.3910	−0.3001	0.1272	0.065
H(81B)	0.5188	−0.2952	0.1547	0.065
H(81C)	0.4173	−0.4025	0.1217	0.065
H(90A)	0.6108	−0.3720	0.0237	0.065
H(90B)	0.5497	−0.4368	0.0665	0.065
H(90C)	0.6652	−0.3314	0.0874	0.065
H(99A)	0.4216	−0.3564	−0.0126	0.065
H(99B)	0.3358	−0.3332	0.0303	0.065
H(99C)	0.3576	−0.4379	0.0220	0.065
H(35A)	0.1599	0.5813	0.3236	0.042
H(35B)	0.1422	0.5986	0.3868	0.042
H(35C)	0.1787	0.5058	0.3589	0.042

Continued on next page

Table S37. – continued from previous page

atom	x	y	x	U(eq)
H(34A)	0.3926	0.6768	0.2396	0.047
H(34B)	0.2639	0.6466	0.2619	0.047
H(34C)	0.2742	0.5872	0.2028	0.047
H(39A)	0.8531	−0.0479	0.1737	0.048
H(39B)	0.9047	−0.0337	0.2362	0.048
H(39C)	0.9473	0.0614	0.2082	0.048
H(36A)	0.3260	0.7637	0.3398	0.059
H(36B)	0.4397	0.7976	0.3869	0.059
H(36C)	0.3074	0.7848	0.4028	0.059
H(40)	0.5356	0.1112	0.1684	0.031
H(41)	0.9544	0.6040	0.3390	0.038
H(42)	0.9430	0.8349	0.3292	0.040
H(43A)	0.9223	0.5730	0.2438	0.076
H(43B)	0.9598	0.6924	0.2693	0.076
H(43C)	0.8202	0.6211	0.2460	0.076
H(44A)	0.7031	0.4902	0.2955	0.057
H(44B)	0.7653	0.4848	0.3525	0.057
H(44C)	0.8077	0.4449	0.2964	0.057
H(45A)	1.0938	0.7702	0.3533	0.067
H(45B)	1.1125	0.8378	0.4141	0.067
H(45C)	1.1410	0.8921	0.3641	0.067
H(46A)	1.0014	0.9765	0.4020	0.069
H(46B)	0.9514	0.9044	0.4432	0.069
H(46C)	0.8584	0.9100	0.3963	0.069
H(47A)	0.9299	0.0140	0.0370	0.054
H(47B)	0.9579	0.0229	0.1012	0.054
H(47C)	1.0356	−0.0183	0.0599	0.054
H(48)	1.1253	0.1417	0.0407	0.030
H(52)	0.4169	0.7359	0.5084	0.036
H(63A)	1.0784	0.2545	−0.1677	0.062
H(63B)	1.0892	0.2059	−0.1166	0.062
H(63C)	1.0856	0.3177	−0.1069	0.062
H(64A)	0.8966	0.1100	−0.2132	0.062
H(64B)	0.7828	0.0674	−0.1817	0.062
H(64C)	0.9081	0.0554	−0.1648	0.062
H(66)	0.4653	0.2037	0.1113	0.048
H(67)	0.4704	0.3673	0.1476	0.058
H(72A)	1.2220	0.1144	0.1184	0.070
H(72B)	1.1506	0.1688	0.1569	0.070
H(72C)	1.2489	0.2330	0.1237	0.070
H(71)	0.6756	−0.0484	−0.0138	0.024
H(68A)	0.8714	0.1582	0.2877	0.052
H(68B)	0.8356	0.0570	0.3106	0.052
H(68C)	0.7347	0.1028	0.2990	0.052
H(75A)	0.6822	0.2661	0.2248	0.060
H(75B)	0.6650	0.2138	0.2758	0.060
H(75C)	0.5534	0.2318	0.2469	0.060
H(76A)	0.8868	0.2785	−0.1947	0.094
H(76B)	0.8937	0.3396	−0.1334	0.094
H(76C)	0.7735	0.2419	−0.1620	0.094
H(77)	0.8232	0.4903	0.1269	0.055

Continued on next page

Table S37. – continued from previous page

atom	x	y	x	U(eq)
H(78)	0.6498	0.5107	0.1573	0.063
H(97)	0.5851	−0.2191	−0.0119	0.027
H(83)	1.0359	0.2388	−0.0346	0.031
H(85)	0.6897	0.1313	−0.1098	0.038
H(86)	0.6173	0.0999	−0.0296	0.037
H(92A)	0.3514	−0.3076	0.0933	0.065
H(92B)	0.4561	−0.2827	0.1432	0.065
H(92C)	0.3839	−0.3980	0.1091	0.065
H(79A)	0.3947	−0.3563	−0.0049	0.065
H(79B)	0.4151	−0.4504	0.0128	0.065
H(79C)	0.5203	−0.3719	−0.0114	0.065
H(80A)	0.5754	−0.4196	0.0881	0.065
H(80B)	0.6413	−0.3092	0.1289	0.065
H(80C)	0.6761	−0.3296	0.0685	0.065

Table S38. Anisotropic displacement parameters (Å^2) for [{PC(sp^3)(OPhP)}^{tBu}PdI] (**11**). The anisotropic displacement factor exponent takes the form: $-2\pi^2[h^2a^{*2}U_{11} + ... + 2hka^*b^*U_{12}]$.

atom	U_{11}	U_{22}	U_{33}	U_{23}	U_{13}	U_{12}
Pd(1)	0.0100(5)	0.0099(5)	0.0099(6)	0.0017(4)	0.0019(3)	0.0040(4)
I(1)	0.0162(5)	0.0185(6)	0.0155(5)	0.0004(4)	−0.0027(3)	0.0007(4)
P(1)	0.0139(5)	0.0187(5)	0.0186(5)	0.0052(4)	0.0002(4)	0.0043(4)
O(1)	0.0246(17)	0.0311(18)	0.0338(18)	0.0039(14)	0.0036(14)	0.0126(14)
C(62)	0.056(4)	0.021(3)	0.066(4)	0.013(3)	0.026(3)	0.011(2)
C(65)	0.037(3)	0.032(3)	0.041(3)	0.006(2)	0.001(2)	0.017(2)
C(60)	0.034(3)	0.028(3)	0.040(3)	0.002(2)	0.010(2)	0.000(2)
C(59)	0.024(2)	0.027(2)	0.022(2)	0.0019(19)	0.0053(18)	0.0017(19)
C(58)	0.035(3)	0.033(3)	0.027(2)	0.004(2)	0.003(2)	0.018(2)
C(57)	0.024(2)	0.037(3)	0.042(3)	−0.002(2)	0.010(2)	0.006(2)
C(56)	0.028(3)	0.025(2)	0.038(3)	0.004(2)	0.004(2)	0.010(2)
C(55)	0.035(3)	0.024(3)	0.051(3)	0.005(2)	0.000(2)	0.009(2)
C(54)	0.048(3)	0.029(3)	0.047(3)	−0.006(2)	−0.007(3)	0.022(3)
C(53)	0.036(3)	0.041(3)	0.034(3)	0.002(2)	0.002(2)	0.023(2)
C(61)	0.031(3)	0.042(3)	0.057(4)	0.003(3)	0.019(3)	−0.003(2)
I	0.0280(2)	0.0650(3)	0.0559(2)	0.0477(2)	−0.0039(2)	−0.0016(2)
O	0.0193(15)	0.0217(15)	0.0262(16)	0.0024(13)	0.0024(12)	0.0079(12)
Pd	0.0130(2)	0.0204(2)	0.0211(2)	0.0097(1)	−0.0001(1)	0.0027(1)
P(2)	0.0134(5)	0.0260(6)	0.0196(6)	0.0099(5)	0.0013(4)	0.0030(4)
P(4)	0.0177(5)	0.0173(5)	0.0205(6)	−0.0034(4)	0.0051(4)	0.0006(4)
C(10)	0.029(2)	0.017(2)	0.034(3)	0.0033(19)	0.005(2)	0.0042(19)
C(11)	0.014(2)	0.017(2)	0.018(2)	0.0048(16)	0.0055(16)	0.0049(16)
C	0.014(2)	0.019(2)	0.019(2)	0.0049(17)	0.0041(16)	0.0047(17)
C(12)	0.017(2)	0.018(2)	0.019(2)	0.0050(16)	0.0067(16)	0.0069(17)
C(13)	0.017(2)	0.024(2)	0.021(2)	0.0035(18)	0.0046(17)	0.0064(18)
C(16)	0.016(2)	0.029(2)	0.025(2)	0.0097(19)	0.0030(17)	0.0085(18)
C(15)	0.027(2)	0.020(2)	0.030(2)	0.0095(19)	0.0071(19)	0.0106(19)
C(14)	0.020(2)	0.021(2)	0.025(2)	0.0052(18)	0.0060(18)	0.0058(18)
C(20)	0.016(2)	0.027(2)	0.023(2)	0.0054(18)	−0.0010(17)	0.0042(18)
C(19)	0.030(3)	0.024(3)	0.051(3)	−0.001(2)	0.010(2)	−0.003(2)
C(18)	0.044(3)	0.021(2)	0.048(3)	0.006(2)	−0.004(2)	0.007(2)
C(17)	0.043(3)	0.023(2)	0.034(3)	−0.003(2)	0.003(2)	0.010(2)
C(21)	0.019(2)	0.017(2)	0.016(2)	0.0032(16)	0.0006(16)	0.0074(17)
C(22)	0.016(2)	0.019(2)	0.018(2)	0.0040(17)	0.0025(16)	0.0067(17)
C(23)	0.015(2)	0.021(2)	0.021(2)	0.0050(17)	0.0056(17)	0.0053(17)
C(24)	0.018(2)	0.019(2)	0.018(2)	0.0027(17)	0.0003(17)	0.0062(17)
C(25)	0.023(2)	0.026(2)	0.018(2)	0.0066(18)	0.0016(17)	0.0085(19)
C(26)	0.016(2)	0.026(2)	0.025(2)	0.0085(18)	0.0053(17)	0.0081(18)
C(27)	0.023(2)	0.046(3)	0.025(2)	0.012(2)	0.0005(19)	0.013(2)
C(28)	0.031(3)	0.034(3)	0.035(3)	0.005(2)	−0.012(2)	0.002(2)
C(29)	0.022(2)	0.056(3)	0.032(3)	0.005(2)	0.001(2)	0.021(2)
C(30)	0.021(2)	0.022(2)	0.017(2)	0.0028(17)	0.0022(17)	0.0021(18)
C(31)	0.017(2)	0.024(2)	0.021(2)	0.0055(18)	−0.0012(17)	0.0045(18)
C(32)	0.023(2)	0.024(2)	0.022(2)	0.0043(18)	0.0007(18)	0.0121(19)
C(33)	0.035(3)	0.042(3)	0.023(2)	0.005(2)	0.006(2)	0.017(2)
C(81)	0.052(3)	0.0198(19)	0.048(3)	0.005(2)	0.014(3)	0.002(2)
C(90)	0.052(3)	0.0198(19)	0.048(3)	0.005(2)	0.014(3)	0.002(2)
C(99)	0.052(3)	0.0198(19)	0.048(3)	0.005(2)	0.014(3)	0.002(2)
C(35)	0.021(2)	0.035(3)	0.028(2)	0.003(2)	0.0044(19)	0.011(2)

Continued on next page

Table S38. – continued from previous page

atom	U_{11}	U_{22}	U_{33}	U_{23}	U_{13}	U_{12}
C(34)	0.027(2)	0.043(3)	0.025(2)	0.014(2)	−0.001(2)	0.012(2)
C(39)	0.029(3)	0.036(3)	0.029(3)	0.009(2)	0.004(2)	0.011(2)
C(36)	0.037(3)	0.036(3)	0.045(3)	0.002(2)	−0.003(2)	0.020(2)
C(40)	0.019(2)	0.029(2)	0.024(2)	−0.0004(19)	0.0057(18)	0.0064(19)
C(41)	0.027(2)	0.051(3)	0.018(2)	0.006(2)	0.0054(19)	0.016(2)
C(42)	0.021(2)	0.036(3)	0.038(3)	0.019(2)	0.003(2)	0.000(2)
C(43)	0.055(4)	0.071(4)	0.025(3)	0.012(3)	0.015(3)	0.021(3)
C(44)	0.042(3)	0.037(3)	0.034(3)	0.003(2)	0.010(2)	0.017(2)
C(45)	0.021(3)	0.051(3)	0.053(3)	0.024(3)	0.004(2)	0.000(2)
C(46)	0.032(3)	0.031(3)	0.066(4)	0.018(3)	0.008(3)	−0.003(2)
C(47)	0.039(3)	0.036(3)	0.038(3)	0.010(2)	0.009(2)	0.019(2)
C(48)	0.022(2)	0.033(3)	0.021(2)	0.0053(19)	0.0062(18)	0.013(2)
C(51)	0.026(2)	0.023(2)	0.026(2)	0.0038(18)	−0.0023(19)	0.0122(19)
C(52)	0.026(2)	0.034(3)	0.031(3)	0.005(2)	0.001(2)	0.015(2)
C(63)	0.043(3)	0.040(3)	0.036(3)	0.011(2)	0.019(2)	0.007(2)
C(64)	0.037(3)	0.052(3)	0.034(3)	0.005(2)	0.009(2)	0.017(3)
C(66)	0.038(3)	0.048(3)	0.035(3)	0.001(2)	0.006(2)	0.024(3)
C(67)	0.053(4)	0.057(4)	0.044(3)	−0.003(3)	0.005(3)	0.037(3)
C(72)	0.040(3)	0.053(3)	0.046(3)	0.004(3)	−0.003(3)	0.022(3)
C(71)	0.018(2)	0.022(2)	0.015(2)	0.0017(17)	0.0020(16)	0.0023(17)
C(70)	0.0106(19)	0.018(2)	0.023(2)	0.0009(17)	0.0010(16)	0.0040(16)
C(69)	0.015(2)	0.018(2)	0.019(2)	−0.0005(17)	0.0036(16)	0.0032(17)
C(68)	0.028(3)	0.039(3)	0.025(3)	0.001(2)	0.002(2)	0.004(2)
C(75)	0.033(3)	0.037(3)	0.045(3)	−0.001(2)	0.012(2)	0.012(2)
C(74)	0.032(3)	0.017(2)	0.030(2)	0.0014(19)	0.007(2)	0.0045(19)
C(73)	0.018(2)	0.019(2)	0.025(2)	0.0019(18)	0.0036(17)	0.0055(17)
C(76)	0.090(5)	0.084(5)	0.064(4)	0.053(4)	0.052(4)	0.065(4)
C(77)	0.052(3)	0.034(3)	0.048(3)	0.002(2)	−0.003(3)	0.017(3)
C(78)	0.059(4)	0.045(3)	0.052(4)	−0.005(3)	−0.004(3)	0.029(3)
C(97)	0.021(2)	0.018(2)	0.019(2)	−0.0053(17)	0.0016(17)	0.0010(18)
C(91)	0.013(2)	0.015(2)	0.027(2)	0.0019(17)	0.0065(17)	0.0035(16)
C(98)	0.021(2)	0.017(2)	0.030(2)	0.0071(18)	0.0051(18)	0.0078(18)
C(82)	0.020(2)	0.018(2)	0.030(2)	0.0012(18)	0.0094(19)	0.0042(18)
C(83)	0.024(2)	0.018(2)	0.029(2)	−0.0005(18)	0.012(2)	0.0016(18)
C(84)	0.031(2)	0.016(2)	0.035(3)	0.0094(19)	0.015(2)	0.0119(19)
C(85)	0.029(3)	0.041(3)	0.032(3)	0.018(2)	0.005(2)	0.016(2)
C(86)	0.019(2)	0.040(3)	0.040(3)	0.021(2)	0.008(2)	0.010(2)
C(87)	0.036(3)	0.033(3)	0.035(3)	0.016(2)	0.014(2)	0.020(2)
C(92)	0.052(3)	0.0198(19)	0.048(3)	0.005(2)	0.014(3)	0.002(2)
C(79)	0.052(3)	0.0198(19)	0.048(3)	0.005(2)	0.014(3)	0.002(2)
C(80)	0.052(3)	0.0198(19)	0.048(3)	0.005(2)	0.014(3)	0.002(2)
I(3)	0.0474(16)	0.0558(19)	0.0388(15)	−0.0255(11)	0.0112(11)	−0.0270(11)
Pd(91)	0.0244(17)	0.0255(19)	0.036(2)	−0.0128(12)	0.0102(13)	−0.0112(11)
P(3)	0.0213(6)	0.0255(6)	0.0262(6)	0.0000(5)	0.0061(5)	0.0010(5)

Table S39. Distances [Å] for [{PC(sp^3)(OPhP)}^{tBu}PdI] (**11**).

atom – atom	distance	atom – atom	distance
Pd(1) – C(91)	2.130(5)	Pd(1) – P(3)	2.284(3)
Pd(1) – P(4)	2.293(3)	Pd(1) – I(1)	2.681(4)
P(1) – C(12)	1.826(4)	P(1) – C(31)	1.837(4)
P(1) – C(32)	1.851(4)	P(1) – Pd	2.3282(10)
O(1) – C(58)	1.378(5)	O(1) – C(91)	1.466(5)
C(62) – C(60)	1.539(7)	C(62) – H(62A)	0.9800
C(62) – H(62B)	0.9800	C(62) – H(62C)	0.9800
C(65) – C(58)	1.386(7)	C(65) – C(77)	1.389(7)
C(65) – H(65)	0.9500	C(60) – C(61)	1.533(7)
C(60) – P(3)	1.833(5)	C(60) – H(60)	1.0000
C(59) – C(68)	1.525(6)	C(59) – C(39)	1.525(6)
C(59) – P(4)	1.851(5)	C(59) – H(59)	1.0000
C(58) – C(66)	1.392(7)	C(57) – C(40)	1.533(6)
C(57) – H(57A)	0.9800	C(57) – H(57B)	0.9800
C(57) – H(57C)	0.9800	C(56) – C(51)	1.383(6)
C(56) – C(55)	1.395(6)	C(56) – H(56)	0.9500
C(55) – C(54)	1.377(7)	C(55) – H(55)	0.9500
C(54) – C(53)	1.380(7)	C(54) – H(54)	0.9500
C(53) – C(52)	1.385(7)	C(53) – H(53)	0.9500
C(61) – H(61A)	0.9800	C(61) – H(61B)	0.9800
C(61) – H(61C)	0.9800	I – Pd	2.6746(4)
O – C(51)	1.378(5)	O – C	1.477(5)
Pd – C	2.089(4)	Pd – P(2)	2.2741(11)
P(2) – C(22)	1.824(4)	P(2) – C(41)	1.849(5)
P(2) – C(42)	1.853(5)	P(4) – C(69)	1.836(4)
P(4) – C(40)	1.850(4)	P(4) – Pd(91)	2.378(7)
C(10) – C(18)	1.522(6)	C(10) – C(19)	1.527(6)
C(10) – C(17)	1.531(7)	C(10) – C(14)	1.543(6)
C(11) – C(16)	1.387(6)	C(11) – C(12)	1.409(5)
C(11) – C	1.518(5)	C – C(21)	1.528(5)
C(12) – C(13)	1.392(6)	C(13) – C(14)	1.406(6)
C(13) – H(13)	0.9500	C(16) – C(15)	1.393(6)
C(16) – H(16)	0.9500	C(15) – C(14)	1.378(6)
C(15) – H(15)	0.9500	C(20) – C(27)	1.523(6)
C(20) – C(29)	1.529(6)	C(20) – C(28)	1.531(6)
C(20) – C(24)	1.536(6)	C(19) – H(19A)	0.9800
C(19) – H(19B)	0.9800	C(19) – H(19C)	0.9800
C(18) – H(18A)	0.9800	C(18) – H(18B)	0.9800
C(18) – H(18C)	0.9800	C(17) – H(17A)	0.9800
C(17) – H(17B)	0.9800	C(17) – H(17C)	0.9800
C(21) – C(26)	1.391(6)	C(21) – C(22)	1.395(6)
C(22) – C(23)	1.389(6)	C(23) – C(24)	1.390(6)
C(23) – H(23)	0.9500	C(24) – C(25)	1.398(6)
C(25) – C(26)	1.380(6)	C(25) – H(25)	0.9500
C(26) – H(26)	0.9500	C(27) – H(27A)	0.9800
C(27) – H(27B)	0.9800	C(27) – H(27C)	0.9800
C(28) – H(28A)	0.9800	C(28) – H(28B)	0.9800
C(28) – H(28C)	0.9800	C(29) – H(29A)	0.9800
C(29) – H(29B)	0.9800	C(29) – H(29C)	0.9800
C(30) – C(69)	1.389(6)	C(30) – C(73)	1.392(6)

Continued on next page

Table S39. – continued from previous page

atom – atom	distance	atom – atom	distance
C(30) – H(30)	0.9500	C(31) – C(34)	1.528(6)
C(31) – C(33)	1.530(6)	C(31) – H(31)	1.0000
C(32) – C(35)	1.527(6)	C(32) – C(36)	1.530(6)
C(32) – H(32)	1.0000	C(33) – H(33A)	0.9800
C(33) – H(33B)	0.9800	C(33) – H(33C)	0.9800
C(81) – C(74)	1.573(18)	C(81) – H(81A)	0.9800
C(81) – H(81B)	0.9800	C(81) – H(81C)	0.9800
C(90) – C(74)	1.463(16)	C(90) – H(90A)	0.9800
C(90) – H(90B)	0.9800	C(90) – H(90C)	0.9800
C(99) – C(74)	1.568(16)	C(99) – H(99A)	0.9800
C(99) – H(99B)	0.9800	C(99) – H(99C)	0.9800
C(35) – H(35A)	0.9800	C(35) – H(35B)	0.9800
C(35) – H(35C)	0.9800	C(34) – H(34A)	0.9800
C(34) – H(34B)	0.9800	C(34) – H(34C)	0.9800
C(39) – H(39A)	0.9800	C(39) – H(39B)	0.9800
C(39) – H(39C)	0.9800	C(36) – H(36A)	0.9800
C(36) – H(36B)	0.9800	C(36) – H(36C)	0.9800
C(40) – C(75)	1.535(6)	C(40) – H(40)	1.0000
C(41) – C(43)	1.521(7)	C(41) – C(44)	1.525(7)
C(41) – H(41)	1.0000	C(42) – C(46)	1.514(7)
C(42) – C(45)	1.519(7)	C(42) – H(42)	1.0000
C(43) – H(43A)	0.9800	C(43) – H(43B)	0.9800
C(43) – H(43C)	0.9800	C(44) – H(44A)	0.9800
C(44) – H(44B)	0.9800	C(44) – H(44C)	0.9800
C(45) – H(45A)	0.9800	C(45) – H(45B)	0.9800
C(45) – H(45C)	0.9800	C(46) – H(46A)	0.9800
C(46) – H(46B)	0.9800	C(46) – H(46C)	0.9800
C(47) – C(48)	1.518(6)	C(47) – H(47A)	0.9800
C(47) – H(47B)	0.9800	C(47) – H(47C)	0.9800
C(48) – C(72)	1.521(6)	C(48) – P(3)	1.860(4)
C(48) – H(48)	1.0000	C(51) – C(52)	1.387(6)
C(52) – H(52)	0.9500	C(63) – C(87)	1.519(7)
C(63) – H(63A)	0.9800	C(63) – H(63B)	0.9800
C(63) – H(63C)	0.9800	C(64) – C(87)	1.528(7)
C(64) – H(64A)	0.9800	C(64) – H(64B)	0.9800
C(64) – H(64C)	0.9800	C(66) – C(67)	1.389(7)
C(66) – H(66)	0.9500	C(67) – C(78)	1.380(8)
C(67) – H(67)	0.9500	C(72) – H(72A)	0.9800
C(72) – H(72B)	0.9800	C(72) – H(72C)	0.9800
C(71) – C(70)	1.386(5)	C(71) – C(97)	1.389(6)
C(71) – H(71)	0.9500	C(70) – C(69)	1.400(6)
C(70) – C(91)	1.524(5)	C(68) – H(68A)	0.9800
C(68) – H(68B)	0.9800	C(68) – H(68C)	0.9800
C(75) – H(75A)	0.9800	C(75) – H(75B)	0.9800
C(75) – H(75C)	0.9800	C(74) – C(92)	1.471(12)
C(74) – C(79)	1.530(10)	C(74) – C(73)	1.534(6)
C(74) – C(80)	1.550(11)	C(73) – C(97)	1.383(6)
C(76) – C(87)	1.531(7)	C(76) – H(76A)	0.9800
C(76) – H(76B)	0.9800	C(76) – H(76C)	0.9800
C(77) – C(78)	1.388(8)	C(77) – H(77)	0.9500
C(78) – H(78)	0.9500	C(97) – H(97)	0.9500

Continued on next page

Table S39. – continued from previous page

**atom – atom**	**distance**	**atom – atom**	**distance**
C(91) – C(98)	1.521(6)	C(91) – Pd(91)	1.983(9)
C(98) – C(86)	1.389(6)	C(98) – C(82)	1.398(6)
C(82) – C(83)	1.402(6)	C(82) – P(3)	1.838(5)
C(83) – C(84)	1.387(6)	C(83) – H(83)	0.9500
C(84) – C(85)	1.401(6)	C(84) – C(87)	1.529(6)
C(85) – C(86)	1.380(6)	C(85) – H(85)	0.9500
C(86) – H(86)	0.9500	C(92) – H(92A)	0.9800
C(92) – H(92B)	0.9800	C(92) – H(92C)	0.9800
C(79) – H(79A)	0.9800	C(79) – H(79B)	0.9800
C(79) – H(79C)	0.9800	C(80) – H(80A)	0.9800
C(80) – H(80B)	0.9800	C(80) – H(80C)	0.9800
I(3) – Pd(91)	2.678(7)	Pd(91) – P(3)	2.248(6)

Table S40. Angles [°] for [{PC(sp^3)(OPhP)}^{tBu}PdI] (**11**).

atom – atom – atom	angle	atom – atom – atom	angle
C(91) – Pd(1) – P(3)	83.96(15)	C(91) – Pd(1) – P(4)	81.64(16)
P(3) – Pd(1) – P(4)	151.40(16)	C(91) – Pd(1) – I(1)	167.44(16)
P(3) – Pd(1) – I(1)	97.12(13)	P(4) – Pd(1) – I(1)	102.27(12)
C(12) – P(1) – C(31)	104.42(19)	C(12) – P(1) – C(32)	106.14(18)
C(31) – P(1) – C(32)	105.78(19)	C(12) – P(1) – Pd	94.94(13)
C(31) – P(1) – Pd	119.81(14)	C(32) – P(1) – Pd	122.58(14)
C(58) – O(1) – C(91)	121.0(3)	C(60) – C(62) – H(62A)	109.5
C(60) – C(62) – H(62B)	109.5	H(62A) – C(62) – H(62B)	109.5
C(60) – C(62) – H(62C)	109.5	H(62A) – C(62) – H(62C)	109.5
H(62B) – C(62) – H(62C)	109.5	C(58) – C(65) – C(77)	119.2(5)
C(58) – C(65) – H(65)	120.4	C(77) – C(65) – H(65)	120.4
C(61) – C(60) – C(62)	111.4(4)	C(61) – C(60) – P(3)	116.6(3)
C(62) – C(60) – P(3)	109.7(4)	C(61) – C(60) – H(60)	106.1
C(62) – C(60) – H(60)	106.1	P(3) – C(60) – H(60)	106.1
C(68) – C(59) – C(39)	110.6(4)	C(68) – C(59) – P(4)	114.0(3)
C(39) – C(59) – P(4)	110.6(3)	C(68) – C(59) – H(59)	107.1
C(39) – C(59) – H(59)	107.1	P(4) – C(59) – H(59)	107.1
O(1) – C(58) – C(65)	125.1(4)	O(1) – C(58) – C(66)	114.6(4)
C(65) – C(58) – C(66)	120.2(4)	C(40) – C(57) – H(57A)	109.5
C(40) – C(57) – H(57B)	109.5	H(57A) – C(57) – H(57B)	109.5
C(40) – C(57) – H(57C)	109.5	H(57A) – C(57) – H(57C)	109.5
H(57B) – C(57) – H(57C)	109.5	C(51) – C(56) – C(55)	119.5(4)
C(51) – C(56) – H(56)	120.3	C(55) – C(56) – H(56)	120.3
C(54) – C(55) – C(56)	120.9(5)	C(54) – C(55) – H(55)	119.5
C(56) – C(55) – H(55)	119.5	C(55) – C(54) – C(53)	119.1(5)
C(55) – C(54) – H(54)	120.5	C(53) – C(54) – H(54)	120.5
C(54) – C(53) – C(52)	120.8(5)	C(54) – C(53) – H(53)	119.6
C(52) – C(53) – H(53)	119.6	C(60) – C(61) – H(61A)	109.5
C(60) – C(61) – H(61B)	109.5	H(61A) – C(61) – H(61B)	109.5
C(60) – C(61) – H(61C)	109.5	H(61A) – C(61) – H(61C)	109.5
H(61B) – C(61) – H(61C)	109.5	C(51) – O – C	121.8(3)
C – Pd – P(2)	85.27(11)	C – Pd – P(1)	81.33(11)
P(2) – Pd – P(1)	157.20(4)	C – Pd – I	175.82(11)
P(2) – Pd – I	93.57(3)	P(1) – Pd – I	101.02(3)
C(22) – P(2) – C(41)	103.7(2)	C(22) – P(2) – C(42)	110.6(2)
C(41) – P(2) – C(42)	105.2(2)	C(22) – P(2) – Pd	102.73(13)
C(41) – P(2) – Pd	115.15(16)	C(42) – P(2) – Pd	118.54(16)
C(69) – P(4) – C(40)	106.09(19)	C(69) – P(4) – C(59)	103.16(19)
C(40) – P(4) – C(59)	107.3(2)	C(69) – P(4) – Pd(1)	97.42(15)
C(40) – P(4) – Pd(1)	124.42(16)	C(59) – P(4) – Pd(1)	115.32(16)
C(69) – P(4) – Pd(91)	95.9(2)	C(40) – P(4) – Pd(91)	120.5(2)
C(59) – P(4) – Pd(91)	120.6(3)	C(18) – C(10) – C(19)	108.7(4)
C(18) – C(10) – C(17)	109.0(4)	C(19) – C(10) – C(17)	109.1(4)
C(18) – C(10) – C(14)	112.0(4)	C(19) – C(10) – C(14)	108.9(4)
C(17) – C(10) – C(14)	109.1(4)	C(16) – C(11) – C(12)	117.6(4)
C(16) – C(11) – C	124.8(4)	C(12) – C(11) – C	117.6(3)
O – C – C(11)	101.7(3)	O – C – C(21)	108.1(3)
C(11) – C – C(21)	114.8(3)	O – C – Pd	111.3(2)
C(11) – C – Pd	105.3(3)	C(21) – C – Pd	114.8(3)
C(13) – C(12) – C(11)	120.4(4)	C(13) – C(12) – P(1)	125.0(3)

Continued on next page

Table S40. – continued from previous page

atom – atom – atom	angle	atom – atom – atom	angle
C(11) – C(12) – P(1)	113.9(3)	C(12) – C(13) – C(14)	121.8(4)
C(12) – C(13) – H(13)	119.1	C(14) – C(13) – H(13)	119.1
C(11) – C(16) – C(15)	121.2(4)	C(11) – C(16) – H(16)	119.4
C(15) – C(16) – H(16)	119.4	C(14) – C(15) – C(16)	122.2(4)
C(14) – C(15) – H(15)	118.9	C(16) – C(15) – H(15)	118.9
C(15) – C(14) – C(13)	116.8(4)	C(15) – C(14) – C(10)	123.2(4)
C(13) – C(14) – C(10)	119.9(4)	C(27) – C(20) – C(29)	107.5(4)
C(27) – C(20) – C(28)	109.0(4)	C(29) – C(20) – C(28)	109.4(4)
C(27) – C(20) – C(24)	112.4(3)	C(29) – C(20) – C(24)	110.4(3)
C(28) – C(20) – C(24)	108.1(3)	C(10) – C(19) – H(19A)	109.5
C(10) – C(19) – H(19B)	109.5	H(19A) – C(19) – H(19B)	109.5
C(10) – C(19) – H(19C)	109.5	H(19A) – C(19) – H(19C)	109.5
H(19B) – C(19) – H(19C)	109.5	C(10) – C(18) – H(18A)	109.5
C(10) – C(18) – H(18B)	109.5	H(18A) – C(18) – H(18B)	109.5
C(10) – C(18) – H(18C)	109.5	H(18A) – C(18) – H(18C)	109.5
H(18B) – C(18) – H(18C)	109.5	C(10) – C(17) – H(17A)	109.5
C(10) – C(17) – H(17B)	109.5	H(17A) – C(17) – H(17B)	109.5
C(10) – C(17) – H(17C)	109.5	H(17A) – C(17) – H(17C)	109.5
H(17B) – C(17) – H(17C)	109.5	C(26) – C(21) – C(22)	118.1(4)
C(26) – C(21) – C	120.9(4)	C(22) – C(21) – C	121.0(3)
C(23) – C(22) – C(21)	120.2(4)	C(23) – C(22) – P(2)	125.3(3)
C(21) – C(22) – P(2)	114.4(3)	C(22) – C(23) – C(24)	122.3(4)
C(22) – C(23) – H(23)	118.8	C(24) – C(23) – H(23)	118.8
C(23) – C(24) – C(25)	116.4(4)	C(23) – C(24) – C(20)	120.2(4)
C(25) – C(24) – C(20)	123.3(4)	C(26) – C(25) – C(24)	122.0(4)
C(26) – C(25) – H(25)	119.0	C(24) – C(25) – H(25)	119.0
C(25) – C(26) – C(21)	120.9(4)	C(25) – C(26) – H(26)	119.6
C(21) – C(26) – H(26)	119.6	C(20) – C(27) – H(27A)	109.5
C(20) – C(27) – H(27B)	109.5	H(27A) – C(27) – H(27B)	109.5
C(20) – C(27) – H(27C)	109.5	H(27A) – C(27) – H(27C)	109.5
H(27B) – C(27) – H(27C)	109.5	C(20) – C(28) – H(28A)	109.5
C(20) – C(28) – H(28B)	109.5	H(28A) – C(28) – H(28B)	109.5
C(20) – C(28) – H(28C)	109.5	H(28A) – C(28) – H(28C)	109.5
H(28B) – C(28) – H(28C)	109.5	C(20) – C(29) – H(29A)	109.5
C(20) – C(29) – H(29B)	109.5	H(29A) – C(29) – H(29B)	109.5
C(20) – C(29) – H(29C)	109.5	H(29A) – C(29) – H(29C)	109.5
H(29B) – C(29) – H(29C)	109.5	C(69) – C(30) – C(73)	121.8(4)
C(69) – C(30) – H(30)	119.1	C(73) – C(30) – H(30)	119.1
C(34) – C(31) – C(33)	110.6(4)	C(34) – C(31) – P(1)	113.1(3)
C(33) – C(31) – P(1)	110.7(3)	C(34) – C(31) – H(31)	107.4
C(33) – C(31) – H(31)	107.4	P(1) – C(31) – H(31)	107.4
C(35) – C(32) – C(36)	114.0(4)	C(35) – C(32) – P(1)	113.4(3)
C(36) – C(32) – P(1)	111.7(3)	C(35) – C(32) – H(32)	105.6
C(36) – C(32) – H(32)	105.6	P(1) – C(32) – H(32)	105.6
C(31) – C(33) – H(33A)	109.5	C(31) – C(33) – H(33B)	109.5
H(33A) – C(33) – H(33B)	109.5	C(31) – C(33) – H(33C)	109.5
H(33A) – C(33) – H(33C)	109.5	H(33B) – C(33) – H(33C)	109.5
C(74) – C(81) – H(81A)	109.5	C(74) – C(81) – H(81B)	109.5
H(81A) – C(81) – H(81B)	109.5	C(74) – C(81) – H(81C)	109.5
H(81A) – C(81) – H(81C)	109.5	H(81B) – C(81) – H(81C)	109.5
C(74) – C(90) – H(90A)	109.5	C(74) – C(90) – H(90B)	109.5

Continued on next page

Table S40. – continued from previous page

atom – atom – atom	angle	atom – atom – atom	angle
H(90A) – C(90) – H(90B)	109.5	C(74) – C(90) – H(90C)	109.5
H(90A) – C(90) – H(90C)	109.5	H(90B) – C(90) – H(90C)	109.5
C(74) – C(99) – H(99A)	109.5	C(74) – C(99) – H(99B)	109.5
H(99A) – C(99) – H(99B)	109.5	C(74) – C(99) – H(99C)	109.5
H(99A) – C(99) – H(99C)	109.5	H(99B) – C(99) – H(99C)	109.5
C(32) – C(35) – H(35A)	109.5	C(32) – C(35) – H(35B)	109.5
H(35A) – C(35) – H(35B)	109.5	C(32) – C(35) – H(35C)	109.5
H(35A) – C(35) – H(35C)	109.5	H(35B) – C(35) – H(35C)	109.5
C(31) – C(34) – H(34A)	109.5	C(31) – C(34) – H(34B)	109.5
H(34A) – C(34) – H(34B)	109.5	C(31) – C(34) – H(34C)	109.5
H(34A) – C(34) – H(34C)	109.5	H(34B) – C(34) – H(34C)	109.5
C(59) – C(39) – H(39A)	109.5	C(59) – C(39) – H(39B)	109.5
H(39A) – C(39) – H(39B)	109.5	C(59) – C(39) – H(39C)	109.5
H(39A) – C(39) – H(39C)	109.5	H(39B) – C(39) – H(39C)	109.5
C(32) – C(36) – H(36A)	109.5	C(32) – C(36) – H(36B)	109.5
H(36A) – C(36) – H(36B)	109.5	C(32) – C(36) – H(36C)	109.5
H(36A) – C(36) – H(36C)	109.5	H(36B) – C(36) – H(36C)	109.5
C(57) – C(40) – C(75)	112.4(4)	C(57) – C(40) – P(4)	113.7(3)
C(75) – C(40) – P(4)	110.8(3)	C(57) – C(40) – H(40)	106.5
C(75) – C(40) – H(40)	106.5	P(4) – C(40) – H(40)	106.5
C(43) – C(41) – C(44)	110.7(4)	C(43) – C(41) – P(2)	113.5(4)
C(44) – C(41) – P(2)	110.2(3)	C(43) – C(41) – H(41)	107.4
C(44) – C(41) – H(41)	107.4	P(2) – C(41) – H(41)	107.4
C(46) – C(42) – C(45)	110.7(4)	C(46) – C(42) – P(2)	111.6(3)
C(45) – C(42) – P(2)	116.8(4)	C(46) – C(42) – H(42)	105.6
C(45) – C(42) – H(42)	105.6	P(2) – C(42) – H(42)	105.6
C(41) – C(43) – H(43A)	109.5	C(41) – C(43) – H(43B)	109.5
H(43A) – C(43) – H(43B)	109.5	C(41) – C(43) – H(43C)	109.5
H(43A) – C(43) – H(43C)	109.5	H(43B) – C(43) – H(43C)	109.5
C(41) – C(44) – H(44A)	109.5	C(41) – C(44) – H(44B)	109.5
H(44A) – C(44) – H(44B)	109.5	C(41) – C(44) – H(44C)	109.5
H(44A) – C(44) – H(44C)	109.5	H(44B) – C(44) – H(44C)	109.5
C(42) – C(45) – H(45A)	109.5	C(42) – C(45) – H(45B)	109.5
H(45A) – C(45) – H(45B)	109.5	C(42) – C(45) – H(45C)	109.5
H(45A) – C(45) – H(45C)	109.5	H(45B) – C(45) – H(45C)	109.5
C(42) – C(46) – H(46A)	109.5	C(42) – C(46) – H(46B)	109.5
H(46A) – C(46) – H(46B)	109.5	C(42) – C(46) – H(46C)	109.5
H(46A) – C(46) – H(46C)	109.5	H(46B) – C(46) – H(46C)	109.5
C(48) – C(47) – H(47A)	109.5	C(48) – C(47) – H(47B)	109.5
H(47A) – C(47) – H(47B)	109.5	C(48) – C(47) – H(47C)	109.5
H(47A) – C(47) – H(47C)	109.5	H(47B) – C(47) – H(47C)	109.5
C(47) – C(48) – C(72)	111.0(4)	C(47) – C(48) – P(3)	109.8(3)
C(72) – C(48) – P(3)	112.3(3)	C(47) – C(48) – H(48)	107.9
C(72) – C(48) – H(48)	107.9	P(3) – C(48) – H(48)	107.9
O – C(51) – C(56)	125.2(4)	O – C(51) – C(52)	115.0(4)
C(56) – C(51) – C(52)	119.8(4)	C(53) – C(52) – C(51)	119.9(5)
C(53) – C(52) – H(52)	120.1	C(51) – C(52) – H(52)	120.1
C(87) – C(63) – H(63A)	109.5	C(87) – C(63) – H(63B)	109.5
H(63A) – C(63) – H(63B)	109.5	C(87) – C(63) – H(63C)	109.5
H(63A) – C(63) – H(63C)	109.5	H(63B) – C(63) – H(63C)	109.5
C(87) – C(64) – H(64A)	109.5	C(87) – C(64) – H(64B)	109.5

Continued on next page

Table S40. – continued from previous page

atom – atom – atom	angle	atom – atom – atom	angle
H(64A) – C(64) – H(64B)	109.5	C(87) – C(64) – H(64C)	109.5
H(64A) – C(64) – H(64C)	109.5	H(64B) – C(64) – H(64C)	109.5
C(67) – C(66) – C(58)	119.5(5)	C(67) – C(66) – H(66)	120.3
C(58) – C(66) – H(66)	120.3	C(78) – C(67) – C(66)	120.9(5)
C(78) – C(67) – H(67)	119.5	C(66) – C(67) – H(67)	119.5
C(48) – C(72) – H(72A)	109.5	C(48) – C(72) – H(72B)	109.5
H(72A) – C(72) – H(72B)	109.5	C(48) – C(72) – H(72C)	109.5
H(72A) – C(72) – H(72C)	109.5	H(72B) – C(72) – H(72C)	109.5
C(70) – C(71) – C(97)	120.7(4)	C(70) – C(71) – H(71)	119.6
C(97) – C(71) – H(71)	119.6	C(71) – C(70) – C(69)	117.6(4)
C(71) – C(70) – C(91)	124.9(4)	C(69) – C(70) – C(91)	117.5(3)
C(30) – C(69) – C(70)	120.7(4)	C(30) – C(69) – P(4)	124.6(3)
C(70) – C(69) – P(4)	114.4(3)	C(59) – C(68) – H(68A)	109.5
C(59) – C(68) – H(68B)	109.5	H(68A) – C(68) – H(68B)	109.5
C(59) – C(68) – H(68C)	109.5	H(68A) – C(68) – H(68C)	109.5
H(68B) – C(68) – H(68C)	109.5	C(40) – C(75) – H(75A)	109.5
C(40) – C(75) – H(75B)	109.5	H(75A) – C(75) – H(75B)	109.5
C(40) – C(75) – H(75C)	109.5	H(75A) – C(75) – H(75C)	109.5
H(75B) – C(75) – H(75C)	109.5	C(90) – C(74) – C(92)	125.8(8)
C(90) – C(74) – C(79)	79.6(8)	C(92) – C(74) – C(79)	112.5(6)
C(90) – C(74) – C(73)	113.1(6)	C(92) – C(74) – C(73)	110.4(5)
C(79) – C(74) – C(73)	111.4(5)	C(92) – C(74) – C(80)	108.2(6)
C(79) – C(74) – C(80)	107.1(6)	C(73) – C(74) – C(80)	106.9(4)
C(90) – C(74) – C(99)	109.0(9)	C(92) – C(74) – C(99)	84.8(7)
C(73) – C(74) – C(99)	109.3(6)	C(80) – C(74) – C(99)	133.8(7)
C(90) – C(74) – C(81)	107.9(9)	C(79) – C(74) – C(81)	126.8(8)
C(73) – C(74) – C(81)	112.8(7)	C(80) – C(74) – C(81)	86.7(8)
C(99) – C(74) – C(81)	104.3(9)	C(97) – C(73) – C(30)	116.7(4)
C(97) – C(73) – C(74)	121.6(4)	C(30) – C(73) – C(74)	121.8(4)
C(87) – C(76) – H(76A)	109.5	C(87) – C(76) – H(76B)	109.5
H(76A) – C(76) – H(76B)	109.5	C(87) – C(76) – H(76C)	109.5
H(76A) – C(76) – H(76C)	109.5	H(76B) – C(76) – H(76C)	109.5
C(78) – C(77) – C(65)	121.1(5)	C(78) – C(77) – H(77)	119.5
C(65) – C(77) – H(77)	119.5	C(67) – C(78) – C(77)	119.0(5)
C(67) – C(78) – H(78)	120.5	C(77) – C(78) – H(78)	120.5
C(73) – C(97) – C(71)	122.4(4)	C(73) – C(97) – H(97)	118.8
C(71) – C(97) – H(97)	118.8	O(1) – C(91) – C(98)	110.1(3)
O(1) – C(91) – C(70)	101.0(3)	C(98) – C(91) – C(70)	114.0(3)
O(1) – C(91) – Pd(91)	105.9(3)	C(98) – C(91) – Pd(91)	112.9(3)
C(70) – C(91) – Pd(91)	111.9(4)	O(1) – C(91) – Pd(1)	108.4(3)
C(98) – C(91) – Pd(1)	115.2(3)	C(70) – C(91) – Pd(1)	107.1(3)
C(86) – C(98) – C(82)	118.3(4)	C(86) – C(98) – C(91)	121.3(4)
C(82) – C(98) – C(91)	120.3(4)	C(98) – C(82) – C(83)	120.0(4)
C(98) – C(82) – P(3)	114.5(3)	C(83) – C(82) – P(3)	125.4(3)
C(84) – C(83) – C(82)	122.3(4)	C(84) – C(83) – H(83)	118.8
C(82) – C(83) – H(83)	118.8	C(83) – C(84) – C(85)	115.9(4)
C(83) – C(84) – C(87)	122.7(4)	C(85) – C(84) – C(87)	121.4(4)
C(86) – C(85) – C(84)	123.0(4)	C(86) – C(85) – H(85)	118.5
C(84) – C(85) – H(85)	118.5	C(85) – C(86) – C(98)	120.3(4)
C(85) – C(86) – H(86)	119.8	C(98) – C(86) – H(86)	119.8
C(63) – C(87) – C(64)	107.5(4)	C(63) – C(87) – C(84)	112.9(4)

Continued on next page

Table S40. – continued from previous page

atom – atom – atom	angle	atom – atom – atom	angle
C(64) – C(87) – C(84)	108.9(4)	C(63) – C(87) – C(76)	109.4(4)
C(64) – C(87) – C(76)	108.5(5)	C(84) – C(87) – C(76)	109.6(4)
C(74) – C(92) – H(92A)	109.5	C(74) – C(92) – H(92B)	109.5
C(74) – C(92) – H(92C)	109.5	C(74) – C(79) – H(79A)	109.5
C(74) – C(79) – H(79B)	109.5	C(74) – C(79) – H(79C)	109.5
C(74) – C(80) – H(80A)	109.5	C(74) – C(80) – H(80B)	109.5
C(74) – C(80) – H(80C)	109.5	C(91) – Pd(91) – P(3)	88.4(3)
C(91) – Pd(91) – P(4)	82.6(2)	P(3) – Pd(91) – P(4)	146.9(3)
C(91) – Pd(91) – I(3)	171.3(3)	P(3) – Pd(91) – I(3)	94.6(2)
P(4) – Pd(91) – I(3)	99.1(2)	C(60) – P(3) – C(82)	109.6(2)
C(60) – P(3) – C(48)	104.4(2)	C(82) – P(3) – C(48)	105.92(19)
C(60) – P(3) – Pd(91)	120.6(2)	C(82) – P(3) – Pd(91)	98.6(3)
C(48) – P(3) – Pd(91)	116.6(3)	C(60) – P(3) – Pd(1)	121.16(19)
C(82) – P(3) – Pd(1)	103.27(16)	C(48) – P(3) – Pd(1)	111.62(16)

5.8 Crystal data for [{PC(sp^3)(PMe$_3$)P}^{tBu}PdI][BAr$^F_4$] (12)

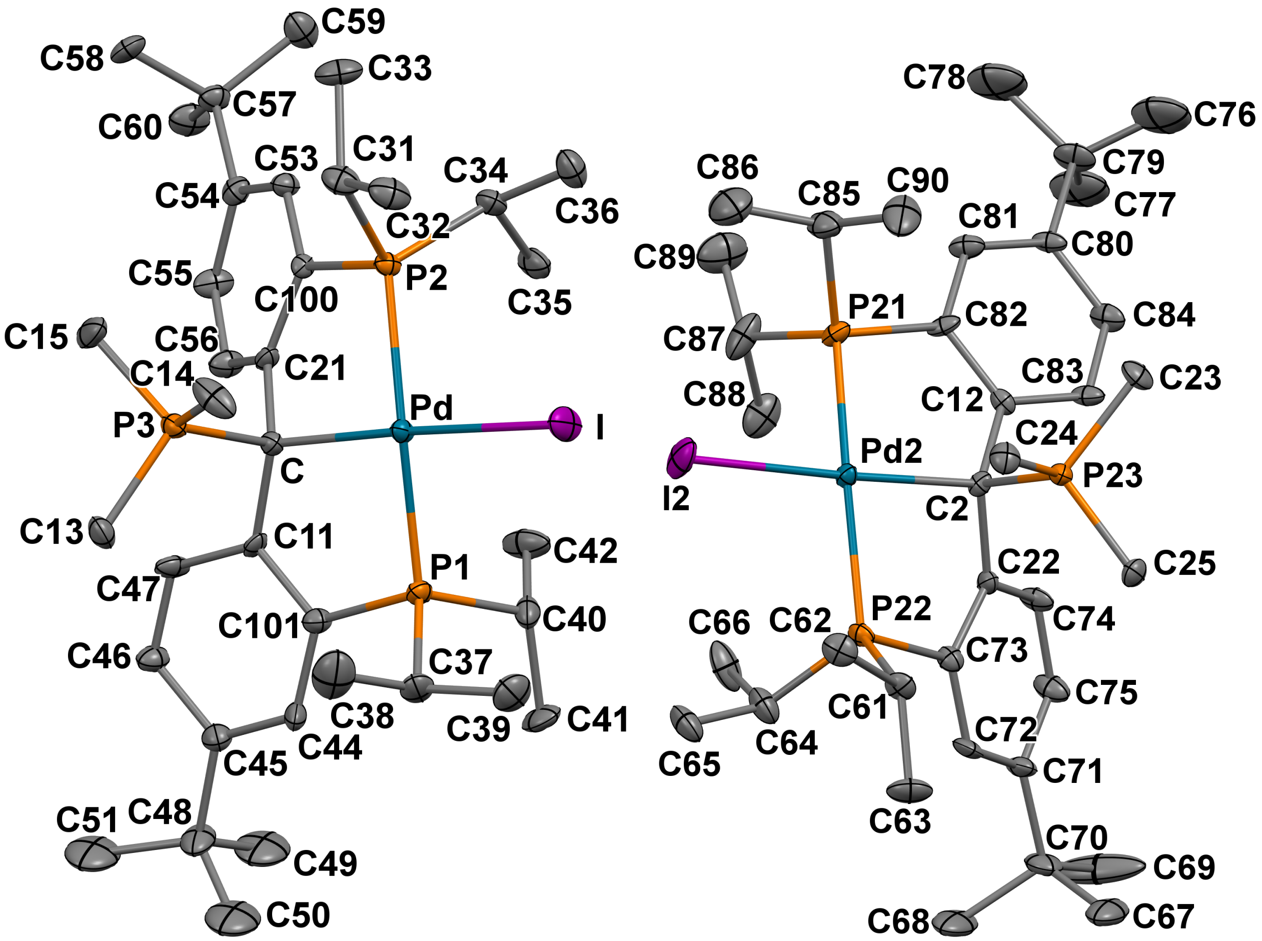

Figure S51. Thermal-ellipsoid representation of the two crystallographically independent molecules of [{PC(sp^3)(PMe$_3$)P}^{tBu}PdI][BAr$^F_4$] (**12**) at 50% probability. Hydrogen atoms and the anions were omitted for clarity.

Table S41. Crystal data and structure refinement for [{PC(sp^3)(PMe$_3$)P}^{tBu}PdI][BAr$^F_4$] (**12**).

Identification code:	pc29	
Empirical formula:	$C_{68}H_{72}BF_{24}IP_3Pd$	
Formula weight:	1682.28	
Temperature:	120(2) K	
Wavelength:	0.71073 Å	
Crystal system:	Monoclinic	
Space group:	$P2_1$	
Unit cell dimensions:	a = 10.0901(7) Å	α = 90°
	b = 23.7955(17) Å	β = 90.532(2)°
	c = 31.205(2) Å	γ = 90°
Volume:	7492.0(9) Å^3	
Z:	4	
Density (calculated):	1.491 g·cm^{-3}	
Absorption coefficient (μ):	0.822 mm^{-1}	
F(000):	3380	
Crystal size:	0.12 × 0.10 × 0.08 mm^3	
θ range for data collection:	0.65 to 25.00°	
Index ranges:	−11 ≤ h ≤ 11, −26 ≤ k ≤ 28, −37 ≤ l ≤ 35	
Reflections collected:	134462	
Independent reflections:	25436 [R_{int} = 0.0348]	
Completeness to θ = 25.00°:	100.0 %	
Absorption correction:	Semi-empirical from equivalents	
Max. and min. transmission:	0.9106 and 0.8668	
Refinement method:	Full-matrix least-squares on F^2	
Data / restraints / parameters:	25436 / 1 / 1563	
Goodness-of-fit on F^2:	1.019	
Final R indices [I>2σ(I)]:	R_1 = 0.0632, wR_2 = 0.1651	
R indices (all data):	R_1 = 0.0658, wR_2 = 0.1675	
Absolute structure parameter:	0.347(17)	
Largest diff. peak and hole:	2.325 and −1.408 e$^-$·Å^{-3}	

Table S42. Atomic coordinates and equivalent isotropic displacement parameters ($Å^2$) for [{PC(sp^3)(PMe$_3$)P}^{tBu}PdI][BAr$^F_4$] (**12**). U(eq) is defined as one third of the trace of the orthogonalized U_{ij} tensor

atom	x	y	z	U(eq)
Pd	0.50136(5)	0.54672(2)	0.77405(2)	0.017(1)
F(1)	−0.0776(11)	0.2811(4)	0.2619(2)	0.102(3)
B(1)	0.0748(8)	0.3330(3)	0.4526(3)	0.018(2)
C	0.2946(7)	0.5378(3)	0.7637(2)	0.018(2)
I	0.75789(5)	0.55439(3)	0.78935(2)	0.041(1)
F(9)	0.3761(5)	0.5606(2)	0.47363(16)	0.038(1)
F(8)	0.4161(6)	0.5079(2)	0.42037(16)	0.043(1)
F(7)	0.2233(5)	0.5409(2)	0.4276(2)	0.052(2)
F(6)	0.4509(9)	0.3632(3)	0.3399(2)	0.077(2)
F(5)	0.3276(9)	0.4218(3)	0.3065(3)	0.090(3)
F(4)	0.3733(9)	0.3479(3)	0.2786(2)	0.084(3)
F(3)	−0.2154(11)	0.3256(4)	0.3026(3)	0.110(4)
P(1)	0.48522(18)	0.46738(8)	0.81371(6)	0.021(1)
F(11)	0.4356(9)	0.3544(3)	0.5823(2)	0.077(2)
C(11)	0.2366(7)	0.4837(3)	0.7826(2)	0.019(2)
P(2)	0.49601(17)	0.60997(8)	0.71715(5)	0.018(1)
F(12)	0.2630(8)	0.3869(4)	0.60801(18)	0.082(3)
C(21)	0.2649(6)	0.5481(4)	0.7161(2)	0.020(1)
P(3)	0.22002(19)	0.59646(8)	0.79439(6)	0.021(1)
F(13)	0.4280(8)	0.4422(2)	0.59633(19)	0.065(2)
C(13)	0.1231(8)	0.5735(4)	0.8390(3)	0.030(2)
F(14)	0.0294(6)	0.1216(3)	0.57180(17)	0.052(1)
C(14)	0.3357(9)	0.6430(4)	0.8196(3)	0.041(2)
C(15)	0.1118(10)	0.6374(4)	0.7614(3)	0.042(2)
F(15)	−0.0096(6)	0.2052(3)	0.59252(18)	0.052(1)
C(200)	0.0386(7)	0.2480(3)	0.5066(2)	0.019(2)
F(16)	−0.1381(6)	0.1662(3)	0.54849(18)	0.052(1)
C(16)	0.832(2)	0.3769(8)	0.1912(5)	0.105(1)
C(017)	0.2063(6)	0.2371(3)	0.4543(2)	0.013(1)
F(17)	0.4248(5)	0.1282(2)	0.47884(15)	0.039(1)
C(17)	0.961(2)	0.3891(8)	0.1282(5)	0.105(1)
C(018)	−0.3405(8)	0.3879(3)	0.4783(3)	0.031(2)
F(18)	0.2865(5)	0.10304(18)	0.43066(15)	0.033(1)
C(18)	0.757(2)	0.3442(8)	0.1214(5)	0.105(1)
F(19)	0.4076(4)	0.17413(19)	0.41981(14)	0.027(1)
C(201)	0.1657(7)	0.1619(3)	0.5044(2)	0.017(1)
C(202)	−0.1860(8)	0.3161(3)	0.4585(2)	0.023(2)
C(203)	−0.0774(7)	0.3521(3)	0.4634(3)	0.022(1)
P(21)	0.52338(19)	0.61029(9)	0.20625(6)	0.022(1)
F(21)	−0.4033(8)	0.2438(3)	0.4749(3)	0.098(2)
F(20)	−0.4237(8)	0.2752(3)	0.4156(4)	0.098(2)
G(21	0.2672(6)	0.6395(3)	0.2217(2)	0.016(1)
P(22)	0.50335(18)	0.69345(8)	0.33694(6)	0.019(1)
F(22)	−0.5376(8)	0.3137(3)	0.4572(4)	0.098(2)
C(22)	0.2827(7)	0.7085(3)	0.2869(2)	0.019(2)
P(23)	0.24672(17)	0.59392(8)	0.30059(6)	0.018(1)
C(23)	0.1458(8)	0.5454(4)	0.2700(3)	0.029(2)
C(24)	0.3670(7)	0.5498(4)	0.3254(3)	0.028(2)

Continued on next page

Table S42. – continued from previous page

atom	x	y	x	U(eq)
F(24)	−0.1708(10)	0.5170(3)	0.4935(4)	0.104(2)
C(25)	0.1428(7)	0.6209(4)	0.3409(2)	0.024(2)
F(25)	−0.3654(10)	0.5056(3)	0.4810(4)	0.104(2)
C(27)	0.3375(7)	0.4596(3)	0.5150(2)	0.022(2)
F(27)	1.1441(6)	0.1708(2)	0.0198(3)	0.067(2)
C(26)	0.2065(7)	0.4292(3)	0.4554(2)	0.021(2)
F(26)	−0.3219(10)	0.4808(3)	0.5375(4)	0.104(2)
C(204)	−0.3123(8)	0.3325(3)	0.4652(3)	0.027(2)
F(28)	1.3481(7)	0.1863(3)	0.0227(4)	0.100(3)
C(28)	0.2281(8)	0.3694(3)	0.5165(2)	0.023(2)
Pd(2)	0.52536(5)	0.63981(2)	0.27521(2)	0.016(1)
F(2)	−0.1595(9)	0.2409(3)	0.3169(2)	0.087(3)
B(2)	0.8942(8)	0.3584(4)	0.0495(3)	0.019(2)
C(2)	0.3172(6)	0.6507(3)	0.2671(2)	0.016(1)
I(2)	0.78300(5)	0.62284(3)	0.28297(2)	0.044(1)
F(29)	1.2309(10)	0.2022(3)	−0.0368(3)	0.087(3)
C(205)	−0.2376(7)	0.4234(3)	0.4851(3)	0.022(1)
C(206)	−0.1058(8)	0.4064(3)	0.4772(3)	0.022(1)
F(30)	0.7813(7)	0.1472(3)	0.0741(3)	0.072(2)
C(31)	0.4976(9)	0.6873(4)	0.7244(3)	0.032(2)
F(31)	0.5848(8)	0.1695(3)	0.0844(2)	0.071(2)
C(32)	0.6050(9)	0.7067(4)	0.7539(3)	0.036(2)
F(32)	0.6340(8)	0.1230(3)	0.0288(2)	0.069(2)
C(33)	0.4955(10)	0.7214(4)	0.6823(3)	0.041(2)
F(33)	0.5730(6)	0.5219(2)	0.0738(2)	0.053(2)
C(34)	0.6078(8)	0.5957(3)	0.6726(2)	0.027(2)
F(34)	0.6506(9)	0.5912(3)	0.0404(3)	0.089(3)
C(207)	0.2134(9)	0.3460(3)	0.3802(2)	0.027(1)
F(36)	0.9516(10)	0.5555(5)	−0.0813(3)	0.105(1)
C(36)	0.7487(8)	0.6191(4)	0.6771(3)	0.037(2)
C(35)	0.6111(9)	0.5329(4)	0.6622(3)	0.035(2)
F(35)	0.5142(7)	0.5330(4)	0.0099(2)	0.080(2)
C(37)	0.5154(8)	0.4619(4)	0.8717(2)	0.033(2)
F(37)	1.1182(11)	0.5200(5)	−0.0513(3)	0.105(1)
C(38)	0.4463(12)	0.5074(6)	0.8950(3)	0.059(3)
F(38)	0.9979(9)	0.4762(4)	−0.0947(2)	0.088(3)
C(39)	0.6639(9)	0.4630(5)	0.8836(3)	0.043(2)
F(39)	1.5006(9)	0.3742(5)	0.0627(5)	0.154(3)
C(208)	0.867(2)	0.3634(8)	0.1018(5)	0.105(1)
C(209)	−0.4205(16)	0.2935(7)	0.4584(7)	0.098(2)
C(210)	0.738(2)	0.3500(8)	0.1652(5)	0.105(1)
C(211)	1.0461(9)	0.3366(3)	0.0413(3)	0.030(2)
C(40)	0.5805(8)	0.4115(3)	0.7875(3)	0.030(2)
F(40)	1.3669(9)	0.4345(5)	0.0766(5)	0.154(3)
C(212)	1.2099(9)	0.2647(4)	0.0228(4)	0.045(2)
C(42)	0.5471(12)	0.4099(4)	0.7410(3)	0.050(3)
C(41)	0.5634(10)	0.3520(4)	0.8074(3)	0.039(2)
F(41)	1.4281(10)	0.4167(5)	0.0159(6)	0.154(3)
C(44)	0.2564(8)	0.4017(3)	0.8283(3)	0.030(2)
F(44)	1.1573(12)	0.3929(5)	0.2003(3)	0.105(1)
F(43)	1.0954(11)	0.4664(4)	0.1839(3)	0.105(1)

Continued on next page

Table S42. – continued from previous page

atom	x	y	x	U(eq)
C(45)	0.1263(8)	0.3882(3)	0.8243(3)	0.027(2)
F(45)	1.0249(11)	0.4270(4)	0.2382(3)	0.105(1)
C(46)	0.0466(8)	0.4254(3)	0.7991(2)	0.027(2)
F(46)	0.5749(11)	0.2767(4)	0.1677(3)	0.105(1)
C(213)	−0.0126(9)	0.3114(3)	0.3738(2)	0.027(1)
C(47)	0.1018(8)	0.4711(4)	0.7795(2)	0.027(2)
F(47)	0.5149(12)	0.3454(4)	0.1693(3)	0.105(1)
C(48)	0.0682(9)	0.3361(4)	0.8455(4)	0.046(3)
C(49)	0.0997(14)	0.2861(5)	0.8136(5)	0.074(2)
C(214)	1.2851(10)	0.3543(5)	0.0424(5)	0.060(4)
C(50)	0.1361(14)	0.3224(5)	0.8884(5)	0.074(2)
C(51)	−0.0818(13)	0.3399(5)	0.8492(5)	0.074(2)
C(53)	0.2997(7)	0.6063(3)	0.6531(2)	0.021(2)
C(54)	0.1922(7)	0.5816(3)	0.6317(2)	0.022(2)
C(56)	0.1668(8)	0.5209(4)	0.6928(2)	0.027(2)
C(55)	0.1335(8)	0.5361(3)	0.6517(2)	0.028(2)
C(57)	0.1495(7)	0.6036(3)	0.5877(2)	0.025(2)
C(58)	0.0731(8)	0.6579(3)	0.5949(3)	0.030(2)
C(59)	0.2693(8)	0.6152(5)	0.5597(2)	0.036(2)
C(215)	0.1215(13)	0.3252(4)	0.3104(3)	0.051(3)
C(60)	0.0619(9)	0.5619(4)	0.5638(3)	0.036(2)
C(61)	0.4898(8)	0.6631(4)	0.3914(2)	0.025(2)
C(62)	0.6069(8)	0.6228(3)	0.4005(2)	0.029(2)
C(63)	0.4713(10)	0.7037(4)	0.4280(3)	0.039(2)
F(63)	0.4648(10)	0.2659(5)	−0.0743(3)	0.111(2)
C(64)	0.6145(8)	0.7544(4)	0.3406(3)	0.036(2)
F(64)	0.6528(10)	0.2708(5)	−0.1024(3)	0.111(2)
C(65)	0.7458(9)	0.7445(4)	0.3627(3)	0.040(2)
F(65)	0.5581(10)	0.3404(5)	−0.0777(3)	0.111(2)
C(66)	0.6345(10)	0.7807(4)	0.2968(3)	0.047(3)
C(67)	0.0878(12)	0.8368(4)	0.3987(4)	0.057(3)
C(68)	0.2656(13)	0.8945(5)	0.3684(4)	0.064(4)
C(69)	0.038(2)	0.8879(7)	0.3328(5)	0.123(9)
C(70)	0.1482(9)	0.8578(4)	0.3544(3)	0.038(2)
C(71)	0.1985(8)	0.8067(3)	0.3310(2)	0.026(2)
C(72)	0.3033(8)	0.7746(3)	0.3451(3)	0.027(2)
C(73)	0.3456(7)	0.7274(3)	0.3236(2)	0.021(2)
C(74)	0.1825(8)	0.7427(3)	0.2704(2)	0.025(2)
C(75)	0.1423(8)	0.7913(3)	0.2905(3)	0.031(2)
C(76)	0.0206(15)	0.5515(7)	0.0967(4)	0.080(2)
C(77)	0.0729(15)	0.6509(7)	0.0723(4)	0.080(2)
C(78)	0.2342(15)	0.5747(7)	0.0642(4)	0.080(2)
C(79)	0.1211(9)	0.5972(4)	0.0930(2)	0.036(2)
C(80)	0.1731(8)	0.6109(3)	0.1382(2)	0.023(2)
C(81)	0.3045(8)	0.6046(3)	0.1493(2)	0.027(2)
C(82)	0.3515(7)	0.6184(3)	0.1907(2)	0.022(2)
C(83)	0.1331(7)	0.6447(3)	0.2105(2)	0.024(2)
C(84)	0.0908(8)	0.6314(3)	0.1695(2)	0.028(2)
C(85)	0.5709(9)	0.5399(4)	0.1891(3)	0.038(2)
C(86)	0.7175(12)	0.5291(5)	0.1892(4)	0.062(3)
C(87)	0.6152(10)	0.6613(4)	0.1733(3)	0.044(2)

Continued on next page

Table S42. – continued from previous page

atom	x	y	x	U(eq)
C(88)	0.5588(10)	0.7188(4)	0.1804(3)	0.044(2)
C(89)	0.6301(13)	0.6512(5)	0.1277(3)	0.061(3)
C(90)	0.4949(11)	0.4959(4)	0.2135(5)	0.059(3)
C(91)	0.8134(9)	0.5233(4)	−0.0126(3)	0.031(2)
C(92)	0.9381(7)	0.4398(3)	−0.0073(2)	0.022(2)
C(93)	0.7649(7)	0.4547(3)	0.0403(2)	0.019(2)
C(94)	1.0788(8)	0.2824(4)	0.0287(3)	0.035(2)
C(95)	1.3162(10)	0.3024(5)	0.0304(5)	0.058(3)
C(96)	1.1528(8)	0.3730(4)	0.0467(4)	0.046(3)
C(97)	0.7741(7)	0.2587(3)	0.0473(2)	0.019(2)
C(98)	0.6291(8)	0.2265(4)	−0.0087(3)	0.031(2)
C(99)	0.7276(8)	0.3194(3)	−0.0095(2)	0.026(2)
C(100)	0.3380(6)	0.5894(3)	0.6936(2)	0.015(1)
C(101)	0.3140(7)	0.4482(3)	0.8073(2)	0.022(2)
C(102)	0.1761(6)	0.3785(3)	0.4751(2)	0.015(1)
C(103)	0.2875(7)	0.4689(3)	0.4750(2)	0.020(2)
C(104)	0.3218(8)	0.5200(3)	0.4503(3)	0.029(2)
C(105)	0.3054(8)	0.4118(3)	0.5361(2)	0.024(2)
C(106)	0.3596(11)	0.3991(4)	0.5797(3)	0.045(3)
C(107)	0.0073(12)	0.3083(4)	0.3290(3)	0.046(3)
C(108)	−0.1090(18)	0.2893(7)	0.3035(4)	0.082(5)
C(109)	0.0953(9)	0.3296(3)	0.4004(2)	0.027(1)
C(110)	0.2254(11)	0.3443(4)	0.3360(3)	0.041(2)
C(111)	0.3432(14)	0.3678(5)	0.3161(4)	0.056(3)
C(112)	0.2347(6)	0.1840(3)	0.4699(2)	0.012(1)
C(113)	0.3388(6)	0.1486(3)	0.4500(2)	0.019(1)
C(114)	−0.0091(11)	0.1716(5)	0.5579(3)	0.052(1)
C(115)	0.0676(7)	0.1946(3)	0.5224(2)	0.018(2)
C(117)	0.1071(6)	0.2700(3)	0.4720(2)	0.014(1)
C(118)	0.7378(8)	0.5051(3)	0.0208(2)	0.023(2)
C(120)	0.8691(7)	0.4193(3)	0.0276(2)	0.019(2)
C(121)	0.9148(8)	0.4906(4)	−0.0267(2)	0.027(2)
C(122)	0.997(2)	0.5093(9)	−0.0627(5)	0.105(1)
C(123)	0.6760(10)	0.1628(4)	0.0527(3)	0.041(2)
C(124)	0.6948(8)	0.2181(3)	0.0293(2)	0.024(2)
C(125)	0.6462(9)	0.2781(4)	−0.0279(3)	0.033(2)
C(126)	0.581(2)	0.2870(10)	−0.0684(5)	0.111(2)
C(127)	0.7930(7)	0.3116(3)	0.0282(2)	0.016(1)
C(128)	0.6207(11)	0.5385(4)	0.0360(3)	0.044(3)
C(129)	−0.2574(19)	0.4806(6)	0.5011(7)	0.104(2)
C(150)	0.622(2)	0.3248(9)	0.1867(5)	0.105(1)
C(151)	1.035(2)	0.4275(9)	0.1984(5)	0.105(1)
C(152)	0.940(2)	0.3960(8)	0.1711(5)	0.105(1)
C(153)	1.3902(19)	0.3923(10)	0.0488(11)	0.154(3)
C(154)	1.2389(9)	0.2052(4)	0.0065(4)	0.045(2)
F(250)	0.5997(11)	0.3464(5)	0.2233(3)	0.105(1)
H(13A)	0.1113	0.6048	0.8590	0.045
H(13B)	0.1688	0.5425	0.8536	0.045
H(13C)	0.0362	0.5606	0.8287	0.045
H(14A)	0.4030	0.6543	0.7988	0.061
H(14B)	0.3786	0.6238	0.8438	0.061

Continued on next page

Table S42. – continued from previous page

atom	x	y	x	U(eq)
H(14C)	0.2890	0.6764	0.8299	0.061
H(15A)	0.1625	0.6544	0.7381	0.063
H(15B)	0.0712	0.6670	0.7787	0.063
H(15C)	0.0424	0.6132	0.7493	0.063
H(200)	−0.0291	0.2696	0.5196	0.022
H(16)	0.8207	0.3814	0.2212	0.125
H(017)	0.2557	0.2514	0.4309	0.015
H(17)	1.0412	0.4022	0.1161	0.125
H(018)	−0.4293	0.4000	0.4822	0.037
H(18)	0.6900	0.3262	0.1046	0.125
H(201)	0.1853	0.1255	0.5153	0.021
H(202)	−0.1703	0.2783	0.4500	0.027
H(23A)	0.0571	0.5613	0.2657	0.044
H(23B)	0.1389	0.5097	0.2855	0.044
H(23C)	0.1866	0.5387	0.2421	0.044
H(24A)	0.3227	0.5233	0.3446	0.042
H(24B)	0.4295	0.5728	0.3420	0.042
H(24C)	0.4151	0.5290	0.3034	0.042
H(25A)	0.0812	0.6482	0.3283	0.037
H(25B)	0.1970	0.6393	0.3630	0.037
H(25C)	0.0926	0.5900	0.3538	0.037
H(27)	0.3945	0.4865	0.5281	0.027
H(26)	0.1708	0.4369	0.4278	0.025
H(28)	0.2115	0.3352	0.5310	0.028
H(206)	−0.0355	0.4325	0.4813	0.027
H(31)	0.4128	0.6966	0.7392	0.038
H(32A)	0.6912	0.7001	0.7406	0.054
H(32B)	0.6003	0.6858	0.7809	0.054
H(32C)	0.5942	0.7469	0.7595	0.054
H(33A)	0.4202	0.7094	0.6645	0.061
H(33B)	0.5782	0.7151	0.6668	0.061
H(33C)	0.4867	0.7615	0.6890	0.061
H(34)	0.5678	0.6146	0.6469	0.032
H(207)	0.2863	0.3585	0.3972	0.033
H(36A)	0.7929	0.6175	0.6493	0.056
H(36B)	0.7985	0.5965	0.6981	0.056
H(36C)	0.7447	0.6582	0.6869	0.056
H(35A)	0.5209	0.5177	0.6630	0.052
H(35B)	0.6666	0.5134	0.6834	0.052
H(35C)	0.6480	0.5274	0.6336	0.052
H(37)	0.4782	0.4252	0.8816	0.039
H(38A)	0.4933	0.5430	0.8906	0.089
H(38B)	0.4446	0.4986	0.9256	0.089
H(38C)	0.3553	0.5108	0.8840	0.089
H(39A)	0.7110	0.4349	0.8665	0.064
H(39B)	0.6752	0.4543	0.9141	0.064
H(39C)	0.7001	0.5004	0.8777	0.064
H(40)	0.6764	0.4216	0.7902	0.037
H(42A)	0.5201	0.3757	0.7280	0.060
H(42B)	0.5528	0.4432	0.7243	0.060
H(41A)	0.4748	0.3376	0.8005	0.058

Continued on next page

Table S42. – continued from previous page

atom	x	y	x	U(eq)
H(41B)	0.5742	0.3543	0.8386	0.058
H(41C)	0.6304	0.3266	0.7958	0.058
H(44)	0.3114	0.3790	0.8461	0.037
H(46)	−0.0456	0.4183	0.7958	0.032
H(213)	−0.0955	0.3016	0.3859	0.033
H(47)	0.0460	0.4953	0.7632	0.033
H(49A)	0.0309	0.2572	0.8159	0.111
H(49B)	0.1861	0.2697	0.8210	0.111
H(49C)	0.1017	0.3004	0.7842	0.111
H(50A)	0.2282	0.3114	0.8834	0.111
H(50B)	0.0890	0.2915	0.9023	0.111
H(50C)	0.1343	0.3557	0.9069	0.111
H(51A)	−0.1071	0.3787	0.8559	0.111
H(51B)	−0.1118	0.3149	0.8721	0.111
H(51C)	−0.1231	0.3286	0.8220	0.111
H(53)	0.3476	0.6355	0.6393	0.026
H(56)	0.1209	0.4905	0.7058	0.033
H(55)	0.0686	0.5149	0.6365	0.034
H(58A)	0.0381	0.6717	0.5674	0.045
H(58B)	0.1324	0.6862	0.6074	0.045
H(58C)	−0.0005	0.6509	0.6144	0.045
H(59A)	0.2394	0.6236	0.5304	0.055
H(59B)	0.3271	0.5821	0.5595	0.055
H(59C)	0.3184	0.6475	0.5712	0.055
H(215)	0.1303	0.3239	0.2802	0.061
H(60A)	0.0463	0.5753	0.5345	0.054
H(60B)	−0.0230	0.5583	0.5785	0.054
H(60C)	0.1059	0.5252	0.5629	0.054
H(61)	0.4085	0.6391	0.3908	0.030
H(62A)	0.6217	0.5989	0.3755	0.044
H(62B)	0.5863	0.5993	0.4253	0.044
H(62C)	0.6871	0.6448	0.4066	0.044
H(63A)	0.5527	0.7255	0.4323	0.058
H(63B)	0.4513	0.6827	0.4542	0.058
H(63C)	0.3977	0.7293	0.4213	0.058
H(64)	0.5671	0.7832	0.3581	0.043
H(65A)	0.7947	0.7152	0.3475	0.060
H(65B)	0.7307	0.7325	0.3923	0.060
H(65C)	0.7975	0.7794	0.3627	0.060
H(66A)	0.6946	0.7572	0.2800	0.071
H(66B)	0.6729	0.8183	0.3002	0.071
H(66C)	0.5490	0.7836	0.2818	0.071
H(67A)	0.0091	0.8137	0.3930	0.085
H(67B)	0.0630	0.8694	0.4161	0.085
H(67C)	0.1541	0.8144	0.4142	0.085
H(68A)	0.3069	0.9111	0.3431	0.096
H(68B)	0.3307	0.8713	0.3838	0.096
H(68C)	0.2343	0.9245	0.3873	0.096
H(69A)	0.0743	0.9148	0.3122	0.185
H(69B)	−0.0139	0.9082	0.3542	0.185
H(69C)	−0.0187	0.8608	0.3178	0.185

Continued on next page

Table S42. – continued from previous page

atom	x	y	x	U(eq)
H(72)	0.3482	0.7853	0.3708	0.033
H(74)	0.1402	0.7321	0.2442	0.030
H(75)	0.0772	0.8147	0.2774	0.037
H(76A)	−0.0643	0.5676	0.1056	0.120
H(76B)	0.0506	0.5240	0.1181	0.120
H(76C)	0.0093	0.5329	0.0689	0.120
H(77A)	0.0546	0.6442	0.0418	0.120
H(77B)	0.1412	0.6800	0.0753	0.120
H(77C)	−0.0083	0.6635	0.0864	0.120
H(78A)	0.2013	0.5706	0.0347	0.120
H(78B)	0.2640	0.5381	0.0750	0.120
H(78C)	0.3085	0.6012	0.0648	0.120
H(81)	0.3648	0.5906	0.1286	0.033
H(83)	0.0712	0.6573	0.2311	0.029
H(84)	−0.0002	0.6367	0.1626	0.033
H(85)	0.5412	0.5365	0.1585	0.045
H(86A)	0.7376	0.4987	0.1691	0.093
H(86B)	0.7465	0.5183	0.2181	0.093
H(86C)	0.7641	0.5634	0.1805	0.093
H(87)	0.7072	0.6623	0.1855	0.053
H(88A)	0.5387	0.7423	0.1566	0.053
H(88B)	0.5432	0.7317	0.2087	0.053
H(89A)	0.5434	0.6541	0.1135	0.091
H(89B)	0.6664	0.6134	0.1233	0.091
H(89C)	0.6905	0.6791	0.1157	0.091
H(90A)	0.5254	0.4954	0.2434	0.071
H(90B)	0.5099	0.4589	0.2006	0.071
H(90C)	0.4001	0.5048	0.2124	0.071
H(91)	0.7953	0.5584	−0.0258	0.037
H(92)	1.0065	0.4170	−0.0187	0.026
H(93)	0.7108	0.4432	0.0634	0.023
H(94)	1.0093	0.2562	0.0238	0.042
H(95)	1.4058	0.2912	0.0270	0.070
H(96)	1.1364	0.4113	0.0535	0.056
H(97)	0.8178	0.2508	0.0737	0.023
H(98)	0.5746	0.1982	−0.0212	0.037
H(99)	0.7375	0.3542	−0.0240	0.031

Table S43. Anisotropic displacement parameters ($Å^2$) for [{PC(sp^3)(PMe$_3$)P}^{tBu}PdI][BAr$^F_4$] (**12**). The anisotropic displacement factor exponent takes the form: $-2\pi^2[h^2a^{*2}U_{11} + ... + 2hka^*b^*U_{12}]$.

atom	U11	U22	U33	U23	U13	U12
Pd	0.0131(2)	0.0211(3)	0.0164(2)	0.0030(2)	−0.0022(2)	−0.0029(2)
F(1)	0.196(10)	0.087(6)	0.022(3)	−0.006(3)	−0.022(4)	−0.032(6)
B(1)	0.021(4)	0.010(4)	0.023(4)	−0.002(3)	0.004(3)	0.004(3)
C	0.019(3)	0.018(4)	0.017(3)	0.002(3)	0.003(3)	0.002(3)
I	0.0160(2)	0.0592(4)	0.0480(3)	0.0197(3)	−0.0090(2)	−0.0083(2)
F(9)	0.060(3)	0.018(3)	0.037(3)	−0.003(2)	0.001(2)	−0.021(2)
F(8)	0.060(3)	0.037(3)	0.031(3)	0.002(2)	0.016(2)	−0.011(3)
F(7)	0.048(3)	0.034(3)	0.073(4)	0.031(3)	−0.022(3)	−0.011(3)
F(6)	0.092(6)	0.070(5)	0.070(5)	0.000(4)	0.049(4)	−0.012(4)
F(5)	0.112(6)	0.033(4)	0.127(7)	0.025(4)	0.075(5)	0.006(4)
F(4)	0.119(6)	0.090(5)	0.044(4)	0.006(4)	0.053(4)	0.037(5)
F(3)	0.128(8)	0.087(6)	0.114(7)	−0.043(5)	−0.078(6)	0.017(6)
P(1)	0.0177(9)	0.0252(10)	0.0201(9)	0.0070(8)	−0.0029(7)	0.0003(7)
F(11)	0.127(7)	0.035(4)	0.068(4)	0.014(3)	−0.061(4)	0.013(4)
C(11)	0.018(3)	0.022(4)	0.016(3)	0.004(3)	−0.006(3)	0.007(3)
P(2)	0.0165(8)	0.0192(10)	0.0175(8)	0.0022(7)	−0.0025(6)	−0.0046(7)
F(12)	0.100(6)	0.123(7)	0.023(3)	0.012(3)	−0.011(3)	−0.045(5)
C(21)	0.019(3)	0.024(4)	0.016(3)	0.005(3)	−0.001(2)	0.007(3)
P(3)	0.0242(9)	0.0190(10)	0.0209(9)	0.0013(7)	0.0065(7)	0.0005(7)
F(13)	0.112(6)	0.032(3)	0.049(3)	0.001(3)	−0.048(4)	−0.022(3)
C(13)	0.021(4)	0.043(5)	0.026(4)	0.002(3)	0.007(3)	0.000(3)
F(14)	0.0538(18)	0.062(2)	0.0403(16)	0.0206(15)	0.0208(14)	−0.0055(16)
C(14)	0.052(5)	0.033(5)	0.038(5)	−0.019(4)	0.021(4)	−0.022(4)
C(15)	0.050(5)	0.041(6)	0.035(5)	0.012(4)	0.014(4)	0.029(5)
F(15)	0.0538(18)	0.062(2)	0.0403(16)	0.0206(15)	0.0208(14)	−0.0055(16)
C(200)	0.014(3)	0.022(4)	0.020(3)	0.002(3)	0.005(3)	0.005(3)
F(16)	0.0538(18)	0.062(2)	0.0403(16)	0.0206(15)	0.0208(14)	−0.0055(16)
C(16)	0.140(3)	0.113(2)	0.0607(15)	0.0074(15)	0.0041(16)	−0.041(2)
C(017)	0.015(3)	0.011(3)	0.013(3)	0.007(2)	0.001(2)	−0.004(3)
F(17)	0.034(3)	0.052(4)	0.031(2)	0.002(2)	0.0011(19)	0.028(2)
C(17)	0.140(3)	0.113(2)	0.0607(15)	0.0074(15)	0.0041(16)	−0.041(2)
C(018)	0.019(4)	0.020(4)	0.053(5)	−0.009(4)	−0.009(3)	0.006(3)
F(18)	0.044(3)	0.011(2)	0.044(3)	−0.0129(19)	0.013(2)	−0.0059(19)
C(18)	0.140(3)	0.113(2)	0.0607(15)	0.0074(15)	0.0041(16)	−0.041(2)
F(19)	0.027(2)	0.027(3)	0.027(2)	−0.0057(19)	0.0140(18)	−0.0020(19)
C(201)	0.022(3)	0.013(4)	0.016(3)	0.003(3)	0.000(3)	−0.002(3)
C(202)	0.031(4)	0.010(4)	0.027(4)	−0.004(3)	−0.014(3)	0.007(3)
C(203)	0.020(2)	0.014(2)	0.033(2)	0.0005(17)	−0.0027(17)	−0.0012(17)
P(21)	0.0230(9)	0.0284(11)	0.0156(8)	−0.0021(7)	0.0021(7)	0.0084(8)
F(21)	0.070(3)	0.061(3)	0.164(5)	0.006(3)	−0.038(3)	−0.038(2)
F(20)	0.070(3)	0.061(3)	0.164(5)	0.006(3)	−0.038(3)	−0.038(2)
G(21	0.018(3)	0.014(3)	0.017(3)	0.004(3)	−0.006(2)	−0.002(3)
P(22)	0.0190(9)	0.0205(10)	0.0182(9)	−0.0021(7)	−0.0065(7)	0.0003(7)
F(22)	0.070(3)	0.061(3)	0.164(5)	0.006(3)	−0.038(3)	−0.038(2)
C(22)	0.013(3)	0.025(4)	0.018(3)	−0.004(3)	−0.002(3)	0.001(3)
P(23)	0.0170(9)	0.0176(10)	0.0188(9)	−0.0041(7)	0.0001(7)	−0.0014(7)
C(23)	0.034(4)	0.020(4)	0.033(4)	0.000(3)	−0.004(3)	−0.011(4)
C(24)	0.029(4)	0.021(4)	0.034(4)	0.005(3)	0.001(3)	0.001(3)
F(24)	0.106(4)	0.037(3)	0.170(5)	−0.031(3)	0.023(4)	0.017(2)

Continued on next page

Table S43. – continued from previous page

atom	U_{11}	U_{22}	U_{33}	U_{23}	U_{13}	U_{12}
C(25)	0.023(4)	0.029(4)	0.021(3)	0.001(3)	0.007(3)	−0.003(3)
F(25)	0.106(4)	0.037(3)	0.170(5)	−0.031(3)	0.023(4)	0.017(2)
C(27)	0.024(4)	0.012(4)	0.030(4)	0.000(3)	−0.005(3)	0.004(3)
F(27)	0.054(4)	0.019(3)	0.130(6)	−0.016(3)	0.009(4)	−0.005(3)
C(26)	0.029(4)	0.015(4)	0.020(3)	−0.004(3)	0.008(3)	0.010(3)
F(26)	0.106(4)	0.037(3)	0.170(5)	−0.031(3)	0.023(4)	0.017(2)
C(204)	0.026(4)	0.018(4)	0.037(5)	−0.001(3)	−0.007(3)	0.001(3)
F(28)	0.044(4)	0.040(4)	0.213(10)	−0.040(5)	−0.044(5)	0.024(3)
C(28)	0.029(4)	0.015(4)	0.027(4)	0.002(3)	0.000(3)	0.004(3)
Pd(2)	0.0135(2)	0.0206(3)	0.0146(2)	0.0006(2)	−0.0011(2)	0.0025(2)
F(2)	0.125(7)	0.082(6)	0.053(4)	0.016(4)	−0.029(4)	−0.064(5)
B(2)	0.017(4)	0.022(5)	0.018(4)	−0.001(3)	−0.007(3)	−0.005(3)
C(2)	0.015(3)	0.018(4)	0.015(3)	0.003(3)	−0.001(2)	0.006(3)
I(2)	0.0169(2)	0.0802(5)	0.0344(3)	−0.0027(3)	−0.0024(2)	0.0138(3)
F(29)	0.128(7)	0.056(5)	0.078(5)	−0.012(4)	0.031(5)	0.029(5)
C(205)	0.020(2)	0.014(2)	0.033(2)	0.0005(17)	−0.0027(17)	−0.0012(17)
C(206)	0.020(2)	0.014(2)	0.033(2)	0.0005(17)	−0.0027(17)	−0.0012(17)
F(30)	0.059(4)	0.033(4)	0.122(6)	0.032(4)	−0.035(4)	−0.007(3)
C(31)	0.039(5)	0.025(5)	0.031(4)	−0.001(3)	0.003(4)	−0.010(4)
F(31)	0.095(5)	0.064(5)	0.054(4)	0.031(3)	0.011(4)	−0.015(4)
C(32)	0.039(5)	0.039(5)	0.030(4)	0.002(4)	−0.005(4)	−0.017(4)
F(32)	0.124(6)	0.028(3)	0.055(4)	0.005(3)	−0.030(4)	−0.035(4)
C(33)	0.061(6)	0.026(5)	0.035(5)	0.012(4)	−0.007(4)	−0.006(4)
F(33)	0.058(4)	0.034(3)	0.068(4)	−0.001(3)	0.033(3)	0.012(3)
C(34)	0.030(4)	0.030(4)	0.021(4)	0.006(3)	0.006(3)	0.001(3)
F(34)	0.118(6)	0.023(3)	0.127(7)	−0.007(4)	0.083(6)	0.002(3)
C(207)	0.052(3)	0.015(2)	0.015(2)	0.0029(16)	−0.0002(19)	−0.0070(19)
F(36)	0.140(3)	0.113(2)	0.0607(15)	0.0074(15)	0.0041(16)	−0.041(2)
C(36)	0.027(4)	0.054(6)	0.030(4)	−0.003(4)	0.007(3)	−0.002(4)
C(35)	0.045(5)	0.031(5)	0.029(4)	0.001(3)	0.020(4)	0.006(4)
F(35)	0.068(4)	0.108(7)	0.064(4)	−0.006(4)	−0.013(3)	0.038(4)
C(37)	0.031(4)	0.046(5)	0.021(4)	0.010(4)	−0.005(3)	0.005(4)
F(37)	0.140(3)	0.113(2)	0.0607(15)	0.0074(15)	0.0041(16)	−0.041(2)
C(38)	0.059(7)	0.085(9)	0.033(5)	−0.006(5)	−0.008(5)	0.017(6)
F(38)	0.118(6)	0.107(6)	0.040(4)	−0.017(4)	0.052(4)	−0.043(5)
C(39)	0.038(5)	0.054(6)	0.036(5)	0.006(4)	−0.014(4)	0.004(4)
F(39)	0.061(3)	0.101(5)	0.298(11)	−0.050(5)	−0.037(4)	−0.034(3)
C(208)	0.140(3)	0.113(2)	0.0607(15)	0.0074(15)	0.0041(16)	−0.041(2)
C(209)	0.070(3)	0.061(3)	0.164(5)	0.006(3)	−0.038(3)	−0.038(2)
C(210)	0.140(3)	0.113(2)	0.0607(15)	0.0074(15)	0.0041(16)	−0.041(2)
C(211)	0.033(5)	0.016(4)	0.041(5)	0.001(3)	−0.011(4)	0.001(3)
C(40)	0.020(4)	0.022(4)	0.050(5)	0.003(4)	0.008(3)	0.002(3)
F(40)	0.061(3)	0.101(5)	0.298(11)	−0.050(5)	−0.037(4)	−0.034(3)
C(212)	0.031(5)	0.025(5)	0.078(7)	−0.015(5)	−0.003(5)	0.008(4)
C(42)	0.092(8)	0.033(5)	0.026(5)	0.004(4)	0.022(5)	0.014(5)
C(41)	0.056(6)	0.020(4)	0.041(5)	0.011(4)	0.003(4)	0.014(4)
F(41)	0.061(3)	0.101(5)	0.298(11)	−0.050(5)	−0.037(4)	−0.034(3)
C(44)	0.017(4)	0.025(4)	0.050(5)	0.011(4)	0.009(3)	0.007(3)
F(44)	0.140(3)	0.113(2)	0.0607(15)	0.0074(15)	0.0041(16)	−0.041(2)
F(43)	0.140(3)	0.113(2)	0.0607(15)	0.0074(15)	0.0041(16)	−0.041(2)
C(45)	0.027(4)	0.022(4)	0.033(4)	0.004(3)	0.004(3)	0.001(3)

Continued on next page

Table S43. – continued from previous page

atom	U_{11}	U_{22}	U_{33}	U_{23}	U_{13}	U_{12}
F(45)	0.140(3)	0.113(2)	0.0607(15)	0.0074(15)	0.0041(16)	−0.041(2)
C(46)	0.025(4)	0.024(4)	0.031(4)	0.008(3)	−0.005(3)	−0.006(3)
F(46)	0.140(3)	0.113(2)	0.0607(15)	0.0074(15)	0.0041(16)	−0.041(2)
C(213)	0.052(3)	0.015(2)	0.015(2)	0.0029(16)	−0.0002(19)	−0.0070(19)
C(47)	0.026(4)	0.032(5)	0.025(4)	0.015(3)	−0.011(3)	−0.007(3)
F(47)	0.140(3)	0.113(2)	0.0607(15)	0.0074(15)	0.0041(16)	−0.041(2)
C(48)	0.033(5)	0.019(5)	0.085(8)	0.019(5)	0.006(5)	−0.002(4)
C(49)	0.073(5)	0.037(4)	0.113(6)	0.032(4)	0.004(4)	−0.023(3)
C(214)	0.021(5)	0.042(6)	0.118(10)	−0.025(6)	−0.021(5)	0.009(4)
C(50)	0.073(5)	0.037(4)	0.113(6)	0.032(4)	0.004(4)	−0.023(3)
C(51)	0.073(5)	0.037(4)	0.113(6)	0.032(4)	0.004(4)	−0.023(3)
C(53)	0.020(3)	0.023(4)	0.021(3)	0.006(3)	−0.002(3)	−0.004(3)
C(54)	0.026(4)	0.019(4)	0.022(4)	−0.003(3)	−0.001(3)	0.003(3)
C(56)	0.028(4)	0.028(4)	0.026(4)	0.006(3)	−0.009(3)	−0.007(3)
C(55)	0.033(4)	0.024(5)	0.027(4)	0.002(3)	−0.015(3)	−0.013(3)
C(57)	0.026(4)	0.029(5)	0.020(4)	0.005(3)	−0.005(3)	−0.001(3)
C(58)	0.036(4)	0.026(4)	0.028(4)	0.009(3)	−0.009(3)	0.008(3)
C(59)	0.027(4)	0.058(6)	0.024(4)	0.001(4)	−0.003(3)	−0.004(4)
C(215)	0.107(9)	0.032(5)	0.014(4)	−0.009(3)	0.010(5)	−0.014(5)
C(60)	0.039(5)	0.042(6)	0.027(4)	0.005(4)	−0.012(3)	−0.007(4)
C(61)	0.026(4)	0.032(4)	0.016(3)	0.001(3)	−0.008(3)	0.002(3)
C(62)	0.036(4)	0.027(4)	0.025(4)	0.007(3)	−0.008(3)	0.003(3)
C(63)	0.064(6)	0.035(5)	0.018(4)	−0.007(3)	−0.003(4)	0.005(4)
F(63)	0.118(4)	0.156(5)	0.058(3)	0.027(3)	−0.056(3)	−0.017(4)
C(64)	0.027(4)	0.032(5)	0.048(5)	0.001(4)	−0.011(4)	−0.008(4)
F(64)	0.118(4)	0.156(5)	0.058(3)	0.027(3)	−0.056(3)	−0.017(4)
C(65)	0.030(4)	0.032(5)	0.057(6)	−0.003(4)	−0.017(4)	−0.007(4)
F(65)	0.118(4)	0.156(5)	0.058(3)	0.027(3)	−0.056(3)	−0.017(4)
C(66)	0.041(5)	0.041(6)	0.060(6)	0.026(5)	−0.014(5)	−0.022(4)
C(67)	0.072(7)	0.029(6)	0.070(7)	−0.025(5)	0.030(6)	−0.011(5)
C(68)	0.084(8)	0.031(6)	0.078(8)	−0.032(5)	0.032(7)	−0.020(5)
C(69)	0.21(2)	0.090(12)	0.066(9)	−0.045(8)	−0.063(11)	0.111(14)
C(70)	0.044(5)	0.024(5)	0.047(5)	−0.020(4)	−0.003(4)	0.000(4)
C(71)	0.028(4)	0.025(4)	0.025(4)	−0.013(3)	−0.006(3)	0.001(3)
C(72)	0.030(4)	0.024(4)	0.027(4)	−0.014(3)	−0.006(3)	−0.006(3)
C(73)	0.021(4)	0.017(4)	0.024(4)	0.001(3)	−0.003(3)	0.000(3)
C(74)	0.029(4)	0.024(4)	0.022(4)	−0.005(3)	−0.014(3)	0.005(3)
C(75)	0.035(4)	0.021(4)	0.037(4)	−0.004(3)	−0.013(4)	0.004(3)
C(76)	0.102(6)	0.099(6)	0.039(4)	−0.026(4)	−0.021(4)	0.004(5)
C(77)	0.102(6)	0.099(6)	0.039(4)	−0.026(4)	−0.021(4)	0.004(5)
C(78)	0.102(6)	0.099(6)	0.039(4)	−0.026(4)	−0.021(4)	0.004(5)
C(79)	0.050(5)	0.041(5)	0.017(4)	−0.003(3)	−0.014(3)	0.001(4)
C(80)	0.032(4)	0.018(4)	0.018(3)	−0.004(3)	−0.010(3)	0.008(3)
C(81)	0.036(4)	0.026(4)	0.019(4)	−0.004(3)	−0.004(3)	0.007(3)
C(82)	0.028(4)	0.022(4)	0.017(3)	−0.003(3)	0.001(3)	0.014(3)
C(83)	0.031(4)	0.020(4)	0.021(3)	−0.008(3)	−0.006(3)	0.009(3)
C(84)	0.029(4)	0.028(5)	0.026(4)	−0.003(3)	−0.015(3)	0.009(3)
C(85)	0.034(4)	0.036(5)	0.043(5)	−0.016(4)	−0.004(4)	0.011(4)
C(86)	0.057(7)	0.055(7)	0.073(8)	−0.016(6)	0.006(6)	0.013(5)
C(87)	0.036(5)	0.050(6)	0.047(5)	0.022(5)	0.020(4)	0.018(4)
C(88)	0.049(6)	0.046(6)	0.039(5)	0.021(4)	0.013(4)	0.008(5)

Continued on next page

Table S43. – continued from previous page

atom	U_{11}	U_{22}	U_{33}	U_{23}	U_{13}	U_{12}
C(89)	0.089(9)	0.051(7)	0.043(6)	0.008(5)	0.017(6)	0.000(6)
C(90)	0.051(6)	0.020(5)	0.107(10)	−0.009(6)	0.019(6)	0.000(4)
C(91)	0.046(5)	0.022(4)	0.025(4)	0.005(3)	−0.011(4)	−0.011(4)
C(92)	0.018(3)	0.027(4)	0.021(4)	−0.003(3)	0.000(3)	−0.004(3)
C(93)	0.027(4)	0.017(4)	0.014(3)	−0.004(3)	0.001(3)	0.003(3)
C(94)	0.023(4)	0.024(5)	0.057(6)	−0.003(4)	−0.007(4)	−0.001(3)
C(95)	0.028(5)	0.039(7)	0.108(10)	0.001(6)	−0.005(5)	0.009(4)
C(96)	0.013(4)	0.019(5)	0.108(9)	−0.006(5)	−0.005(4)	0.007(3)
C(97)	0.021(3)	0.011(4)	0.027(4)	0.004(3)	−0.007(3)	0.001(3)
C(98)	0.029(4)	0.035(5)	0.029(4)	0.001(4)	−0.007(3)	−0.011(4)
C(99)	0.035(4)	0.021(4)	0.021(4)	0.005(3)	−0.003(3)	−0.008(3)
C(100)	0.013(3)	0.013(4)	0.018(3)	−0.001(3)	−0.003(3)	−0.003(3)
C(101)	0.025(4)	0.020(4)	0.022(4)	0.004(3)	−0.001(3)	−0.002(3)
C(102)	0.006(3)	0.020(4)	0.020(3)	0.002(3)	0.006(2)	0.002(3)
C(103)	0.014(3)	0.015(4)	0.032(4)	−0.006(3)	−0.001(3)	0.004(3)
C(104)	0.035(4)	0.017(4)	0.035(4)	−0.003(3)	0.002(3)	−0.007(3)
C(105)	0.024(4)	0.022(4)	0.026(4)	0.003(3)	−0.006(3)	−0.002(3)
C(106)	0.074(7)	0.024(5)	0.034(5)	0.004(4)	−0.032(5)	−0.019(5)
C(107)	0.080(7)	0.041(6)	0.016(4)	0.002(4)	−0.007(4)	−0.015(5)
C(108)	0.125(13)	0.088(11)	0.032(6)	−0.022(6)	−0.024(7)	−0.030(10)
C(109)	0.052(3)	0.015(2)	0.015(2)	0.0029(16)	−0.0002(19)	−0.0070(19)
C(110)	0.080(7)	0.023(5)	0.019(4)	0.001(3)	0.021(4)	−0.005(4)
C(111)	0.081(9)	0.051(7)	0.037(6)	−0.006(5)	0.019(6)	0.007(6)
C(112)	0.012(3)	0.013(3)	0.012(3)	−0.002(2)	0.002(2)	0.000(2)
C(113)	0.015(3)	0.019(4)	0.022(3)	−0.001(3)	−0.001(3)	0.004(3)
C(114)	0.0538(18)	0.062(2)	0.0403(16)	0.0206(15)	0.0208(14)	−0.0055(16)
C(115)	0.017(3)	0.022(4)	0.016(3)	−0.004(3)	0.001(3)	−0.001(3)
C(117)	0.013(3)	0.013(4)	0.015(3)	−0.007(3)	−0.003(2)	−0.002(3)
C(118)	0.031(4)	0.022(4)	0.017(3)	−0.001(3)	−0.012(3)	0.000(3)
C(120)	0.018(3)	0.018(4)	0.020(3)	−0.001(3)	0.002(3)	−0.003(3)
C(121)	0.041(5)	0.028(4)	0.011(3)	0.002(3)	−0.002(3)	−0.008(4)
C(122)	0.140(3)	0.113(2)	0.0607(15)	0.0074(15)	0.0041(16)	−0.041(2)
C(123)	0.045(5)	0.036(5)	0.042(5)	0.009(4)	−0.017(4)	−0.012(4)
C(124)	0.026(4)	0.024(4)	0.023(4)	0.003(3)	−0.001(3)	−0.007(3)
C(125)	0.038(5)	0.032(5)	0.029(4)	0.002(3)	−0.012(4)	−0.004(4)
C(126)	0.118(4)	0.156(5)	0.058(3)	0.027(3)	−0.056(3)	−0.017(4)
C(127)	0.021(3)	0.013(4)	0.012(3)	−0.002(3)	−0.001(3)	−0.003(3)
C(128)	0.078(7)	0.035(6)	0.018(4)	−0.003(4)	−0.020(4)	0.030(5)
C(129)	0.106(4)	0.037(3)	0.170(5)	−0.031(3)	0.023(4)	0.017(2)
C(150)	0.140(3)	0.113(2)	0.0607(15)	0.0074(15)	0.0041(16)	−0.041(2)
C(151)	0.140(3)	0.113(2)	0.0607(15)	0.0074(15)	0.0041(16)	−0.041(2)
C(152)	0.140(3)	0.113(2)	0.0607(15)	0.0074(15)	0.0041(16)	−0.041(2)
C(153)	0.061(3)	0.101(5)	0.298(11)	−0.050(5)	−0.037(4)	−0.034(3)
C(154)	0.033(5)	0.016(5)	0.086(8)	−0.010(4)	−0.006(5)	−0.003(4)
F(250)	0.140(3)	0.113(2)	0.0607(15)	0.0074(15)	0.0041(16)	−0.041(2)

Table S44. Distances [Å] for [{PC(sp^3)(PMe_3)P}^{tBu}PdI][BAr^F_4] (**12**).

atom – atom	distance	atom – atom	distance
Pd – C	2.119(7)	Pd – P(1)	2.264(2)
Pd – P(2)	2.3279(18)	Pd – I	2.6340(7)
F(1) – C(108)	1.355(14)	B(1) – C(203)	1.639(11)
B(1) – C(102)	1.642(11)	B(1) – C(109)	1.645(11)
B(1) – C(117)	1.650(11)	C – C(21)	1.533(9)
C – C(11)	1.534(10)	C – P(3)	1.856(7)
F(9) – C(104)	1.325(9)	F(8) – C(104)	1.370(10)
F(7) – C(104)	1.313(10)	F(6) – C(111)	1.315(15)
F(5) – C(111)	1.329(14)	F(4) – C(111)	1.299(12)
F(3) – C(108)	1.378(19)	P(1) – C(101)	1.797(8)
P(1) – C(37)	1.838(8)	P(1) – C(40)	1.838(8)
F(11) – C(106)	1.313(13)	C(11) – C(101)	1.381(10)
C(11) – C(47)	1.395(10)	P(2) – C(100)	1.817(7)
P(2) – C(34)	1.831(8)	P(2) – C(31)	1.853(9)
F(12) – C(106)	1.354(13)	C(21) – C(56)	1.383(11)
C(21) – C(100)	1.418(10)	P(3) – C(15)	1.782(9)
P(3) – C(14)	1.786(9)	P(3) – C(13)	1.794(8)
F(13) – C(106)	1.338(10)	C(13) – H(13A)	0.9800
C(13) – H(13B)	0.9800	C(13) – H(13C)	0.9800
F(14) – C(114)	1.322(13)	C(14) – H(14A)	0.9800
C(14) – H(14B)	0.9800	C(14) – H(14C)	0.9800
C(15) – H(15A)	0.9800	C(15) – H(15B)	0.9800
C(15) – H(15C)	0.9800	F(15) – C(114)	1.344(13)
C(200) – C(117)	1.389(10)	C(200) – C(115)	1.391(11)
C(200) – H(200)	0.9500	F(16) – C(114)	1.338(13)
C(16) – C(152)	1.34(2)	C(16) – C(210)	1.40(2)
C(16) – H(16)	0.9500	C(017) – C(112)	1.383(10)
C(017) – C(117)	1.390(9)	C(017) – H(017)	0.9500
F(17) – C(113)	1.335(8)	C(17) – C(152)	1.36(2)
C(17) – C(208)	1.39(2)	C(17) – H(17)	0.9500
C(018) – C(205)	1.355(11)	C(018) – C(204)	1.408(12)
C(018) – H(018)	0.9500	F(18) – C(113)	1.345(8)
C(18) – C(208)	1.35(2)	C(18) – C(210)	1.39(2)
C(18) – H(18)	0.9500	F(19) – C(113)	1.324(8)
C(201) – C(115)	1.382(10)	C(201) – C(112)	1.392(9)
C(201) – H(201)	0.9500	C(202) – C(204)	1.351(11)
C(202) – C(203)	1.399(11)	C(202) – H(202)	0.9500
C(203) – C(206)	1.392(11)	P(21) – C(82)	1.807(7)
P(21) – C(85)	1.825(9)	P(21) – C(87)	1.845(9)
P(21) – Pd(2)	2.2636(18)	F(21) – C(209)	1.300(19)
F(20) – C(209)	1.41(2)	G(21 – C(82)	1.386(10)
G(21 – C(83)	1.399(10)	G(21 – C(2)	1.525(9)
P(22) – C(73)	1.830(8)	P(22) – C(64)	1.837(8)
P(22) – C(61)	1.852(8)	P(22) – Pd(2)	2.3231(19)
F(22) – C(209)	1.276(19)	C(22) – C(73)	1.380(10)
C(22) – C(74)	1.394(10)	C(22) – C(2)	1.549(10)
P(23) – C(25)	1.765(7)	P(23) – C(24)	1.777(8)
P(23) – C(23)	1.807(8)	P(23) – C(2)	1.853(7)
C(23) – H(23A)	0.9800	C(23) – H(23B)	0.9800
C(23) – H(23C)	0.9800	C(24) – H(24A)	0.9800

Continued on next page

Table S44. – continued from previous page

atom – atom	distance	atom – atom	distance
C(24) – H(24B)	0.9800	C(24) – H(24C)	0.9800
F(24) – C(129)	1.26(2)	C(25) – H(25A)	0.9800
C(25) – H(25B)	0.9800	C(25) – H(25C)	0.9800
F(25) – C(129)	1.39(2)	C(27) – C(105)	1.356(11)
C(27) – C(103)	1.362(11)	C(27) – H(27)	0.9500
F(27) – C(154)	1.329(12)	C(26) – C(103)	1.387(11)
C(26) – C(102)	1.390(11)	C(26) – H(26)	0.9500
F(26) – C(129)	1.31(2)	C(204) – C(209)	1.448(15)
F(28) – C(154)	1.287(12)	C(28) – C(102)	1.405(10)
C(28) – C(105)	1.410(11)	C(28) – H(28)	0.9500
Pd(2) – C(2)	2.129(7)	Pd(2) – I(2)	2.6398(7)
F(2) – C(108)	1.329(17)	B(2) – C(120)	1.619(11)
B(2) – C(211)	1.641(12)	B(2) – C(127)	1.647(10)
B(2) – C(208)	1.663(18)	F(29) – C(154)	1.355(14)
C(205) – C(206)	1.414(11)	C(205) – C(129)	1.463(15)
C(206) – H(206)	0.9500	F(30) – C(123)	1.303(11)
C(31) – C(32)	1.487(12)	C(31) – C(33)	1.545(11)
C(31) – H(31)	1.0000	F(31) – C(123)	1.364(13)
C(32) – H(32A)	0.9800	C(32) – H(32B)	0.9800
C(32) – H(32C)	0.9800	F(32) – C(123)	1.275(11)
C(33) – H(33A)	0.9800	C(33) – H(33B)	0.9800
C(33) – H(33C)	0.9800	F(33) – C(128)	1.337(11)
C(34) – C(35)	1.528(12)	C(34) – C(36)	1.532(11)
C(34) – H(34)	1.0000	F(34) – C(128)	1.297(12)
C(207) – C(110)	1.386(11)	C(207) – C(109)	1.408(12)
C(207) – H(207)	0.9500	F(36) – C(122)	1.32(2)
C(36) – H(36A)	0.9800	C(36) – H(36B)	0.9800
C(36) – H(36C)	0.9800	C(35) – H(35A)	0.9800
C(35) – H(35B)	0.9800	C(35) – H(35C)	0.9800
F(35) – C(128)	1.349(12)	C(37) – C(38)	1.482(14)
C(37) – C(39)	1.540(12)	C(37) – H(37)	1.0000
F(37) – C(122)	1.29(2)	C(38) – H(38A)	0.9800
C(38) – H(38B)	0.9800	C(38) – H(38C)	0.9800
F(38) – C(122)	1.27(2)	C(39) – H(39A)	0.9800
C(39) – H(39B)	0.9800	C(39) – H(39C)	0.9800
F(39) – C(153)	1.27(2)	C(210) – C(150)	1.48(2)
C(211) – C(94)	1.390(12)	C(211) – C(96)	1.391(12)
C(40) – C(42)	1.487(13)	C(40) – C(41)	1.556(11)
C(40) – H(40)	1.0000	F(40) – C(153)	1.35(3)
C(212) – C(94)	1.402(12)	C(212) – C(95)	1.417(15)
C(212) – C(154)	1.535(13)	C(42) – H(42A)	0.9500
C(42) – H(42B)	0.9500	C(41) – H(41A)	0.9800
C(41) – H(41B)	0.9800	C(41) – H(41C)	0.9800
F(41) – C(153)	1.24(3)	C(44) – C(45)	1.357(12)
C(44) – C(101)	1.413(11)	C(44) – H(44)	0.9500
F(44) – C(151)	1.49(2)	F(43) – C(151)	1.201(18)
C(45) – C(46)	1.428(11)	C(45) – C(48)	1.524(11)
F(45) – C(151)	1.246(17)	C(46) – C(47)	1.368(11)
C(46) – H(46)	0.9500	F(46) – C(150)	1.37(2)
F(46) – F(47)	1.744(16)	C(213) – C(107)	1.417(11)
C(213) – C(109)	1.430(12)	C(213) – H(213)	0.9500

Continued on next page

Table S44. – continued from previous page

atom – atom	distance	atom – atom	distance
C(47) – H(47)	0.9500	F(47) – C(150)	1.30(2)
C(48) – C(51)	1.522(16)	C(48) – C(50)	1.533(18)
C(48) – C(49)	1.585(18)	C(49) – H(49A)	0.9800
C(49) – H(49B)	0.9800	C(49) – H(49C)	0.9800
C(214) – C(95)	1.329(15)	C(214) – C(153)	1.41(2)
C(214) – C(96)	1.414(12)	C(50) – H(50A)	0.9800
C(50) – H(50B)	0.9800	C(50) – H(50C)	0.9800
C(51) – H(51A)	0.9800	C(51) – H(51B)	0.9800
C(51) – H(51C)	0.9800	C(53) – C(100)	1.379(10)
C(53) – C(54)	1.398(10)	C(53) – H(53)	0.9500
C(54) – C(55)	1.384(11)	C(54) – C(57)	1.526(10)
C(56) – C(55)	1.373(11)	C(56) – H(56)	0.9500
C(55) – H(55)	0.9500	C(57) – C(60)	1.520(11)
C(57) – C(59)	1.523(11)	C(57) – C(58)	1.523(11)
C(58) – H(58A)	0.9800	C(58) – H(58B)	0.9800
C(58) – H(58C)	0.9800	C(59) – H(59A)	0.9800
C(59) – H(59B)	0.9800	C(59) – H(59C)	0.9800
C(215) – C(107)	1.356(16)	C(215) – C(110)	1.389(16)
C(215) – H(215)	0.9500	C(60) – H(60A)	0.9800
C(60) – H(60B)	0.9800	C(60) – H(60C)	0.9800
C(61) – C(63)	1.509(11)	C(61) – C(62)	1.547(11)
C(61) – H(61)	1.0000	C(62) – H(62A)	0.9800
C(62) – H(62B)	0.9800	C(62) – H(62C)	0.9800
C(63) – H(63A)	0.9800	C(63) – H(63B)	0.9800
C(63) – H(63C)	0.9800	F(63) – C(126)	1.29(2)
C(64) – C(65)	1.508(11)	C(64) – C(66)	1.518(13)
C(64) – H(64)	1.0000	F(64) – C(126)	1.34(2)
C(65) – H(65A)	0.9800	C(65) – H(65B)	0.9800
C(65) – H(65C)	0.9800	F(65) – C(126)	1.32(2)
C(66) – H(66A)	0.9800	C(66) – H(66B)	0.9800
C(66) – H(66C)	0.9800	C(67) – C(70)	1.596(15)
C(67) – H(67A)	0.9800	C(67) – H(67B)	0.9800
C(67) – H(67C)	0.9800	C(68) – C(70)	1.531(14)
C(68) – H(68A)	0.9800	C(68) – H(68B)	0.9800
C(68) – H(68C)	0.9800	C(69) – C(70)	1.479(16)
C(69) – H(69A)	0.9800	C(69) – H(69B)	0.9800
C(69) – H(69C)	0.9800	C(70) – C(71)	1.510(11)
C(71) – C(72)	1.374(12)	C(71) – C(75)	1.428(11)
C(72) – C(73)	1.378(11)	C(72) – H(72)	0.9500
C(74) – C(75)	1.379(11)	C(74) – H(74)	0.9500
C(75) – H(75)	0.9500	C(76) – C(79)	1.493(18)
C(76) – H(76A)	0.9800	C(76) – H(76B)	0.9800
C(76) – H(76C)	0.9800	C(77) – C(79)	1.510(17)
C(77) – H(77A)	0.9800	C(77) – H(77B)	0.9800
C(77) – H(77C)	0.9800	C(78) – C(79)	1.554(16)
C(78) – H(78A)	0.9800	C(78) – H(78B)	0.9800
C(78) – H(78C)	0.9800	C(79) – C(80)	1.536(10)
C(80) – C(81)	1.375(11)	C(80) – C(84)	1.378(11)
C(81) – C(82)	1.413(10)	C(81) – H(81)	0.9500
C(83) – C(84)	1.381(10)	C(83) – H(83)	0.9500
C(84) – H(84)	0.9500	C(85) – C(86)	1.501(14)

Continued on next page

Table S44. – continued from previous page

atom – atom	distance	atom – atom	distance
C(85) – C(90)	1.508(15)	C(85) – H(85)	1.0000
C(86) – H(86A)	0.9800	C(86) – H(86B)	0.9800
C(86) – H(86C)	0.9800	C(87) – C(89)	1.451(14)
C(87) – C(88)	1.499(14)	C(87) – H(87)	1.0000
C(88) – H(88A)	0.9500	C(88) – H(88B)	0.9500
C(89) – H(89A)	0.9800	C(89) – H(89B)	0.9800
C(89) – H(89C)	0.9800	C(90) – H(90A)	0.9800
C(90) – H(90B)	0.9800	C(90) – H(90C)	0.9800
C(91) – C(121)	1.361(13)	C(91) – C(118)	1.368(12)
C(91) – H(91)	0.9500	C(92) – C(121)	1.371(12)
C(92) – C(120)	1.388(10)	C(92) – H(92)	0.9500
C(93) – C(118)	1.370(11)	C(93) – C(120)	1.408(10)
C(93) – H(93)	0.9500	C(94) – H(94)	0.9500
C(95) – H(95)	0.9500	C(96) – H(96)	0.9500
C(97) – C(124)	1.371(11)	C(97) – C(127)	1.407(10)
C(97) – H(97)	0.9500	C(98) – C(124)	1.367(11)
C(98) – C(125)	1.379(12)	C(98) – H(98)	0.9500
C(99) – C(127)	1.358(10)	C(99) – C(125)	1.399(12)
C(99) – H(99)	0.9500	C(103) – C(104)	1.482(11)
C(105) – C(106)	1.492(11)	C(107) – C(108)	1.481(17)
C(110) – C(111)	1.459(16)	C(112) – C(113)	1.487(9)
C(114) – C(115)	1.465(11)	C(118) – C(128)	1.504(12)
C(121) – C(122)	1.473(18)	C(123) – C(124)	1.519(12)
C(125) – C(126)	1.434(15)	C(150) – F(250)	1.274(19)
C(151) – C(152)	1.48(2)		

Table S45. Angles [°] for [{PC(sp^3)(PMe_3)P}^{tBu}PdI][BAr^F_4] (**12**).

atom – atom – atom	angle	atom – atom – atom	angle
C – Pd – P(1)	85.6(2)	C – Pd – P(2)	86.13(19)
P(1) – Pd – P(2)	162.70(7)	C – Pd – I	177.58(19)
P(1) – Pd – I	91.96(5)	P(2) – Pd – I	96.27(5)
C(203) – B(1) – C(102)	108.0(6)	C(203) – B(1) – C(109)	110.2(6)
C(102) – B(1) – C(109)	111.9(6)	C(203) – B(1) – C(117)	111.0(6)
C(102) – B(1) – C(117)	108.7(6)	C(109) – B(1) – C(117)	107.0(6)
C(21) – C – C(11)	115.8(6)	C(21) – C – P(3)	107.6(5)
C(11) – C – P(3)	105.9(5)	C(21) – C – Pd	108.4(4)
C(11) – C – Pd	113.8(5)	P(3) – C – Pd	104.4(3)
C(101) – P(1) – C(37)	104.0(4)	C(101) – P(1) – C(40)	105.8(4)
C(37) – P(1) – C(40)	107.7(4)	C(101) – P(1) – Pd	103.0(3)
C(37) – P(1) – Pd	125.9(3)	C(40) – P(1) – Pd	108.7(3)
C(101) – C(11) – C(47)	116.9(7)	C(101) – C(11) – C	120.8(6)
C(47) – C(11) – C	122.0(6)	C(100) – P(2) – C(34)	100.8(3)
C(100) – P(2) – C(31)	108.9(4)	C(34) – P(2) – C(31)	105.8(4)
C(100) – P(2) – Pd	98.5(2)	C(34) – P(2) – Pd	116.7(3)
C(31) – P(2) – Pd	123.2(3)	C(56) – C(21) – C(100)	116.0(6)
C(56) – C(21) – C	124.5(7)	C(100) – C(21) – C	119.5(6)
C(15) – P(3) – C(14)	108.0(5)	C(15) – P(3) – C(13)	106.2(4)
C(14) – P(3) – C(13)	101.9(4)	C(15) – P(3) – C	111.2(4)
C(14) – P(3) – C	115.3(4)	C(13) – P(3) – C	113.4(4)
P(3) – C(13) – H(13A)	109.5	P(3) – C(13) – H(13B)	109.5
H(13A) – C(13) – H(13B)	109.5	P(3) – C(13) – H(13C)	109.5
H(13A) – C(13) – H(13C)	109.5	H(13B) – C(13) – H(13C)	109.5
P(3) – C(14) – H(14A)	109.5	P(3) – C(14) – H(14B)	109.5
H(14A) – C(14) – H(14B)	109.5	P(3) – C(14) – H(14C)	109.5
H(14A) – C(14) – H(14C)	109.5	H(14B) – C(14) – H(14C)	109.5
P(3) – C(15) – H(15A)	109.5	P(3) – C(15) – H(15B)	109.5
H(15A) – C(15) – H(15B)	109.5	P(3) – C(15) – H(15C)	109.5
H(15A) – C(15) – H(15C)	109.5	H(15B) – C(15) – H(15C)	109.5
C(117) – C(200) – C(115)	121.0(6)	C(117) – C(200) – H(200)	119.5
C(115) – C(200) – H(200)	119.5	C(152) – C(16) – C(210)	115.7(16)
C(152) – C(16) – H(16)	122.1	C(210) – C(16) – H(16)	122.1
C(112) – C(017) – C(117)	121.5(6)	C(112) – C(017) – H(017)	119.3
C(117) – C(017) – H(017)	119.3	C(152) – C(17) – C(208)	121.5(18)
C(152) – C(17) – H(17)	119.3	C(208) – C(17) – H(17)	119.3
C(205) – C(018) – C(204)	118.2(7)	C(205) – C(018) – H(018)	120.9
C(204) – C(018) – H(018)	120.9	C(208) – C(18) – C(210)	121.9(18)
C(208) – C(18) – H(18)	119.0	C(210) – C(18) – H(18)	119.0
C(115) – C(201) – C(112)	117.8(7)	C(115) – C(201) – H(201)	121.1
C(112) – C(201) – H(201)	121.1	C(204) – C(202) – C(203)	123.0(7)
C(204) – C(202) – H(202)	118.5	C(203) – C(202) – H(202)	118.5
C(206) – C(203) – C(202)	116.0(7)	C(206) – C(203) – B(1)	121.1(7)
C(202) – C(203) – B(1)	122.8(7)	C(82) – P(21) – C(85)	105.9(4)
C(82) – P(21) – C(87)	105.4(4)	C(85) – P(21) – C(87)	107.8(4)
C(82) – P(21) – Pd(2)	102.8(2)	C(85) – P(21) – Pd(2)	124.3(3)
C(87) – P(21) – Pd(2)	109.0(4)	C(82) – G(21 – C(83)	117.2(6)
C(82) – G(21 – C(2)	120.7(6)	C(83) – G(21 – C(2)	121.8(6)
C(73) – P(22) – C(64)	101.2(4)	C(73) – P(22) – C(61)	108.0(3)
C(64) – P(22) – C(61)	107.5(4)	C(73) – P(22) – Pd(2)	98.3(2)

Continued on next page

Table S45. – continued from previous page

atom – atom – atom	angle	atom – atom – atom	angle
C(64) – P(22) – Pd(2)	115.0(3)	C(61) – P(22) – Pd(2)	123.7(3)
C(73) – C(22) – C(74)	116.3(7)	C(73) – C(22) – C(2)	121.2(6)
C(74) – C(22) – C(2)	122.4(6)	C(25) – P(23) – C(24)	108.2(4)
C(25) – P(23) – C(23)	105.9(4)	C(24) – P(23) – C(23)	103.4(4)
C(25) – P(23) – C(2)	111.7(4)	C(24) – P(23) – C(2)	114.4(3)
C(23) – P(23) – C(2)	112.7(3)	P(23) – C(23) – H(23A)	109.5
P(23) – C(23) – H(23B)	109.5	H(23A) – C(23) – H(23B)	109.5
P(23) – C(23) – H(23C)	109.5	H(23A) – C(23) – H(23C)	109.5
H(23B) – C(23) – H(23C)	109.5	P(23) – C(24) – H(24A)	109.5
P(23) – C(24) – H(24B)	109.5	H(24A) – C(24) – H(24B)	109.5
P(23) – C(24) – H(24C)	109.5	H(24A) – C(24) – H(24C)	109.5
H(24B) – C(24) – H(24C)	109.5	P(23) – C(25) – H(25A)	109.5
P(23) – C(25) – H(25B)	109.5	H(25A) – C(25) – H(25B)	109.5
P(23) – C(25) – H(25C)	109.5	H(25A) – C(25) – H(25C)	109.5
H(25B) – C(25) – H(25C)	109.5	C(105) – C(27) – C(103)	119.5(7)
C(105) – C(27) – H(27)	120.3	C(103) – C(27) – H(27)	120.3
C(103) – C(26) – C(102)	121.9(7)	C(103) – C(26) – H(26)	119.1
C(102) – C(26) – H(26)	119.1	C(202) – C(204) – C(018)	120.6(7)
C(202) – C(204) – C(209)	120.2(10)	C(018) – C(204) – C(209)	119.2(10)
C(102) – C(28) – C(105)	119.4(7)	C(102) – C(28) – H(28)	120.3
C(105) – C(28) – H(28)	120.3	C(2) – Pd(2) – P(21)	85.69(19)
C(2) – Pd(2) – P(22)	85.98(19)	P(21) – Pd(2) – P(22)	163.48(7)
C(2) – Pd(2) – I(2)	177.6(2)	P(21) – Pd(2) – I(2)	92.28(5)
P(22) – Pd(2) – I(2)	96.29(5)	C(120) – B(2) – C(211)	111.0(6)
C(120) – B(2) – C(127)	110.0(6)	C(211) – B(2) – C(127)	107.4(6)
C(120) – B(2) – C(208)	108.9(9)	C(211) – B(2) – C(208)	109.9(9)
C(127) – B(2) – C(208)	109.6(8)	G(21 – C(2) – C(22)	116.9(6)
G(21 – C(2) – P(23)	105.8(5)	C(22) – C(2) – P(23)	109.5(5)
G(21 – C(2) – Pd(2)	114.0(4)	C(22) – C(2) – Pd(2)	106.6(4)
P(23) – C(2) – Pd(2)	103.2(3)	C(018) – C(205) – C(206)	120.9(7)
C(018) – C(205) – C(129)	121.8(10)	C(206) – C(205) – C(129)	117.3(10)
C(203) – C(206) – C(205)	121.1(7)	C(203) – C(206) – H(206)	119.4
C(205) – C(206) – H(206)	119.4	C(32) – C(31) – C(33)	111.4(7)
C(32) – C(31) – P(2)	112.9(7)	C(33) – C(31) – P(2)	114.7(6)
C(32) – C(31) – H(31)	105.6	C(33) – C(31) – H(31)	105.6
P(2) – C(31) – H(31)	105.6	C(31) – C(32) – H(32A)	109.5
C(31) – C(32) – H(32B)	109.5	H(32A) – C(32) – H(32B)	109.5
C(31) – C(32) – H(32C)	109.5	H(32A) – C(32) – H(32C)	109.5
H(32B) – C(32) – H(32C)	109.5	C(31) – C(33) – H(33A)	109.5
C(31) – C(33) – H(33B)	109.5	H(33A) – C(33) – H(33B)	109.5
C(31) – C(33) – H(33C)	109.5	H(33A) – C(33) – H(33C)	109.5
H(33B) – C(33) – H(33C)	109.5	C(35) – C(34) – C(36)	110.6(7)
C(35) – C(34) – P(2)	110.9(5)	C(36) – C(34) – P(2)	116.1(6)
C(35) – C(34) – H(34)	106.2	C(36) – C(34) – H(34)	106.2
P(2) – C(34) – H(34)	106.2	C(110) – C(207) – C(109)	121.3(8)
C(110) – C(207) – H(207)	119.4	C(109) – C(207) – H(207)	119.4
C(34) – C(36) – H(36A)	109.5	C(34) – C(36) – H(36B)	109.5
H(36A) – C(36) – H(36B)	109.5	C(34) – C(36) – H(36C)	109.5
H(36A) – C(36) – H(36C)	109.5	H(36B) – C(36) – H(36C)	109.5
C(34) – C(35) – H(35A)	109.5	C(34) – C(35) – H(35B)	109.5
H(35A) – C(35) – H(35B)	109.5	C(34) – C(35) – H(35C)	109.5

Continued on next page

Table S45. – continued from previous page

atom – atom – atom	angle	atom – atom – atom	angle
H(35A) – C(35) – H(35C)	109.5	H(35B) – C(35) – H(35C)	109.5
C(38) – C(37) – C(39)	109.3(8)	C(38) – C(37) – P(1)	110.8(6)
C(39) – C(37) – P(1)	112.8(6)	C(38) – C(37) – H(37)	107.9
C(39) – C(37) – H(37)	107.9	P(1) – C(37) – H(37)	107.9
C(37) – C(38) – H(38A)	109.5	C(37) – C(38) – H(38B)	109.5
H(38A) – C(38) – H(38B)	109.5	C(37) – C(38) – H(38C)	109.5
H(38A) – C(38) – H(38C)	109.5	H(38B) – C(38) – H(38C)	109.5
C(37) – C(39) – H(39A)	109.5	C(37) – C(39) – H(39B)	109.5
H(39A) – C(39) – H(39B)	109.5	C(37) – C(39) – H(39C)	109.5
H(39A) – C(39) – H(39C)	109.5	H(39B) – C(39) – H(39C)	109.5
C(18) – C(208) – C(17)	116.2(16)	C(18) – C(208) – B(2)	124.3(15)
C(17) – C(208) – B(2)	119.5(14)	F(22) – C(209) – F(21)	118.4(14)
F(22) – C(209) – F(20)	94.3(12)	F(21) – C(209) – F(20)	95.4(13)
F(22) – C(209) – C(204)	117.3(13)	F(21) – C(209) – C(204)	115.4(13)
F(20) – C(209) – C(204)	110.4(14)	C(18) – C(210) – C(16)	121.3(18)
C(18) – C(210) – C(150)	121.3(17)	C(16) – C(210) – C(150)	117.2(15)
C(94) – C(211) – C(96)	115.3(8)	C(94) – C(211) – B(2)	124.2(7)
C(96) – C(211) – B(2)	120.5(7)	C(42) – C(40) – C(41)	110.1(8)
C(42) – C(40) – P(1)	109.7(6)	C(41) – C(40) – P(1)	114.8(6)
C(42) – C(40) – H(40)	107.3	C(41) – C(40) – H(40)	107.3
P(1) – C(40) – H(40)	107.3	C(94) – C(212) – C(95)	120.1(9)
C(94) – C(212) – C(154)	120.2(8)	C(95) – C(212) – C(154)	119.6(8)
C(40) – C(42) – H(42A)	120.0	C(40) – C(42) – H(42B)	120.0
H(42A) – C(42) – H(42B)	120.0	C(40) – C(41) – H(41A)	109.5
C(40) – C(41) – H(41B)	109.5	H(41A) – C(41) – H(41B)	109.5
C(40) – C(41) – H(41C)	109.5	H(41A) – C(41) – H(41C)	109.5
H(41B) – C(41) – H(41C)	109.5	C(45) – C(44) – C(101)	123.0(8)
C(45) – C(44) – H(44)	118.5	C(101) – C(44) – H(44)	118.5
C(44) – C(45) – C(46)	116.3(7)	C(44) – C(45) – C(48)	121.9(8)
C(46) – C(45) – C(48)	121.8(7)	C(47) – C(46) – C(45)	120.6(7)
C(47) – C(46) – H(46)	119.7	C(45) – C(46) – H(46)	119.7
C(150) – F(46) – F(47)	47.6(10)	C(107) – C(213) – C(109)	118.3(8)
C(107) – C(213) – H(213)	120.9	C(109) – C(213) – H(213)	120.9
C(46) – C(47) – C(11)	122.8(7)	C(46) – C(47) – H(47)	118.6
C(11) – C(47) – H(47)	118.6	C(150) – F(47) – F(46)	51.1(10)
C(51) – C(48) – C(45)	111.8(8)	C(51) – C(48) – C(50)	112.5(10)
C(45) – C(48) – C(50)	112.4(8)	C(51) – C(48) – C(49)	107.3(10)
C(45) – C(48) – C(49)	105.0(8)	C(50) – C(48) – C(49)	107.4(9)
C(48) – C(49) – H(49A)	109.5	C(48) – C(49) – H(49B)	109.5
H(49A) – C(49) – H(49B)	109.5	C(48) – C(49) – H(49C)	109.5
H(49A) – C(49) – H(49C)	109.5	H(49B) – C(49) – H(49C)	109.5
C(95) – C(214) – C(153)	117.2(13)	C(95) – C(214) – C(96)	123.0(10)
C(153) – C(214) – C(96)	119.7(13)	C(48) – C(50) – H(50A)	109.5
C(48) – C(50) – H(50B)	109.5	H(50A) – C(50) – H(50B)	109.5
C(48) – C(50) – H(50C)	109.5	H(50A) – C(50) – H(50C)	109.5
H(50B) – C(50) – H(50C)	109.5	C(48) – C(51) – H(51A)	109.5
C(48) – C(51) – H(51B)	109.5	H(51A) – C(51) – H(51B)	109.5
C(48) – C(51) – H(51C)	109.5	H(51A) – C(51) – H(51C)	109.5
H(51B) – C(51) – H(51C)	109.5	C(100) – C(53) – C(54)	121.7(7)
C(100) – C(53) – H(53)	119.1	C(54) – C(53) – H(53)	119.1
C(55) – C(54) – C(53)	116.5(7)	C(55) – C(54) – C(57)	123.7(7)

Continued on next page

Table S45. – continued from previous page

atom – atom – atom	angle	atom – atom – atom	angle
C(53) – C(54) – C(57)	119.8(7)	C(55) – C(56) – C(21)	122.3(7)
C(55) – C(56) – H(56)	118.9	C(21) – C(56) – H(56)	118.9
C(56) – C(55) – C(54)	121.8(7)	C(56) – C(55) – H(55)	119.1
C(54) – C(55) – H(55)	119.1	C(60) – C(57) – C(59)	107.4(7)
C(60) – C(57) – C(58)	109.5(7)	C(59) – C(57) – C(58)	109.6(7)
C(60) – C(57) – C(54)	112.0(7)	C(59) – C(57) – C(54)	111.1(6)
C(58) – C(57) – C(54)	107.3(6)	C(57) – C(58) – H(58A)	109.5
C(57) – C(58) – H(58B)	109.5	H(58A) – C(58) – H(58B)	109.5
C(57) – C(58) – H(58C)	109.5	H(58A) – C(58) – H(58C)	109.5
H(58B) – C(58) – H(58C)	109.5	C(57) – C(59) – H(59A)	109.5
C(57) – C(59) – H(59B)	109.5	H(59A) – C(59) – H(59B)	109.5
C(57) – C(59) – H(59C)	109.5	H(59A) – C(59) – H(59C)	109.5
H(59B) – C(59) – H(59C)	109.5	C(107) – C(215) – C(110)	119.4(8)
C(107) – C(215) – H(215)	120.3	C(110) – C(215) – H(215)	120.3
C(57) – C(60) – H(60A)	109.5	C(57) – C(60) – H(60B)	109.5
H(60A) – C(60) – H(60B)	109.5	C(57) – C(60) – H(60C)	109.5
H(60A) – C(60) – H(60C)	109.5	H(60B) – C(60) – H(60C)	109.5
C(63) – C(61) – C(62)	110.9(6)	C(63) – C(61) – P(22)	117.2(6)
C(62) – C(61) – P(22)	110.4(5)	C(63) – C(61) – H(61)	105.9
C(62) – C(61) – H(61)	105.9	P(22) – C(61) – H(61)	105.9
C(61) – C(62) – H(62A)	109.5	C(61) – C(62) – H(62B)	109.5
H(62A) – C(62) – H(62B)	109.5	C(61) – C(62) – H(62C)	109.5
H(62A) – C(62) – H(62C)	109.5	H(62B) – C(62) – H(62C)	109.5
C(61) – C(63) – H(63A)	109.5	C(61) – C(63) – H(63B)	109.5
H(63A) – C(63) – H(63B)	109.5	C(61) – C(63) – H(63C)	109.5
H(63A) – C(63) – H(63C)	109.5	H(63B) – C(63) – H(63C)	109.5
C(65) – C(64) – C(66)	110.7(8)	C(65) – C(64) – P(22)	116.0(6)
C(66) – C(64) – P(22)	110.9(6)	C(65) – C(64) – H(64)	106.2
C(66) – C(64) – H(64)	106.2	P(22) – C(64) – H(64)	106.2
C(64) – C(65) – H(65A)	109.5	C(64) – C(65) – H(65B)	109.5
H(65A) – C(65) – H(65B)	109.5	C(64) – C(65) – H(65C)	109.5
H(65A) – C(65) – H(65C)	109.5	H(65B) – C(65) – H(65C)	109.5
C(64) – C(66) – H(66A)	109.5	C(64) – C(66) – H(66B)	109.5
H(66A) – C(66) – H(66B)	109.5	C(64) – C(66) – H(66C)	109.5
H(66A) – C(66) – H(66C)	109.5	H(66B) – C(66) – H(66C)	109.5
C(70) – C(67) – H(67A)	109.5	C(70) – C(67) – H(67B)	109.5
H(67A) – C(67) – H(67B)	109.5	C(70) – C(67) – H(67C)	109.5
H(67A) – C(67) – H(67C)	109.5	H(67B) – C(67) – H(67C)	109.5
C(70) – C(68) – H(68A)	109.5	C(70) – C(68) – H(68B)	109.5
H(68A) – C(68) – H(68B)	109.5	C(70) – C(68) – H(68C)	109.5
H(68A) – C(68) – H(68C)	109.5	H(68B) – C(68) – H(68C)	109.5
C(70) – C(69) – H(69A)	109.5	C(70) – C(69) – H(69B)	109.5
H(69A) – C(69) – H(69B)	109.5	C(70) – C(69) – H(69C)	109.5
H(69A) – C(69) – H(69C)	109.5	H(69B) – C(69) – H(69C)	109.5
C(69) – C(70) – C(71)	115.1(8)	C(69) – C(70) – C(68)	115.4(12)
C(71) – C(70) – C(68)	109.6(8)	C(69) – C(70) – C(67)	104.8(11)
C(71) – C(70) – C(67)	107.4(7)	C(68) – C(70) – C(67)	103.5(8)
C(72) – C(71) – C(75)	116.1(7)	C(72) – C(71) – C(70)	123.6(7)
C(75) – C(71) – C(70)	120.2(7)	C(71) – C(72) – C(73)	122.5(7)
C(71) – C(72) – H(72)	118.8	C(73) – C(72) – H(72)	118.8
C(72) – C(73) – C(22)	121.9(7)	C(72) – C(73) – P(22)	121.4(6)

Continued on next page

Table S45. – continued from previous page

atom – atom – atom	angle	atom – atom – atom	angle
C(22) – C(73) – P(22)	115.9(6)	C(75) – C(74) – C(22)	122.5(7)
C(75) – C(74) – H(74)	118.8	C(22) – C(74) – H(74)	118.8
C(74) – C(75) – C(71)	120.1(7)	C(74) – C(75) – H(75)	119.9
C(71) – C(75) – H(75)	119.9	C(79) – C(76) – H(76A)	109.5
C(79) – C(76) – H(76B)	109.5	H(76A) – C(76) – H(76B)	109.5
C(79) – C(76) – H(76C)	109.5	H(76A) – C(76) – H(76C)	109.5
H(76B) – C(76) – H(76C)	109.5	C(79) – C(77) – H(77A)	109.5
C(79) – C(77) – H(77B)	109.5	H(77A) – C(77) – H(77B)	109.5
C(79) – C(77) – H(77C)	109.5	H(77A) – C(77) – H(77C)	109.5
H(77B) – C(77) – H(77C)	109.5	C(79) – C(78) – H(78A)	109.5
C(79) – C(78) – H(78B)	109.5	H(78A) – C(78) – H(78B)	109.5
C(79) – C(78) – H(78C)	109.5	H(78A) – C(78) – H(78C)	109.5
H(78B) – C(78) – H(78C)	109.5	C(76) – C(79) – C(77)	115.7(10)
C(76) – C(79) – C(80)	108.0(8)	C(77) – C(79) – C(80)	108.7(8)
C(76) – C(79) – C(78)	107.3(10)	C(77) – C(79) – C(78)	106.3(10)
C(80) – C(79) – C(78)	110.9(8)	C(81) – C(80) – C(84)	116.6(6)
C(81) – C(80) – C(79)	121.9(7)	C(84) – C(80) – C(79)	121.6(7)
C(80) – C(81) – C(82)	121.2(7)	C(80) – C(81) – H(81)	119.4
C(82) – C(81) – H(81)	119.4	G(21 – C(82) – C(81)	121.3(7)
G(21 – C(82) – P(21)	116.5(5)	C(81) – C(82) – P(21)	122.3(5)
C(84) – C(83) – G(21	120.0(7)	C(84) – C(83) – H(83)	120.0
G(21 – C(83) – H(83)	120.0	C(80) – C(84) – C(83)	123.7(7)
C(80) – C(84) – H(84)	118.2	C(83) – C(84) – H(84)	118.2
C(86) – C(85) – C(90)	112.7(9)	C(86) – C(85) – P(21)	114.6(7)
C(90) – C(85) – P(21)	110.6(6)	C(86) – C(85) – H(85)	106.0
C(90) – C(85) – H(85)	106.0	P(21) – C(85) – H(85)	106.0
C(85) – C(86) – H(86A)	109.5	C(85) – C(86) – H(86B)	109.5
H(86A) – C(86) – H(86B)	109.5	C(85) – C(86) – H(86C)	109.5
H(86A) – C(86) – H(86C)	109.5	H(86B) – C(86) – H(86C)	109.5
C(89) – C(87) – C(88)	109.8(8)	C(89) – C(87) – P(21)	119.6(9)
C(88) – C(87) – P(21)	108.9(6)	C(89) – C(87) – H(87)	105.9
C(88) – C(87) – H(87)	105.9	P(21) – C(87) – H(87)	105.9
C(87) – C(88) – H(88A)	120.0	C(87) – C(88) – H(88B)	120.0
H(88A) – C(88) – H(88B)	120.0	C(87) – C(89) – H(89A)	109.5
C(87) – C(89) – H(89B)	109.5	H(89A) – C(89) – H(89B)	109.5
C(87) – C(89) – H(89C)	109.5	H(89A) – C(89) – H(89C)	109.5
H(89B) – C(89) – H(89C)	109.5	C(85) – C(90) – H(90A)	109.5
C(85) – C(90) – H(90B)	109.5	H(90A) – C(90) – H(90B)	109.5
C(85) – C(90) – H(90C)	109.5	H(90A) – C(90) – H(90C)	109.5
H(90B) – C(90) – H(90C)	109.5	C(121) – C(91) – C(118)	119.5(8)
C(121) – C(91) – H(91)	120.3	C(118) – C(91) – H(91)	120.3
C(121) – C(92) – C(120)	124.9(7)	C(121) – C(92) – H(92)	117.6
C(120) – C(92) – H(92)	117.6	C(118) – C(93) – C(120)	123.1(7)
C(118) – C(93) – H(93)	118.4	C(120) – C(93) – H(93)	118.4
C(211) – C(94) – C(212)	122.8(8)	C(211) – C(94) – H(94)	118.6
C(212) – C(94) – H(94)	118.6	C(214) – C(95) – C(212)	117.1(9)
C(214) – C(95) – H(95)	121.5	C(212) – C(95) – H(95)	121.5
C(211) – C(96) – C(214)	121.5(9)	C(211) – C(96) – H(96)	119.2
C(214) – C(96) – H(96)	119.2	C(124) – C(97) – C(127)	122.5(7)
C(124) – C(97) – H(97)	118.8	C(127) – C(97) – H(97)	118.8
C(124) – C(98) – C(125)	116.4(8)	C(124) – C(98) – H(98)	121.8

Continued on next page

Table S45. – continued from previous page

atom – atom – atom	angle	atom – atom – atom	angle
C(125) – C(98) – H(98)	121.8	C(127) – C(99) – C(125)	122.5(7)
C(127) – C(99) – H(99)	118.7	C(125) – C(99) – H(99)	118.7
C(53) – C(100) – C(21)	120.8(6)	C(53) – C(100) – P(2)	122.0(5)
C(21) – C(100) – P(2)	116.5(5)	C(11) – C(101) – C(44)	120.3(7)
C(11) – C(101) – P(1)	116.4(6)	C(44) – C(101) – P(1)	123.1(6)
C(26) – C(102) – C(28)	117.2(7)	C(26) – C(102) – B(1)	121.4(6)
C(28) – C(102) – B(1)	121.3(6)	C(27) – C(103) – C(26)	120.4(7)
C(27) – C(103) – C(104)	121.6(7)	C(26) – C(103) – C(104)	118.0(7)
F(7) – C(104) – F(9)	109.1(7)	F(7) – C(104) – F(8)	103.8(7)
F(9) – C(104) – F(8)	103.9(6)	F(7) – C(104) – C(103)	114.4(7)
F(9) – C(104) – C(103)	114.2(7)	F(8) – C(104) – C(103)	110.5(7)
C(27) – C(105) – C(28)	121.6(7)	C(27) – C(105) – C(106)	121.6(7)
C(28) – C(105) – C(106)	116.6(7)	F(11) – C(106) – F(13)	107.3(8)
F(11) – C(106) – F(12)	102.1(8)	F(13) – C(106) – F(12)	106.3(8)
F(11) – C(106) – C(105)	115.5(8)	F(13) – C(106) – C(105)	112.5(7)
F(12) – C(106) – C(105)	112.2(8)	C(215) – C(107) – C(213)	122.4(9)
C(215) – C(107) – C(108)	122.2(9)	C(213) – C(107) – C(108)	115.3(10)
F(2) – C(108) – F(1)	105.6(10)	F(2) – C(108) – F(3)	104.4(14)
F(1) – C(108) – F(3)	105.1(12)	F(2) – C(108) – C(107)	113.5(12)
F(1) – C(108) – C(107)	111.6(13)	F(3) – C(108) – C(107)	115.7(11)
C(207) – C(109) – C(213)	117.9(7)	C(207) – C(109) – B(1)	122.9(7)
C(213) – C(109) – B(1)	119.1(7)	C(207) – C(110) – C(215)	120.6(9)
C(207) – C(110) – C(111)	119.5(10)	C(215) – C(110) – C(111)	119.6(9)
F(4) – C(111) – F(6)	106.1(10)	F(4) – C(111) – F(5)	100.3(9)
F(6) – C(111) – F(5)	107.7(11)	F(4) – C(111) – C(110)	116.3(11)
F(6) – C(111) – C(110)	113.5(10)	F(5) – C(111) – C(110)	111.8(10)
C(017) – C(112) – C(201)	120.9(6)	C(017) – C(112) – C(113)	121.0(6)
C(201) – C(112) – C(113)	118.0(6)	F(19) – C(113) – F(17)	107.7(6)
F(19) – C(113) – F(18)	104.9(5)	F(17) – C(113) – F(18)	105.1(6)
F(19) – C(113) – C(112)	114.4(6)	F(17) – C(113) – C(112)	112.4(6)
F(18) – C(113) – C(112)	111.6(6)	F(14) – C(114) – F(16)	105.6(9)
F(14) – C(114) – F(15)	105.9(8)	F(16) – C(114) – F(15)	102.9(8)
F(14) – C(114) – C(115)	115.4(9)	F(16) – C(114) – C(115)	112.9(8)
F(15) – C(114) – C(115)	113.0(9)	C(201) – C(115) – C(200)	121.3(6)
C(201) – C(115) – C(114)	118.7(8)	C(200) – C(115) – C(114)	119.9(7)
C(200) – C(117) – C(017)	117.5(6)	C(200) – C(117) – B(1)	122.1(6)
C(017) – C(117) – B(1)	120.4(6)	C(91) – C(118) – C(93)	120.3(8)
C(91) – C(118) – C(128)	121.2(8)	C(93) – C(118) – C(128)	118.4(7)
C(92) – C(120) – C(93)	113.0(7)	C(92) – C(120) – B(2)	124.7(7)
C(93) – C(120) – B(2)	122.1(6)	C(91) – C(121) – C(92)	119.2(7)
C(91) – C(121) – C(122)	120.5(12)	C(92) – C(121) – C(122)	120.3(12)
F(38) – C(122) – F(37)	109.0(18)	F(38) – C(122) – F(36)	99.9(13)
F(37) – C(122) – F(36)	106.3(15)	F(38) – C(122) – C(121)	114.8(14)
F(37) – C(122) – C(121)	112.9(13)	F(36) – C(122) – C(121)	112.8(17)
F(32) – C(123) – F(30)	110.6(9)	F(32) – C(123) – F(31)	106.7(8)
F(30) – C(123) – F(31)	102.5(8)	F(32) – C(123) – C(124)	113.8(8)
F(30) – C(123) – C(124)	112.8(8)	F(31) – C(123) – C(124)	109.6(8)
C(98) – C(124) – C(97)	122.0(7)	C(98) – C(124) – C(123)	118.8(7)
C(97) – C(124) – C(123)	119.2(7)	C(98) – C(125) – C(99)	121.6(7)
C(98) – C(125) – C(126)	117.1(11)	C(99) – C(125) – C(126)	121.3(11)
F(63) – C(126) – F(65)	100.5(15)	F(63) – C(126) – F(64)	105.7(14)

Continued on next page

Table S45. – continued from previous page

atom – atom – atom	angle	atom – atom – atom	angle
F(65) – C(126) – F(64)	101.4(14)	F(63) – C(126) – C(125)	118.5(15)
F(65) – C(126) – C(125)	114.4(15)	F(64) – C(126) – C(125)	114.2(15)
C(99) – C(127) – C(97)	115.0(6)	C(99) – C(127) – B(2)	123.5(6)
C(97) – C(127) – B(2)	121.4(6)	F(34) – C(128) – F(33)	106.1(7)
F(34) – C(128) – F(35)	110.0(8)	F(33) – C(128) – F(35)	102.3(9)
F(34) – C(128) – C(118)	111.2(9)	F(33) – C(128) – C(118)	114.4(7)
F(35) – C(128) – C(118)	112.4(7)	F(24) – C(129) – F(26)	120.9(16)
F(24) – C(129) – F(25)	99.3(14)	F(26) – C(129) – F(25)	89.8(12)
F(24) – C(129) – C(205)	118.6(14)	F(26) – C(129) – C(205)	111.7(14)
F(25) – C(129) – C(205)	110.8(15)	F(250) – C(150) – F(47)	94.0(17)
F(250) – C(150) – F(46)	131.3(15)	F(47) – C(150) – F(46)	81.3(13)
F(250) – C(150) – C(210)	113.0(16)	F(47) – C(150) – C(210)	108.4(16)
F(46) – C(150) – C(210)	114.5(16)	F(43) – C(151) – F(45)	115.3(16)
F(43) – C(151) – C(152)	120.2(15)	F(45) – C(151) – C(152)	120.9(16)
F(43) – C(151) – F(44)	90.9(15)	F(45) – C(151) – F(44)	91.7(14)
C(152) – C(151) – F(44)	105.9(16)	C(16) – C(152) – C(17)	123.4(18)
C(16) – C(152) – C(151)	115.0(15)	C(17) – C(152) – C(151)	121.6(17)
F(41) – C(153) – F(39)	100(2)	F(41) – C(153) – F(40)	104(2)
F(39) – C(153) – F(40)	101.0(19)	F(41) – C(153) – C(214)	115(2)
F(39) – C(153) – C(214)	119(2)	F(40) – C(153) – C(214)	116(2)
F(28) – C(154) – F(27)	106.2(9)	F(28) – C(154) – F(29)	114.5(10)
F(27) – C(154) – F(29)	104.1(9)	F(28) – C(154) – C(212)	110.9(8)
F(27) – C(154) – C(212)	109.0(9)	F(29) – C(154) – C(212)	111.7(9)
